# Planarity Is Not Plain: Closed- vs Open-Shell Reactivity of a Structurally Constrained, Doubly Reduced Arylborane toward Fluorobenzenes

**DOI:** 10.1021/jacs.5c05588

**Published:** 2025-05-29

**Authors:** Christoph D. Buch, Alexander Virovets, Eugenia Peresypkina, Burkhard Endeward, Hans-Wolfram Lerner, Felipe Fantuzzi, Shigehiro Yamaguchi, Matthias Wagner

**Affiliations:** † Institut für Anorganische und Analytische Chemie, 9173 Goethe-Universität Frankfurt, Max-von-Laue-Straße 7, D-60438 Frankfurt am Main, Germany; ‡ School of Chemistry and Forensic Science, 2240 University of Kent, Park Wood Rd, Canterbury CT2 7NH, U.K.; § Department of Chemistry, Graduate School of Science, and Integrated Research Consortium on Chemical Sciences (IRCCS), 12965 Nagoya University, Furo, Chikusa, Nagoya 464-8602, Japan

## Abstract

The ability to activate small molecules is imparted to 9,10-dihydro-9,10-diboraanthracenes (DBAs) through the injection of two electrons. We report on the activation of fluorobenzenes C_6_F_*n*_H_6–*n*_ by the doubly reduced, structurally constrained DBA [**1**]^2–^ in THF (*n*: 1,3,4,5,6). Compound **1** is a 9,10-diphenyl DBA, forced into planarity by methylene bridges between the phenyl substituents and the DBA core. This rigidity results in enhanced stability under ambient conditions and an elevated planar-to-pyramidal reorganization energy upon boron tetracoordination, unlocking new reactivity. The dianion salts M_2_[**1**] were synthesized in excellent yields by stirring neutral **1** with alkali metals M in THF (M: Li, Na, K); comproportionation of Li_2_[**1**] with **1** generates the blue radical salt Li­[**1**], characterized by EPR spectroscopy and X-ray diffraction. While Li_2_[**1**] is inert toward C_6_FH_5_ up to 120 °C, it reacts with 1,3,5-C_6_F_3_H_3_ at 100 °C to yield a B­(sp^2^)/B­(sp^3^) adduct with a difluorophenyl ligand (Li­[**2**]). Treatment of Li_2_[**1**] with 1 eq. of C_6_F_5_H or C_6_F_6_ induces selective monohydrodefluorination, occurring in parallel with the formation of a unique B­(sp^2^)/B­(sp^3^) tetrahydrofuran-2-yl adduct (Li­[**3**]). The three isomers of C_6_F_4_H_2_ represent intermediate cases, where the competition between trifluorophenyl- and tetrahydrofuran-2-yl-adduct formation is governed by the relative positions of the F substituents and the nature of the countercation (M^+^: Li^+^, K^+^). Through experimental and quantum-chemical studies, we unveil the underlying reaction mechanisms and show that Li_2_[**1**] acts either as a B-centered nucleophile in an S_N_Ar-type conversion (low benzene fluorination) or as a reducing agent in a single-electron transfer/H atom abstraction sequence (high benzene fluorination).

## Graphical abstract

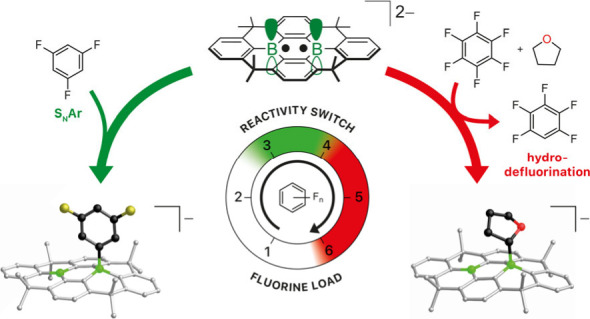

## Introduction

Despite scattered earlier examples,^1–5^ the advent of frustrated Lewis pairs (FLPs)^6–8^ marked the pivotal paradigm shift, after which element–element-bond activation was no longer considered the exclusive domain of transition-metal complexes. More recently, it has also been demonstrated that the FLP concept can be complemented by moving on to subvalent p-block compounds.^9–17^ In this regard, extended π-electron systems containing embedded trigonal-planar-coordinated boron atoms are particularly promising platforms due to their high affinity for injected electrons and ability to delocalize charge.^18–22^ The 9,10-dihydro-9,10-diboraanthracenes (DBAs) that our group has been primarily employing incorporates two closely spaced boron atoms, enabling their cooperative interaction. In the past few years, we disclosed that the doubly reduced forms [R_2_-DBA]^2–^ readily activate a number of small molecules,^23^ among them H–H,^24,25^ H–Bpin,^26^ H–CC*t*Bu,^23^ *t*BuO–O*t*Bu,^27^ and F–Ar^F^ (best performing B-bonded substituents R: H, Me; Ar^F^: fluoroaryl).^28^

Taking the nonpolar substrate H_2_ as the model system, the activation mechanism was elucidated through quantum-chemical calculations:^24,25^ It was found that the HOMO and LUMO of [R_2_-DBA]^2–^ possess the same local symmetries at the B atoms as the LUMO and HOMO of H_2_, respectively, pointing to a possible concerted oxidative addition reaction. As the polarity of the bond to be activated increases (cf. F–Ar^F^), it becomes increasingly plausible that the concerted mechanism shifts toward an S_N_Ar-type scenario involving a nucleophilic B site. Following this rationale, we achieved the synthesis of the fluorophenyl borates Li­[**A**] and Li­[**B**] by treating Li_2_[Me_2_-DBA] with 1,3,5-C_6_F_3_H_3_ and C_6_F_6_, respectively (Figure 1a).^28^ A strong indication of an S_N_Ar-type mechanism as opposed to oxidative F–C addition across both B sites is provided by the reactivity of the borafluorene dianion salt Li_2_[H-BFlu]: Despite possessing only a single B center, Li_2_[H-BFlu] exhibits the same reactivity toward F–Ar^F^ as Li_2_[Me_2_-DBA].^28,29^ Although the considerable potential of [R_2_-DBA]^2–^ for small molecule activation and even transition metal-free catalysis is rapidly emerging, an important aspect remains unresolved to-date: An efficient reaction requires unimpeded access of the substrate to the B_2_ core, which necessitates that the B atoms carry only small substituents such as R = H or Me. However, handling even neutral H_2_- and Me_2_-DBAs requires considerable experience, as they are chemically sensitive due to the lack of steric protection. The most obvious solution–replacing H/Me with Mes (mesityl)–proves ineffective, as it not only prevents the undesired attack of, e.g., H_2_O/O_2_, but also blocks any intended interactions at the B sites, even with the smallest substrate, H_2_ (Figure 1b).^25,30^ An alternative strategy is to apply the ‘concept of structural constraint’. In the present case, this leads us to compound **1** (Figure 1b),^31^ which has already demonstrated superior chemical stability in the context of emissive materials research. The stability of **1** arises from the rigidly fixed cyclic skeleton around the B atoms and renders any shielding of the B­(p_*z*_) orbitals unnecessary. Thus, in its dianionic form [**1**]^2–^, the compound may also be an attractive candidate for mediating chemical conversions.

**Figure 1:**
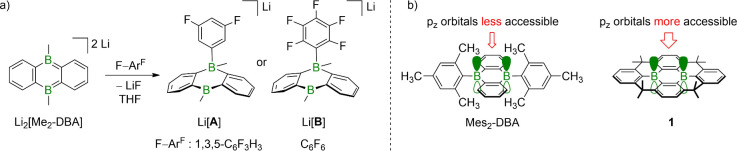
a) Activation of C–F bonds in 1,3,5-C_6_F_3_H_3_ or C_6_F_6_ by Li_2_[Me_2_-DBA] via an S_N_Ar-type mechanism. b) Kinetic stabilization of Mes_2_-DBA blocks substrate access to the B­(p_*z*_) orbitals; stabilization of **1** by structural constraint retains substrate access to the B­(p_*z*_) orbitals.

Herein, we investigate the suitability of [**1**]^2–^ for reactions with fluorobenzenes, because (i) this allows a direct comparison with the behavior of structurally unconstrained dianions [R_2_-DBA]^2–^, (ii) the available variety of isomeric partially fluorinated benzenes enables the investigation of reactivity trends in response to subtle substrate modifications, (iii) targeted derivatization of F–Ar^F^ can provide value-added products. First, we establish that both the radical [**1**]^•–^ and the closed shell species [**1**]^2–^ are accessible in essentially quantitative yields and can be fully characterized. Second, we disclose an unprecedented reactivity shift from the formation of fluorophenyl borates analogous to Li­[**A**]/Li­[**B**] to hydrodefluorination products, with the crossover point governed by the fluorine load of the substrate and the polarizability of the countercation M^+^ in M_2_[**1**] (M^+^: hard Li^+^ or soft K^+^; cf. Figure 3). Third, we prove that a nucleophilic aromatic substitution (S_N_Ar) pathway competes with a single-electron-transfer-induced (SET) radical scenario.

## Results and Discussion

### Synthesis of **1**, [**1**]^•–^, [**1**]^2–^

The structurally constrained, doubly B-doped polycyclic aromatic hydrocarbon **1** (Figure 2a) can be synthesized via an eight-step sequence, as reported by Yamaguchi et al.^31^ Each of the first seven steps achieves yields >60%, thus requiring no further optimization. However, in the final step–a 4-fold Friedel–Crafts-type cyclization on the precursor **C**–the reaction proceeds with only a 25% yield, despite extensive screening of various (Lewis) acids, which identified the employed Sc­(OTf)_3_ as the most effective catalyst among them.^32^ Given that compound **1** is central to this study, improving its yield through additional catalyst screening was imperative.^32^ It was thereby discovered that the soft Lewis acid Ag­(OTf) mediates the reaction with yields of 60–65%, regardless of the specific batch used. Notably, the best-performing catalyst, Ag­(OTf), induces the most pronounced downfield shifts of the propen-2-yl proton signals of **C** in the room-temperature ^1^H NMR spectrum, indicating a significant Ag^+^···olefin π-interaction prior to the high-temperature C–C-bond formations ().

**Figure 2:**
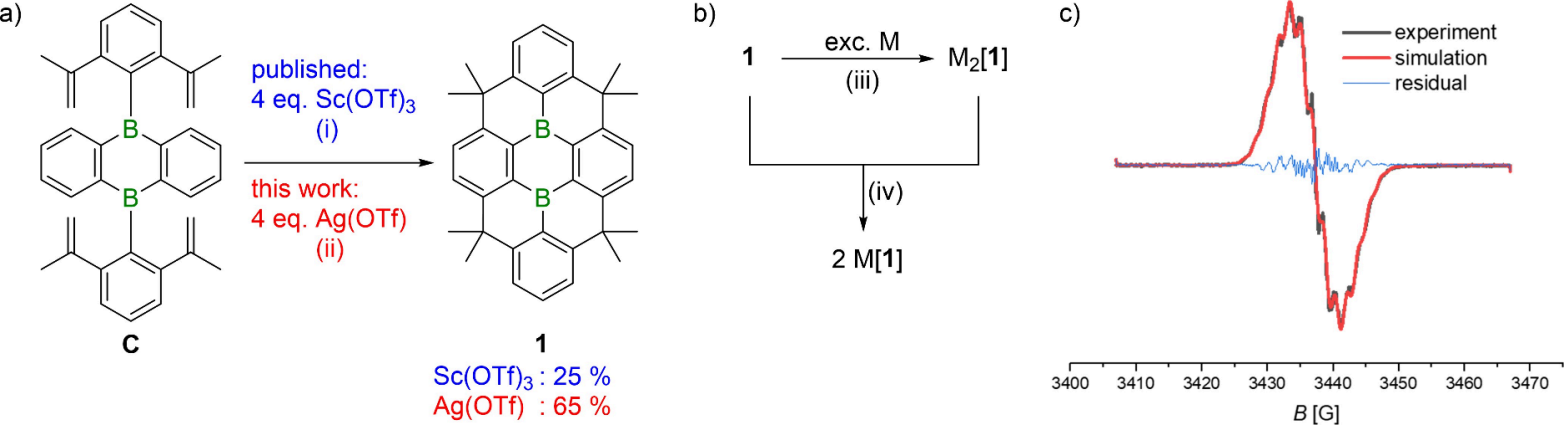
a) Replacing the Lewis acid catalyst Sc­(OTf)_3_ with Ag­(OTf) in the Friedel–Crafts-type cyclization of **C** improves the yield of **1** to 65%. b) Synthesis of the DBA-dianion salts M_2_[**1**] via two-electron reduction of **1** with excess alkali metal (M: Li, Na, K). Generation of the radical-anion salt M­[**1**] by comproportionation of **1** and M_2_[**1**] (M^+^: Li^+^, K^+^). c) Experimental (black) and simulated (red) EPR spectrum of Li­[**1**] in THF (*c* = 0.49 mmol L^–1^) at room temperature. The difference between the experimental and simulated spectra likely arises from couplings of Me protons that could not be included into the simulation. Reaction conditions: (i) 1,2-dichloroethane, 83 °C, 24 h; (ii) 1,2-dichloroethane, 100 °C, 2 d; (iii) THF, room temperature, 3 h, quantitative; (iv) THF, room temperature, instantaneous conversion, quantitative.

The cyclic voltammogram of **1** shows two reversible reduction events at *E*_1/2_ = −2.04 and −2.56 V (vs FcH/FcH^+^, [*n*Bu_4_N]­[PF_6_], THF),^31^ suggesting that chemical one- and two-electron reduction of **1** should be feasible on a preparative scale. Considering that our previous reactivity studies on [DBA]^2–^ dianions revealed decisive differences depending on the choice of the countercation,^24–26^ we performed the corresponding reduction experiments using excess Li, Na, and K metal (Figure 2b). In all cases, quantitative conversions were confirmed by ^1^H NMR spectroscopy, affording the corresponding dianion salts M_2_[**1**]. These salts, which have distinct colors in THF solution–dark green (Li^+^), brown (Na^+^), and purple (K^+^)–all appear dark green in the solid state. The radical-anion salts M­[**1**] were prepared via comproportionation reactions between equimolar amounts of **1** and M_2_[**1**] (M^+^: Li^+^, K^+^; Figure 2b). Both salts are dark blue in THF and nearly black in the solid state.

Upon exposure to ambient air, M­[**1**] and M_2_[**1**] undergo oxidation to **1** without the formation of any NMR-detectable decomposition products. This is a decisive difference compared to M­[R_2_-DBA] and M_2_[R_2_-DBA] (R: H, Me), where also the neutral DBAs are only stable under inert conditions.^33^ We note in this context the related radical species [**1** ^**C**^]^•^, which is formally derived by replacing one of the B atoms in [**1**]^•–^ with C­(sp^2^).^34^ [**1** ^**C**^]^•^ is so remarkably inert that it could even be purified by column chromatography on silica gel in air. Even at elevated temperatures, neither M­[**1**] nor M_2_[**1**] react with the THF solvent over the long-term; moreover, neither H^•^ nor H^+^ abstraction occurs in the presence of 1,4-cyclohexadiene. Yet, upon the addition of HSn­(*n*Bu)_3_, M­[**1**] affords the B­(sp^2^)···B­(sp^3^)H single hydridoborate [**1**·H]^−^, while M_2_[**1**] is converted to the corresponding symmetrical HB­(sp^3^)···B­(sp^3^)­H double hydridoborate [**1**·2H]^2–^ (see the and for more details).

The diamagnetic species M_2_[**1**] were characterized by (heteronuclear) NMR spectroscopy (M^+^: Li^+^, Na^+^, K^+^; see the for the fully assigned spectra) and single-crystal X-ray diffraction (M^+^: Li^+^, K^+^; ). In line with DFT calculations, the obtained results consistently point toward the conclusion that, despite incorporation of the boron-bonded C_6_H_3_ rings into the conjugation pathway, even in [**1**]^2–^ most of the added charge still resides on the DBA unit.

An EPR spectrum confirming the open-shell electronic structure of [**1**]^•–^ was recorded on Li­[**1**] in THF at room temperature (*c* = 0.49 mmol L^–1^; Figure 2c). The observed spectrum was simulated by assuming hyperfine coupling of the unpaired electron (*g*_iso_ = 2.0033 ± 0.0003) to two magnetically equivalent B nuclei with isotropic coupling constants of *a*(^11^B) = 1.6 ± 0.4 G and *a*(^10^B) = 0.5 ± 0.4 G. We further assumed coupling to sets of four, four, and two magnetically equivalent protons with *a*(^1^H) = 2.0 ± 0.4 G, 1.5 ± 0.4 G, and 0.7 ± 0.4 G, respectively (weak coupling of the odd electron to the CH_3_ groups was also observed, but not included in the simulation; see the Supporting Information, , for the computed spin density of [**1**]^•–^).

Single-crystal X-ray diffraction of two radical-anion salts, [Li­(12-c-4)_2_]­[**1**] and [K­(2.2.2crypt)]­[**1**]·2THF, confirmed the molecular structure of [**1**]^•–^ (, respectively). The salts form solvent-separated ion pairs in the crystal lattice.

### Reactivity of M_2_[**1**] toward Fluorobenzenes C_6_F_*n*_H_6–*n*_

With [**1**]^2–^ in hand, we next examined whether this dianion continues to function as a B-centered nucleophile or if the reorganization energy required for the planar-to-pyramidal conformational transition of the B site becomes prohibitively high due to the structurally constrained framework. We note in this context that **1** is inert toward hydrolysis yet reacts smoothly with appropriate F^–^ sources to form the corresponding mono- and difluoroborates [**1·**F]^−^ and [**1·**2F]^2–^, respectively.^31,35–37^ In the following, the reactions of M_2_[**1**] (M^+^: Li^+^, K^+^) with equimolar amounts of C_6_F_*n*_H_6–*n*_ in THF are described.^38^ Given the rather complex overall scenario (Figure 3), we will, for the sake of clarity, first provide an overview of the product distributions and discuss analytical details of the isolated products in a subsequent section. A third section is dedicated to mechanistic considerations.

**Figure 3:**
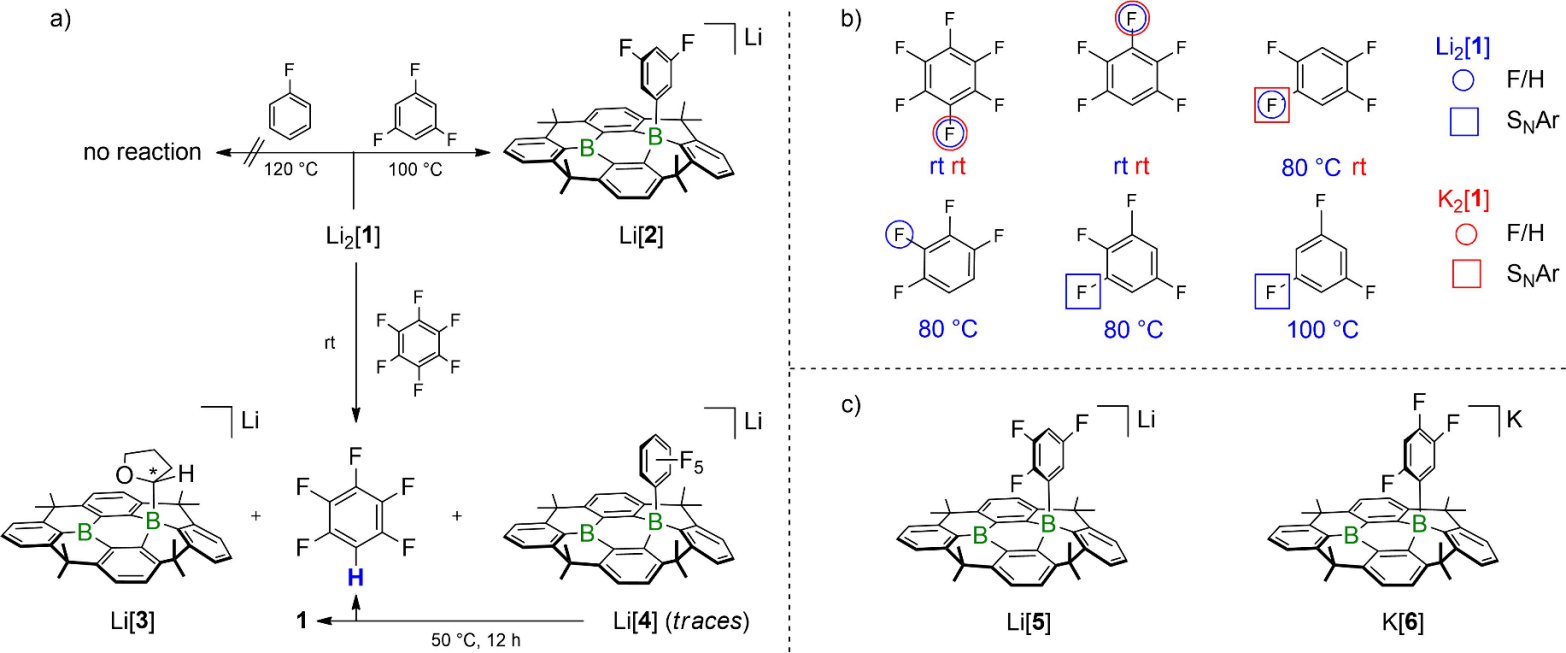
a) Reactivity of Li_2_[**1**] in THF toward C_6_FH_5_, 1,3,5-C_6_F_3_H_3_, and C_6_F_6_. b) Fluoroarenes employed and types of their conversions using Li_2_[**1**] or K_2_[**1**]. Circles: hydrodefluorinations (F/H) mediated by the Li^+^ (blue) or K^+^ salt (red). Squares: S_N_Ar-type reactions of the Li^+^ (blue) or K^+^ salt (red). c) Products of S_N_Ar-type reactions: Li_2_[**1**] and 1,2,3,5-C_6_F_4_H_2_ give Li­[**5**] (75%); K_2_[**1**] and 1,2,4,5-C_6_F_4_H_2_ give K­[**6**] (quantitative).

Similar to the structurally unconstrained Li_2_[Me_2_-DBA],^28^ Li_2_[**1**] does not react with C_6_FH_5_ up to *T* = 120 °C. With 1,3,5-C_6_F_3_H_3_, both species undergo a quantitative S_N_Ar-type reaction to furnish the difluorophenylborates Li­[**A**] (*T* = 50 °C; Figure 1a) and Li­[**2**] (*T* = 100 °C; Figure 3a).^39^ However, a striking contrast between the two species emerges in their room-temperature reactions with C_6_F_6_: While Li_2_[Me_2_-DBA] continues to undergo nucleophilic F substitution, furnishing the respective pentafluorophenylborate Li­[**B**] (Figure 1a),^28^ the reaction with Li_2_[**1**] instead leads to the formation of the tetrahydrofuran-2-yl adduct Li­[**3**] and the monohydrodefluorination product C_6_F_5_H (Figure 3a). Here, the pentafluoro-phenylborate Li­[**4**] is only formed in trace amounts; upon heating to 50 °C, Li­[**4**] is slowly converted to C_6_F_5_D and neutral **1** (in situ ^1^H NMR spectroscopy in THF-*d*_8_). Upon switching the substrate of Li_2_[**1**] from C_6_F_6_ to C_6_F_5_H, the qualitative outcome remains the same, since Li­[**3**] and 1,2,4,5-C_6_F_4_H_2_ are obtained as the by far main products at room temperature (Figure 3b). Reducing the fluorine load of the substrate further made it necessary to consider all three isomers of C_6_F_4_H_2_ and to apply different temperatures: the less thermodynamically stable^40^ 1,2,4,5- and 1,2,3,4-isomers underwent clean hydrodefluorination (Figure 3b); as indicated by a color change from green (Li_2_[**1**]) to blue, the reactions set in at room temperature and 50 °C, respectively, and required 80 °C to reach completion (judged by full decolorization and ^19^F NMR spectroscopy). In the case of the thermodynamically most favored 1,2,3,5-isomer,^40^ mainly S_N_Ar reactivity with F^1^/B substitution took place in an overall less selective conversion (75%, *T* = 80 °C; Li­[**5**], Figure 3c).

To investigate the extent to which the softer K^+^ cation influences the reaction outcome, selected experiments were repeated with K_2_[**1**]. Notably, a pronounced countercation effect was evident for the substrate 1,2,4,5-C_6_F_4_H_2_, which showed a reactivity switch from hydrodefluorination (as seen with Li_2_[**1**]) to an S_N_Ar pathway (K­[**6**]; Figure 3c). To determine whether this distinct behavior arises from differences in ion-pairing, specifically the formation of contact vs solvent-separated ion pairs by Li_2_[**1**] and K_2_[**1**] in THF solution,^41^ we conducted the experiment with Li_2_[**1**] in the presence of excess crown ether 12-c-4 at room temperature. Under these conditions, selective nucleophilic F substitution rather than hydrodefluorination occurred also with the Li^+^ salt.

At this stage, the following conclusions can be drawn: (i) Contact ion-pair formation sterically hinders substrate access to the reactive B sites of [**1**]^2–^. (ii) The suppressive effect on S_N_Ar reactivity is more pronounced than on hydrodefluorination reactivity. (iii) When both reaction pathways are sterically feasible, the S_N_Ar pathway seems to be preferred, consistent with results for the unconstrained [R_2_-DBA]^2–^ analogues. (iv) Provided that the reactant Li_2_[**1**] is not present in excess, the hydrodefluorination reaction on hexa-, penta-, and tetrafluoro-benzenes removes precisely one F atom with complete regioselectivity (Figure 3b). (v) The formation of the thermally stable (at least up to 100 °C) tetrahydrofuran-2-yl adduct Li­[**3**] as the almost exclusive byproduct of the hydrodefluorination reaction is truly remarkable in light of the known susceptibility of THF to decomposition via ring-fragmentation upon α-metalation.^42^ Other examples of authentic α-metalated THF heterocycles are rare: Mulvey et al. achieved the targeted α-zincation of THF by exploiting the unique reactivity of a bimetallic sodium dialkyl­(amido)­zincate (50% yield after 14 d).^43^ Soon after, the same group reported the ‘structurally engineered deprotonation/alumination of THF’ by means of a lithium aluminate base aggregate (35%).^44^ A second example of an α-aluminated THF molecule stems from Driess et al. and was plausibly formed through deprotonation of THF by an elusive, in situ-formed, donor–acceptor-stabilized Al­(I)–H complex.^13^ Finally, Okuda et al. synthesized a tetrahydrofuran-2-yl zirconium­(III) complex from a corresponding [Zr­(III)–SiH_2_Ph] precursor and THF (61% yield after 48 h).^45^

### Product Characterization

All NMR spectra were recorded in THF-*d*_8_ at room temperature. The ^1^H NMR spectrum of the *D*_2h_-symmetric dianion [**1**]^2–^ is characterized by one singlet resonance for the eight equivalent Me groups, alongside one singlet (4H), one doublet (4H), and one triplet (2H), all assignable to the aryl protons. The presence of the 3,5-C_6_F_2_H_3_ substituent in [**2**]^−^ lowers the symmetry of the anion, resulting in six aryl signals from the B_2_-PAH framework and four distinct Me resonances (each integrating to 6H). A ^1^H–^1^H NOESY experiment revealed that the most magnetically shielded Me group is the one in closest spatial proximity to the difluorophenyl substituent. The two ^1^H resonances of the 3,5-C_6_F_2_H_3_ ring are upfield shifted relative to the signal of 1,3,5-C_6_F_3_H_3_, with multiplicities matching those of the analogous DBA derivative Li­[**A**].^28^ The presence of 20 B_2_-PAH and 4 difluorophenyl signals in the ^13^C­{^1^H} NMR spectrum further supports the proposed molecular structure of Li­[**2**]. Its ^11^B NMR spectrum shows only a resonance for the tetracoordinate B site (δ = −16.7); as is often observed for B-PAHs, the signal corresponding to the tricoordinate B atom is broadened beyond detection.^46^ A key characteristic of the tetrahydrofuran-2-yl adduct Li­[**3**] is the further increase in the number of distinct Me resonances to eight in both the ^1^H and ^13^C­{^1^H} NMR spectra; all other H and C atoms of the B_2_-PAH scaffold are also magnetically unique. This arises from the B-bonded, stereogenic, and configurationally stable α-C atom, which imparts *C*_1_ symmetry to [**3**]^−^. Concomitantly, seven ^1^H and four ^13^C signals are detected for the tetrahydrofuran-2-yl substituent. The most striking chemical shift difference within this signal set is observed for the O-bonded α-C atoms: while δ­(^13^C) = ca. 67 for the O–*C*H_2_–C fragment closely matches that of free THF, δ­(^13^C) = 91.7 for the O–C*H–B fragment is significantly downfield shifted (the C*H proton resonates at 3.06 ppm). The B_2_-PAH unit of Li­[**5**] (Figure 3c), the major product of the S_N_Ar substitution reaction on 1,2,3,5-C_6_F_4_H_2_ (75%), shows NMR features nearly identical to those of Li­[**2**]. The ^19^F­{^1^H} NMR spectrum of Li­[**5**] displays two doublets (^3^ *J*_FF_ = 24.4 Hz, ^4^ *J*_FF_ = 17.5 Hz) and a corresponding doublet of doublets. The ^1^H and ^19^F­{^1^H} NMR spectra also provide evidence for the formation of Li­[**3**] and of hydrodefluorinated species as side products of this reaction. Additionally, X-ray crystallography revealed the generation of (at least) one isomeric substitution product cocrystallizing with Li­[**5**] (). Based on these observations, we conclude that for Li_2_[**1**], 1,2,3,5-C_6_F_4_H_2_ marks the crossover point between the S_N_Ar vs hydrodefluorination scenarios. Although the position of the B atom on the respective trifluorophenyl ring differs between compounds K­[**6**] and Li­[**5**] (Figure 3c), the spatial relationship of the F atoms remains identical. Consequently, the qualitative NMR characteristics of both compounds are so similar that a detailed discussion of the K­[**6**] spectra is not warranted (the contains the fully assigned NMR spectra and the solid-state structure of K­[**6**]). The fluorobenzenes obtained in this work are all known. In addition to in situ GC-MS, ^1^H and ^19^F NMR spectra were recorded (see the for plots of the spectra); the obtained chemical shifts and signal multiplicities align with the literature data.^47^

In addition to NMR characterization, the molecular structures of Li­[**2**], Li­[**3**], Li­[**5**], and K­[**6**] were confirmed by single-crystal X-ray diffraction (see the for full details). As representative examples, [Li­(thf)_4_]­[**2**]·0.2 THF and [Li­(thf)_3_]­[**3**]·THF are discussed in more detail (Figure 4). [Li­(thf)_4_]­[**2**]·0.2 THF crystallizes as solvent-separated ion pairs. Each anionic moiety [**2**]^−^ comprises one tetracoordinate B(1) and one tricoordinate B(2) site, with the former bearing the 3,5-C_6_F_2_H_3_ ligand. The F^–^ ion released during Li­[**2**] formation does obviously not remain coordinated to the second available Lewis-acidic center.

**Figure 4:**
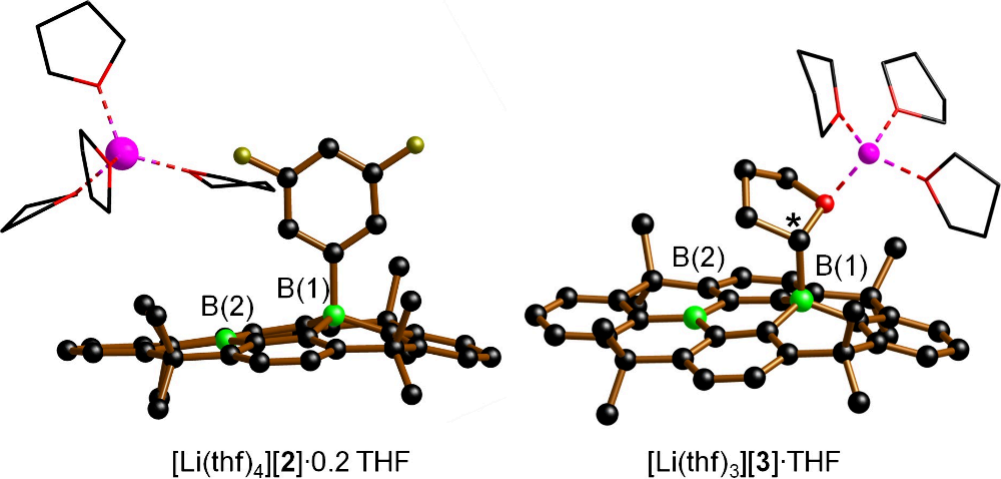
Molecular structures of [Li­(thf)_4_]­[**2**]·0.2 THF and [Li­(thf)_3_]­[**3**]·THF in the solid state. H atoms and noncoordinating solvent molecules are omitted for clarity. Li, purple; B, green; C, black; O, red; F, yellow-green.

It is revealing to compare selected structural parameters of [**2**]^−^ with those of the analogous, structurally unconstrained [Li­(12-c-4)_2_]­[**A**] (cf. Figure 1a).^28^ Reference values from [**A**]^−^ are given in curly brackets after each metrical value of [**2**]^−^: The average B(2)–C bond length of [**2**]^−^ is 1.53 Å {1.56 Å}, and the sum of bond angles about B(2) amounts to 360° {360°}, in line with a planar, sp^2^-hybridized atom. Conversely, the average length of the three endocyclic B(1)–C bonds amounts to 1.59 Å {1.64 Å}, indicating significant elongation due to rehybridization; the corresponding three endocyclic C–B(1)–C bond angles sum to 336° {332°} (ideal tetrahedron: 329°). The B(1)–C bond to the terminal difluorophenyl substituent measures 1.683(8) Å {1.665(2) Å}. Overall, B(1) in [**2**]^−^ is significantly pyramidalized, albeit to a lesser degree than the corresponding B site in [**A**]^−^. Moreover, the difference between endocyclic and exocyclic B–C bonds is more pronounced in [**2**]^−^ than in [**A**]^−^. Although the observed effects may seem to be straightforwardly attributable to the structural constraint of **1**, this is not necessarily the whole story, as its F^–^-adduct is even slightly more pyramidalized than [**A**]^−^.^28,31^ We therefore propose that the steric repulsion of the bulky Lewis base also matters, as reflected particularly in the long B(1)–C_6_F_2_H_3_ bond of [**2**]^−^. Compound [Li­(thf)_3_]­[**3**]·THF crystallizes as a contact-ion pair, with the [Li­(thf)_3_]^+^ cation bonded to the O atom of the B(1)-appended tetrahydrofuran-2-yl substituent. The bond connecting B(1) to the stereogenic C* atom of the tetrahydrofuran-2-yl residue measures 1.698(5) Å. Consistent with Bent’s rule,^48^ the C*–O/C*–CH_2_ bonds are significantly longer than the analogous bonds involving the other α-C atom, i.e., 1.477(4)/1.543(5) vs 1.434(5)/1.498(6) Å.^49^ A structurally comparable product [Li­(thf)_4_]­[**3** ^**Me**^] was obtained when the reaction between Li_2_[**1**] and C_6_F_6_ was carried out in 2,5-dimethyltetrahydrofuran (THF-Me_2_; Figure 5). As the main difference, the 2,5-dimethyltetrahydrofuran-2-yl substituent no longer coordinates to the Li^+^ cation in the solid state. Instead, the crystal lattice is composed of solvent-separated ion pairs with [Li­(thf)_4_]^+^ cations, likely as a result of the steric shielding effect imparted by the two Me groups adjacent to the O-donor atom ().

**Figure 5:**
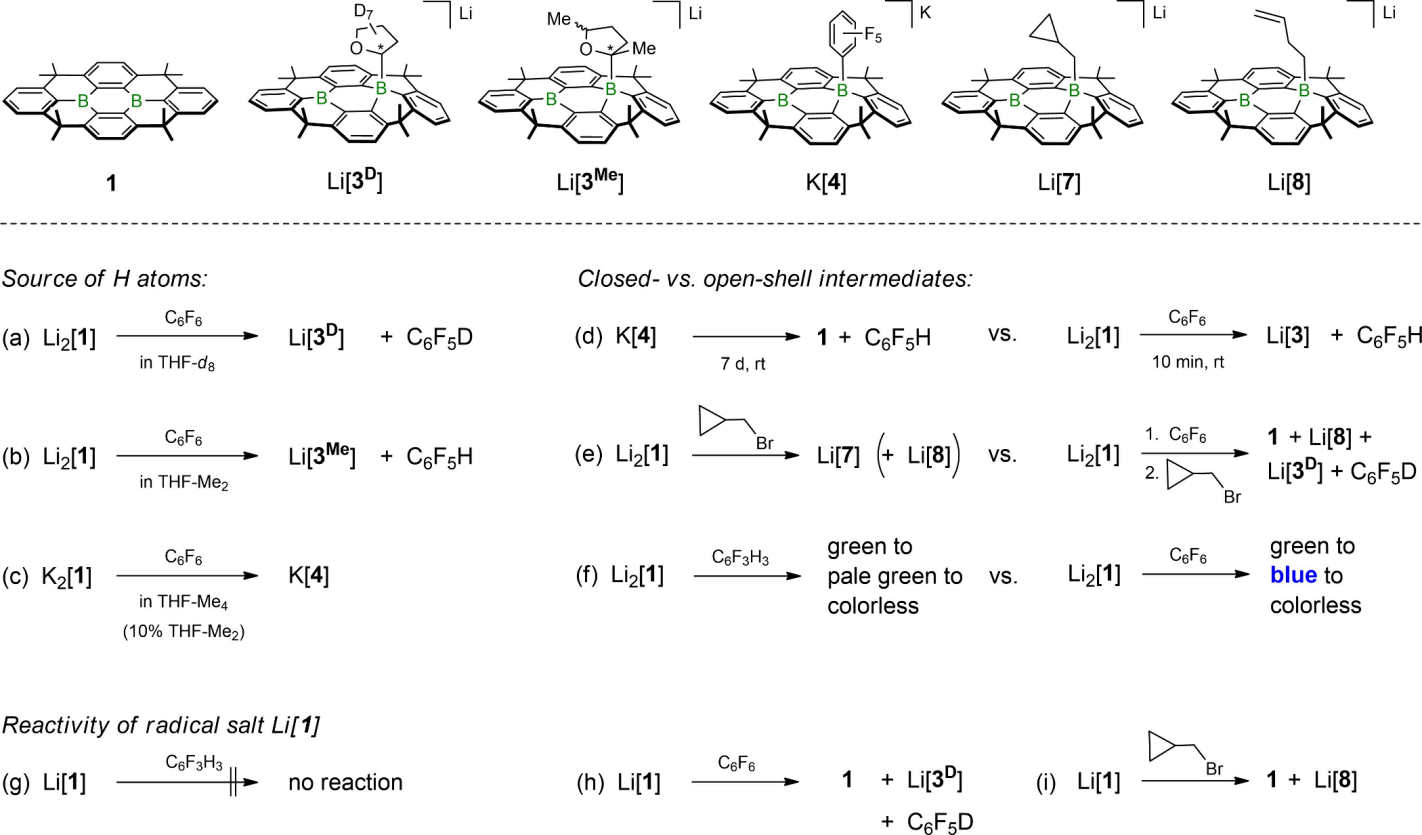
Experiments (a)–(i) provide insight into key elementary steps of the reaction mechanism underlying the reaction of Li_2_[**1**] with C_6_F_6_, leading to the formation of Li­[**3**] and C_6_F_5_H. All experiments were performed in THF or THF-*d*_8_, unless stated otherwise. (h) **1**, Li­[**3** ^**D**^], and C_6_F_5_D were formed in a 1:1:1 ratio; (i) **1** and Li­[**8**] were formed in a 1:1 ratio.

### Mechanistic Investigations

Most of the compounds newly synthesized during the mechanistic studies closely resemble those reported above, rendering a detailed discussion of their NMR and solid-state structural characteristics redundant. Given the complexity of the reaction mechanisms and the numerous details involved, we aim to keep the focus entirely on this aspect and refer to the for a compilation of all analytical data relevant to confirming the nature of the additional products (note that all products discussed in the following have been structurally characterized by single-crystal X-ray diffraction). Based on the experimental evidence gathered from the reactions of Li_2_[Me_2_-DBA] (and Li_2_[H-BFlu]) with F–Ar^F^, an S_N_Ar-type mechanism involving B-centered nucleophiles is a plausible postulate.^28^ While all available findings suggest that the transformation of Li_2_[**1**] with 1,3,5-C_6_F_3_H_3_ to afford Li­[**2**] follows a similar pathway, the reaction of Li_2_[**1**] with C_6_F_6_ takes a different route, furnishing the tetrahydrofuran-2-yl adduct Li­[**3**] and C_6_F_5_H (Figure 3a).

This process therefore warrants more detailed scrutiny (Figure 5). We began by exploring the source of the H atoms in the hydrodefluorination products: (a) Replacing THF with THF-*d*_8_ resulted in the preferential formation of C_6_F_5_D over C_6_F_5_H. While the NMR signature of the B_2_-PAH byproduct remained essentially identical to that of [**3**]^−^, we no longer detected ^1^H resonances for the tetrahydrofuran-2-yl substituent, confirming its identity as a C_4_D_7_O moiety in Li­[**3** ^**D**^]. (b) Switching from THF to THF-Me_2_ still yielded the 2,5-dimethyltetrahydrofuran-2-yl analogue Li­[**3** ^**Me**^] of Li­[**3**], along with C_6_F_5_H. (c) Removal of the remaining two α-H atoms using 2,2,5,5-tetramethyltetrahydrofuran (THF-Me_4_) required the more soluble K_2_[**1**] instead of Li_2_[**1**] and 5–10 vol % of THF-Me_2_ as solubilizing agent. Under these conditions, the C_6_F_5_ adduct K­[**4**] (Figure 5; ) was obtained as the dominant initial product, which subsequently converted to neutral **1** and C_6_F_5_H over 7 d at room temperature or within 1 d at 50 °C. These results lead to the following conclusions: (a) The THF solvent is the source of the H atom in C_6_F_5_H. (b) H atom abstraction from the solvent remains the preferred–albeit somewhat slowed down–reaction, even when the site of attack is sterically shielded in THF-Me_2_; likewise, the increased steric bulk does not prevent the formation of the B–C bond in Li­[**3** ^**Me**^] (). (c) K­[**4**] becomes isolable only if the majority of the solvent molecules lack α-H atoms (THF-Me_4_). Although compounds M­[**4**], once generated, degrade slowly via α-deprotonation of THF, yielding C_6_F_5_H and the extremely short-lived^50^ tetrahydrofuran-2-yl anion [C_4_H_7_O]^−^, the absence of [**3**]^−^ in this process suggests that hydrodefluorination within the M_2_[**1**]/C_6_F_6_/THF system does not proceed via M­[**4**] as the typical pathway. Instead, the following observations point toward a different key intermediate (Figure 5): (d) While in situ ^19^F NMR monitoring of a solution of K­[**4**] in THF confirms the gradual accumulation of C_6_F_5_H over time, the overall reaction is 3 orders of magnitude slower than the formation of C_6_F_5_H directly from the M_2_[**1**]/C_6_F_6_/THF mixture. (e) Treatment of a THF-*d*_8_ solution of Li_2_[**1**] with 1.2 eq. of the fast radical clock (bromomethyl)­cyclopropane [(C_3_H_5_)­CH_2_Br] yields the (C_3_H_5_)­CH_2_ adduct Li­[**7**] along with the ring-opened H_2_C=C­(H)­CH_2_CH_2_ adduct Li­[**8**] (85% and 15%, respectively; ). This result confirms that [**1**]^2–^ can indeed behave as a B-centered nucleophile in a predominantly closed-shell reaction.^51^ However, when 1 eq. of C_6_F_6_ was added to the mixture prior to introducing the radical clock (keeping all other conditions unchanged), exclusively the ring-opened Li­[**8**] was formed besides **1**, Li­[**3** ^**D**^], and C_6_F_5_D, providing evidence for the presence of radicals in the sample.^51^ (f) For the mixtures Li_2_[Me_2_-DBA]/1,3,5-C_6_F_3_H_3_, Li_2_[Me_2_-DBA]/C_6_F_6_, or Li_2_[**1**]/1,3,5-C_6_F_3_H_3_, the original green color of the dianion salt gradually fades as the reaction progresses. In stark contrast, the green solution of Li_2_[**1**] in THF instantaneously turns deep blue when C_6_F_6_ is added, before decolorizing within minutes (the products Li­[**3**] and C_6_F_5_H are colorless). The observed color change in (f) is particularly diagnostic, as the emerging blue tint of the reaction mixture appears visually identical to that of the radical-anion salt Li­[**1**] (see above). This prompted us to explore the possibility of a radical pathway. Given that the UV–vis spectral differences between K­[**1**] and K_2_[**1**] are more distinct than those between Li­[**1**] and Li_2_[**1**] (), we conducted subsequent experiments using the K^+^ salts, which show the same reactivity toward C_6_F_6_ as the Li^+^ salts. Absorption bands at 940 and 705 nm are particularly useful for distinguishing K­[**1**] from K_2_[**1**] in THF, as they have minimal overlap with the absorption bands of the respective counterpart (cf. the normalized spectra shown in bold black and red lines in Figure 6).^52^ The preparation of K_2_[**1**]/C_6_F_6_/THF samples under strictly inert conditions, their transfer to the cuvette, and the initiation of the UV–vis measurement typically required 40 s. Consequently, the first recorded spectrum reflects the chemical composition of the reaction mixture at room temperature after this time, revealing the presence of both unconsumed K_2_[**1**] and newly formed K­[**1**] (blue line in Figure 6). After an additional 75 s, the absorption bands of K_2_[**1**] had largely disappeared, while those of K­[**1**] remained at approximately their original intensity (brown line). After a further 9 min, however, the spectral features of K­[**1**] are also barely discernible (green line). Taken together, this supports the view that K_2_[**1**] undergoes continuous conversion to K­[**1**], which reacts in parallel at a comparable rate, resulting in a steady concentration of K­[**1**]. Once the conversion of K_2_[**1**] to K­[**1**] ceases due to the depletion of K_2_[**1**], the concentration of K­[**1**] begins to decline as well. The final product, tetrahydrofuran-2-yl adduct K­[**3**], has no absorption bands of appreciable intensity in the wavelength range of 400–1000 nm. An analogous UV–vis monitoring of the K_2_[**1**]/1,2,4,5-C_6_F_4_H_2_/THF system, which quantitatively generates the 2,4,5-C_6_F_3_H_2_ adduct K­[**6**] at room temperature (Figure 3c), revealed a gradual fading of the K_2_[**1**] bands without the intermediate emergence of the UV–vis signature of K­[**1**] (). We consider the results of the two UV–vis monitoring studies as strong evidence that the reaction of 1,2,4,5-C_6_F_4_H_2_ with K_2_[**1**] follows a closed-shell pathway, whereas an open-shell mechanism dominates in the case of C_6_F_6_.

**Figure 6:**
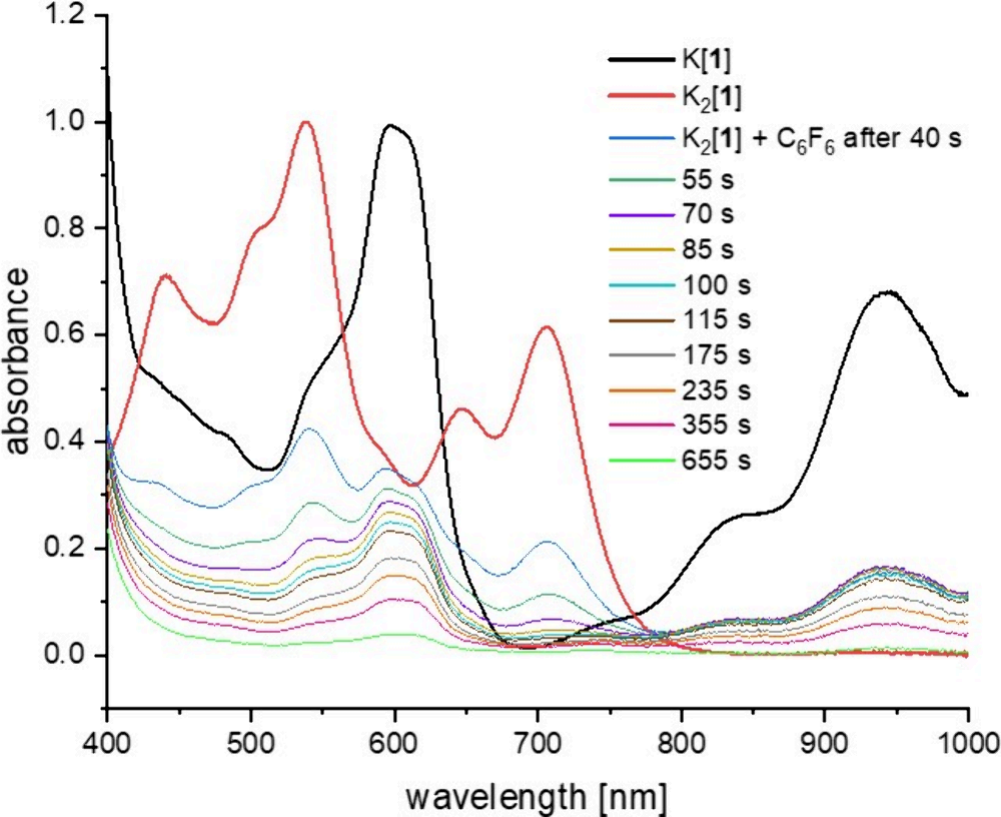
Normalized UV–vis absorption spectra of K­[**1**] (bold black line) and K_2_[**1**] (bold red line) in THF. Time-dependent UV–vis spectroscopic monitoring of an equimolar mixture of K_2_[**1**] and C_6_F_6_ in THF, recorded from 40 s after mixing (blue line).

How does the reactivity pattern differ when the radical-anion salt Li­[**1**] is used directly, rather than being generated in situ from Li_2_[**1**]/C_6_F_6_? The following series of experiments provided insights (Figure 5): (g) Li­[**1**] remains inert toward 1,3,5-C_6_F_3_H_3_ up to temperatures of 120 °C (THF-*d*_8_; the ^1^H and ^19^F NMR signals of 1,3,5-C_6_F_3_H_3_ are detectable in the otherwise NMR-silent sample and do not change over time; the dark blue color of Li­[**1**] persists). (h) With the higher fluorinated substrate C_6_F_6_, however, a room-temperature reaction occurs, furnishing **1**, Li­[**3** ^**D**^], and C_6_F_5_D in a 1:1:1 ratio. According to in situ ^19^F NMR spectroscopy, the use of 0.5 eq. C_6_F_6_ results in its quantitative conversion to C_6_F_5_D, whereas the use of 1.0 eq. C_6_F_6_ generates an equimolar mixture of C_6_F_5_D and unconsumed C_6_F_6_. (i) The radical clock (C_3_H_5_)­CH_2_Br reacts with Li­[**1**] to produce equimolar amounts of **1** and the ring-opened adduct Li­[**8**]. Experiments (g) and (h) indicate that Li­[**1**] is not sufficiently reducing to transfer an electron to 1,3,5-C_6_F_3_H_3_, yet it is a strong enough electron donor to reduce C_6_F_6_ (computed adiabatic electron affinities: –0.565 vs 0.400 eV).^53^ In turn, some Li­[**1**] molecules are converted to **1**, while others form Li­[**3** ^**D**^]. The invariant outcome of experiment (h), regardless of whether 0.5 or 1.0 eq. C_6_F_6_ is added, indicates that the SET from Li­[**1**] to C_6_F_6_ is the rate-determining step. Otherwise, the expected extent of Li­[**1**] → **1** oxidation would exceed 50% as soon as more than 0.5 eq. C_6_F_6_ is introduced. A similar reasoning holds for experiment (i): Initially, Li­[**1**] undergoes an SET with (C_3_H_5_)­CH_2_Br. Following Br^–^ elimination from [(C_3_H_5_)­CH_2_Br]^•–^, the resulting [(C_3_H_5_)­CH_2_]^•^ radical rearranges to the [H_2_C=C­(H)­CH_2_CH_2_]^•^ radical, which afterward recombines with unconsumed Li­[**1**] to furnish Li­[**8**].

Experiments (a)–(i) outlined in Figure 5 provide a consistent picture of all key steps underlying the reactions of M_2_[**1**] with fluorobenzenes. These reactions can proceed via either closed-shell S_N_Ar or open-shell SET/HAT mechanisms (HAT: hydrogen-atom transfer^54^). The most important parameter that defines the boundary between these two scenarios is the fluorine load on the benzene ring: an increasing degree *n* of benzene fluorination stabilizes the π* acceptor orbital, thereby leading to more positive adiabatic electron affinities (AEAs) of C_6_F_*n*_H_6–*n*_. According to quantum-chemical calculations and photoelectron spectroscopy, only C_6_F_6_ and C_6_F_5_H possess AEAs of appreciably positive values (>0.2 eV).^53,55–57^ This aligns with our observation that the deep blue radical intermediate [**1**]^•–^ is detected during the reactions of M_2_[**1**] with C_6_F_6_ and C_6_F_5_H, but not in those of Li_2_[**1**] with trifluorinated 1,3,5-C_6_F_3_H_3_ (computed Δ*G*_SET_ values for the corresponding SETs: –6.5, –4.3, and +9.5 kcal/mol; ). The borderline nature of tetrafluorinated 1,2,4,5-C_6_F_4_H_2_ becomes apparent from the fact that its hydrodefluorination to 1,2,4-C_6_F_3_H_3_ with Li_2_[**1**] involves [**1**]^•–^ (Δ*G*_SET_ = +4.0 kcal/mol), whereas no [**1**]^•–^ is observed during the formation of [**6**]^−^ from [Li­(12-c-4)_*n*_]_2_[**1**] or K_2_[**1**].^58^ Upon electron uptake, C_6_F_*n*_H_6–*n*_ lose their planar conformations. Specifically, C_6_F_6_ undergoes out-of-plane distortion along normal mode-like coordinates, resulting in the *C*_2v_-symmetric [C_6_F_6_]^•–^ anion: Two *para*-positioned F atoms bend upward, whereas the remaining four exhibit a slight downward bend. Consequently, the singly occupied molecular orbital (SOMO) exhibits significant π/σ*­(C–F) mixing, which explains why the injected electron can efficiently induce C–F-bond cleavage to release F^–^ and afford the [C_6_F_5_]^•^ radical (see the for an NBO analysis).^56^

In summary, considering all experimental and computational data, we propose that the reaction of [**1**]^2–^ with C_6_F_6_ is initiated by an SET, generating blue [**1**]^•–^ and [C_6_F_6_]^•–^ (blue pathway in Figure 7). While [**1**]^•–^ itself is stable in THF over the long-term, [C_6_F_6_]^•–^ rapidly releases one F^–^ ion to form the neutral [C_6_F_5_]^•^ radical,^59–61^ driven by LiF precipitation. In a subsequent HAT step, the [C_6_F_5_]^•^ radical abstracts an α-H atom from THF, yielding the hydrodefluorination product C_6_F_5_H, along with a [C_4_H_7_O]^•^ radical, which then recombines with the long-lived [**1**]^•–^ to furnish Li­[**3**].^62^ A quantum-chemical evaluation of the entire reaction sequence reveals an essentially barrierless and strongly exergonic process (). The trace amounts of side product Li­[**4**] originate either from an S_N_Ar attack of green [**1**]^2–^ on C_6_F_6_ (green pathway in Figure 7), from an attack of [C_6_F_5_]^•^ on the closed-shell arene [**1**]^2–^ (followed by one-electron oxidation of the primary product Li_2_[**4**] by C_6_F_6_), or from [C_6_F_5_]^•^/[**1**]^•–^ coupling as a minor competing pathway to the HAT step. Switching from C_6_F_6_ to 1,3,5-C_6_F_3_H_3_ shuts down the SET pathway due to the negative AEA of the new substrate. Consequently, the Li­[**4**] analogue Li­[**2**] is obtained selectively along the S_N_Ar pathway.

**Figure 7:**
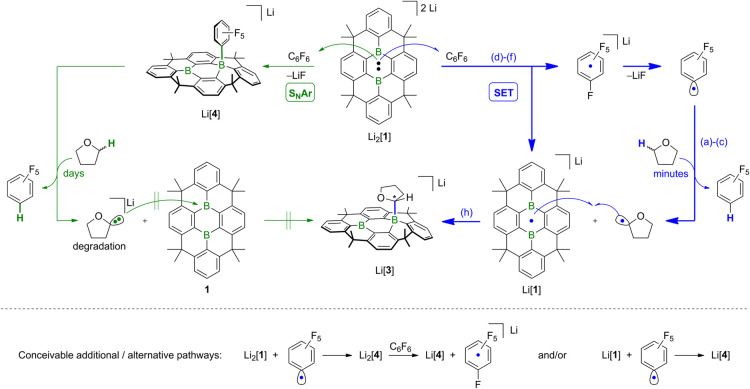
Proposed mechanistic scenario for the reaction of Li_2_[**1**] with C_6_F_6_ in THF. Blue arrows: Single-electron transfer (SET) pathway via the blue radical salt Li­[**1**] and [C_6_F_6_]^•–^ (major process). The signature product of this sequence is Li­[**3**]; labels (a)–(f), (h) refer to the experiments outlined in Figure 5 that support the proposed individual step. Green arrows: Nucleophilic aromatic substitution (S_N_Ar) pathway leading to the formation of Li­[**4**] (minor process); subsequent slow deprotonation of THF gradually converts Li­[**4**] to **1** and C_6_F_5_H. Formation of Li­[**4**] through coupling between [**1**]^2–^ or [**1**]^•–^ and [C_6_F_5_]^•^ cannot be excluded.

The two most closely related HDF/HAT reactions involving fluoroarenes reported to date have been disclosed by Weaver et al.^63^ and Zhang et al.^64^ In both cases, the reactions proceed via light-driven processes employing Ir- or pyrene-based photocatalysts, respectively. In the initial step, these photocatalysts mediate electron transfer from the sacrificial electron donor *i*Pr_2_NEt to the respective fluoroarene acceptor. The resulting fluoroarene radical anion then undergoes a similar transformation cascade as shown for Li­[C_6_F_6_] in Figure 7. However, unlike our system, the H atom donors are not the solvent but the [*i*Pr_2_NEt]^•+^ radical cations generated in the course of the photoinduced SET process. Moreover, HDF reactions driven by Li_2_[**1**] proceed within minutes and even in the dark, whereas the photocatalysis protocols require continuous irradiation over 24 h (see the for more details). Of the two literature processes, only Zhang’s–like ours–avoids the use of a transition metal. Notably, however, the two reference processes are truly catalytic, achieving turnover numbers >20.000, whereas the M_2_[**1**]/C_6_F_*n*_H_6–*n*_ reaction under current conditions proceeds in a stoichiometric manner. Nevertheless, the structural constraint that stabilizes **1** enables its easy regeneration from the side product Li­[**3**] in the presence of weak H^+^ donors, indicating the potential of our protocol for future development into a catalytic process.

## Conclusion

The search for transition metal-free homogeneous catalysts is both intellectually appealing and practically important. We previously demonstrated that doubly reduced 9,10-dihydro-9,10-diboraanthracenes ([R_2_-DBA]^2–^; R: H, Me) provide a promising compound class for activating element–element bonds. A critical limitation of R_2_-DBAs, however, is their air and moisture sensitivity, as the two B sites must remain accessible to the substrate and therefore should not be sterically protected. To overcome this issue, we introduce the structurally constrained DBA derivative **1** for element–element bond-activation, where the B atoms are embedded in a planar framework, ensuring ambient stability. Fluorobenzenes were selected as substrates due to the robust nature of their C–F bonds, which makes them challenging to address.

Despite its rigid skeleton, and similar to the unconstrained [Me_2_-DBA]^2–^, [**1**]^2–^ acts as a B-centered nucleophile toward moderately fluorinated benzenes. For example, 1,3,5-C_6_F_3_H_3_ undergoes S_N_Ar-type reactions to afford the difluorophenyl borate [**2**]^−^. In contrast to [Me_2_-DBA]^2–^, the reactivity strikingly switches when [**1**]^2–^ reacts with highly fluorinated benzenes: In THF, [**1**]^2–^ mediates hydrodefluorination of C_6_F_6_ to C_6_F_5_H, accompanied by the formation of the tetrahydrofuran-2-yl adduct [**3**]^−^ via a radical pathway. This high-yielding open-shell mechanism, unprecedented for [R_2_-DBA]^2–^ derivatives, greatly expands their reactivity spectrum. The pronounced hydrodefluorination (regio)­selectivities observed for C_6_F_6_, C_6_F_5_H, as well as 1,2,4,5- and 1,2,3,4-C_6_F_4_H_2_ suggest potential applications in the targeted synthesis of partially fluorinated arenes or in the degradation of organofluorine pollutants. The chemical stability of **1** allows its recycling by simple exposure of [**3**]^−^ to air. Additionally, [**3**]^−^ itself is notable as an extremely rare α-metalated THF derivative with potential as a tetrahydrofuran-2-yl transfer reagent in synthetic applications. Comparable adducts cannot be obtained via Lewis acid–base pairing between **1** and [C_4_H_7_O]^−^, as α-deprotonated THF undergoes instantaneous fragmentation. The α-radical, in contrast, has a sufficiently long lifetime to form [**3**]^−^ through an open-shell pathway—a vastly underexplored method for B–C-bond formation. The unique reactivity of [**1**]^2–^ showcases the power of structural constraint in tuning main-group element reactivity and provides new perspectives for catalyst design beyond transition metal chemistry.

## Supplementary Material





## Notes

All experimental and computational details (PDF)
Calculated_structures.xyz (TXT)

## ABBREVIATIONS

PAH: polycyclic aromatic hydrocarbon

## References

[1] Frey G. D., Lavallo V., Donnadieu B., Schoeller W. W., Bertrand G. (2007). Facile Splitting of Hydrogen and Ammonia by Nucleophilic
Activation at a Single Carbon Center. Science.

[2] Kenward A. L., Piers W. E. (2008). Heterolytic H_2_ Activation by Nonmetals. Angew. Chem., Int. Ed..

[3] Power P. P. (2010). Main-Group
Elements as Transition Metals. Nature.

[4] Frey G. D., Masuda J. D., Donnadieu B., Bertrand G. (2010). Activation of Si−H,
B−H, and P−H Bonds at a Single Nonmetal Center. Angew. Chem., Int. Ed..

[5] Zhao L., Huang F., Lu G., Wang Z.-X., Schleyer P. v. R. (2012). Why
the Mechanisms of Digermyne and Distannyne Reactions with H_2_ Differ So Greatly. J. Am. Chem. Soc..

[6] Welch G. C., Juan R. R. S., Masuda J. D., Stephan D. W. (2006). Reversible, Metal-Free
Hydrogen Activation. Science.

[7] Stephan D. W., Erker G. (2010). Frustrated Lewis Pairs:
Metal-free Hydrogen Activation and More. Angew.
Chem., Int. Ed..

[8] Jupp A. R., Stephan D. W. (2019). New Directions for Frustrated Lewis Pair Chemistry. Trends Chem..

[9] Weetman C., Inoue S. (2018). The Road Travelled: After Main-Group
Elements as Transition Metals. ChemCatChem..

[10] Yao S., Xiong Y., Saddington A., Driess M. (2021). Entering New Chemical
Space with Isolable Complexes of Single, Zero-Valent Silicon and Germanium
Atoms. Chem. Commun..

[11] Rodriguez R., Gau D., Kato T., Saffon-Merceron N., De Cózar A., Cossío F. P., Baceiredo A. (2011). Reversible Binding of Ethylene to
Silylene-Phosphine Complexes at Room Temperature. Angew. Chem., Int. Ed..

[12] Rodriguez R., Contie Y., Gau D., Saffon-Merceron N., Miqueu K., Sotiropoulos J., Baceiredo A., Kato T. (2013). Reversible Insertion of Unactivated
Alkenes into Silicon­(II)−Tin
Bonds. Angew. Chem., Int. Ed..

[13] Tan G., Szilvási T., Inoue S., Blom B., Driess M. (2014). An Elusive
Hydridoaluminum­(I) Complex for Facile C−H and C−O Bond
Activation of Ethers and Access to Its Isolable Hydridogallium­(I)
Analogue: Syntheses, Structures, and Theoretical Studies. J. Am. Chem. Soc..

[14] Hicks J., Vasko P., Goicoechea J. M., Aldridge S. (2018). Synthesis, Structure
and Reaction Chemistry of a Nucleophilic Aluminyl Anion. Nature.

[15] Wang Y., Karni M., Yao S., Kaushansky A., Apeloig Y., Driess M. (2019). Synthesis of an Isolable
Bis­(silylene)-Stabilized
Silylone and Its Reactivity Toward Small Gaseous Molecules. J. Am. Chem. Soc..

[16] Feng G., Chan K. L., Lin Z., Yamashita M. (2024). Alumanyl-Samarium­(II):
Synthesis, Characterization, and Reactivity Studies. J. Am. Chem. Soc..

[17] Dabringhaus P., Scherer H., Krossing I. (2024). In Situ Formation
of Reactive (Di)­Gallenes
for Bond Activation. Nat. Synth..

[18] Budy H., Gilmer J., Trageser T., Wagner M. (2020). Anionic Organoboranes:
Delicate Flowers Worth Caring For. Eur. J. Inorg.
Chem..

[19] Prey S. E., Wagner M. (2021). Threat to the Throne: Can Two Cooperating Boron Atoms
Rival Transition Metals in Chemical Bond Activation and Catalysis?. Adv. Synth. Catal..

[20] Su Y., Li Y., Ganguly R., Kinjo R. (2018). Engineering the Frontier Orbitals
of a Diazadiborinine for Facile Activation of H_2_, NH_3_, and an Isonitrile. Angew. Chem., Int.
Ed..

[21] Wang B., Kinjo R. (2019). Boron-Based Stepwise Dioxygen Activation with 1,4,2,5-Diazadiborinine. Chem. Sci..

[22] Barker J. E., Obi A. D., Dickie D. A., Gilliard R. J. (2023). Boron-Doped Pentacenes:
Isolation of Crystalline 5,12- and 5,7-Diboratapentacene Dianions. J. Am. Chem. Soc..

[23] Lorbach A., Bolte M., Lerner H.-W., Wagner M. (2010). Dilithio 9,10-Diborataanthracene:
Molecular Structure and 1,4-Addition Reactions. Organometallics.

[24] von
Grotthuss E., Diefenbach M., Bolte M., Lerner H.-W., Holthausen M. C., Wagner M. (2016). Reversible Dihydrogen Activation
by Reduced Aryl Boranes as Main-Group Ambiphiles. Angew. Chem., Int. Ed..

[25] von
Grotthuss E., Prey S. E., Bolte M., Lerner H.-W., Wagner M. (2019). Dual Role of Doubly Reduced Arylboranes as Dihydrogen-
and Hydride-Transfer Catalysts. J. Am. Chem.
Soc..

[26] Prey S. E., Herok C., Fantuzzi F., Bolte M., Lerner H.-W., Engels B., Wagner M. (2023). Multifaceted Behavior of a Doubly
Reduced Arylborane in B−H-Bond Activation and Hydroboration
Catalysis. Chem. Sci..

[27] von
Grotthuss E., Nawa F., Bolte M., Lerner H.-W., Wagner M. (2019). Chalcogen−Chalcogen-Bond Activation by an Ambiphilic,
Doubly Reduced Organoborane. Tetrahedron.

[28] Budy H., Prey S. E., Buch C. D., Bolte M., Lerner H.-W., Wagner M. (2022). Nucleophilic Borylation of Fluorobenzenes with Reduced
Arylboranes. Chem. Commun..

[29] Segawa Y., Suzuki Y., Yamashita M., Nozaki K. (2008). Chemistry of Boryllithium: Synthesis, Structure, and
Reactivity. J. Am. Chem. Soc..

[30] Taylor J. W., McSkimming A., Guzman C. F., Harman W. H. (2017). N-Heterocyclic Carbene-Stabilized
Boranthrene as a Metal-Free Platform for the Activation of Small Molecules. J. Am. Chem. Soc..

[31] Zhou Z., Wakamiya A., Kushida T., Yamaguchi S. (2012). Planarized
Triarylboranes: Stabilization by Structural Constraint and Their Plane-to-Bowl
Conversion. J. Am. Chem. Soc..

[32] Catalysts screened in ref 31: “various proton acids”, BF_3_·OEt_2_, AlCl_3_, FeCl_3_, TiCl_4_, Sc(OTf)_3_, Sn(OTf)_2_, Bi(OTf)_3_. Catalysts screened in this work: Al(OTf)_3_, In(OTf)_3_, Bi(OTf)_3_, Gd(OTf)_3_, Cu(OTf)·C_6_H_6_, Ag(OTf), Au(NTf_2_)PPh_3_, Hg(OTf)_2_.

[33] von
Grotthuss E., John A., Kaese T., Wagner M. (2018). Doping Polycyclic
Aromatics with Boron for Superior Performance in Materials Science
and Catalysis. Asian J. Org. Chem..

[34] Kushida T., Shirai S., Ando N., Okamoto T., Ishii H., Matsui H., Yamagishi M., Uemura T., Tsurumi J., Watanabe S., Takeya J., Yamaguchi S. (2017). Boron-Stabilized
Planar Neutral π-Radicals with Well-Balanced Ambipolar Charge-Transport
Properties. J. Am. Chem. Soc..

[35] Hirai M., Tanaka N., Sakai M., Yamaguchi S. (2019). Structurally
Constrained Boron-, Nitrogen-, Silicon-, and Phosphorus-Centered Polycyclic
π-Conjugated Systems. Chem. Rev..

[36] Matsuo K., Saito S., Yamaguchi S. (2014). Photodissociation of B−N Lewis
Adducts: A Partially Fused Trinaphthylborane with Dual Fluorescence. J. Am. Chem. Soc..

[37] Ando N., Yamada T., Narita H., Oehlmann N. N., Wagner M., Yamaguchi S. (2021). Boron-Doped
Polycyclic π-Electron Systems with
an Antiaromatic Borole Substructure That Forms Photoresponsive B−P
Lewis Adducts. J. Am. Chem. Soc..

[38] For practical reasons, primarily to avoid an undesired excess of M_2_[**1**], the fluorobenzenes were added in slight excess, ranging from 1.1–1.5 equiv.

[39] All other regioisomers of C_6_F_3_H_3_ also undergo S_N_Ar reactions; however, these proceed without regioselectivity.

[40] Fuhrer T. J., Houck M., Iacono S. T. (2021). Fluoromaticity: The Molecular Orbital
Contributions of Fluorine Substituents to the π-Systems of Aromatic
Rings. ACS Omega.

[41] Based on the same arguments previously used to assess which M_2_[H_2_-DBA] salts (M^+^ = Li^+^, Na^+^, K^+^) exist in THF solution as contact or solvent-separated ion pairs,^25^ we arrive at the following conclusion for M_2_[**1**]: Li_2_[**1**] likely exists in THF as a contact ion pair, whereas Na_2_[**1**] and K_2_[**1**] form solvent-separated ion pairs.

[42] Maercker A. (1987). Ether Cleavage
with Organo-Alkali-Metal Compounds and Alkali Metals. Angew. Chem., Int. Ed. Engl..

[43] Kennedy A. R., Klett J., Mulvey R. E., Wright D. S. (2009). Synergic Sedation
of Sensitive Anions: Alkali-Mediated Zincation of Cyclic Ethers and
Ethene. Science.

[44] Crosbie E., García-Álvarez P., Kennedy A. R., Klett J., Mulvey R. E., Robertson S. D. (2010). Structurally
Engineered Deprotonation/Alumination of THF and THTP with Retention
of Their Cycloanionic Structures. Angew. Chem.,
Int. Ed..

[45] Kulinna H., Spaniol T. P., Maron L., Okuda J. (2013). Selective α-Metalation
of THF by a Cationic Zirconium Complex Supported by an (NNNN)-Type
Macrocyclic Ligand. Chem. - A Eur. J..

[46] Nöth, H. ; Wrackmeyer, B. Nuclear Magnetic Resonance Spectroscopy of Boron Compounds. In NMR—Basic Principles and Progress; Diehl, P. , Fluck, E. , Kosfeld, R. , Eds.; Springer: Berlin, Heidelberg, 1978.

[47] Rosenau C. P., Jelier B. J., Gossert A. D., Togni A. (2018). Exposing the Origins
of Irreproducibility in Fluorine NMR Spectroscopy. Angew. Chem., Int. Ed..

[48] Foster J. P., Weinhold F. (1980). Natural Hybrid Orbitals. J. Am.
Chem. Soc..

[49] Luger P., Buschmann J. (1983). Twist Conformation of Tetrahydrofuran in the Crystal. Angew. Chem., Int. Ed. Engl..

[50] Bates R. B., Kroposki L. M., Potter D. E. (1972). Cycloreversions
of Anions from Tetrahydrofurans.
Convenient Synthesis of Lithium Enolates of Aldehydes. J. Org. Chem..

[51] Nonhebel D. C. (1993). The Chemistry
of Cyclopropylmethyl and Related Radicals. Chem.
Soc. Rev..

[52] The most bathochromic absorption band of the B,C congener [**1** ^ **C** ^]^•^ in the UV–vis spectrum appears at 592 nm (toluene; ref. 34) and thus at markedly higher energies compared to the corresponding absorption band of K[**1**] (940 nm; THF).

[53] Davis J. U., Jarrold C. C., Sommerfeld T. (2023). Charge Distribution in Oxygen•Fluorobenzene
Complex Anions [O_2_•C_6_H_6‑*n*
_F_
*n*
_]^−^ (*n* = 0−6). Chem. Phys..

[54] Mayer J. M. (2011). Understanding
Hydrogen Atom Transfer: From Bond Strengths to Marcus Theory. Acc. Chem. Res..

[55] Miller T. M., Van Doren J. M., Viggiano A. A. (2004). Electron Attachment and Detachment:
C_6_F_6_. Int. J. Mass Spectrom..

[56] Freeman P. K., Srinivasa R. (1987). Photochemistry
of Polyhaloarenes. 6. Fragmentation of Polyfluoroarene Radical Anions. J. Org. Chem..

[57] McGee C. J., McGinnis K. R., Jarrold C. C. (2023). Anion Photoelectron
Imaging Spectroscopy
of C_6_HF_5_
^–^, C_6_F_6_
^–^, and the Absence of C_6_H_2_F_4_
^–^. J.
Phys. Chem. A.

[58] The Gibbs free energy changes (Δ*G* _SET_) were computed at the SMD(THF)/ωB97X-D/def2-QZVP level of theory, using geometries optimized at the SMD(THF)/ωB97X-D/def2-SVP level.

[59] Laev S. S., Shteingarts V. D. (1998). Reductive
Defluorination of Perfluoroarenes by Zinc in Aqueous Ammonia. J. Fluorine Chem..

[60] McGinnis K. R., McGee C. J., Sommerfeld T., Jarrold C. C. (2024). Anion Photoelectron
Imaging Spectroscopy of C_6_F_5_X^–^ (X = F, Cl, Br, I). J. Phys. Chem. A.

[61] McGinnis K. R., McGee C. J., Jarrold C. C. (2024). Isomer-Dependent Electron Affinities
of Fluorophenyl Radicals, •C_6_H_5‑x_F_x_ (2 ≤ *x* ≤ 4). J. Am. Chem. Soc..

[62] C–B-bond formation between [C_4_H_7_O]^•^ and yet unconsumed [**1**]^2–^ is also conceivable. Since the resulting [**3**]^•2–^ is likely a strong reducing agent, it should rapidly undergo SET with remaining C_6_F_6_ to afford [**3**]^−^ and [C_6_F_6_]^•–^. While this possibility cannot be excluded, radical–radical recombination has been experimentally confirmed.

[63] Senaweera S. M., Singh A., Weaver J. D. (2014). Photocatalytic Hydrodefluorination:
Facile Access to Partially Fluorinated Aromatics. J. Am. Chem. Soc..

[64] Lu J., Khetrapal N. S., Johnson J. A., Zeng X. C., Zhang J. (2016). “π-Hole-π”
Interaction Promoted Photocatalytic Hydrodefluorination via Inner-Sphere
Electron Transfer. J. Am. Chem. Soc..

