# Supplementary material for: Planarity Is Not Plain: Closed- vs Open-Shell Reactivity of a Structurally Constrained, Doubly Reduced Arylborane toward Fluorobenzenes
Source: J Am Chem Soc. 2025 May 29;147(23):20071–81. doi: 10.1021/jacs.5c05588 (PMC12164271; doi:10.1021/jacs.5c05588)
Supplement: Supplementary file 1 [file ja5c05588_si_001.pdf]

Supporting Information

Planarity is not Plain: Closed- vs. Open-Shell Reactivity of a Structurally Constrained, Doubly Reduced Arylborane toward Fluorobenzenes

Christoph D. Buch,[a] Alexander Virovets,[a] Eugenia Peresypkina,[a] Burkhard Endeward,[a] Hans-Wolfram Lerner,[a] Felipe Fantuzzi,[b] Shigehiro Yamaguchi[c] and Matthias Wagner*[a]

[a]*Institut für Anorganische und Analytische Chemie, Goethe-Universität Frankfurt, Max-von-Laue Straße 7, D-60438 Frankfurt (Main), Germany*
[b] *School of Chemistry and Forensic Science, University of Kent, Park Wood Rd, Canterbury CT2 7NH, UK*
[c] *Department of Chemistry, Graduate School of Science, and Integrated Research Consortium on Chemical Sciences (IRCCS), Nagoya University, Furo, Chikusa, Nagoya 464-8602, Japan*

*To whom correspondence should be addressed.
Email: matthias.wagner@chemie.uni-frankfurt.de

Table of contents

1. Experimental details and characterization data ... 5
1.1 General considerations ... 5
1.2 Optimization of the synthesis of **1** ... 6
1.3 Chemical reduction of **1** ... 8
1.3.1 Synthesis of Li_2[**1**] ... 8
1.3.2 Synthesis of Na_2[**1**] ... 9
1.3.3 Synthesis of K_2[**1**] ... 10
1.3.4 Reduction of **1** in different THF derivatives and ethers ... 11
1.3.5 Synthesis of Li[**1**] ... 12
1.3.6 Insights into the electronic structures of **1**, [**1**]$^{\bullet-}$, and [**1**]$^{2-}$ ... 13
1.3.6.1 Bond length changes upon reduction of **1** ... 13
1.3.6.2 $^{13}C\{^1H\}$ NMR shift changes upon reduction of Mes_2-DBA and **1** ... 14
1.3.6.3 Conclusion ... 16
1.4 Reactivity of Li[**1**] and Li_2[**1**] toward H-atom donors ... 17
1.4.1 Reaction of Li[**1**] with 1,4-cyclohexadiene ... 17
1.4.2 Reaction of Li[**1**] with *n*Bu_3SnH ... 18
1.4.3 Reaction of Li_2[**1**] with *n*Bu_3SnH ... 19
1.5 Reactions of Li_2[**1**] with different fluorobenzene derivatives ... 20
1.5.1 Reaction of Li_2[**1**] with C_6F_6 ... 20
1.5.2 General protocol for the reactions of Li_2[**1**] with fluorobenzenes ... 26
1.5.3 Reaction of Li_2[**1**] with C_6F_6 in THF-Me_2 ... 34
1.6 Reaction of Na_2[**1**] with C_6F_6 ... 36
1.7 Reactions of K_2[**1**] with different fluorobenzene derivatives ... 37
1.7.1 Reaction of K_2[**1**] with C_6F_6 ... 37
1.7.2 Reaction of K_2[**1**] with 1,2,4,5-$C_6F_4H_2$ ... 38
1.7.3 Synthesis of K[**4**] in 2,2,5,5-Me_4-THF ... 40
1.8 Mechanistic investigations ... 42
1.8.1 Stability of K[**4**] ... 42
1.8.2 Reaction of Li[**1**] with C_6F_6 ... 43
1.8.3 Reaction of Li[**1**] with 1,3,5-$C_6F_3H_3$ ... 44
1.8.4 Reaction of Li_2[**1**] with 1,2,4,5-$C_6F_4H_2$ in the presence of 12-c-4 ... 45
1.8.5 Reaction of Li_2[**1**] with (bromomethyl)cyclopropane ... 46
1.8.6 Reaction of Li_2[**1**] with 4-bromo-1-butene ... 48
1.8.7 Reaction of Li[**1**] with (bromomethyl)cyclopropane ... 49
1.8.8 Reaction of Li_2[**1**] with C_6F_6 and (bromomethyl)cyclopropane ... 50
1.9 Reactivity of Li[**3**] ... 51

1.9.1 Reaction of Li[**3**] with HCl 51
1.9.2 Reaction of Li[**3**^{D}] with ambient air 52
2 NMR spectra 53
3 General procedure for UV-vis experiments 104
3.1 Procedure for recording UV-vis spectra of the compounds Li_2[**1**], Li[**1**], K_2[**1**], and K[**1**] 105
3.2 Procedure for reaction monitoring of K_2[**1**] and C_6F_6 by UV-vis spectroscopy 105
3.3 Procedure for reaction monitoring of K_2[**1**] and 1,2,4,5-$C_6F_4H_2$ by UV-vis spectroscopy 105
4 Plots of UV-vis spectra 106
5 EPR spectrum of Li[**1**] 109
6 X-ray crystal structure determinations 110
6.1 Single-crystal X-ray structure analysis of **1**•THF 118
6.2 Single-crystal X-ray structure analysis of $[Li(thf)_2]_2$[**1**] 119
6.3 Single-crystal X-ray structure analysis of $[K(thf)_{2.5}]_2$[**1**] 119
6.4 Single-crystal X-ray structure analysis of $[Li(12\text{-}c\text{-}4)_2]$[**1**] 122
6.5 Single-crystal X-ray structure analysis of $[Li(thf)_3]$[**3**]•THF 123
6.6 Single-crystal X-ray structure analysis of $[Li(thf)_4]$[**3**^{Me}] 124
6.7 Single-crystal X-ray structure analysis of $[Li(thf)_4]$[**5**]•0.4 THF 125
6.8 Single-crystal X-ray structure analysis of $[Li(thf)_4]$[**2**]•0.2 THF 126
6.9 Single-crystal X-ray structure analysis of $[K(thf)_4]$[**6**] 127
6.10 Single-crystal X-ray structure analysis of $[K(thf)_4]$[**4**] 128
6.11 Single-crystal X-ray structure analysis of $[Li(12\text{-}c\text{-}4)_2]$[**7**]$_{0.79}$[**8**]$_{0.21}$•2THF 129
6.12 Single-crystal X-ray structure analysis of $[Li(thf)_4]$[**8**] 130
6.13 Single-crystal X-ray structure analysis of [K(2.2.2crypt)][**1**]•2THF 131
7 Computational Details 133
7.1 Frontier orbitals of **1**, [**1**]$^{\bullet-}$, [**1**]$^{2-}$, fluorobenzenes and fluorobenzene radical anions 134
7.1.1 Spin density of [**1**]$^{\bullet-}$ 139
7.1.2 Distorted structures of fluorobenzene radical anions 140
7.2 Mechanism of the hydrodefluorination reaction 142
7.2.1 Gibbs free energy values (ΔG_{SET}) for the SET from [**1**]$^{2-}$ to $C_6F_nH_{6-n}$ 142
7.2.4 Natural bond orbital calculations of $[C_6F_6]^{\bullet-}$ 143
7.2.4.1 Symmetrically distorted structure of $[C_6F_6]^{\bullet-}$: NBO calculations 143
7.2.4.2 Asymmetrically distorted structure of $[C_6F_6]^{\bullet-}$: NBO calculations 145
7.2.5 LiF-elimination 148
7.2.6 H·-abstraction 150
7.2.7 Recombination of the THF radical with [**1**]$^{\bullet-}$ or [**1**]$^{2-}$ 152
7.3 Summarized mechanism 153

7.4 Computed structures and corrected free energies 154
8 References 158

1. Experimental details and characterization data

1.1 General considerations

Unless noted otherwise, all reactions, manipulations, and analyses were carried out in an Ar-filled glovebox or by applying standard Schlenk techniques under an argon atmosphere. 1,4-Dioxane, tetrahydropyran (THP), 2,2,5,5-Me_4-THF (synthesized according to a literature procedure),[S1] 2,5-Me_2-THF, tetrahydrofuran (THF), dimethoxyethane (DME), diethyl ether (Et_2O), methyl *tert*-butyl ether (MTBE), hexamethyldisiloxane ($Me_3SiOSiMe_3$), and toluene were dried over Na/benzophenone; *n*-hexane was dried over Na without benzophenone; THF-d_8 was dried over Na-K alloy without benzophenone. *Ortho*-dichlorobenzene (*o*DCB), 1,2-dichloroethane (DCE), and $CDCl_3$ were dried over CaH_2. Prior to use, the solvents were distilled from the drying agent, degassed by applying three freeze-pump-thaw cycles, and stored over activated molecular sieves (3 Å). All fluorobenzenes employed in this study are commercially available (Sigma-Aldrich, TCI); they were dried over CaH_2, degassed after distillation using three freeze-pump-thaw cycles, and stored over activated molecular sieves (3 Å). (Bromomethyl)cyclopropane, 4-bromo-1-butene, 12-crown-4 and 1,4-cyclohexadiene were degassed by applying three freeze-pump-thaw cycles and stored over activated molecular sieves (3 Å) in a glovebox. All other commercially available reagents were used as received. Compound **C** was synthesized according to a literature procedure.[S2]

NMR spectra were recorded at 298 K using the following *Bruker* spectrometers: *Avance-300™, Avance-400 ™*, *Avance-500 ™*, or *Avance DRX 600 ™*. Chemical shifts are referenced to (residual) solvent signals ($^{1}H/^{13}C\{^{1}H\}$: THF-d_8: δ = 3.58/67.21 ppm; $CDCl_3$: δ = 7.26/77.16 ppm)[S3] or external $BF_3{\cdot}OEt_2$ (^{11}B; $^{11}B\{^{1}H\}$), $CFCl_3$ (^{19}F; $^{19}F\{^{1}H\}$), $SnMe_4$ in C_6D_6 ($^{119}Sn\{^{1}H\}$) and LiCl in D_2O (^{7}Li). Abbreviations: s = singlet, d = doublet, t = triplet, q = quartet, quint = quintet, sext = sextet, m = multiplet, v = virtual, br = broad, $h_{1/2}$ = full width at half maximum, *i* = ipso, *o* = ortho, *m* = meta, *p* = para. If not stated otherwise, resonance assignments were aided by ^{1}H-^{13}C-HSQC, ^{1}H-^{13}C-HMBC, ^{1}H-^{1}H-COSY, and ^{1}H-^{1}H-NOESY NMR experiments. The numbering schemes follow the nomenclature given in the reaction schemes.

UV-vis absorption spectra were recorded at room temperature using a Varian *Cary 60 Scan* UV-vis spectrophotometer.

1.2 Optimization of the synthesis of **1**

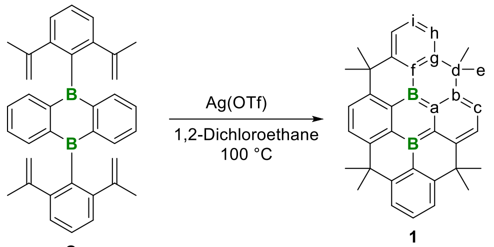

1 was first synthesized by Yamaguchi et al., employing $Sc(OTf)_3$ as the Lewis acid catalyst in the final step (**C** → **1**).[S2] Since **1** serves as the key compound for all reactions in this work, improving the reported yield of 25 % for this step became an objective. Consequently, a range of Lewis acids was screened (Table S1). The procedure using Ag(OTf) as the Lewis acid is described below, as it provided the highest yields. All other reactions were conducted following the same protocol used for Ag(OTf).

Already at room temperature, Ag(OTf) interacts significantly more strongly than $Sc(OTf)_3$ with the double bonds of **C**, as evidenced by the downfield-shifted allylic resonances in the ^{1}H NMR spectrum of the **C**/Ag(OTf) sample ($CDCl_3$; Figure S2). This phenomenon was most pronounced for Ag(OTf) (though observed to a lesser extent also for $Cu(OTf)\cdot C_6H_6$), indicating that Ag(OTf) has the highest activating effect among the Lewis acids tested.

General procedure:

In a glovebox, a thick-walled glass ampoule was charged with **C** (100 mg, 205 µmol, 1 eq.), Ag(OTf) (210 mg, 820 µmol, 4 eq.), and dry, degassed 1,2-dichloroethane (20 mL). The ampoule was flame-sealed under vacuum and heated to 100 °C in an oven for 2 d. Under ambient conditions, the reaction mixture was filtered through a silica plug to remove excess Ag(OTf). The crude product was purified by flash chromatography (C_6H_{12}:CH_2Cl_2 = 8:1). The obtained slightly yellow solid was further purified by washing with cold H_3CCN (3 x 5 mL) to obtain **1** as a colorless solid (65 mg, 133 µmol, 65 %; the yield reproducibly falls in the range 60 – 65%).

^{1}H-, ^{11}B-, and ^{13}C{^{1}H}-NMR spectra (in $CDCl_3$) of **1** have been previously published.[S2] In the following, nearly all reactions were carried out in THF-d_8 (or THF) as the solvent. Therefore, our own spectra (in THF-d_8) along with complete signal assignments are provided below (Figures S3 – S4).

Note: Compound **1** is poorly soluble in THF, resulting in relatively low signal intensity in the NMR spectra.

*NMR shifts of compound **1***

^{1}H NMR (500.2 MHz, THF-d_8): δ = 8.03 (s, 4H; H_c), 7.76 – 7.70 (m, 6H; H_h and H_i), 1.80 (s, 24H; H_e).

^{11}B NMR (160.5 MHz, THF-d_8): δ = n.o.

$^{13}C\{^1H\}$ NMR (125.8 MHz, THF-d_8): δ = 157.6 (C_g), 155.0 (C_b), 138.6 (br; C_a)*, 133.6 (C_i), 132.0 (C_c), 124.9 (C_h), 43.0 (C_d), 33.5 (C_e); n.o. C_f. *) The position of the signal was confirmed by cross-peaks in the $^1H/^{13}C$-HMBC NMR spectrum.

Table S1: Screened Lewis acids and reaction conditions employed to optimize the synthesis of **1**; DCE: 1,2-dichloroethane, *o*DCB: *ortho*-dichlorobenzene.

Lewis acid	Equivalents	Solvent	Temperature	Isolated yield
$Sc(OTf)_3$	4	DCE	100 °C	25 % [S2]
**Ag(OTf)**	**4**	**DCE**	**100 °C**	**65 %**
$Al(OTf)_3$	4	DCE	120 °C	25 %
$Au(NTf_2)PPh_3$	0.3	DCE	100 °C	45 %
$Bi(OTf)_3$	4	DCE	120 °C	30 %
$CuOTf \cdot C_6H_6$	4	DCE	100 °C	Decomposition
$Gd(OTf)_3$	4	DCE	180 °C	No reaction
$Hg(OTf)_2$	4	*o*DCB	120 °C	30 %
$In(OTf)_3$	4	DCE	120 °C	25 %
$Sn(OTf)_2$	4	DCE or *o*DCB	120 °C	Decomposition

1.3 Chemical reduction of **1**

1.3.1 Synthesis of Li_2[**1**]

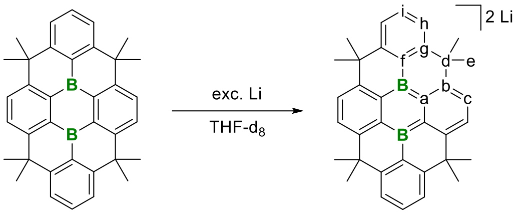

1 Li2[**1**]

In a glovebox, **1** (10.0 mg, 20.5 µmol, 1 eq.) was placed in a screw-cap vial, followed by the addition of THF-d_8 (0.4 mL). Li granules (10 mg, exc.) were added to the suspension, and the reaction mixture was stirred for a minimum of 3 h. The resulting dark green solution was transferred to an NMR tube, which was then flame-sealed. NMR spectroscopy confirmed the quantitative formation of Li_2[**1**] (Figures S5 – S8).

X-ray quality single crystals of [$Li_2(thf)_4$][**1**] were obtained by slow vapor diffusion of *n*-hexane (1 mL) into a solution of Li_2[**1**] in THF (39 mmol L^{-1}).

Note: When solutions of Li_2[**1**] in THF-d_8 are exposed to ambient air, the subsequently recorded NMR spectra show exclusively the resonances of **1** and no signals assignable to any decomposition products.

*NMR shifts of compound Li_2[**1**]*

^{1}H NMR (500.2 MHz, THF-d_8): δ = 7.21 (d, $^3J_{HH}$ = 7.7 Hz, 4H; H_h), 6.96 (s, 4H; H_c), 6.90 (t, $^3J_{HH}$ = 7.7 Hz, 2H; H_i), 1.68 (s, 24H; H_e).

^{7}Li NMR (194.4 MHz, THF-d8) δ = − 6.9 (br s).

^{11}B NMR (160.5 MHz, THF-d_8): δ = 17.8 (br, $h_{1/2}$ = 500 Hz).

^{13}C{^{1}H} NMR (125.8 MHz, THF-d_8): δ = 151.1 (C_b), 149.3 (C_g), 140.9* (br; C_f), 129.3* (br; C_a), 124.4 (C_i), 122.3 (C_h), 116.6 (C_c), 42.8 (C_d), 34.8 (C_e).
*) The positions of these signals were confirmed by cross-peaks in the ^{1}H/^{13}C-HMBC NMR spectrum.

1.3.2 Synthesis of Na_2[**1**]

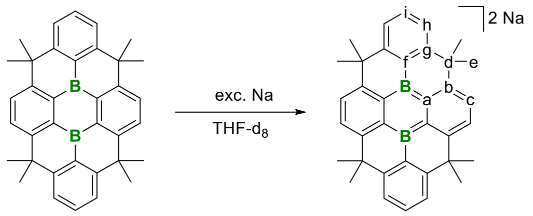

In a glovebox, **1** (10.0 mg, 20.5 µmol, 1 eq) was placed in a screw-cap vial, followed by the addition of THF-d_8 (0.4 mL). Na metal (15 mg, exc.) was added to the suspension, and the reaction mixture was stirred for a minimum of 3 h. The resulting dark brown solution was transferred to an NMR tube, which was then flame-sealed. NMR spectroscopy confirmed the quantitative formation of Na_2[**1**] (Figures S9 – S11).

Note: When solutions of Na_2[**1**] in THF-d_8 are exposed to ambient air, the subsequently recorded NMR spectra show exclusively the resonances of **1** and no signals assignable to any decomposition products.

*NMR shifts of compound Na_2[**1**]*

^{1}H NMR (500.2 MHz, THF-d_8): δ = 7.07 (d, $^3J_{HH}$ = 7.6 Hz, 4H; H_h), 6.80 (s, 4H; H_c) 6.69 (t, $^3J_{HH}$ = 7.6 Hz, 2H; H_i), 1.62 (s, 24H; H_e).

^{11}B NMR (160.5 MHz, THF-d_8): δ = 18.2 (br, $h_{1/2}$ = 500 Hz).

^{13}C{^{1}H} NMR (125.8 MHz, THF-d_8): δ = 150.9 (C_b), 149.0 (C_g), 142.8* (br; C_f), 132.4* (br; C_a), 122.8 (C_h), 122.6 (C_i), 115.2 (C_c), 42.4 (C_d), 35.9 (C_e).
*) The positions of these signals were confirmed by cross-peaks in the ^{1}H/^{13}C-HMBC NMR spectrum.

1.3.3 Synthesis of K_2[**1**]

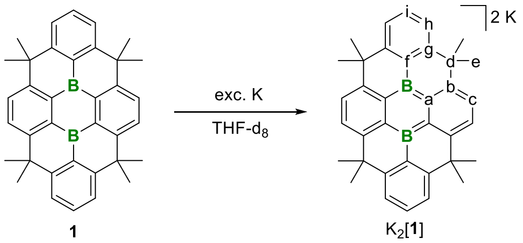

1 K2[**1**]

In a glovebox, **1** (10.0 mg, 20.5 µmol, 1 eq.) was placed in a screw-cap vial, followed by the addition of THF-d_8 (0.4 mL). K metal (15 mg, exc.) was added to the suspension, and the reaction mixture was stirred for a minimum of 3 h. The resulting dark purple solution was transferred to an NMR tube, which was then flame-sealed. NMR spectroscopy confirmed the quantitative formation of K_2[**1**] (Figures S12 – S14).

X-ray quality single crystals of [$K_2(thf)_5$][**1**] were obtained by slow vapor diffusion of *n*-hexane (1 mL) into a solution of K_2[**1**] in THF (48 mmol L^{-1}).

Note: When solutions of K_2[**1**] in THF-d_8 are exposed to ambient air, the subsequently recorded NMR spectra show exclusively the resonances of **1** and no signals assignable to any decomposition products.

*NMR shifts of compound K_2[**1**]*

^{1}H NMR (500.2 MHz, THF-d_8): δ = 7.05 (d, $^3J_{HH}$ = 7.6 Hz, 4H; H_h), 6.73 (s, 4H; H_c), 6.65 (t, $^3J_{HH}$ = 7.6 Hz, 2H; H_i), 1.60 (s, 24H; H_e).

^{11}B NMR (160.5 MHz, THF-d_8): δ = 20.0 (br, $h_{1/2}$ = 400 Hz).

^{13}C{^{1}H} NMR (125.8 MHz, THF-d_8): δ = 150.5 (C_b), 149.1 (C_g), 143.4* (br, C_f), 134.4* (br, C_a), 122.9 (C_h), 122.1 (C_i), 114.6 (C_c), 42.2 (C_d), 36.2 (C_e).
*) The positions of these signals were confirmed by cross-peaks in the ^{1}H/^{13}C-HMBC NMR spectrum.

1.3.4 Reduction of **1** in different THF derivatives and ethers

In order to investigate the solvent dependence of the reduction chemistry of **1**, reductions were carried out in different THF derivatives and ethers.

General procedure:
In a glovebox, **1** (6.0 mg, 12 µmol, 1 eq.) was placed in a screw-cap vial, followed by the addition of the respective solvent (0.6 mL). Li granules (15 mg, exc.) or K pieces (20 mg, exc.) were added to the suspension, and the reaction mixture was stirred overnight. The resulting dark solution was transferred to a pointed flask, and all volatiles were removed under reduced pressure. The solid residue was re-dissolved in THF-d_8, transferred to an NMR tube, and the tube was then flame-sealed. The reaction outcomes are compiled in Table S2.

Table S2: Outcomes of reduction experiments on **1**, conducted in various ethers.

**Ether**	**Reducing agent**	**Reaction outcome**
2-Methyl-THF	Lithium	quant. Li_2[**1**]
2,5-Dimethyl-THF	Lithium	quant. Li_2[**1**]
2,2,5,5-Tetramethyl-THF	Lithium	Li[**1**] precipitates
2,2,5,5-Tetramethyl-THF	Potassium	quant. K_2[**1**]
1,4-Dioxane	Lithium	quant. Li_2[**1**]
Tetrahydropyran	Lithium	quant. Li_2[**1**]
Et_2O	Lithium	Li[**1**] precipitates
DME	Lithium	Li[**1**] precipitates
H_3COtBu	Lithium	Li[**1**] precipitates
$Me_3SiOSiMe_3$	Lithium	Li[**1**] precipitates

1.3.5 Synthesis of Li[**1**]

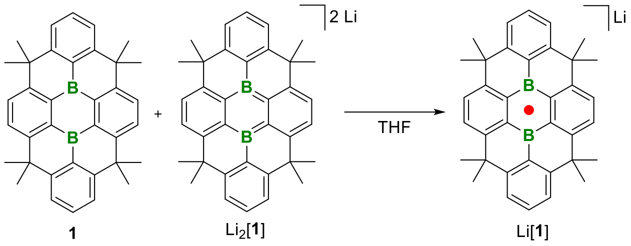

In a glovebox, **1** (4.0 mg, 8.2 µmol, 1 eq.) was reduced to Li_2[**1**] in THF (0.4 mL) using the protocol described above. The dark green reaction solution containing Li_2[**1**] was combined with an equimolar amount of **1** (4.0 mg, 8.2 µmol, 1 eq.). The screw-cap vial used for the reduction was rinsed with THF (0.1 mL), and the rinse was added to the reaction mixture. After stirring for 5 min at room temperature, the solution was transferred to an NMR tube, which was subsequently flame-sealed under vacuum. ^{1}H NMR spectroscopy revealed an NMR silent sample, which was consequently examined by EPR spectroscopy. Details regarding the preparation of an EPR sample of Li[**1**] are provided in paragraph 5, while the corresponding EPR and UV-vis spectra are presented in paragraphs 5 and 4, respectively.

Note: Evidence for the identity of Li[**1**] is provided by the following observations: (i) Quantitative regeneration of the neutral compound **1** occurs upon exposure of Li[**1**] in THF to ambient air (NMR spectroscopic control). (ii) Addition of further Li metal quantitatively converts Li[**1**] to Li_2[**1**].

X-ray-quality single crystals of [Li(12-c-4)$_2$][**1**] were obtained by slow diffusion of a solution of 12-crown-4 (12-c-4) in THF (approx. 200 mmol L^{-1}) into a solution of Li[**1**] in THF (26 mmol L^{-1}) . *Note*: Prior to use, 12-c-4 was dried by storing it over molecular sieves (3 Å) for several d.

Note: The corresponding potassium salt was synthesized *via* an analogous comproportionation reaction of K_2[**1**] and **1**, using the same method as described above.

X-ray-quality single crystals of [K(2.2.2crypt)][**1**] were obtained by slow diffusion of a solution of 2.2.2-cryptand in THF (approx. 100 mmol L^{-1}) into a solution of K[**1**] in THF (30 mmol L^{-1}) .

Note: When solutions of Li[**1**] or K[**1**] in THF-*d*$_8$ are exposed to ambient air, the subsequently recorded NMR spectra show exclusively the resonances of **1** and no signals assignable to any decomposition products.

1.3.6 Insights into the electronic structures of **1**, [**1**]$^{\bullet-}$, and [**1**]$^{2-}$

1.3.6.1 Bond length changes upon reduction of **1**

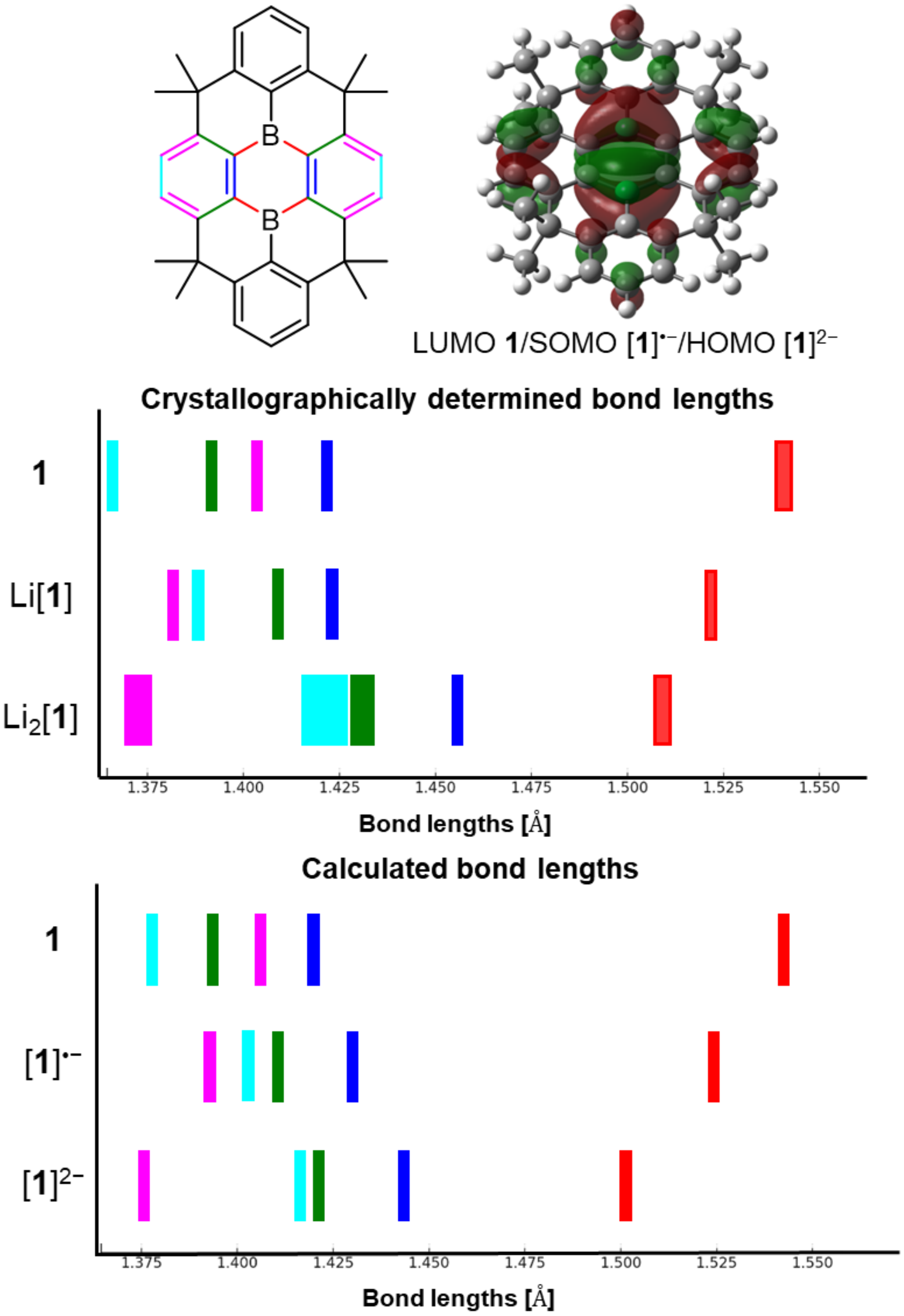

Figure S1: Top left: Color code for the respective selected bonds. Top right: The LUMO of **1** has the same nodal structure as the SOMO of [**1**]$^{\bullet-}$ and the HOMO of [**1**]$^{2-}$. Middle and bottom: Crystallographically determined and calculated bond lengths of **1**, [**1**]$^{\bullet-}$, and [**1**]$^{2-}$ (values in Tables S3 and S4). If the respective frontier orbital predicts a bonding interaction, bond shortening occurs upon reduction. Conversely, if an antibonding interaction is predicted, the bond elongates. This effect is generally more pronounced in two-electron reductions compared to one-electron reductions.

Table S3: Crystallographically determined lengths of selected bonds in **1**, Li[**1**], and Li_2[**1**] (see Figure S1 for the color code). Ranges are given for bond lengths that should be identical due to symmetry, but are in fact different due to crystal-packing effects.

	**1** [Å]	Li[**1**] [Å]	Li_2[**1**] [Å]
**Bond**	1.5382 (3) − 1.5428 (2)	1.5211 (1)	1.5066 (1) – 1.5114 (1)
**Bond**	1.4214 (3)	1.4222 (1)	1.4543 (1) – 1.4564 (1)
**Bond**	1.3907 (2) – 1.3923 (2)	1.4089 (1)	1.4272 (1) – 1.4336 (1)
**Bond**	1.4029 (2) – 1.4037 (3)	1.3809 (1)	1.3683 (1) – 1.3767 (1)
**Bond**	1.3645 (3)	1.3879 (1)	1.4133 (1) – 1.4274 (1)

Table S4: Calculated lengths of selected bonds in **1**, [**1**]$^{•-}$, and [**1**]$^{2-}$.

	**1** [Å]	[**1**]$^{•-}$ [Å]	[**1**]$^{2-}$ [Å]
**Bond**	1.5437	1.5271	1.5036
**Bond**	1.4224	1.4322	1.4432
**Bond**	1.3965	1.4136	1.4211
**Bond**	1.4098	1.3950	1.3764
**Bond**	1.3872	1.4066	1.4194

1.3.6.2 $^{13}C\{^1H\}$ NMR shift changes upon reduction of Mes_2-DBA and **1**

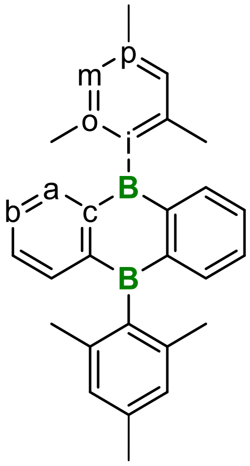

Table S5: $^{13}C\{^1H\}$ NMR shifts δ (75.5 MHz, THF-d_8) of C(sp^2) atoms of Mes_2-DBA and Li_2[Mes_2-DBA]; shift differences $\Delta\delta$ (Mes_2-DBA − Li_2[Mes_2-DBA]).[S4]

**Carbon atom**	**Mes_2-DBA**	**Li_2[Mes_2-DBA]**	**$\Delta\delta$ (Mes_2-DBA − Li_2[Mes_2-DBA])**
C_a	139.6	137.5	2.1
C_b	134.3	118.8	15.5
C_c	146.1	134.9	11.2
C_i	141.6	148.3	-6.7
C_o	138.3	141.5	-3.2
C_m	127.8	127.7	0.1
C_p	137.4	133.3	4.1

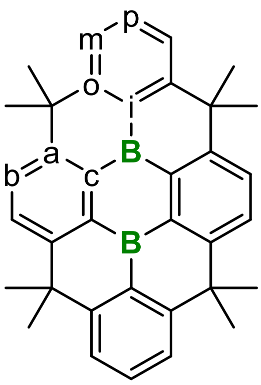

Table S6: $^{13}C\{^1H\}$ NMR shifts δ (125.8 MHz, THF-d_8) of C(sp^2) atoms of **1** and Li_2[**1**]; shift differences $\Delta\delta$ (**1** − Li_2[**1**]).

Carbon atom	1	Li_2[1]	$\Delta\delta$ (1 − Li_2[1])
C_a	155.0	151.1	3.9
C_b	132.0	116.6	15.4
C_c	138.6	129.3	9.3
C_i	n.o.	140.9	-
C_o	157.6	149.3	8.3
C_m	124.9	122.3	2.6
C_p	133.6	124.4	9.2

Table S7: $^{13}C\{^1H\}$ NMR shifts δ (125.8 MHz, THF-d_8) of C(sp^2) atoms of **1** and Na_2[**1**]; shift differences $\Delta\delta$ (**1** − Na_2[**1**]).

Carbon atom	1	Na_2[1]	$\Delta\delta$ (1 − Na_2[1])
C_a	155.0	150.9	4.1
C_b	132.0	115.2	16.8
C_c	138.6	132.4	6.2
C_i	n.o.	142.8	-
C_o	157.6	149.0	8.6
C_m	124.9	122.8	2.1
C_p	133.6	122.6	11.0

Table S8: $^{13}C\{^1H\}$ NMR shifts δ (125.8 MHz, THF-d_8) of C(sp^2) atoms of **1** and K_2[**1**]; shift differences $\Delta\delta$ (**1** − K_2[**1**]).

Carbon atom	1	K_2[1]	$\Delta\delta$ (1 − K_2[1])
C_a	155.0	150.5	4.5
C_b	132.0	114.6	17.4
C_c	138.6	134.4	4.2
C_i	n.o.	143.4	-
C_o	157.6	149.1	8.5
C_m	124.9	122.9	2.0
C_p	133.6	122.1	11.5

1.3.6.3 Conclusion

In addition to molecular-structure elucidation, these tools also provide experimental insight into the charge-density distribution within [**1**]$^{2-}$ compared to [Mes_2-DBA]$^{2-}$: In $^{13}C\{^1H\}$ NMR spectroscopy, the shielding of a specific arene carbon atom increases with the π-electron density at that position and is largely unaffected by the solvent employed.[S4] We first observe that the spectra of all three compounds, M_2[**1**], are nearly identical, ruling out any appreciable counter-cation effect on the electronic structures of the dianions in solution (though not on their reactivity, as discussed below). When comparing trends in the spectra of **1**/M_2[**1**] vs. Mes_2-DBA/M_2[Mes_2-DBA], we find that reduction leads to similarly pronounced upfield shifts of the ^{13}C resonances of their DBA cores. In contrast, the effects on the B-appended six-membered rings differ remarkably (Tables S5 – S8): for **1**/[**1**]$^{2-}$, the absolute differences $\Delta\delta(^{13}C)$ are higher than for [Mes_2-DBA]/[Mes_2-DBA]$^{2-}$, indicating that the B-bonded substituent contributes to the delocalization of the injected electrons only when the ring is forced into planarity with the DBA moiety. A similar picture is gained from the solid-state structures. In previous studies,[S4] we have demonstrated that reduction of B-doped PAHs leads to bond-length alterations that mirror the nodal structure of the LUMO and HOMO of the neutral and reduced forms, respectively: whenever a bonding [antibonding] interaction is indicated in these frontier orbitals, reduction leads to a shortening [elongation] of the respective bonds. The same trends are also evident in both the experimentally determined and the computed bond lengths of the **1**/[**1**]$^{2-}$ pair (Figure S1, Tables S3 and S4), albeit this effect is more pronounced in the DBA core and less so in the planarized B-bonded substituent.

The experimentally determined and calculated bond-length variations $\Delta = d([\mathbf{1}]^{\bullet-}) - d(\mathbf{1})$ match the nodal structure of the SOMO of [**1**]$^{\bullet-}$, but the absolute Δ values are smaller than in the case of the **1**/[**1**]$^{2-}$ pair (Figure S1; Tables S3 and S4).

Note: The solid-state structures of [Li(12-c-4)$_2$][**1**] and [K(2.2.2crypt)][**1**] both exhibit this intermediate bond-length alteration. However, the crystallographic data for [K(2.2.2crypt)][**1**] is more reliable (see Tables S13 and S18, respectively).

1.4 Reactivity of Li[**1**] and Li_2[**1**] toward H-atom donors

1.4.1 Reaction of Li[**1**] with 1,4-cyclohexadiene

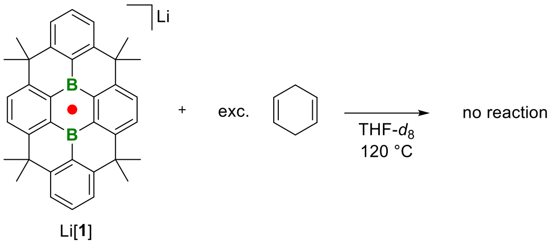

In a glovebox, Li[**1**] (12.3 µmol, 1 eq.) was synthesized in THF-d_8 (0.4 mL) following the protocol described above. In a separate screw-cap vial, 1,4-cyclohexadiene (4.7 µL, 4.0 mg, 50 µmol, 4 eq.) was dissolved in THF-d_8 (0.1 mL). The two solutions were combined and transferred to an NMR tube, which was then flame-sealed under vacuum. Even after gradually increasing the temperature up to 120 °C, no reaction was detected in the ^{1}H NMR spectrum (Figure S15).

1.4.2 Reaction of Li[**1**] with *n*Bu_3SnH

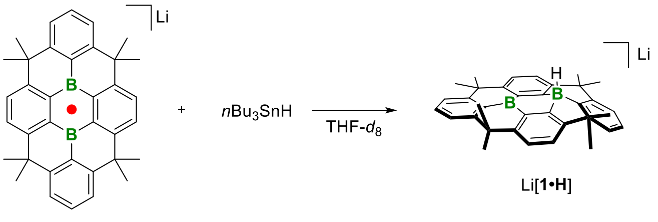

In a glovebox, Li[**1**] (13.5 µmol, 1 eq.) was synthesized in THF-d_8 (0.4 mL), following the protocol described above. In a separate screw-cap vial, *n*Bu_3SnH (5.4 µL, 5.9 mg, 20 µmol, 1.5 eq.) was dissolved in THF-d_8 (0.1 mL). The two solutions were combined and transferred to an NMR tube, which was subsequently flame-sealed under vacuum. Since no reaction was observed at room temperature, the reaction mixture was subsequently heated to 50 °C overnight. NMR spectroscopy revealed that Li[**1**•H] and *n*Bu_6Sn_2 were by far the major products (Figures S16 – S17).

*NMR shifts of compound Li[**1**•H]*

^{1}H{^{11}B} NMR (300.0 MHz, THF-*d*$_8$): δ = 7.55 – 7.51 (m, 4H), 7.48 – 7.44 (m, 1H), 7.24 (d, $^{3}J_{HH}$ = 8.1 Hz, 2H), 7.14 (d, $^{3}J_{HH}$ = 7.6 Hz, 2H), 6.82 (t, $^{3}J_{HH}$ = 7.6 Hz, 1H), 3.39* (s, 1H; B*H*), 1.91 (s, 6H), 1.77 (s, 6H), 1.61 (s, 6H), 1.52 (s, 6H).

^{11}B NMR (96.3 MHz, THF-*d*$_8$): δ = −20.0** (d, $^{1}J_{BH}$ = 75 Hz); n.o. BAr_3.

*) This signal was observed only in the ^{1}H{^{11}B} NMR spectrum.
**) The doublet observed in the ^{11}B NMR spectrum collapses to a singlet in the ^{11}B{^{1}H} NMR spectrum (Figure S17).

Note: Attempts to purify Li[**1**•H] were unsuccessful. Precipitation with *n*-hexane, followed by drying the resulting solid under reduced pressure, afforded a sample in which Li[**1**•H] was only a minor component, as revealed by the ^{1}H{^{11}B} NMR spectrum. All data indicates a redistribution of the hydride ligands forming neutral **1** and Li_2[**1**•2H].[S5] Consequently, all NMR spectra were recorded on the crude reaction mixture, and no ^{13}C NMR spectrum was measured.

nBu_6Sn_2 was observed as a side product in the ^{119}Sn{^{1}H} NMR spectrum. However, due to the small excess of *n*Bu_3SnH used, clear assignments of ^{1}H NMR shifts could not be made. The ^{119}Sn NMR shift of *n*Bu_6Sn_2 is provided below and matches the value reported in the literature (Figure S18):[S6]

^{119}Sn{^{1}H} NMR (149.3 MHz, THF-*d*$_8$): δ = −83.2 (s).

1.4.3 Reaction of Li_2[**1**] with *n*Bu_3SnH

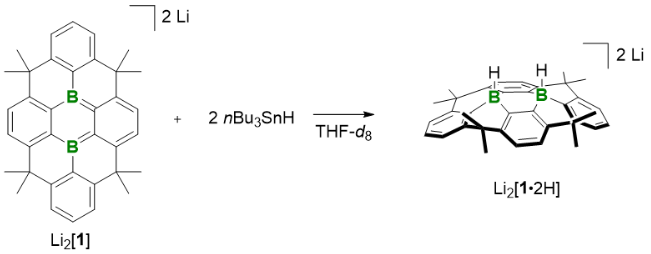

In a glovebox, Li_2[**1**] (14.5 µmol, 1 eq.) was synthesized in THF-d_8 (0.4 mL), following the protocol described above. In a separate screw-cap vial, *n*Bu_3SnH (8.1 µL, 8.9 mg, 31 µmol, 2.1 eq.) was dissolved in THF-d_8 (0.1 mL). The two solutions were combined and transferred to an NMR tube, which was subsequently flame-sealed under vacuum. NMR spectroscopy revealed the selective formation of Li_2[**1**•2H] (alongside *n*Bu_6Sn_2; Figures S19 – S20).

*NMR shifts of compound Li[**1**•2H]*

^{1}H{^{11}B} NMR (300.0 MHz, THF-*d*$_8$): δ = 7.07 – 7.05 (m, 8H), 6.75 (t, $^3J_{HH}$ = 7.5 Hz, 2H), 2.95* (s, 2H; B*H)*, 1.84 (s*,* 12 H), 1.55** (s, 12H).

^{11}B NMR (96.3 MHz, THF-*d*$_8$): δ = −20.6*** (d, $^1J_{BH}$ = 75 Hz); n.o. BAr_3.

*) This signal was observed only in the ^{1}H{^{11}B} NMR spectrum.
**) This signal overlaps with resonances of *n*Bu_3SnH and *n*Bu_6Sn_2.
***) The doublet observed in the ^{11}B NMR spectrum collapses to a singlet in the ^{11}B{^{1}H} NMR spectrum (Figure S20).

Note: Attempts to purify Li_2[**1**•2H] by crystallization were unsuccessful. Consequently, all NMR spectra were recorded on the crude reaction mixture, and no ^{13}C NMR spectrum was measured.

nBu_6Sn_2 was observed as a side product in the ^{119}Sn{^{1}H} NMR spectrum. Due to the small excess of *n*Bu_3SnH used, clear assignments of ^{1}H NMR shifts could not be made. The ^{119}Sn NMR shift of *n*Bu_6Sn_2 is provided below and matches the value reported in the literature:[S6]

^{119}Sn{^{1}H} NMR (149.3 MHz, THF-*d*$_8$): δ = −83.2 (s).

1.5 Reactions of Li_2[**1**] with different fluorobenzene derivatives

1.5.1 Reaction of Li_2[**1**] with C_6F_6

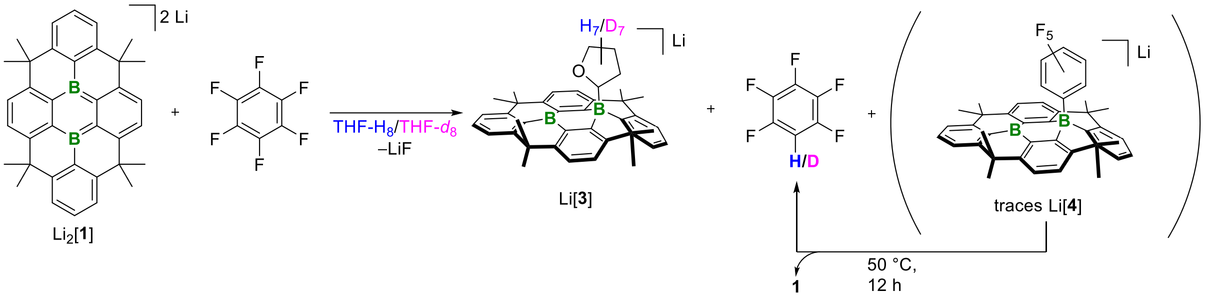

In a glovebox, **1** (8.0 mg, 16 µmol, 1 eq.) was reduced to Li_2[**1**] in THF or THF-d_8 as described above. In a separate screw-cap vial, C_6F_6 (2.8 µL, 4.5 mg, 24 µmol, 1.5 eq.) was dissolved in THF or THF-d_8 (0.05 mL). The dark green solution of Li_2[**1**] was then added slowly at room temperature to the C_6F_6 solution. The reaction mixture exhibited a color change from dark green to dark blue to colorless within approximately 10 s, indicating completion of the reaction. The colorless solution was transferred to an NMR tube, which was subsequently flame-sealed in vacuum (Figure S21).

Main products of the reaction in THF:

NMR-spectroscopy showed the formation of two primary products, Li[**3**] and C_6F_5H, along with the presence of Li[**4**] as a minor side product (Figures S22, S32 and S33). The formation of Li[**4**] depends on concentration: in more concentrated samples, the relative amount of Li[**4**] is higher, whereas in dilute samples, approximately 90–95 % of Li_2[**1**] is converted to Li[**3**] with only 10–5 % forming Li[**4**]. Li[**4**] is thermolabile: when the respective initial reaction mixture was heated to 50 °C for 12 h, Li[**4**] eliminated the C_6F_5 substituent, resulting in the quantitative formation of neutral **1** and C_6F_5H (^{19}F NMR spectroscopic control; Figure S32). *Note*: The corresponding potassium salt, K[**4**], has been isolated and fully characterized (paragraph 1.7.3, Figures S75 – S78).

Main products of the reaction in THF-d_8:

Conducting the reaction in THF-d_8 instead of THF furnishes identical products in a similar distribution, with the main difference being that the positions marked in pink in the Scheme above are now predominantly deuterated. This deuteration experiment confirms that the H atom in C_6F_5H indeed originates from the solvent (Figure S34). *Note*: Despite the use of THF-d_8, some C_6F_5H (approx. 20 % relative to the amount of C_6F_5D formed) could still be observed in the ^{19}F NMR spectrum: we find two sets of signals with very similar chemical shift values for C_6F_5D and C_6F_5H,[S6–8] with each set consisting of three multiplets assignable to the *o*-, *m*-, and *p*-F atoms (Figures S36). Upon proton decoupling, the $C_6\mathit{F}_5D$ resonances remain unchanged, while the *o-F* signal of $C_6\mathit{F}_5H$ partially collapses into a simpler multiplet in the $^{19}F\{^1H\}$ NMR spectrum (Figures S35). The reason for the formation of C_6F_5H is likely that THF-d_8 is not completely deuterated; the remaining H atoms are preferentially abstracted by the $C_6F_5^{\bullet}$ radical due to the kinetic isotope effect.

X-ray quality single crystals of [Li(thf)$_3$][**3**] were obtained by slow evaporation of a solution of Li[**3**] in THF (20 mmol L^{-1}; inert conditions).

*NMR shifts of Li[**3**].*

Note: These spectra were not recorded directly from the reaction mixture but rather from recrystallized Li[**3**] (Figures S22 – S27).

^{1}H NMR(500.2 MHz, THF-d_8): δ = 7.60 (d, $^3J_{HH}$ = 8.0 Hz, 1H), 7.60 (d, $^3J_{HH}$ = 7.6 Hz, 1H), 7.58 (d, $^3J_{HH}$ = 8.3 Hz, 1H), 7.55 (d, $^3J_{HH}$ = 8.4 Hz, 1H), 7.53 (dd, $^3J_{HH}$ = 7.6 Hz, $^3J_{HH}$ = 8.0 Hz, 1H), 7.38 (d, $^3J_{HH}$ = 8.3 Hz, 1H), 7.35 (d, $^3J_{HH}$ = 8.4 Hz, 1H), 7.30 (d, $^3J_{HH}$ = 7.8 Hz, 1H), 7.29 (d, $^3J_{HH}$ = 7.7 Hz, 1H), 7.02 (dd, $^3J_{HH}$ = 7.8 Hz, $^3J_{HH}$ = 7.7 Hz, 1H), 3.06 (dd, $^3J_{HH}$ = 7.6 Hz, $^3J_{HH}$ = 7.4 Hz, 1H), 3.02 – 2.98 (m, 1H), 2.75 – 2.71 (m, 1H), 1.90 (s, 3H), 1.89 (s, 3H), 1.82 (s, 3H), 1.77 (s, 3H), 1.66 (s, 3H), 1.63 (s, 3H), 1.60 (s, 3H), 1.56 (s, 3H), 1.24 – 1.18 (m, 1H), 1.09 – 1.02 (m, 1H), 1.02 – 0.96 (m, 1H), 0.95 – 0.89 (m, 1H).

^{7}Li NMR (194.4 MHz, THF-d_8) δ = −1.8.

^{11}B NMR (160.5 MHz, THF-d_8): δ = −18.3 (s, $Ar_3B{-}C_4H_7O$); n.o. Ar_3B.

^{13}C{^{1}H} NMR (125.8 MHz, THF-d_8): δ = 169.5* (br), 160.1* (br), 157.2, 157.0, 153.0, 152.9, 151.7* (br), 150.0, 149.9, 146.9, 146.1, 137.2 (br), 136.4* (br), 133.6 (br), 131.3, 129.1, 128.7, 124.2, 124.0, 123.2, 123.2, 122.9, 121.7, 121.5, 91.7* (br), ca. 67**, 42.5, 42.5, 42.5, 42.5, 37.2, 36.3, 35.9, 35.4 (2 x C), 35.0, 34.8, 34.3, 29.4, 27.4.

*) This signal is severely broadened and was only detected in the ^{1}H/^{13}C-HMBC experiment.

**) This signal overlaps with the THF-d_8 solvent signal and was detected in the ^{1}H/^{13}C-HMBC experiment.

Assignment: C_4H_7O substituent

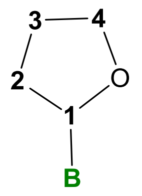

^{1}H NMR (600.2 MHz, THF-d_8): δ = 3.06 (dd, $^3J_{HH}$ = 7.6 Hz, $^3J_{HH}$ = 7.4 Hz, 1H; H_1), 3.02 – 2.98 & 2.75 – 2.71 (2 x m, 2 x 1H; 2 x H_4), 1.24 – 1.18 & 1.02 – 0.96 (2 x m, 2 x 1H; 2 x H_2), 1.09 – 1.02 & 0.95 – 0.89 (2 x m, 2 x 1H; 2 x H_3).

^{13}C{^{1}H} NMR (125.8 MHz, THF-d_8): δ = 91.7 (br; C_1), ca. 67 (C_4), 29.4 (C_2), 27.4 (C_3).

Assignment: DBA

According to the 2D NMR experiments, $^{1}H/^{13}C$ signals assigned the same color belong to the same spin systems:

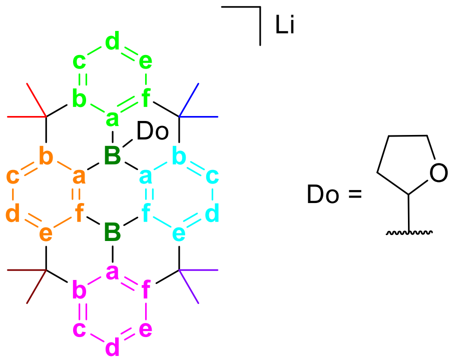

^{1}H NMR(600.2 MHz, THF-d_8): δ = 7.60 (d, $^{3}J_{HH}$ = 8.0 Hz, 1H; H_c or H_e) & 7.60 (d, $^{3}J_{HH}$ = 7.6 Hz, 1H; H_c or H_e) & 7.53 (dd, $^{3}J_{HH}$ = 7.6 Hz, $^{3}J_{HH}$ = 8.0 Hz, 1H; H_d), 7.58 (d, $^{3}J_{HH}$ = 8.3 Hz, 1H; H_c) & 7.38 (d, $^{3}J_{HH}$ = 8.3 Hz, 1H; H_d), 7.55 (d, $^{3}J_{HH}$ = 8.4 Hz, 1H; H_c) & 7.35 (d, $^{3}J_{HH}$ = 8.4 Hz, 1H; H_d), 7.30 (d, $^{3}J_{HH}$ = 7.8 Hz, 1H; H_c or H_e) & 7.29 (d, $^{3}J_{HH}$ = 7.7 Hz, 1H; H_c or H_e) & 7.02 (dd, $^{3}J_{HH}$ = 7.7 Hz, $^{3}J_{HH}$ = 7.8 Hz, 1H; H_d), 1.90* & 1.56 (2 x s, 2 x 3H; C*Me*$_2$), 1.89* & 1.60 (2 x s, 2 x 3H; C*Me*$_2$), 1.82 & 1.63 (2 x s, 2 x 3H; C*Me*$_2$), 1.77 & 1.66 (2 x s, 2 x 3H; C*Me*$_2$).

*) This signal showed a cross-peak with the H_1 and H_4 proton resonances of the C_4H_7O substituent in the NOESY experiment and can therefore be assigned to the two adjacent Me groups situated on the same side as the substituent. The assignment of the C*Me* signals at 1.90 and 1.89 ppm allows for the distinction between the upper and lower halves of the molecule, based on the ^{3}J-couplings to C_b, C_b, C_b and C_f observed in the ^{1}H-^{13}C-HMBC NMR spectrum.

$^{13}C\{^{1}H\}$ NMR (125.8 MHz, THF-d_8): δ = 169.5 (br; C_a) & 153.0 (C_e) & 146.9 (C_b) & 137.2 (br; C_f) & 129.1 (C_c) & 121.7 (C_d), 160.1 (br; C_a) & 152.9 (C_e) & 146.1 (C_b) & 136.4 (br; C_f) & 128.7 (C_c) & 121.5 (C_d), 157.2 (C_b or C_f) & 157.0 (C_b or C_f) & 133.6 (br; C_a) & 131.3 (C_d) & 124.2 (C_c or C_e) & 124.0 (C_c or C_e), 151.7* (br; C_a) & 150.0 (C_b or C_f) & 149.9 (C_b or C_f) & 123.2 (C_c or C_e) & 123.2 (C_c or C_e) & 122.9 (C_d), 42.5 & 42.5 & 42.5 & 42.5 (4 x CMe$_2$), 37.2 & 35.4 (C*Me*$_2$), 36.3 & 35.9 (C*Me*$_2$), 35.4 & 34.3 (C*Me*$_2$), 35.0 & 34.8 (C*Me*$_2$).

*NMR shifts of Li[**3^D**].*

Note: These spectra were not recorded directly from the reaction mixture but rather from recrystallized Li[**3^D**] (Figures S28 – S31).

^{1}H NMR(500.2 MHz, THF-d_8): δ = 7.60 (d, $^3J_{HH}$ = 8.0 Hz, 1H), 7.60 (d, $^3J_{HH}$ = 7.6 Hz, 1H), 7.58 (d, $^3J_{HH}$ = 8.3 Hz, 1H), 7.55 (d, $^3J_{HH}$ = 8.4 Hz, 1H), 7.53 (dd, $^3J_{HH}$ = 7.6 Hz, $^3J_{HH}$ = 8.0 Hz, 1H), 7.38 (d, $^3J_{HH}$ = 8.2 Hz, 1H), 7.34 (d, $^3J_{HH}$ = 8.4 Hz, 1H), 7.30 (d, $^3J_{HH}$ = 7.8 Hz, 1H), 7.29 (d, $^3J_{HH}$ = 7.7 Hz, 1H), 7.03 (dd, $^3J_{HH}$ = 7.8 Hz, $^3J_{HH}$ = 7.7 Hz, 1H), 1.90 (s, 3H), 1.89 (s, 3H), 1.82 (s, 3H), 1.77 (s, 3H), 1.65 (s, 3H), 1.63 (s, 3H), 1.60 (s, 3H), 1.56 (s, 3H).

^{7}Li NMR (194.4 MHz, THF-d8) δ = −1.8.

^{11}B NMR (160.5 MHz, THF-d_8): δ = −18.4 (s, $Ar_3B{-}C_4D_7O$); n.o. Ar_3B.

^{13}C{^{1}H} NMR (125.8 MHz, THF-d_8): δ = 169.9* (vbr), 159.7* (vbr), 157.2, 157.0, 153.0, 152.9, 152.4* (vbr), 150.0, 149.9, 146.8, 146.0, 137.2 (br), 136.4 (br), 133.5 (br), 131.3, 129.1, 128.7, 124.2, 124.0, 123.3, 123.2, 122.9, 121.7, 121.5, 42.5, 42.5, 42.5, 42.5, 37.1, 36.2, 35.9, 35.4 (2 x C), 35.0, 34.8, 34.3.

*) This signal is severely broadened and was only detected in the ^{1}H/^{13}C-HMBC experiment.

Assignment: THF-substituent

Due to the perdeuteration of the C_4D_7O substituent of Li[**3^D**], no signals from this group were observed in the ^{1}H NMR spectrum. Additionally, no ^{13}C NMR resonances of C_4D_7O were detected, likely owing to significant signal broadening resulting from (unresolved) $^nJ_{DC}$ coupling.

Assignment: DBA

Due to the absence of H atoms in the C_4D_7O substituent, NOESY experiments could not be conducted, precluding a definitive assignment based on this 2D NMR spectrum. Given the close similarity of ^{1}H and ^{13}C NMR shifts, with only minor differences, the assignment from Li[**3**] was applied.

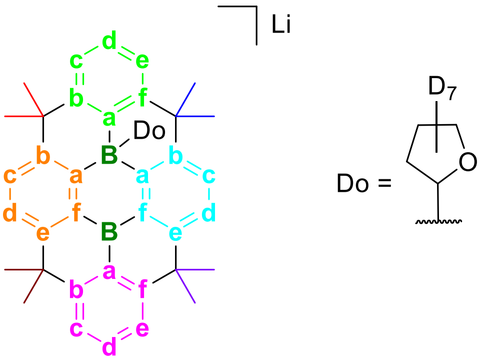

^{1}H NMR (600.2 MHz, THF-d_8): δ = 7.60 (d, $^3J_{HH}$ = 8.0 Hz, 1H; H_c or H_e) & 7.60 (d, $^3J_{HH}$ = 7.6 Hz, 1H; H_c or H_e) & 7.53 (dd, $^3J_{HH}$ = 7.6 Hz, $^3J_{HH}$ = 8.0 Hz, 1H; H_d), 7.58 (d, $^3J_{HH}$ = 8.3 Hz, 1H; H_c) & 7.38 (d, $^3J_{HH}$ = 8.2 Hz, 1H; H_d), 7.55 (d, $^3J_{HH}$ = 8.4 Hz, 1H; H_c) & 7.34 (d, $^3J_{HH}$ = 8.4 Hz, 1H; H_d), 7.30 (d, $^3J_{HH}$ = 7.8 Hz, 1H; H_C or H_e) & 7.29 (d, $^3J_{HH}$ = 7.7 Hz, 1H; H_C or H_e) & 7.03 (dd, $^3J_{HH}$ = 7.7 Hz, $^3J_{HH}$ = 7.8 Hz, 1H; H_d), 1.90 & 1.56 (2 x s, 2 x 3H; C*Me*$_2$), 1.89 & 1.60 (2 x s, 2 x 3H; C*Me*$_2$), 1.82 & 1.63 (2 x s, 2 x 3H; C*Me*$_2$), 1.77 & 1.65 (2 x s, 2 x 3H; C*Me*$_2$).

^{13}C{^{1}H} NMR (125.8 MHz, THF-d_8): δ = 169.9* (br; C_a) & 153.0 (C_e) & 146.8 (C_b) &137.2 (br; C_f) & 129.1 (C_c) & 121.7 (C_d), 159.7* (br; C_a) & 152.9 (C_e) & 146.0 (C_b) & 136.4 (br; C_f) & 128.7 (C_c) & 121.5 (C_d), 157.2 (C_b or C_f) & 157.0 (C_b or C_f) & 133.6 (br; C_a) & 131.3 (C_d) & 124.2 (C_c or C_e) & 124.0 (C_c or C_e), 151.4* (br; C_a) & 150.0 (C_b or C_f) & 149.9 (C_b or C_f) & 123.3 (C_c or C_e) & 123.2 (C_c or C_e) & 122.9 (C_d), 42.5 & 42.5 & 42.5 & 42.5 (4 x *C*Me$_2$), 37.1 & 35.4 (C*Me*$_2$), 36.2 & 35.9 (C*Me*$_2$), 35.4 & 34.3 (C*Me*$_2$), 35.0 & 34.8 (C*Me*$_2$).

NMR shifts of C_6F_5H

Note: C_6F_5H is well-documented in the literature; therefore, only its ^{19}F NMR spectrum is presented (Figure S33), and its ^{1}H NMR spectrum has been omitted.

^{1}H NMR (300.0 MHz, THF-d_8): δ = 7.47 – 7.34 (m).

Note: To unequivocally identify the ^{1}H NMR shifts of C_6F_5H, Li_2[**1**] was reacted with 1 eq. of C_6F_6 in THF. Upon completion of the reaction, all volatile compounds were trapped under reduced pressure in an NMR tube, which was cooled to −196 °C, to isolate C_6F_5H. This process allowed for the determination of the ^{1}H NMR shifts of C_6F_5H, as otherwise, signal overlap with Li[**3**] would occur.

^{19}F NMR (282.3 MHz, THF-d_8): δ = −140.0 – (−140.2) (m, 2F), −156.2 (t, $^3J_{FF}$ = 20.0 Hz, 1F), −164.0 – (−164.2) (m, 2F).

The signals match those published in the literature.[S8,S11]

NMR shifts of C_6F_5D

^{19}F NMR (282.3 MHz, THF-d_8): δ = −140.4 – (−140.5) (m, 2F), −156.3 (t, $^3J_{FF}$ = 20.0 Hz, 1F), −164.2 – (−164.3) (m, 2F).

The signals match those published in the literature.[S8]

*NMR shifts of Li[**4**]*

Note: Li[**4**] appears in this reaction only as a minor side product and could not be isolated (Figure S32); therefore, only its ^{19}F NMR shifts are provided. K[**4**] was successfully isolated, and a complete characterization of this compound can be found in Chapter 1.7.3.

^{19}F NMR (282.3 MHz, THF-d_8): δ = −127.7 – (−127.8) (m, 1F), −168.5 (t, $^3J_{FF}$ = 22 Hz, 1F), −168.8 – (−168.9) (m, 2F).

1.5.2 General protocol for the reactions of Li_2[**1**] with fluorobenzenes

In a glovebox, a solution of Li_2[**1**] in THF or THF-d_8 (0.4 mL) was slowly added to a screw-cap vial containing a solution of the respective fluorobenzene in THF or THF-d_8 (0.05 mL). The reaction mixture was subsequently transferred to an NMR tube and flame-sealed under vacuum. Upon storage of the tube at a temperature T-1 for a duration of t-1, decolorization of the reaction mixture was observed, indicating the completion of the reaction (Table S3).

THF as the solvent

The reaction mixture was analyzed by ^{19}F NMR spectroscopy and then heated to a temperature T-2 for a duration of t-2. All volatile compounds were subsequently trapped under reduced pressure in a second NMR tube, which was cooled to −196 °C to isolate the respective hydrodefluorination product. (a) The solid residue was re-dissolved in THF-d_8 (0.4 mL) and further analyzed by ^{1}H NMR spectroscopy. (b) Concentrated samples of the trapped hydrodefluorination products were obtained by distillation using a Vigreux column; however, complete removal of THF was not achieved. THF-d_8 (0.35 mL) was added to the concentrated sample, and the resulting solution was re-analyzed by ^{1}H and ^{19}F NMR spectroscopy.

THF-d_8 as the solvent

The reaction mixture was analyzed by ^{1}H and ^{19}F NMR spectroscopy and then heated to a temperature T-2 for a duration of t-2. Afterwards, the sample was re-analyzed by NMR spectroscopy. The NMR tube was opened and stored under ambient conditions for 1 d, whereupon ^{1}H NMR spectroscopy revealed the selective and quantitative regeneration of compound **1** in all cases.

Details of the quantities used, as well as the respective reaction temperatures (T-1, T-2) and reaction times (t-1, t-2), are provided in Table S9.

Table S9: Quantities of Li_2[**1**] and fluorobenzenes used in the individual reactions. T-1 and t-1 refer to the temperature and time required to complete the reaction, as indicated by the complete decolorization of the reaction mixture. T-2 and t-2 refer to the temperature and time required to eliminate the fluorinated substituent from the respective thermolabile side product (Li[**4**], Li[**1•C_6F_4H**], or Li[**6**], and Li[**1•$C_6F_3H_2$**]) resulting in the quantitative formation of the corresponding hydrodefluorination product and compound **1**. Color code for entries 0-7: green – selective single hydrodefluorination; black – unselective reaction; blue – selective nucleophilic aromatic substitution.

Entry	Li_2[1] [µmol]	Fluorobenzene [µL, mg, µmol]	T-1; t-1	T-2; t-2
**0**	16 µmol	C_6F_6 (2.8 µL, 4.5 mg, 24 µmol)	rt; 10 s	50 °C; 12 h
**1**	16 µmol	C_6F_5H (2.2 µL, 3.3 mg, 20 µmol)	rt; 12 h	50 °C; 12 h
**2**	16 µmol	1,2,4,5-$C_6F_4H_2$ (2.2 µL, 2.9 mg, 19 µmol)	80 °C; 12 h	100 °C; 3 d
**3**	16 µmol	1,2,3,4-$C_6F_4H_2$ (2.1 µL, 2.9 mg, 20 µmol)	80 °C; 12 h	100 °C; 3 d
**4**	15 µmol	1,2,3,5-$C_6F_4H_2$ (2.0 µL, 2.7 mg, 18 µmol)	80 °C; 2 d	-
**5**	15 µmol	1,2,3-$C_6F_3H_3$ (1.9 µL, 2.0 mg, 18 µmol)	80 °C; 2 d	-
**6**	19 µmol	1,2,4-$C_6F_3H_3$ (2.3 µL, 2.5 mg, 22 µmol)	80 °C; 2 d	-
**7**	23 µmol	1,3,5-$C_6F_3H_3$ (3.5 µL, 4.5 mg, 34 µmol)	100 °C; 3 d	thermally stable up to 120 °C

General observations

- All reactions of Li_2[**1**] with fluorobenzenes yield the same product distributions and proceed with identical reaction rates, even when the reactions are carried out in the dark: all room-temperature reactions were repeated in an argon-filled glovebox under dark conditions, using sealed brown-glass flasks that were additionally wrapped completely in aluminum foil, with the lights switched off.
- Fluorobenzenes with a higher fluorine load are more reactive than those with a lower fluorine load (cf. T-1 and t-1 across the entries).
- All reactions in which Li[**3**] is formed as a product exhibit a distinct color change from deep green to deep blue before becoming colorless. In cases where Li[**3**] is not observed, the reaction mixture transitions directly from deep green to colorless, without the intermediate deep blue stage.
- The primary products vary across the entries: for entries 0–3, the reactions afford Li[**3**], a fluorobenzene that underwent a selective single F/H exchange, and a nucleophilic substitution product with a B-bonded fluorophenyl substituent. Upon heating to T-2, these substitution products undergo thermolysis to give compound **1** and the respective free fluorobenzene (Scheme S1). Under the conditions of entry 4, an unselective reaction occurs, affording two distinct substitution products, at either the F_2 or F_3 position, along with minor formation of Li[**3**] and hydrodefluorination products (Scheme S2). Entries 5 and 6 also represent unselective reactions, primarily producing nucleophilic substitution products, though the exact substitution sites remain unclear (Scheme S2). In the case of entry 7, the nucleophilic substitution product, Li[**2**], is formed quantitatively and remains stable up to 120 °C.
- A kinetic isotope effect is observed for all hydrodefluorination reactions conducted in THF-d_8, increasingly favoring H over D incorporation as the number of F substituents on the benzene ring decreases (Figures S47).
- Compound **1** can be regenerated by exposing Li[**3**] to ambient air for 1 d (re-isolation in 95% yield), highlighting its potential utility in catalytic applications (see Chapter 1.9 for more details).

Entries 1-3: selective hydrodefluorination reactions

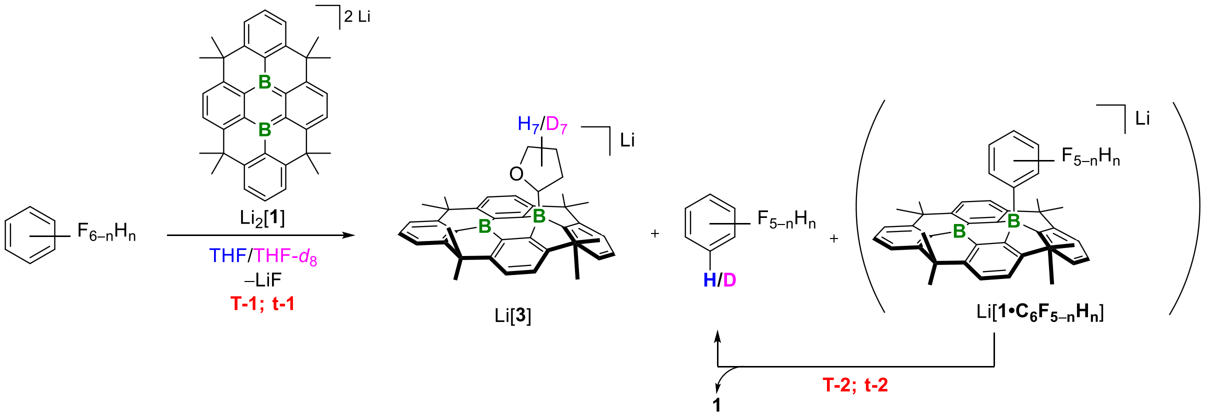

Scheme S1: General outcomes of the reaction of Li_2[**1**] with $C_6F_{6-n}H_n$ (n = 0, 1, 2).

Reaction with C_6F_5H

Reaction outcome with THF as the solvent

^{19}F NMR spectroscopy on the crude reaction mixture revealed two major products: 1,2,4,5-$C_6F_4H_2$ (65 %) and the Li[C_6F_4H] adduct of **1** Li[**1**·C_6F_4H] (35 %). Upon heating to 50 °C for 12 h, the thermolabile Li[**1**·C_6F_4H] undergoes elimination of its C_6F_4H substituent, leading to the formation of additional 1,2,4,5-$C_6F_4H_2$ (^{19}F NMR spectroscopic control; Figures S37 and S38 show the NMR spectra of isolated 1,2,4,5-$C_6F_4H_2$).
After workup under inert conditions, ^{1}H NMR spectroscopy (THF-d_8) confirmed the formation of Li[**3**] and **1**.

Reaction outcome with THF-d_8 as the solvent

After heating to 50 °C for 12 h, ^{1}H and ^{19}F NMR spectroscopy on the crude reaction mixture revealed three major products: Li[**3^D**] and 1,2,4,5-$C_6F_4H_2$ / 1,2,4,5-C_6F_4DH (45 % / 55 % ratio). Compound **1** was observed as a byproduct (Figure S39). Compound **1** could be (almost) fully regenerated after the reaction mixture was exposed to ambient air for 1 d (Figure S40).

NMR shifts of 1,2,4,5-$C_6F_4H_2$

^{1}H NMR (300.0 MHz, THF-d_8): δ = 7.46 (vquint, $^3J_{HF} \approx {}^4J_{HF} \approx$ 8.9 Hz)

^{19}F NMR (282.3 MHz, THF-d_8): δ = −142.7 (vt, $^3J_{FH} \approx {}^4J_{FH} \approx$ 8.9 Hz)

The signals match those published in the literature.[S7]

NMR shifts of 1,2,4,5-C_6F_4DH (Figure S47; bottom)

^{19}F NMR (282.3 MHz, THF-d_8): δ = −142.3 – (−142.4) (m, 2F), −142.7 – (−142.8) (m, 2F).

The signals match those published in the literature.[S11]

*NMR shifts of Li[**1**·C_6F_4H]*

^{19}F NMR (282.3 MHz, THF-d_8): δ = −129.2 – (−129.3) (m, 2F), −170.8 – (−171.1) (m, 2F).

Note: Li[**1**·C_6F_4H] is a side product of the reaction and could not be isolated. Therefore, only ^{19}F NMR shifts are provided.

Reaction with 1,2,4,5-$C_6F_4H_2$

Note: The reaction leading to F/H exchange commenced already at 50 °C, as indicated by *in situ* ^{19}F NMR spectroscopy and a color change from deep green to deep blue.

Reaction outcome with THF as the solvent

^{19}F NMR spectroscopy (THF) on the crude reaction mixture revealed two major products: 1,2,4-$C_6F_3H_3$ (50 %) and Li[**6**] (50 %). Upon heating to 100 °C for 3 d, the thermolabile Li[**6**] undergoes elimination of its $C_6F_3H_2$ substituent, leading to the formation of additional 1,2,4-$C_6F_3H_3$ (^{19}F NMR spectroscopic control; Figures S41 and S42 show the NMR spectra of isolated 1,2,4-$C_6F_3H_3$).
After workup under inert conditions, ^{1}H NMR spectroscopy (THF-d_8) confirmed the formation of Li[**3**] and **1**.

Reaction outcome with THF-d_8 as the solvent

After heating to 100 °C for 3 d, ^{1}H and ^{19}F NMR spectroscopy on the crude reaction mixture revealed three major products: Li[**3**^{D}] and 1,2,4-$C_6F_3H_3$ / 1,2,4-F_3-5-D-C_6H_2 (60% / 40 % ratio). Compound **1** was observed as a byproduct (Figure S43). Compound **1** could be (almost) fully regenerated after the reaction mixture was exposed to ambient air for 1 d (Figure S44).

NMR shifts of 1,2,4 -$C_6F_3H_3$

^{1}H NMR (300.0 MHz, THF-*d*$_8$): *δ* = 7.35 – 7.24 (m, 1H), 7.24 – 7.13 (m, 1H), 6.99 – 6.91 (m, 1H).

^{19}F NMR (282.3 MHz, THF-*d*$_8$): *δ* = −118.3 – (−118.4) (m, 1F), −136.9 – (−137.1) (m, 1F), −146.8 – (−147.0) (m, 1F).

The signals match those published in the literature.[S8,S11]

NMR shifts of 1,2,4-F_3-5-D-C_6H_2 (Figure S47; middle)

^{19}F NMR (282.3 MHz, THF-*d*$_8$): *δ* = −118.6 – (−118.7) (br. m, 1F), −137.0 – (−137.2) (m, 1F), −146.9 – (−147.0) (m, 1F).

The signals match those published in the literature.[S11]

*NMR shifts of Li[**6**]*

^{19}F NMR (282.3 MHz, THF-*d*$_8$): *δ* = −104.5 – (−104.6) (m, 1F), −149.8 – (−150.0) (m, 1F), −152.8 – (−153.0) (m, 1F).

Note: Li[**6**] is a side product of the reaction and could not be isolated. Therefore, only ^{19}F NMR shifts are provided. The corresponding potassium salt, K[**6**], has been isolated, and a complete characterization of this compound can be found in Chapter 1.7.2.

Reaction with 1,2,3,4-$C_6F_4H_2$

Note: The reaction leading to F/H exchange commenced already at rt, as indicated by *in situ* ^{19}F NMR spectroscopy and a color change from deep green to deep blue.

Reaction outcome with THF as the solvent

^{19}F NMR spectroscopy (THF) on the crude reaction mixture revealed two major products: 1,2,4-$C_6F_3H_3$ (65 %) and Li[**1**·1,2,4-$C_6F_3H_2$] (35 %). Upon heating to 100 °C for 3 d, the thermolabile Li[**1**·1,2,4$C_6F_3H_2$] undergoes elimination of its $C_6F_3H_2$ substituent, leading to the formation of additional 1,2,4-$C_6F_3H_3$ (^{1}H and ^{19}F NMR spectroscopic control; Figures S41 and S42 show the NMR spectra of isolated 1,2,4-$C_6F_3H_3$).

After workup under inert conditions, ^{1}H NMR spectroscopy (THF-*d*$_8$) confirmed the formation of Li[**3**] and **1.**

Reaction outcome with THF-d_8 as the solvent

After heating to 100 °C for 3 d, ^{1}H and ^{19}F NMR spectroscopy on the crude reaction mixture revealed three major products: Li[**3**^{D}] and 1,2,4-$C_6F_3H_3$ / 1,2,4-F_3-3-D-C_6H_2 (60 % / 40 % ratio). Compound **1** was observed as a byproduct (Figure S45). Compound **1** could be (almost) fully regenerated after the reaction mixture was exposed to ambient air for 1 d (Figure S46).

NMR shifts of 1,2,4-F_3-3-D-C_6H_2 (Figure S47; top)

^{19}F NMR (282.3 MHz, THF-d_8): δ = −118.5 – (−118.7) (br. m, 1F), −137.2 – (−137.3) (m, 1F), −146.8 – (−147.0) (m, 1F).

The signals match those published in the literature.[S11]

*NMR shifts of Li[**1**·1,2,4-$C_6F_3H_2$]*

^{19}F NMR (282.3 MHz, THF-d_8): δ = −131.6 – (−131.8) (m, 1F), −165.1 – (−165.3) (m, 1F), −171.4 – (−171.7) (m, 1F).

Note: Li[**1**·1,2,4-$C_6F_3H_2$] is a side product of the reaction and could not be isolated. Therefore, only ^{19}F NMR shifts are provided.

Entries 4-6: unselective reactions

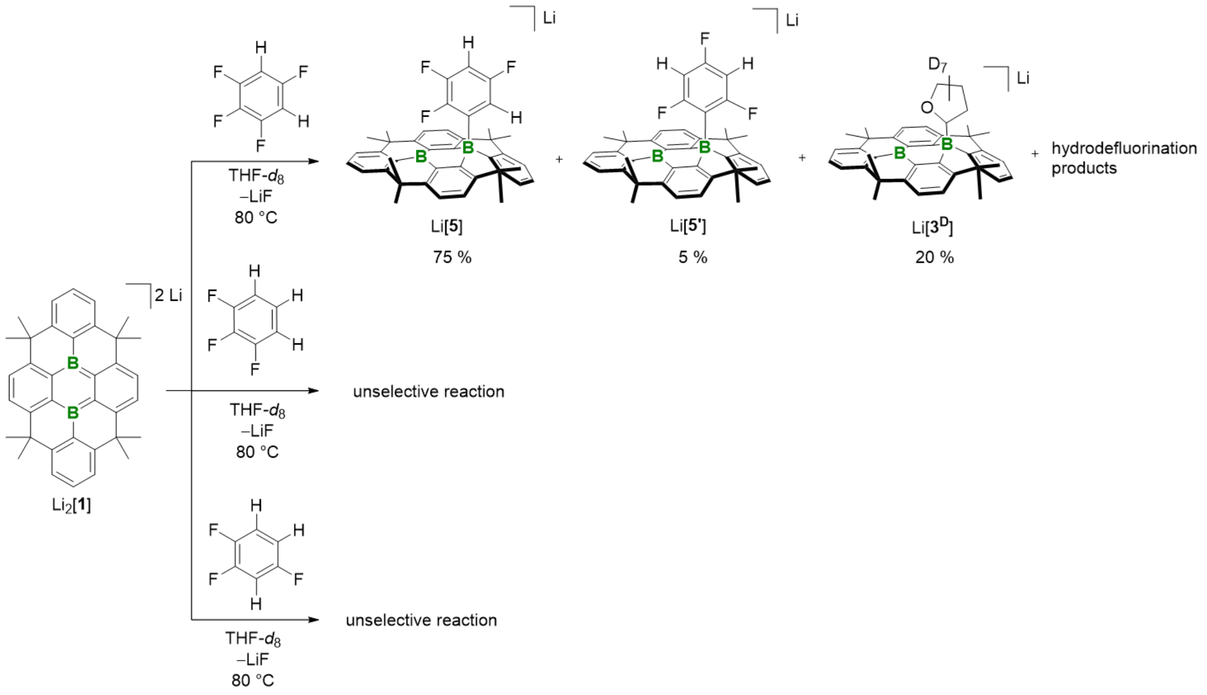

Scheme 2: Unselective reactions of Li$_2$[**1**] with different fluorobenzenes. Bottom: reaction with 1,2,4-$C_6F_3H_3$; middle: reaction with 1,2,3-$C_6F_3H_3$; top: reaction with 1,2,3,5-$C_6F_4H_2$.

Reaction with 1,2,3,5-$C_6F_4H_2$

The reaction was performed according to the general procedure. After combining the two reactants, no color change was observed at room temperature. Consequently, the reaction mixture was gradually heated to 80 °C. After 2 d at 80 °C, the decolorization of the reaction mixture indicated that the reaction had reached completion.

^{1}H and ^{19}F NMR spectroscopy revealed Li[**5**] as the main product (ca. 75 %), along with Li[**5'**] (ca. 5 %), Li[**3^D**] (ca. 20 %), and hydrodefluorination products (Figures S48 – S50).

Note: Slow evaporation of the product mixture from the reaction of Li$_2$[**1**] with 1,2,3,5-$C_6F_4H_2$ over several days afforded X-ray quality co-crystals, enabling the identification of the main product (Li[**5**]) and the side product (Li[**5'**]); for more details, see chapter 6.7). Due to the low abundance of Li[**5'**] in solution, its NMR shifts could not be unambiguously assigned.

However, its higher proportion in the co-crystal allowed for its identification as the side product.

*NMR shifts of Li[**5**]*

Note: Li[**5**] could not be isolated. However, based on the results of single-crystal X-ray diffraction, Li[**5**] is expected to give the most intense signals in the ^{1}H and ^{19}F{^{1}H} NMR spectra. Thus, the corresponding chemical shift values are reported. This assumption is further supported by the ^{19}F{^{1}H} NMR-derived *J*-coupling constants, which were determined from the most intense signals and are consistent with those expected for Li[**5**], as only this species should have the observed number of ^{3}J and ^{4}J couplings. Due to the product mixture, the ^{11}B and ^{13}C NMR signals could not be assigned.

^{1}H NMR (300.0 MHz, THF-d_8): δ = 7.61 – 7.59 (m, 2H), 7.54 – 7.51 (m, 1H), 7.48 (d, $^{3}J_{HH}$ = 8.4 Hz, 2H), 7.41 (d, $^{3}J_{HH}$ = 7.8 Hz, 2H), 7.30 (d, $^{3}J_{HH}$ = 8.4 Hz, 2H), 7.10 (t, $^{3}J_{HH}$ = 7.8 Hz, 1H), 6.03 – 5.90 (m, 2H), 1.78 (s, 6H), 1.75 (s, 6H), 1.69 (s, 6H), 1.39 (s, 6H).

^{19}F{^{1}H} NMR (282.3 MHz, THF-d_8): δ = −124.5 (d, $^{4}J_{FF}$ = 18 Hz), −136.5 (dd, $^{3}J_{FF}$ = 24 Hz, $^{4}J_{FF}$ = 18 Hz), −142.3 (d, $^{3}J_{FF}$ = 24 Hz).

Reaction with 1,2,3,- or 1,2,4-$C_6F_3H_3$

The reactions were performed according to the general procedure. After combining the two reactants, no color change was observed at rt. Consequently, the reaction mixtures were gradually heated to 80 °C. After 2 d at 80 °C, the decolorization of the reaction mixtures indicated that the reactions had reached completion. In both cases, NMR spectroscopic analysis revealed a non-selective reaction, resulting in a variety of products, none of which could be unambiguously identified by NMR spectroscopy (^{11}B NMR: Figures S51 and S52). Therefore, these approaches were not pursued further.

Entry 7: selective nucleophilic aromatic substitution

Reaction with 1,3,5-$C_6F_3H_3$

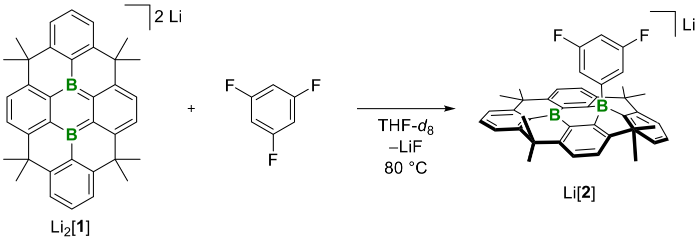

The reaction was performed according to the general procedure. After combining the two reactants, no color change was observed at rt. Consequently, the reaction mixture was gradually heated to 100 °C. After heating the reaction mixture to 100 °C for 3 d, the reaction mixture decolorized, indicating completion. NMR spectroscopy confirmed a quantitative conversion to Li[**2**] (Figures S53 – S59).

X-ray-quality single crystals of [Li(thf)$_4$][**2**] were obtained by slow vapor diffusion of *n*-hexane (1 mL) into a solution of Li[**2**] in THF (42 mmol/l).

*NMR shifts of Li[**2**]*

^{1}H NMR (500.2 MHz, THF-d_8): δ = 7.63 – 7.61 (m, 2H), 7.56 – 7.51 (m, 1H), 7.45 (d, $^{3}J_{HH}$ = 8.4 Hz, 2H), 7.41 (d, $^{3}J_{HH}$ = 7.8 Hz, 2H), 7.28 (d, $^{3}J_{HH}$ = 8.3 Hz, 2H), 7.10 (t, $^{3}J_{HH}$ = 7.8 Hz, 1H), 6.24 (dd, $^{3}J_{HF}$ = 10.4 Hz, $^{4}J_{HH}$ = 2.3 Hz, 2H), 5.72 (tt, $^{3}J_{HF}$ = 9.2 Hz, $^{4}J_{HH}$ = 2.3 Hz, 1H), 1.79 (s, 6H), 1.74 (s, 6H), 1.68 (s, 6H), 1.37 (s, 6H).

^{7}Li NMR (194.4 MHz, THF-d_8): δ = −0.8 (s).

^{11}B NMR (160.5 MHz, THF-d_8): δ = −16.7 (s, Ar^F−BAr_3); n.o. = BAr_3.

^{13}C{^{1}H} NMR (125.8 MHz, THF-d_8): δ = 172.9* (vbr), 171.7* (vbr), 161.8 (dd, $^{1}J_{CF}$ = 245 Hz, $^{3}J_{CF}$ = 10.7 Hz), 157.2, 153.2, 153.0* (vbr), 150.2, 145.5, 136.6 (br), 133.6 (br), 131.1, 127.8, 124.1, 124.0, 122.5, 121.3, 115.6 (dd, $^{2}J_{CF}$ = 15.3 Hz, $^{4}J_{CF}$ = 2.5 Hz), 95.3 (t, $^{2}J_{CF}$ = 26.3 Hz), 42.6, 42.1, 35.9, 35.6, 34.3, 32.7.

*) This signal is severely broadened and was only detected in the ^{1}H/^{13}C-HMBC experiment.

^{19}F NMR (470.6 MHz, THF-d_8): δ = −117.5 (dd, $^{3}J_{FH} \approx {}^{3}J_{FH} \approx$ 10.1 Hz).

Assignment: $C_6F_2H_3$-substituent

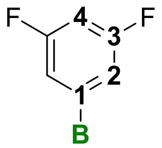

^{1}H NMR (500.2 MHz, THF-d_8): δ = 6.24 (dd, $^3J_{HF}$ = 10.1 Hz, $^4J_{HH}$ = 2.3 Hz, 2H; H_2), 5.72 (tt, $^3J_{HF}$ = 9.2 Hz, $^4J_{HH}$ = 2.3 Hz, 1H; H_4).

^{13}C{^{1}H} NMR (125.8 MHz, THF-d_8): δ = 172.9* (vbr; C_1), 161.8 (dd, $^1J_{CF}$ = 245 Hz, $^3J_{CF}$ = 10.7 Hz; C_3), 115.6 (dd, $^2J_{CF}$ = 15.3 Hz, $^4J_{CF}$ = 2.5 Hz; C_2), 95.3 (t, $^2J_{CF}$ = 26.3 Hz; C_4).

^{19}F NMR (470.6 MHz, THF-d_8): δ = −117.5 (dd, $^3J_{FH} \approx {}^3J_{FH} \approx$ 10.1 Hz).

Assignment: DBA

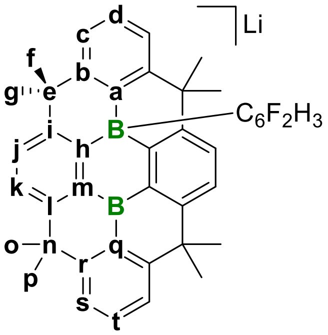

^{1}H NMR (500.2 MHz, THF-d_8): δ = 7.63 – 7.61 (m, 2H; H_s), 7.56 – 7.51 (m, 1H; H_t), 7.45 (d, $^3J_{HH}$ = 8.4 Hz, 2H; H_j), 7.41 (d, $^3J_{HH}$ = 7.8 Hz, 2H; H_c), 7.28 (d, $^3J_{HH}$ = 8.3 Hz, 2H; H_k), 7.10 (t, $^3J_{HH}$ = 7.8 Hz, 1H; H_d), 1.79 (s, 6H, 2 x CH_3; H_o or H_p), 1.74 (s, 6H, 2 x CH_3; H_g), 1.68 (s, 6H, 2 x CH_3; H_o or H_p), 1.37* (s, 6H, 2 x CH_3; H_f).

*) This signal gave a cross-peak with the proton resonances of the $C_6F_2H_3$ substituent (6.24 ppm; H_2) in the NOESY experiment and can therefore be assigned to the two adjacent Me groups situated on the same side as the substituent. The assignment of the C*Me* signal at 1.37 ppm to H_f allows for the distinction between the upper and lower halves of the molecule, based on the ^3J-couplings to C_b and C_i observed in the ^{1}H-^{13}C-HMBC NMR spectrum.

^{13}C{^{1}H} NMR (125.8 MHz, THF-d_8): δ = 171.7 (vbr; C_h), 157.2 (C_r), 153.2 (C_l), 153.0 (vbr; C_a), 150.2 (C_b), 145.5 (C_i), 136.6 (br; C_m), 133.6 (br; C_q), 131.1 (C_t), 127.8 (C_j), 124.1 (C_d), 124.0 (C_s), 122.5 (C_c), 121.3 (C_k), 42.6 (C_n), 42.1 (C_e), 35.9 (C_o or C_p), 35.6 (C_f), 34.3 (C_o or C_p), 32.7 (C_g).

1.5.3 Reaction of Li_2[**1**] with C_6F_6 in THF-Me_2

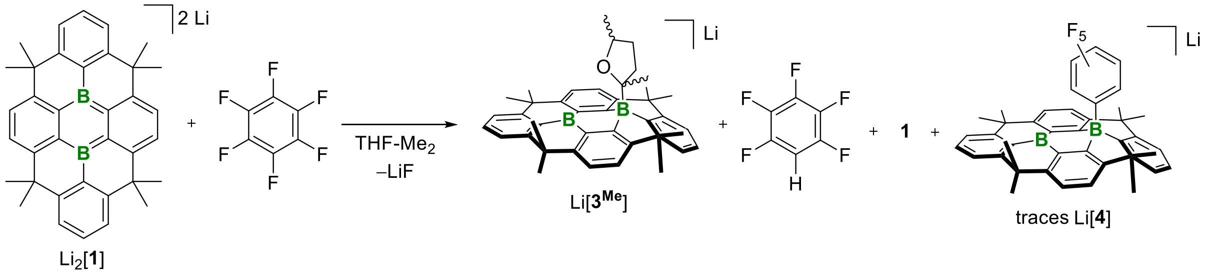

In a glovebox, **1** (7.8 mg, 16 µmol, 1 eq.) was placed in a screw-cap vial, followed by the addition of THF-Me_2 (0.7 mL). Li granules (10 mg, exc.) were added to the suspension, and the reaction mixture was stirred overnight, turning dark red. In a separate screw-cap vial, C_6F_6 (2.8 µL, 4.5 mg, 24 µmol, 1.5 eq.) was dissolved in THF-Me_2 (0.1 mL), and the dark red Li_2[**1**]/THF-Me_2 solution was added dropwise with stirring. The reaction mixture was stirred at room temperature for 10 h, after which time complete decolorization was observed. At this stage, *in situ* ^{19}F NMR spectroscopy confirmed the formation of C_6F_5H along with traces of Li[**4**]. The reaction mixture was transferred to a pointed flask, and all volatiles were removed under reduced pressure. The resulting colorless solid was re-dissolved in THF-d_8 (0.4 mL). ^{1}H NMR spectroscopy confirmed that Li[$\mathbf{3^{Me}}$] (85 %) was by far the major product, while compound **1** (15 %) was also detected (Figure S60).

X-ray-quality single crystals of [Li(thf)$_4$][$\mathbf{3^{Me}}$] were obtained by slow vapor diffusion of *n*-hexane (1 mL) into a solution of Li[$\mathbf{3^{Me}}$] in THF (38 mmol/L).

NMR shifts of Li[$\mathbf{3^{Me}}$]

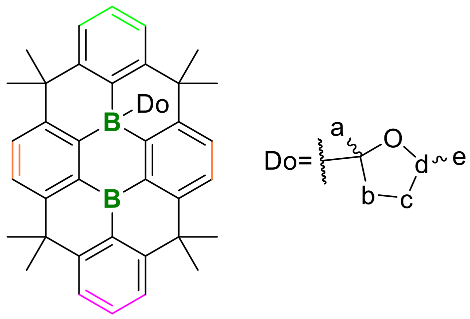

Assignment: $C_4H_5OMe_2$ substituent

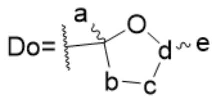

Note: The ^{1}H NMR spectrum reveals the presence of two distinct diastereomers in the reaction mixture, with one being predominant. A dublicated set of signals, corresponding to the diastereomeric pair, was observed for both the substituent and DBA; however, only the substituent exhibited sufficiently large chemical shift differences with minimal signal overlap, allowing for unambiguous differentiation. To determine the relative diastereomer ratio, we

normalized the integral of the CH_3 signal with the highest intensity to 3H (doublet at 0.65 ppm) and found an approximate 2:1 ratio between the major (Figure S60, green triangle) and minor species (Figure S60, blue triangle). The assignments were further corroborated by ^{1}H/^{1}H-COSY correlations.

major diastereomer

^{1}H NMR (400.2 MHz, THF-d_8): δ = 2.28 – 2.19 (m, 1H; H_d), 0.80 – 0.69 (m, 2H; H_c), 0.65 (d, $^3J_{HH}$ = 6.0 Hz, 3H; H_e), 0.38 (s, 3H; H_a), 0.31 – 0.24 (m, 2H; H_b).

minor diastereomer

^{1}H NMR (400.2 MHz, THF-d_8): δ = 3.50 – 3.42 (m, 1H; H_d), 1.27 – 1.20 (m, 2H; H_c), 0.35 (s, 3H; H_a), 0.31 (d, $^3J_{HH}$ = 6.0 Hz, 3H; H_e), 0.24 – 0.14 (m, 2H; H_b).

Assignment: DBA

Note: The ^{1}H NMR spectrum shows a diastereomeric mixture originating from the use of diastereomeric THF-Me_2. The integral of a distinct methyl group of the DBA was set to 3H, which enabled the detection of the expected integral ratios for the DBA unit. While signal splitting into diastereomeric pairs was observed, extensive overlap in the aromatic region prevented the differentiation of individual signals. As a result, these signals were integrated together, and the chemical shifts values were reported as ranges. In the alkyl region, more than eight methyl resonances (detected as singlets with shoulders) were observed, which can be attributed to the presence of diastereomeric pairs (affected signals are marked with an asterisk after each individual shift value given). ^{1}H NMR shifts in the same colors correspond to the same spin system. ^{13}C NMR was not assigned due to extensive signal overlap. The ^{1}H and ^{11}B NMR spectra clearly indicate the formation of Li[**3^{Me}**]. The ^{1}H NMR assignments were based on ^{1}H/^{1}H-COSY data and comparison of shift values with Li[**3**].

^{1}H NMR (400.2 MHz, THF-d_8): δ = 7.57 – 7.55 (m, 2H), 7.49 – 7.45 (m, 1H), 7.41 – 7.37 (m, 2H), 7.23 – 7.20 (m, 2H), 7.16 – 7.14 (m, 2H), 6.91 – 6.86 (m, 1H), 2.09 (s, 3H, CH_3), 2.06 (s, 3H, CH_3), 1.74* (s, 3H, CH_3), 1.70* (s, 3H, CH_3), 1.69 (s, 3H, CH_3), 1.66* (s, 3H, CH_3), 1.39 (s, 3H, CH_3), 1.37 (s, 3H, CH_3).

^{7}Li NMR (155.5 MHz, THF-d_8): δ = −0.8 (s).

^{11}B NMR (128.4 MHz, THF-d_8): δ = −16.6 (s, $Ar_3B{-}C_4H_5OMe_2$); n.o. BAr_3.

*) This signal exhibits a shoulder due to the presence of the second diastereomer.

1.6 Reaction of Na_2[**1**] with C_6F_6

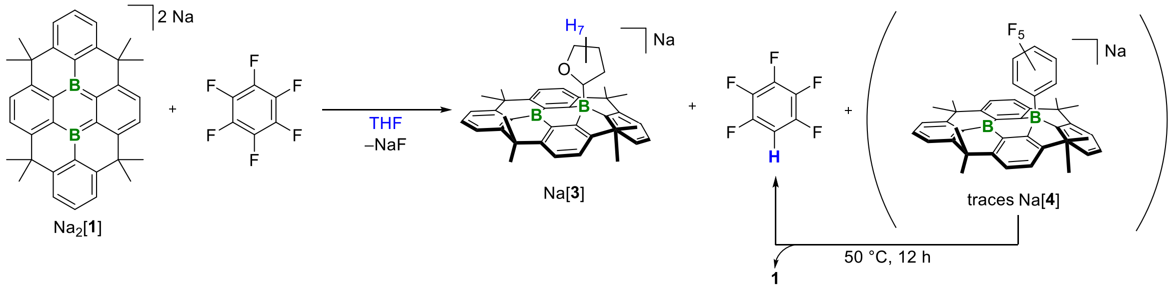

In a glovebox, **1** (8.0 mg, 16 µmol, 1 eq.) was reduced to Na_2[**1**] in THF as described above. In a separate screw-cap vial, C_6F_6 (2.9 µL, 4.6 mg, 25 µmol, 1.5 eq.) was dissolved in THF (0.05 mL) and the dark brown solution of Na_2[**1**] was slowly added. The reaction mixture underwent color change from dark brown to dark blue and then to colorless within approximately 10 s at rt, indicating the reaction had reached completion. The colorless solution was transferred to an NMR tube, which was subsequently flame-sealed under vacuum.

After analyzing the sample by ^{19}F NMR spectroscopy, it was heated to 50 °C for 12 h. The NMR tube was opened inside a glovebox, and the reaction mixture was transferred to a pointed flask, where all volatiles were removed under reduced pressure. The resulting colorless solid was re-dissolved in THF-d_8 (0.4 mL) and transferred to an NMR-tube, which was subsequently flame-sealed under vacuum (^{1}H and ^{11}B NMR spectra: Figures S63 and S64).

^{19}F NMR spectroscopy (THF) showed the formation of C_6F_5H (main product) and Na[**4**] (side product) at rt. After workup under inert conditions, ^{1}H and ^{11}B NMR spectroscopy (THF-d_8) confirmed the formation of Na[**4**] and **1** (Figures S63 and S64).
Note: Na[**4**] is thermolabile: when the initial reaction mixture was heated to 50 °C for 12 h, Na[**4**] eliminated its C_6F_5 substituent, resulting in the quantitative formation of neutral **1** and C_6F_5H (^{19}F NMR spectroscopic control).

Since the NMR spectra were acquired directly from the crude reaction mixture, complete signal assignment was not performed. Nevertheless, a comparison with the fully assigned ^{1}H NMR spectrum of Li[**3**] indicates that Na[**3**] is indeed the predominant product (Figure S65): The aryl region displays a similar set of signals with slightly different chemical shift values. In the range of 3.10 – 2.90 ppm, two multiplets are observed, corresponding to the three H atoms in α positions to the O atom of the B-bonded C_4H_7O substituent. The alkyl region exhibits the characteristic eight Me resonances, consistent with the formation of Na[**3**]. The differences in chemical shift values are attributed to cation effects.

Note: This reaction was conducted exclusively in THF as the solvent, as its primary purpose was a proof of concept. No purification was performed, since the crystallization method used to isolate Li[**3**] was not successful for Na[**3**], and no alternative purification method has been successful to date.

*NMR shift of Na[**3**]*

^{11}B NMR (96.3 MHz, THF-d_8): δ = −20.3 (s, $Ar_3B{-}C_4H_7O$); n.o. Ar_3B.

Note: The reactions of Na_2[**1**] with C_6F_5H, 1,2,4,5-$C_6F_4H_2$ and 1,2,3,4-$C_6F_4H_2$ furnish identical products to those obtained with Li_2[**1**] (see chapter 1.5.2, entries 1-3). Therefore, they are not described here, as they don't provide additional information.

1.7 Reactions of K_2[1] with different fluorobenzene derivatives

1.7.1 Reaction of K_2[1] with C_6F_6

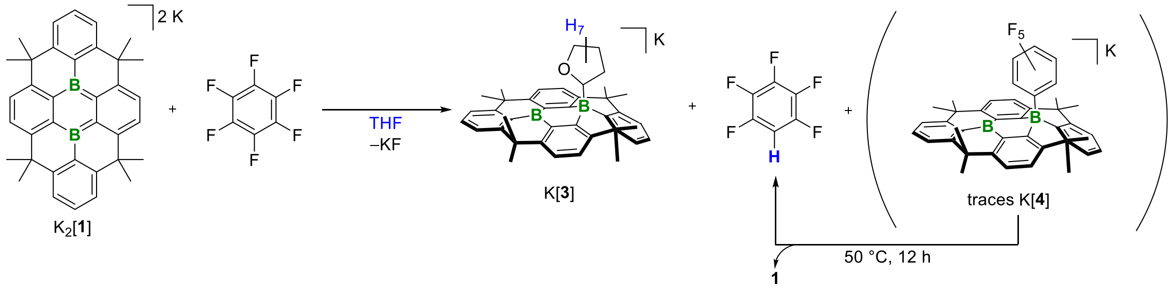

In a glovebox, **1** (8.0 mg, 16 µmol, 1 eq.) was reduced to K_2[**1**] in THF as described above. In a separate screw-cap vial, C_6F_6 (2.9 µL, 4.6 mg, 25 µmol, 1.5 eq.) was dissolved in THF (0.05 mL) and the dark purple solution of K_2[**1**] was slowly added. The reaction mixture underwent color change from dark purple to dark blue and then to colorless within approximately 10 s at rt, indicating the reaction had reached completion. The colorless solution was transferred to an NMR tube, which was subsequently flame-sealed under vacuum.

After analyzing the sample by ^{19}F NMR spectroscopy, it was heated to 50 °C for 12 h. The NMR tube was opened, and the reaction mixture was transferred to a pointed flask, where all volatiles were removed under reduced pressure. The resulting colorless solid was re-dissolved in THF-d_8 (0.4 mL) and transferred to an NMR-tube, which was subsequently flame-sealed under vacuum (^{1}H and ^{11}B NMR spectra: Figures S66 and S67).

^{19}F NMR spectroscopy (THF) showed the formation of C_6F_5H (main product) and K[**4**] (side product) at rt. After workup under inert conditions, ^{1}H and ^{11}B NMR spectroscopy (THF-d_8) confirmed the formation of K[**3**] and **1** (Figures S66 and S67).
Note: K[**4**] is thermolabile: when the initial reaction mixture was heated to 50 °C for 12 h, K[**4**] eliminated its C_6F_5 substituent, resulting in the quantitative formation of neutral **1** and C_6F_5H (^{19}F NMR spectroscopic control).

Since the NMR spectra were acquired directly from the crude reaction mixture, complete signal assignment was not performed. Nevertheless, a comparison with the fully assigned ^{1}H NMR spectrum of Li[**3**] indicates that K[**3**] is indeed the predominant product (Figure S68): The aryl region displays a similar set of signals with slightly different chemical shift values. In the range of 3.05 – 2.85 ppm, a multiplet is observed, corresponding to the three H atoms in α positions to the O atom of the B-bonded C_4H_7O substituent. The alkyl region exhibits the characteristic eight Me resonances, consistent with the formation of K[**3**]. The differences in chemical shift values are attributed to cation effects.

Note: This reaction was conducted exclusively in THF as the solvent, as its primary purpose was a proof of concept. No purification was performed, since the crystallization method used to isolate Li[**3**] was not successful for K[**3**], and no alternative purification method has been successful to date.

*NMR shift of K[**3**]*

^{11}B NMR (96.3 MHz, THF-d_8): δ = −20.0 (s, $Ar_3B{-}C_4H_7O$); n.o. Ar_3B

Note: The reaction of K_2[**1**] with C_6F_5H furnishes identical products to those obtained with Li_2[**1**] (see chapter 1.5.2, entry 1). Therefore, it is not described here, as it does not provide additional information.

1.7.2 Reaction of K_2[1] with 1,2,4,5-$C_6F_4H_2$

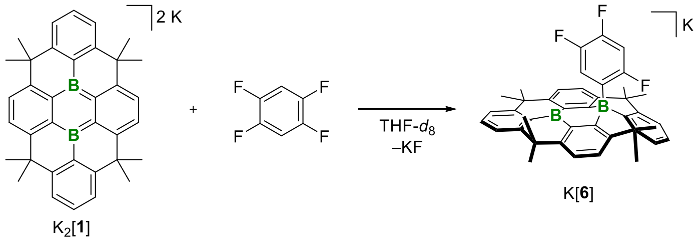

1 (8.2 mg, 17 µmol, 1 eq.) was reduced to K_2[**1**] in THF-d_8 as described above. In a separate screw-cap vial, 1,2,4,5-$C_6F_4H_2$ (2.3 µL, 3.0 mg, 20 µmol, 1.2 eq.) was dissolved in THF-d_8 (0.05 mL) and the dark purple solution of K_2[**1**] was slowly added. The purple solution was transferred to an NMR tube, which was subsequently flame-sealed in vacuum. The reaction mixture underwent color change from dark purple to colorless within about 2 d at rt or after 1 d at 50 °C, indicating the reaction had reached completion. NMR spectroscopy revealed the quantitative formation of K[**6**] (Figures S69 – S74).

X-ray-quality single crystals of [K(thf)$_4$][**6**] were obtained by slow vapor diffusion of *n*-hexane (1 mL) into a solution of K[**6**] in THF (67 mmol/l) and subsequent storage of the mixture at −30 °C for 4 d.

*NMR shifts of K[**6**]*

^{1}H NMR (500.2 MHz, THF-d_8): δ = 7.62 (d, $^3J_{HH}$ = 7.7 Hz, 2H), 7.55 (t, $^3J_{HH}$ = 7.7 Hz, 1H), 7.55 (d, $^3J_{HH}$ = 8.3 Hz, 2H), 7.49 (d, $^3J_{HH}$ = 7.7 Hz, 2H), 7.37 (d, $^3J_{HH}$ = 8.3 Hz, 2H). 7.19 (t, $^3J_{HH}$ = 7.7 Hz, 1H), 6.23 – 6.17 (m, 1H), 5.96 – 5.91 (m, 1H), 1.78 (s, 6H), 1.77 (s, 6H), 1.72 (s, 6H), 1.39 (s, 6H).

^{11}B NMR (160.5 MHz, THF-d_8): δ = −18.1 (s, $Ar^F{-}BAr_3$); n.o. = BAr_3.

^{13}C{^{1}H} NMR (125.8 MHz, THF-d_8): δ = 168.2* (vbr), 161.3 (ddd, $^1J_{CF}$ = 240 Hz, $^3J_{CF}$ = 7.8 Hz, $^4J_{CF}$ = 2 Hz), 157.3, 153.3, 153.3* (vbr), 150.6, 146.6 (ddd, $^1J_{CF}$ = 240 Hz, $^2J_{CF}$ = 16 Hz, $^3J_{CF}$ = 13 Hz), 145.9, 145.8 (ddd, $^1J_{CF}$ = 240 Hz, $^2J_{CF}$ = 11 Hz, $^4J_{CF}$ = 2 Hz), 137.7 (br), 133.2 (br), 131.4, 127.8, 125.0, 124.6 (vt, $^2J_{CF} \approx {}^2J_{CF} \approx$ 16 Hz), 123.9, 123.1, 122.2, 102.9 (dd, $^2J_{CF}$ = 35 Hz, $^3J_{CF}$ = 18 Hz), 42.7, 42.2, 36.4, 35.2, 33.5, 32.2.

*) This signal is severely broadened and was only detected in the ^{1}H-^{13}C-HMBC experiment.

^{19}F NMR (470.6 MHz, THF-d_8): δ = −105.0 – (−105.1) (m, 1F), −149.2 – (−149.3) (m, 1F), −152.5 – (−152.6) (m, 1F).

^{19}F{^{1}H} NMR (470.6 MHz, THF-d_8): δ = −105.0 (dd, $^4J_{FF}$ = 17 Hz, $^5J_{FF}$ = 3 Hz), −149.2 (dd, $^3J_{FF}$ = 22 Hz, $^5J_{FF}$ = 3 Hz), −152.5 (dd, $^3J_{FF}$ = 22 Hz, $^4J_{FF}$ = 17 Hz).

Assignment: $C_6F_3H_2$-substituent

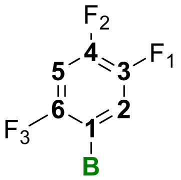

^{1}H NMR (500.2 MHz, THF-d_8): δ = 6.23 – 6.17 (m, 1H; H_5), 5.96 – 5.91 (m, 1H; H_2).

Note: The assignment of H_2 and H_5 is based on the ^3J couplings observed with F-bonded C atoms in the ^{1}H-^{13}C-HMBC NMR spectrum. H_2 has two ^3J couplings to C_4 and C_6, whereas H_5 has only a single ^3J coupling to C_3 (the signal of C_1 is broadened beyond detection).

^{13}C{^{1}H} NMR (125.8 MHz, THF-d_8): δ = 161.3 (ddd, $^1J_{CF}$ = 240 Hz, $^3J_{CF}$ = 7.8 Hz, $^4J_{CF}$ = 2 Hz; C_6), 146.6 (ddd, $^1J_{CF}$ = 240 Hz, $^2J_{CF}$ = 16 Hz, $^3J_{CF}$ = 13 Hz; C_4), 145.8 (ddd, $^1J_{CF}$ = 240 Hz, $^2J_{CF}$ = 11 Hz, $^4J_{CF}$ = 2 Hz; C_3), 124.6 (vt, $^2J_{CF} \approx {}^2J_{CF} \approx$ 16 Hz; C_5), 102.9 (dd, $^2J_{CF}$ = 35 Hz, $^3J_{CF}$ = 18 Hz; C_2); n.o. C_1.

^{19}F NMR (470.6 MHz, THF-d_8): δ = −105.0 – (−105.1) (m, 1F; F_3), −149.2 – (−149.3) (m, 1F; F_1), −152.5 – (−152.6) (m, 1F; F_2).

^{19}F{^{1}H} NMR (470.6 MHz, THF-d_8): δ = −105.0 (dd, $^4J_{FF}$ = 17 Hz, $^5J_{FF}$ = 3 Hz; F_3), −149.2 (dd, $^3J_{FF}$ = 22 Hz, $^5J_{FF}$ = 3 Hz; F_1), −152.5 (dd, $^3J_{FF}$ = 22 Hz, $^4J_{FF}$ = 17 Hz; F_2).

Assignment: DBA

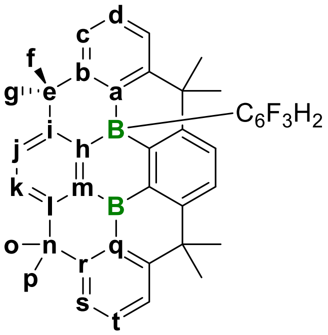

^{1}H NMR (500.2 MHz, THF-d_8): δ = 7.62 (d, $^3J_{HH}$ = 7.7 Hz, 2H; H_s), 7.55 (t, $^3J_{HH}$ = 7.7 Hz, 1H; H_t), 7.55 (d, $^3J_{HH}$ = 8.3 Hz, 2H; H_j), 7.49 (d, $^3J_{HH}$ = 7.7 Hz, 2H; H_c), 7.37 (d, $^3J_{HH}$ = 8.3 Hz, 2H; H_k). 7.19 (t, $^3J_{HH}$ = 7.7 Hz, 1H; H_d), 1.78 (s, 6H, 2 x CH_3; H_g), 1.77 (s, 6H, 2 x CH_3; H_o or H_p), 1.72 (s, 6H, 2 x CH_3; H_o or H_p), 1.39* (s, 6H, 2 x CH_3; H_f).

*) This signal gave two cross-peaks with the proton resonances of the $C_6F_3H_2$ substituent (6.23 – 6.17; H_5 and 5.96 – 5.91; H_2) in the NOESY experiment and can therefore be assigned to the two adjacent Me groups situated on the same side as the substituent. The assignment of the C*Me* signal at 1.39 ppm to H_f allows for the distinction between the upper and lower halves of the molecule, based on the ^3J couplings to C_b and C_i observed in the ^{1}H-^{13}C-HMBC NMR spectrum.

$^{13}C\{^1H\}$ NMR (125.8 MHz, THF-d_8): δ = 168.2 (vbr; C_h), 157.3 (C_l or C_r), 153.3 (C_l or C_r), 153.3 (vbr; C_a), 150.6 (C_b), 145.9 (C_i), 137.7 (br; C_m), 133.2 (br; C_q), 131.4 (C_t), 127.8 (C_j), 125.0 (C_d), 123.9 (C_s), 123.1 (C_c), 122.2 (C_k), 42.7 (C_n), 42.2 (C_e), 36.4 (C_o or C_p), 35.2 (C_f), 33.5 (C_o or C_p), 32.2 (C_g).

1.7.3 Synthesis of K[4] in 2,2,5,5-Me$_4$-THF

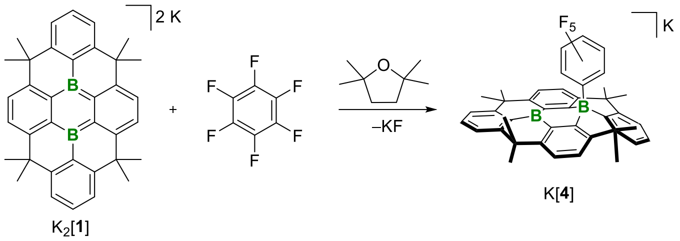

In a glovebox, compound **1** (5.3 mg, 11 µmol, 1 eq.) was suspended in 2,2,5,5-Me$_4$-THF (0.7 mL) in a screw-cap vial containing a stirring bar. Potassium chunks (20 mg, excess) were added, and the reaction mixture was stirred overnight, whereupon it adopted a dark color, indicative of the formation of K$_2$[**1**]. This solution was then separated from the unconsumed K chunks and transferred to a new screw-cap vial. C_6F_6 (3.8 µL, 6.1 mg, 33 µmol, 3 eq.) in 2,5-Me$_2$-THF (0.05 mL) was added, and the reaction mixture was stirred at rt for 3 h. The resulting decolorized solution was transferred to a pointed flask, and all volatiles were removed under reduced pressure. The resulting solid was re-dissolved in THF-d_8 (0.4 mL) and transferred to an NMR tube, which was subsequently flame-sealed.

NMR spectroscopy confirmed the selective formation of K[**4**]·(2,5-Me$_2$-THF)$_{2.5}$ (Figures S75 – S78; the amount of incorporated 2,5-Me$_2$-THF was calculated by integrating the respective ^{1}H NMR signals). However, upon measuring 2D NMR spectra over several hours at rt, conversion to **1** and C_6F_5H was observed, indicating a limited stability of K[**4**].

Note: The addition of small amounts of 2,5-Me$_2$-THF to the reaction mixture was crucial for stabilizing K[**4**] in the solid state after workup, likely due to the enhanced donor properties of 2,5-Me$_2$-THF compared to 2,2,5,5-Me$_4$-THF. Without the addition of 2,5-Me$_2$-THF, significantly higher amounts of **1** were observed after the workup process.

X-ray-quality single crystals of [K(thf)$_2$][**4**] were obtained by slow evaporation of a solution of K[**4**] in THF (20 mmol/l) over several days and subsequent storage at −30 °C for 3 d.

Note: Crystallization of K[**4**] from 2,2,5,5-Me$_4$-THF was unsuccessful due to the limited long-term stability of K[**4**] in weakly donating solvents.

*NMR shifts of K[**4**]*

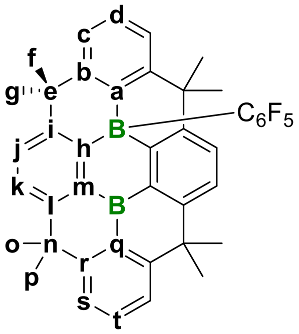

^{1}H NMR (500.2 MHz, THF-d_8): δ = 7.61 (d, $^{3}J_{HH}$ = 7.8 Hz, 2H; H_s), 7.60 (d, $^{3}J_{HH}$ = 8.4 Hz, 2H; H_j), 7.55 – 7.52 (m, 1H; H_t), 7.43 (d, $^{3}J_{HH}$ = 7.9 Hz; 2H; H_c), 7.39 (d, $^{3}J_{HH}$ = 8.4 Hz, 2H; H_k), 7.12 (t, $^{3}J_{HH}$ = 7.9 Hz, 1H; H_d), 1.82 (s, 6H; H_g), 1.78 (s, 6H; H_o or H_p), 1.70 (s, 6H; H_o or H_p), 1.44* (s, 6H; H_f).
*) Due to the absence of H atoms in the C_6F_5 substituent, no NOESY NMR spectrum was measured. The assignment of the C*Me* signal at 1.44 ppm to H_f is based on the observation that, for all DBA compounds carrying a boron-bonded fluoroaryl substituent, the C*Me* signal exhibiting the strongest upfield shift consistently corresponds to H_f. This assumption allows for the distinction between the upper and lower halves of the molecule, based on the ^{3}J-couplings to aromatic quaternary C atoms observed in the ^{1}H-^{13}C-HMBC NMR spectrum.

^{11}B NMR (160.5 MHz, THF-d_8): δ = −17.9 (s, Ar^F−BAr_3); n.o. = BAr_3.

^{13}C{^{1}H} NMR (125.8 MHz, THF-d_8): δ = 165.6* (vbr; C_h), 157.2 (C_r), 152.7 (C_l), 152.9* (vbr; C_a), 150.7 (C_b), 146.9 (C_i), 138.2 (br; C_m), 133.1 (br; C_q), 131.4 (C_t), 127.4 (C_j), 124.8 (C_d), 123.9 (C_s), 122.5 (C_c), 122.4 (C_k), 42.6 (C_n), 42.3 (C_e), 36.3 (C_o or C_p), 34.9 (C_f), 33.6 (C_o or C_p), 31.4 (C_g); n.o. 3 x C_{Ar}−F; n.o $C_{Ar\text{-}F}$−B.
*) This signal is severely broadened and was only detected in the ^{1}H-^{13}C-HMBC experiment.

^{19}F NMR (470.6 MHz, THF-d_8): δ = −127.7 – (−127.8) (m, 2F; *o*-F), −168.5 (t, $^{3}J_{FF}$ = 22 Hz, 1F; *p*-F), −168.8 – (−168.9) (m, 2F; *m*-F).

Note: The ^{13}C NMR shifts of the C_6F_5 substituent could not be unambiguously identified, even by ^{13}C-^{19}F-HSQC NMR spectroscopy. Nevertheless, the presence of (i) three characteristic ^{19}F NMR resonances within a range typical for C_6F_5 substituents attached to a four-coordinate B site,[S12] (ii) a signal at −17.9 ppm in the ^{11}B NMR spectrum confirming a four-coordinate B atom, and (iii) ^{13}C NMR shifts that closely match those observed for K[**6**], a structurally closely related compound, strongly supports the selective formation of K[**4**].

1.8 Mechanistic investigations

1.8.1 Stability of K[**4**]

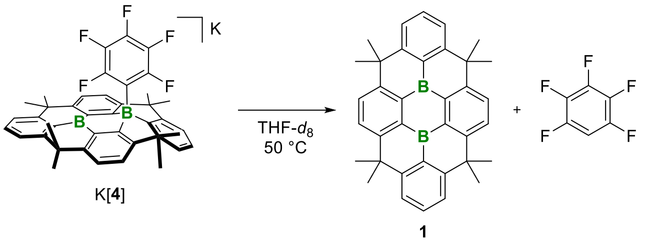

K[**4**]

1

A freshly prepared sample of K[**4**]·(2,5-Me_2-THF)$_{2.5}$ (11 µmol) was dissolved in THF-d_8 (0.4 mL). The solution was transferred to an NMR tube which was subsequently flame-sealed. Heating the sample to 50 °C for 12 h, with periodic monitoring by ^{1}H and ^{19}F NMR spectroscopy, revealed a gradual and selective conversion to **1** and C_6F_5H.

Note: The selective breakdown of K[**4**] to **1** and C_6F_5H can also be observed when the reaction mixture is stored at rt for several days. For convenience, we monitored the reaction at 50 °C to facilitate observation of the process. The corresponding NMR spectra are presented in Figures S79 and S80.

The absence of K[**3**] suggests that K[**3**] is not generated through a closed-shell mechanism involving K[**4**] as an intermediate.

Note: Despite using THF-d_8 as the solvent for this reaction, no formation of C_6F_5D was observed. This is likely due to the presence of 2,5-Me_2-THF in the reaction mixture, which remains coordinated to the K^+ ion after workup of K[**4**]. Due to the kinetic isotope effect, the α-H atoms of 2,5-Me_2-THF are preferentially abstracted.

1.8.2 Reaction of Li[**1**] with C_6F_6

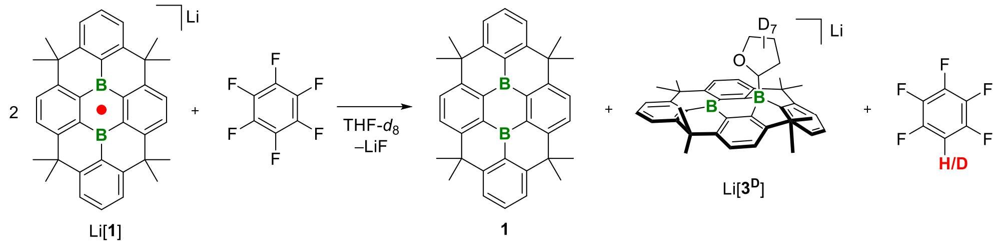

In a glovebox, a freshly prepared sample of Li[**1**] (16 µmol) in THF-d_8 (0.4 mL) was added to a solution of C_6F_6 (2.8 µL, 4.5 mg, 24 µmol, 1.5 eq.) in THF-d_8 (0.05 mL) in a screw-cap vial. The reaction mixture decolorized after 10 s and was transferred to an NMR-tube which was subsequently flame-sealed under vacuum. NMR spectroscopy revealed the formation of **1** and Li[$\mathbf{3^D}$] in a 1:1 ratio. A mixture of C_6F_5H and C_6F_5D formed quantitatively from C_6F_6 (Figure S81).

1.8.3 Reaction of Li[**1**] with 1,3,5-$C_6F_3H_3$

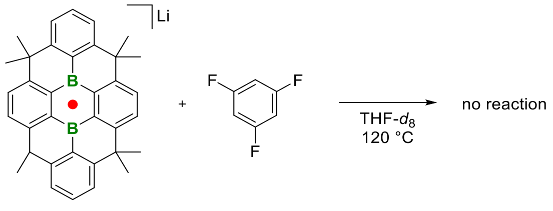

In a glovebox, a freshly prepared sample of Li[**1**] (16 µmol, 1 eq.) in THF-d_8 (0.4 mL) was added to a solution of 1,3,5-$C_6F_3H_3$ (5.1 µL, 6.5 mg, 49 µmol, 3 eq.) in THF-d_8 (0.05 mL) in a screw-cap vial. The deep blue reaction mixture was transferred to an NMR tube, which was subsequently flame-sealed under vacuum. Despite continuous heating up to 120 °C for 2 d, no reaction was observed, as the sample remained NMR silent, indicating that Li[**1**] did not react (Figure S82).

1.8.4 Reaction of Li_2[**1**] with 1,2,4,5-$C_6F_4H_2$ in the presence of 12-c-4

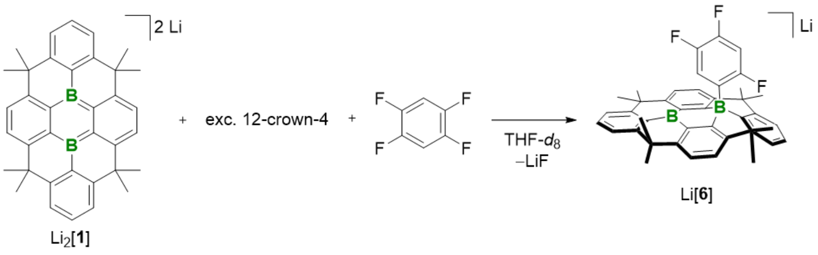

In a screw-cap vial, 12-crown-4 (12-c-4; 20 µL, 22 mg, excess), was added to a freshly prepared sample of Li_2[**1**] (14 µmol, 1 eq.) in THF-d_8 (0.4 mL). The solution was stirred for approximately 10 min before a solution of 1,2,4,5-$C_6F_4H_2$ (1.9 µL, 2.6 mg, 17 µmol, 1.2 eq.) in THF-d_8 (0.05 mL) was added. The reaction was stirred for an additional 30 min before being transferred to an NMR tube, which was subsequently flame-sealed under vacuum. NMR spectroscopy indicated a nucleophilic substitution reaction (Figure S83); however, product characterization was inconclusive due to prominent 12-c-4 signals. After removing volatiles under reduced pressure, the remaining solid was redissolved in THF-d_8 (0.4 mL). NMR spectroscopy subsequently revealed the formation of Li[**6**]·(12-c-4)$_8$, with no formation of Li[**3^D**] observed (Figures S84 – S86; the amount of residual 12-c-4 was calculated by integrating the respective ^{1}H NMR signals).

Note: In the ^{19}F NMR spectrum, only small amounts of 1,2,4-$C_6F_3H_3$ and 1,2,4-F_3-5-D-C_6H_2 were observed. This, along with the absence of Li[**3^D**], suggests that performing the reaction in the presence of 12-c-4 almost completely suppresses the radical pathway that would otherwise occur.

*NMR-shifts of Li[**6**]*·(12-c-4)$_8$

^{1}H NMR: (300.0 MHz, THF-d_8): δ = 7.65 (d, $^3J_{HH}$ = 7.6 Hz, 2H), 7.59 – 7.53 (m, 3H), 7.49 (d, $^3J_{HH}$ = 7.8 Hz, 2H), 7.37 (d, $^3J_{HH}$ = 8.2 Hz, 2H), 7.14 (t, $^3J_{HH}$ = 7.8 Hz, 1H), 6.58 – 6.47 (m, 1H), 6.05 – 5.96 (m, 1H), 1.87 (s, 6H), 1.79 (s, 6H), 1.73* (s, 6H), 1.33 (s, 6H).

*) This signal overlaps with the residual solvent signal of THF-d_8.

^{11}B NMR: (96.3 MHz, THF-d_8): δ = −19.6 (s, Ar^F−BAr$_3$) (n.o. = BAr$_3$).

^{19}F NMR: (282.3 MHz, THF-d_8): δ = −104.1 – (−104.3) (m, 1F), −148.7 – (−148.9) (m, 1F), −152.3 – (−152.5) (m, 1F).

Note: Further purification of Li[**6**]·(12-c-4)$_8$ was not feasible due to very similar solubility of all (side) products. Consequently, all NMR spectra were recorded without additional purification, and neither ^{13}C{^{1}H} nor 2D NMR spectra were measured. However, a comparative analysis of the NMR spectra of the fully characterized compound K[**6**] and Li[**6**], as shown in Fig. S87, confirms their structural identity.

1.8.5 Reaction of Li_2[**1**] with (bromomethyl)cyclopropane

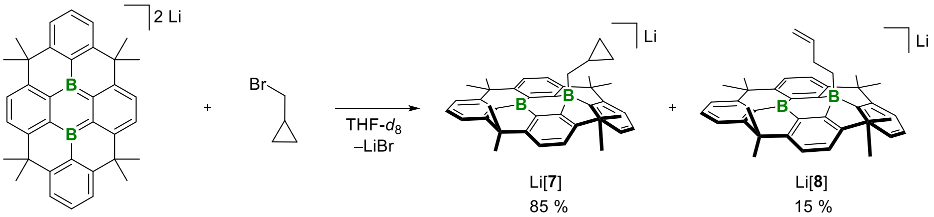

In a screw-cap vial, a sample of Li_2[**1**] (17 µmol, 1 eq.) in THF-d_8 (0.4 mL) was prepared. To the dark green solution, (bromomethyl)cyclopropane (2.0 µL, 2.8 mg, 20 µmol, 1.2 eq.) was added. The reaction mixture decolorized after approximately 20 s. It was filtered to remove inorganic salts and transferred to a pointed flask. All volatiles were removed under reduced pressure. NMR spectroscopy revealed a mixture of Li[**7**] (85%) and Li[**8**] (15%), indicating that $[\mathbf{1}]^{2-}$ preferentially undergoes closed-shell reactions with this sp^3-electrophile (Figures S88 – S92). For NMR shifts of an authentic sample of Li[**8**], see Chapter 1.8.6.

X-ray quality co-crystals of $[Li(12\text{-c-}4)_2]$[**7**] and Li[**8**] were obtained by slow diffusion of 12-c-4 in THF (approx. 150 mmol/l) into a reaction mixture of Li_2[**1**] with (bromomethyl)cyclopropane in THF (32 mmol/l), and subsequent concentration of the solution over a period of several days.

Note: The ratio of $[\mathbf{7}]^-$ and $[\mathbf{8}]^-$ in the crystal structure differs slightly from the ratio observed in solution. Further details can be found in Chapter 6.11.

*NMR shifts of Li[**7**]*

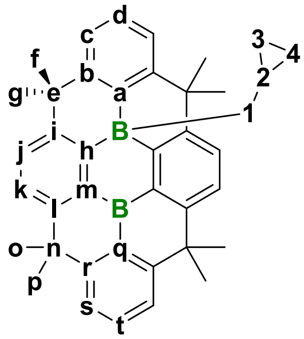

^{1}H NMR (600.0 MHz, THF-d_8): δ = 7.55 (d, $^3J_{HH}$ = 7.8 Hz, 2H; H_s), 7.49 (d, $^3J_{HH}$ = 8.2 Hz, 2H; H_j), 7.46 (t, $^3J_{HH}$ = 7.8 Hz, 1H; H_t), 7.24 (d, $^3J_{HH}$ = 8.2 Hz, 2H; H_k), 7.20 (d, $^3J_{HH}$ = 7.7 Hz, 2H; H_c), 6.88 (t, $^3J_{HH}$ = 7.7 Hz, 1H; H_d), 1.75 (s, 6H; H_o or H_p), 1.73*/** (s, 6H; H_f), 1.70 (s, 6H; H_g), 1.68 (s, 6H; H_o or H_p), 0.10 (d, $^3J_{HH}$ = 6.5 Hz, 2H; H_1), −0.27 - (−0.34) (m, 1H; H_2), −0.57 - (−0.6) (m, 2H; H_{3a} and H_{4a}), −1.11 - (−1.14) (m, 2H; H_{3b} and H_{4b}).

*) This signal overlaps with the residual solvent signal of THF-d_8 (1.73 ppm).

**) This signal gave a cross-peak with the proton resonance of the alkyl substituent (0.10 ppm; H_1) in the NOESY experiment and can therefore be assigned to the two adjacent Me groups situated on the same side as the substituent. The assignment of the C*Me* signal at 1.73 ppm to H_f allows for the distinction between the upper and lower halves of the molecule based on the ^3J-couplings to C_b and C_i observed in the ^{1}H-^{13}C-HMBC NMR spectrum.

^{7}Li NMR (194.4 MHz, THF-d_8): δ = −0.1 (s).

^{11}B NMR (160.5 MHz, THF-d_8): δ = −18.3 (s, C_4H_7−BAr_3) (n.o. = BAr_3).

^{13}C{^{1}H} NMR (125.8 MHz, THF-d_8): δ = 174.9* (vbr.; C_h), 157.8* (vbr.; C_a), 157.1 (C_r), 151.9 (C_l), 149.2 (C_b), 145.0 (C_i), 137.7 (C_m), 134.2 (C_q), 130.5 (C_t), 127.5 (C_j), 123.8 (C_s), 122.4 (2 x C; C_c and C_d), 120.1 (C_k), 45.6* (vbr.; C_1), 42.4 (C_n), 42.0 (C_e), 37.4 (C_f), 36.0 (C_o or C_p), 34.4 (C_o or C_p), 34.0 (C_g), 10.8 (C_2), 6.8 (2 x C; C_3 and C_4).

*) This signal is severely broadened and was only detected in the ^{1}H-^{13}C-HMBC experiment.

1.8.6 Reaction of Li_2[**1**] with 4-bromo-1-butene

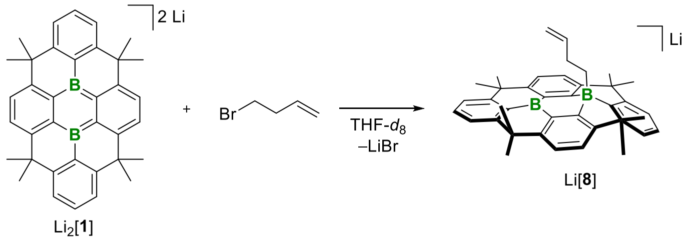

In a screw-cap vial, a sample of Li_2[**1**] (16 µmol, 1 eq.) in THF-d_8 was prepared. To the dark green solution, 4-bromo-1-butene (2.4 µL, 3.2 mg, 24 µmol, 1.5 eq.) was added. The reaction mixture decolorized after approximately 20 s. It was filtered to remove inorganic salts and transferred to a pointed flask. All volatiles were removed under reduced pressure. After re-dissolving the residue in THF-d_8 (0.4 mL), NMR spectroscopy confirmed the quantitative formation of Li[**8**] (Figures S93 – S97).

*NMR shifts and assignment of Li[**8**]*

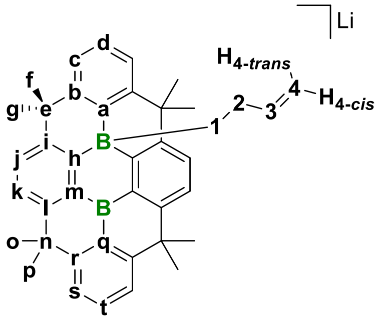

^{1}H NMR (500.2 MHz, THF-d_8): δ = 7.56 (d, $^3J_{HH}$ = 7.8 Hz, 2H; H_s), 7.51 (d, $^3J_{HH}$ = 8.2 Hz, 2H; H_j), 7.49 – 7.46 (m, 1H; H_t), 7.24 (d, $^3J_{HH}$ = 8.2 Hz, 2H; H_k), 7.23 (d, $^3J_{HH}$ = 7.7 Hz, 2H; H_c), 6.89 (t, $^3J_{HH}$ = 7.7 Hz, 1H; H_d), 5.27 – 5.19 (m, 1H; H_3), 4.25 – 4.20 (dm, $^3J_{HH}$ = 17.3 Hz, 1H; $H_{4\text{-}trans}$), 4.14 – 4.11 (dm, $^3J_{HH}$ = 10.1 Hz, 1H; $H_{4\text{-}cis}$), 1.76 (s, 6H; H_o or H_p), 1.74* (s, 6H; H_f), 1.71 (s, 6H; H_g), 1.67 (s, 6H; H_o or H_p), 1.04 – 1.00 (m, 2H; H_2), 0.2 – 0.16 (m, 2H; H_1).

*) This signal gave a cross-peak with the proton resonances of the substituent (0.2 – 0.16 ppm; H_1) in the NOESY experiment and can therefore be assigned to the two adjacent Me groups situated on the same side as the substituent. The assignment of the C*Me* signal at 1.74 ppm to H_f allows for the distinction between the upper and lower halves of the molecule, based on the ^3J-couplings to C_b and C_i observed in the ^{1}H-^{13}C-HMBC NMR spectrum.

^{7}Li NMR (194.4 MHz, THF-d_8): δ = −0.1 (s).

^{11}B NMR (160.5 MHz, THF-d_8): δ = −18.5 (s, C_4H_7−BAr_3); (n.o. = BAr_3).

^{13}C{^{1}H} NMR (125.8 MHz, THF-d_8): δ = 174.8* (vbr.; C_h), 157.8* (vbr.; C_a), 157.1 (C_r), 152.0 (C_l), 149.1 (C_b), 147.5 (C_3), 145.3 (C_i), 137.4 (br.; C_m), 134.1 (br.; C_q), 130.6 (C_t), 127.7 (C_j), 123.8 (C_s), 122.4 (C_c), 122.4 (C_d), 120.2 (C_k), 108.3 (C_4), 42.4 (C_n), 42.0 (C_e), 37.6* (vbr.; C_1), 37.5 (C_f), 35.8 (C_o or C_p), 34.4 (C_o or C_p), 33.8 (C_g), 33.2 (C_2).

*) The positions of these signals were confirmed by cross-peaks in the ^{1}H-^{13}C-HMBC NMR spectrum

1.8.7 Reaction of Li[**1**] with (bromomethyl)cyclopropane

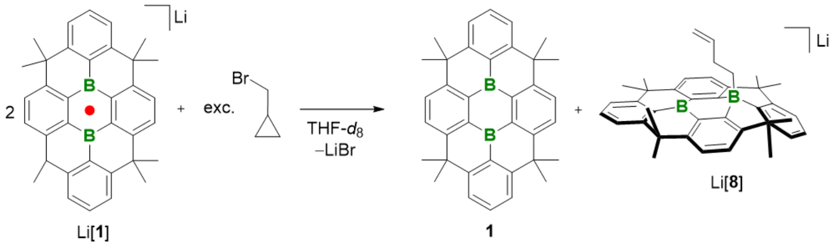

Li[**1**] (21 µmol, 2 eq.) was freshly prepared in THF-d_8 (0.4 mL). (Bromomethyl)cyclopropane (1.0 µL, 1.4 mg, 11 µmol, 1 eq.) was dissolved in THF-d_8 (0.05 mL). The two solutions were combined, whereupon the deep blue reaction mixture decolorized after 15 s. NMR spectroscopy revealed the formation of **1** and Li[**8**] in a 1:1 ratio (Figure S98).

1.8.8 Reaction of Li_2[**1**] with C_6F_6 and (bromomethyl)cyclopropane

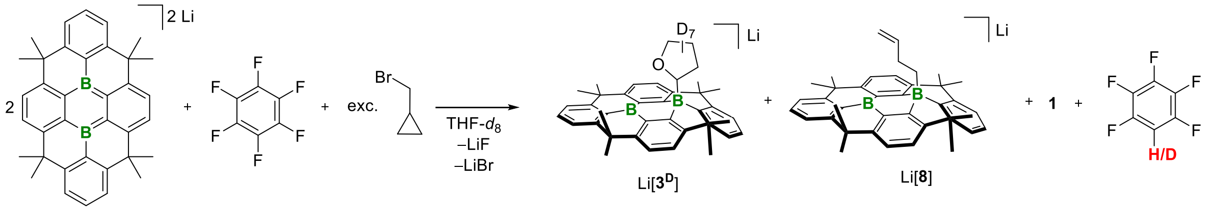

Li_2[**1**]

In a glovebox, Li_2[**1**] (18 µmol, 1 eq.) was freshly prepared in THF-d_8 (0.4 mL; screw-cap vial with a stirring bar). In two separate screw-cap vials, C_6F_6 (3.1 µL, 5.0 mg, 27 µmol, 1.5 eq.) and (bromomethyl)cyclopropane (7.0 µL, 9.7 mg, 72 µmol, 4 eq.) were each dissolved in THF-d_8 (0.05 mL). The C_6F_6 solution was added to the dark green solution of Li_2[**1**] with constant stirring, immediately followed by the addition of the (bromomethyl)cyclopropane solution. The reaction mixture decolorized within approximately 10 s and was then transferred to an NMR tube, which was subsequently flame-sealed under vacuum.

^{1}H NMR spectroscopy indicated the formation of Li[$\mathbf{3^D}$] as the main product, with Li[**8**] and **1** as side products (Figures S99, S100, S101). The formation of C_6F_5H and C_6F_5D was observed in the ^{19}F NMR spectrum. The absence of Li[**7**] suggests a radical pathway for the reaction of Li_2[**1**] with C_6F_6.

Note: When the experiment was conducted by adding a mixture of C_6F_6 and (bromomethyl)cyclopropane, the formation of Li[**7**] was also observed, likely because the reaction rates of Li_2[**1**] with either C_6F_6 or (bromomethyl)cyclopropane are sufficiently similar to compete with each other. To prevent this undesired competition, the addition of (bromomethyl)cyclopropane to the reaction mixture had to be delayed to suppress the formation of Li[**7**]. As a result, Li[**8**] is formed only in small amounts.

1.9 Reactivity of Li[**3**]

1.9.1 Reaction of Li[**3**] with HCl

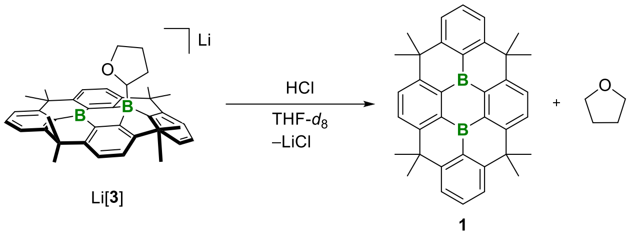

A screw-cap vial was charged with Li[**3**]·2thf (5 mg, 7 µmol, 1 eq.) in THF-d_8 (0.4 mL). Ethereal HCl (2.0 mol L^{-1}, 3.5 µL, 7.0 µmol, 1 eq.) was added with stirring at rt. The reaction mixture was transferred to an NMR-tube, which was subsequently flame-sealed under vacuum. After 1 h at rt, compound **1** had precipitated in the NMR tube, indicating completion of the reaction. NMR-spectroscopy showed the selective conversion of Li[**3**] to **1**, along with the formation of THF (Figure S102).

Note: Following the addition of HCl, compound **1** gradually precipitated from the saturated THF solution (see image below). Once the precipitation was complete, the supernatant was removed. The solid residue was dissolved in $CDCl_3$. NMR spectroscopy confirmed that the solid consisted exclusively of compound **1**.

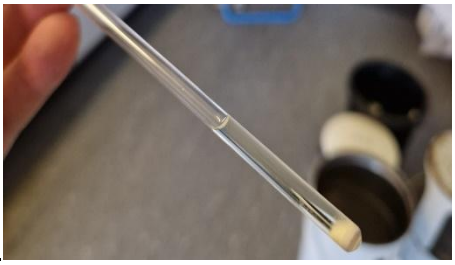
.

1.9.2 Reaction of Li[**3**$^{\mathbf{D}}$] with ambient air

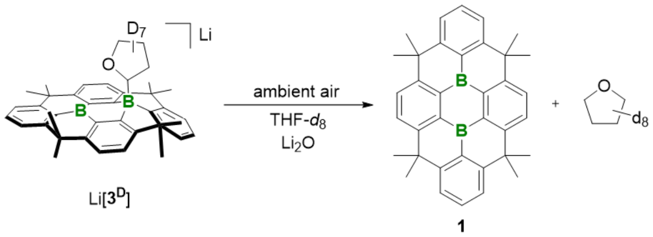

Li[**3**$^{\mathbf{D}}$] (5.6 µmol, 1 eq.) was dissolved in THF-d_8 and exposed to ambient air for 24 h. After approximately 12 h, some of compound **1** had already precipitated from the solution, indicating that the solution had reached saturation with respect to compound **1**. Monitoring the reaction mixture by ^{1}H NMR spectroscopy revealed the gradual conversion of Li[**3**$^{\mathbf{D}}$] to **1**, THF-d_8, and a minor amount of an unidentified side product (Figure S103). **1** (2.6 mg, 5.3 µmol, 95 %) was recovered using a filter column (eluent $CHCl_3$: C_6H_{12} = 1 : 6).

2 NMR spectra

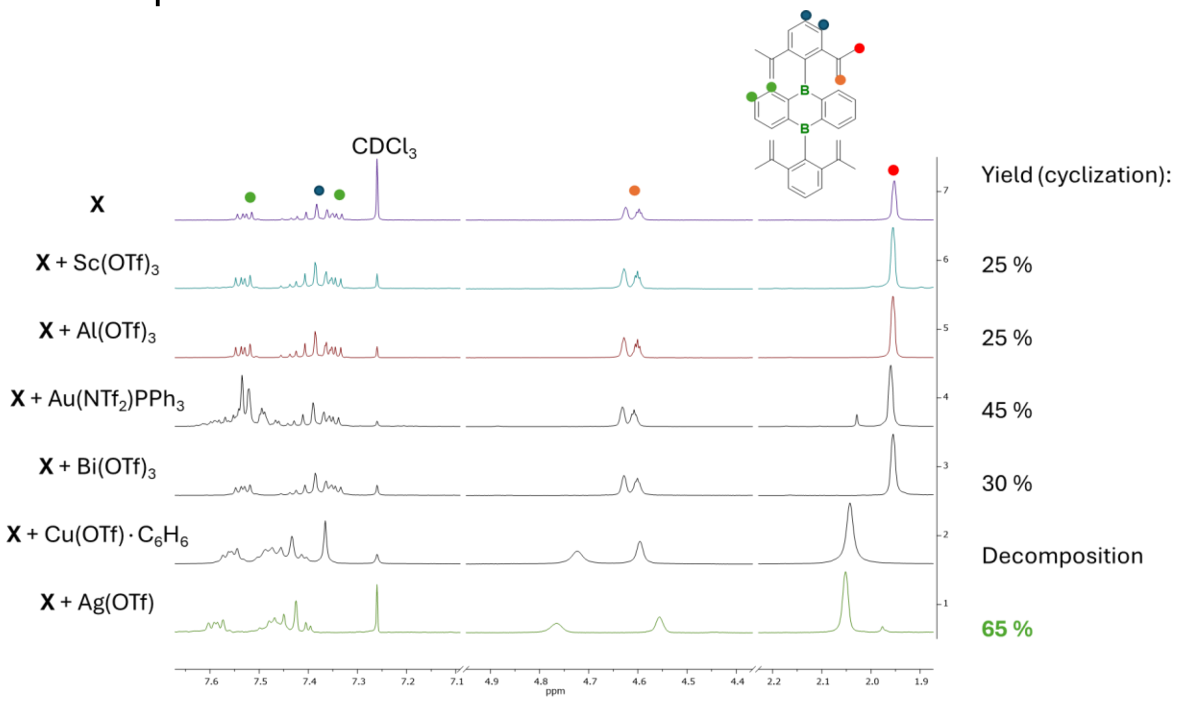

Figure S2: Sections of the ^{1}H NMR spectra (300.0 MHz, $CDCl_3$) of reaction mixtures of **C** with the respective Lewis acids.

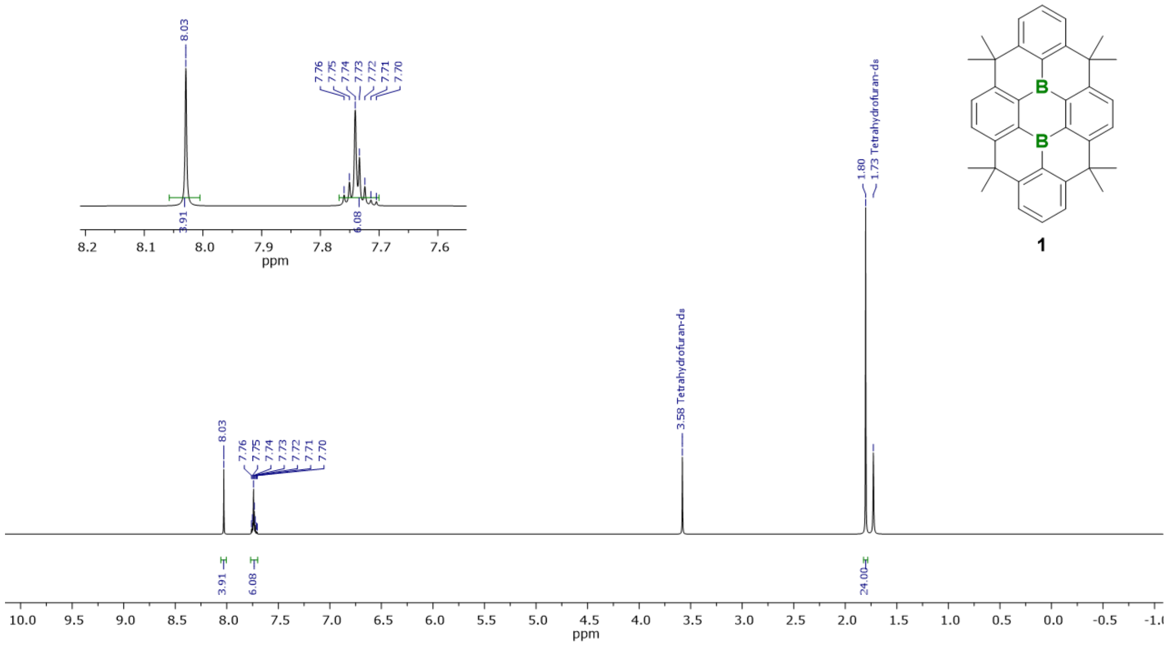

Figure S3: ^{1}H NMR spectrum (500.2 MHz, THF-*d*$_8$) of **1**.

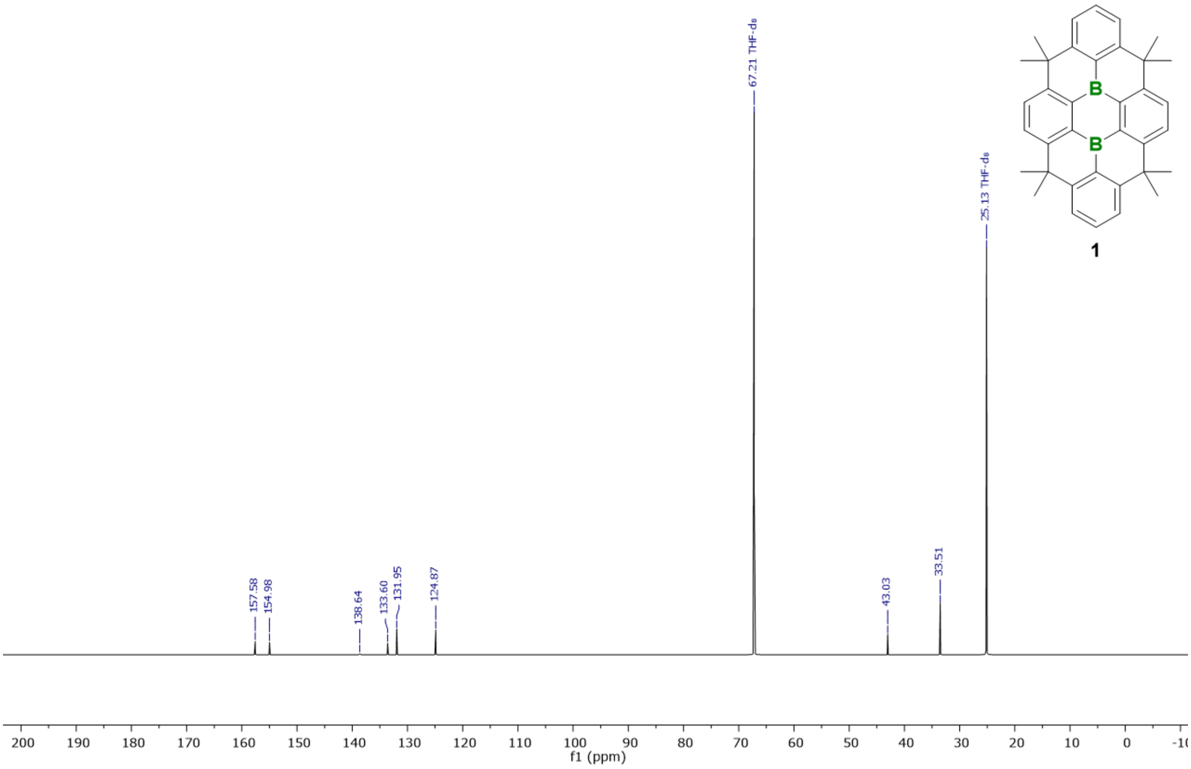

Figure S4: $^{13}C\{^{1}H\}$ NMR spectrum (125.8 MHz, THF-d_8) of **1**.

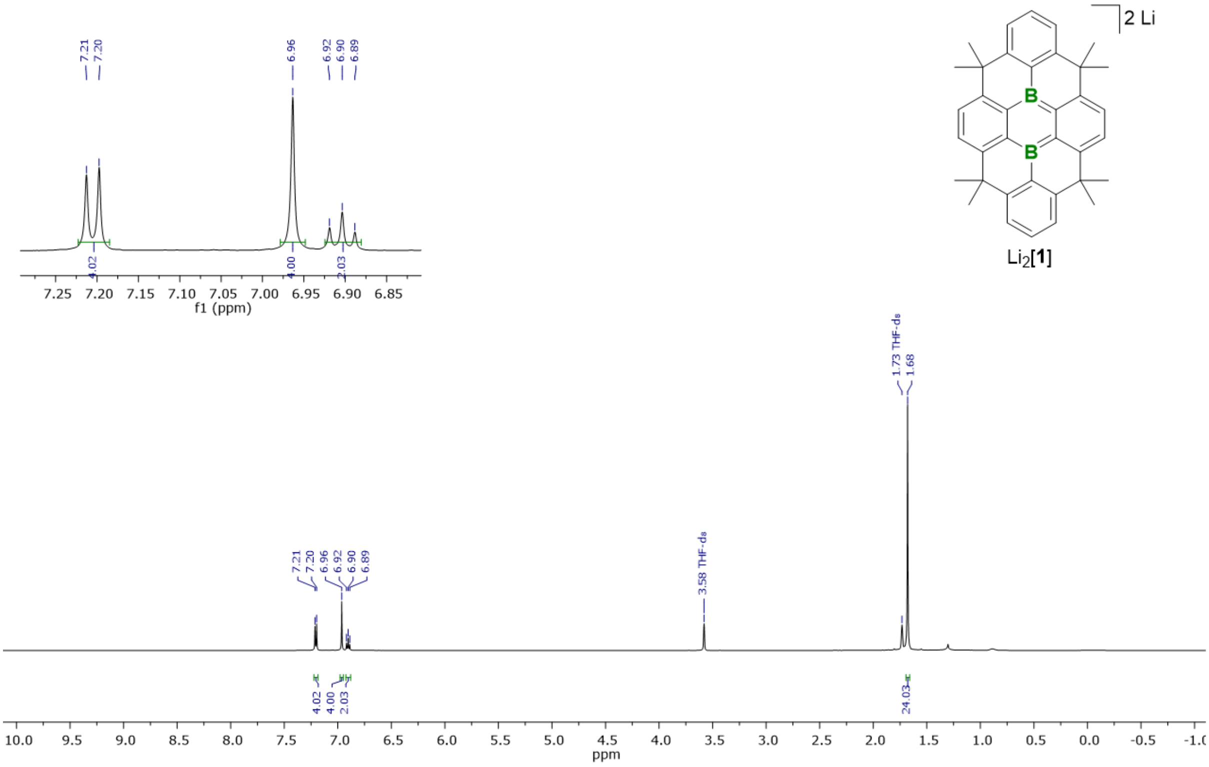

Figure S5: ^{1}H NMR spectrum (500.2 MHz, THF-d_8) of Li_2[**1**].

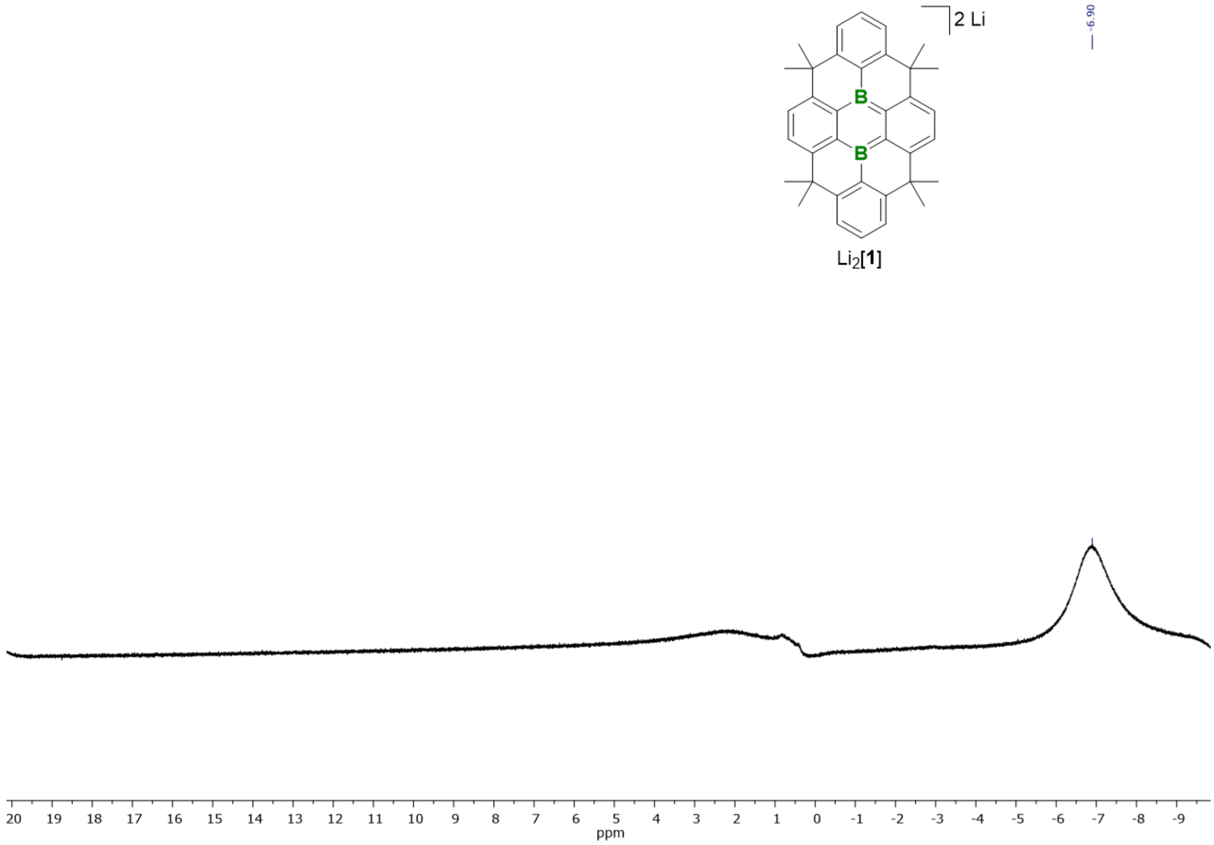

Figure S6: ^{7}Li NMR spectrum (194.4 MHz, THF-d_8) of Li_2[**1**].

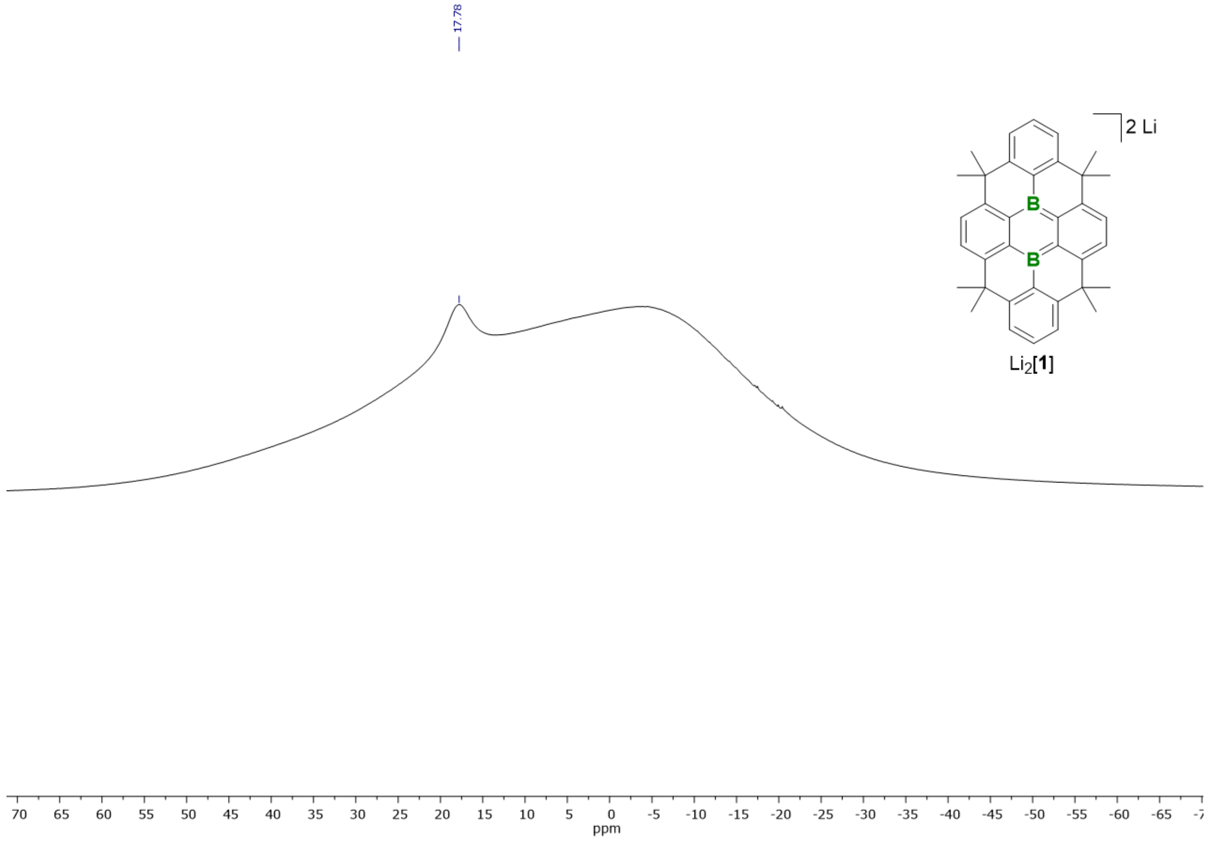

Figure S7: ^{11}B NMR spectrum (160.5 MHz, THF-d_8) of Li_2[**1**].

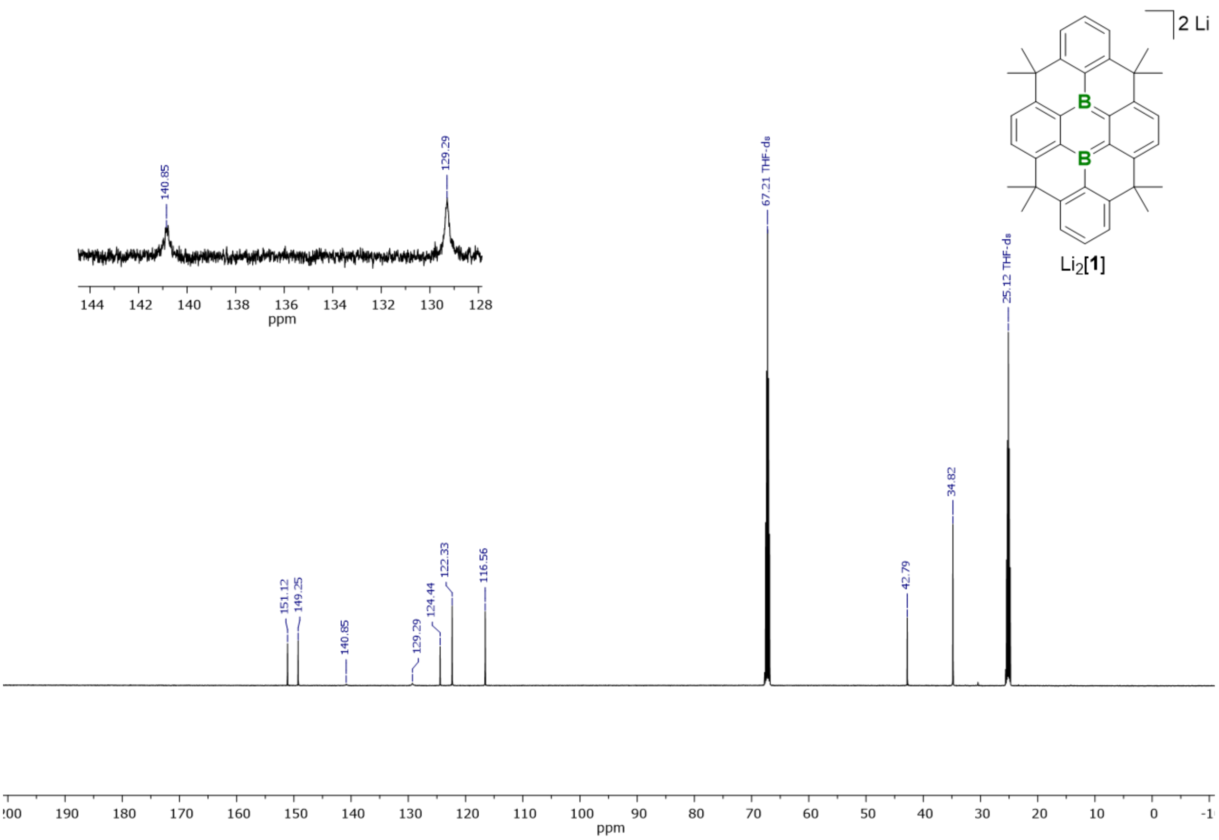

Figure S8: $^{13}C\{^1H\}$ NMR spectrum (125.8 MHz, THF-d_8) of Li_2[**1**].

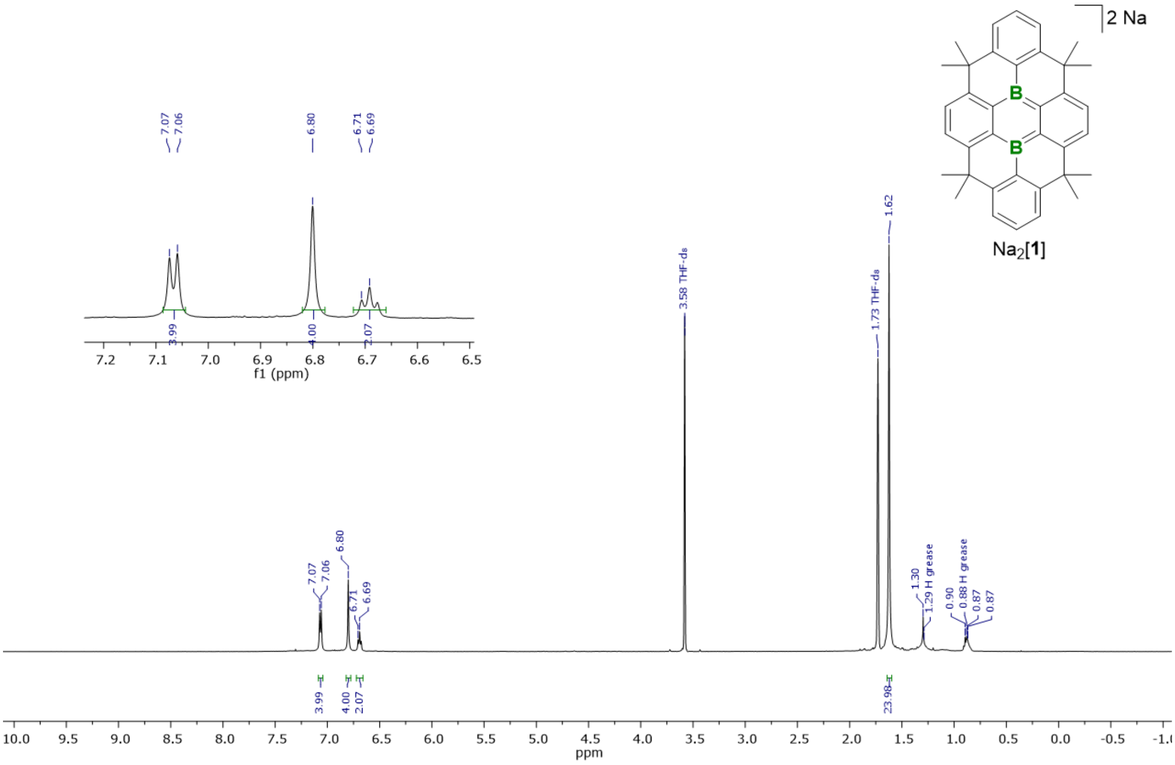

Figure S9: ^1H NMR spectrum (500.2 MHz, THF-d_8) of Na_2[**1**]. The H-grease originates from the needles used.

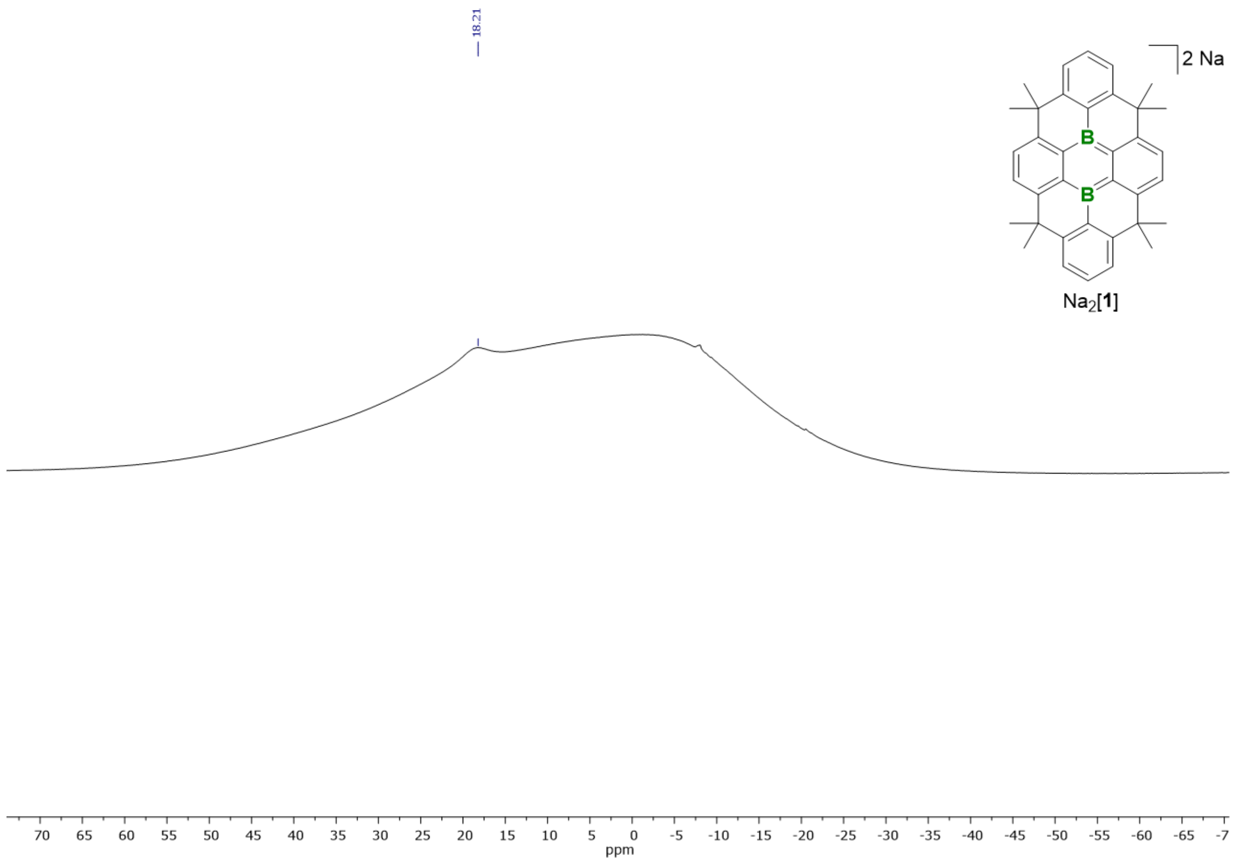

Figure S10: ^{11}B NMR spectrum (160.5 MHz, THF-d_8) of Na$_2$[**1**].

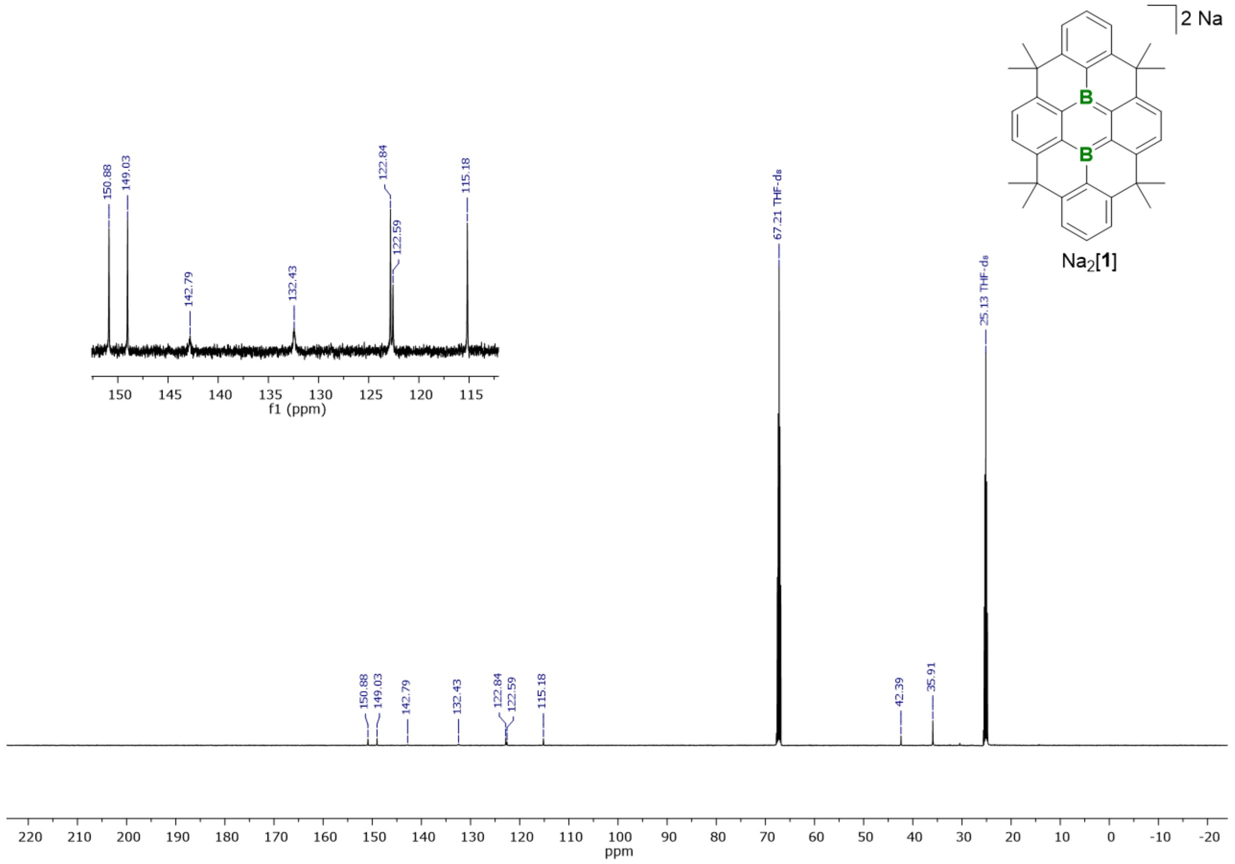

Figure S11: ^{13}C{^{1}H} NMR spectrum (125.8 MHz, THF-d_8) of Na$_2$[**1**]

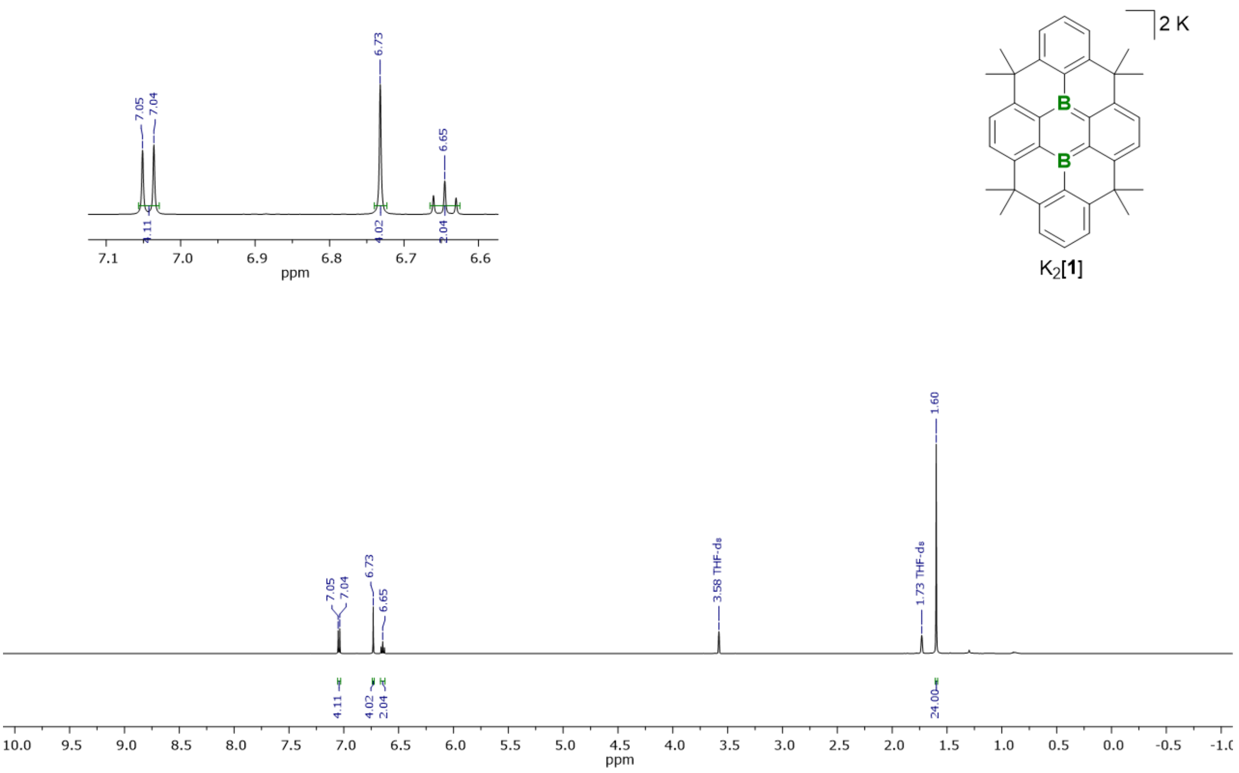

Figure S12: ^{1}H NMR spectrum (500.2 MHz, THF-d_8) of $K_2[\mathbf{1}]$.

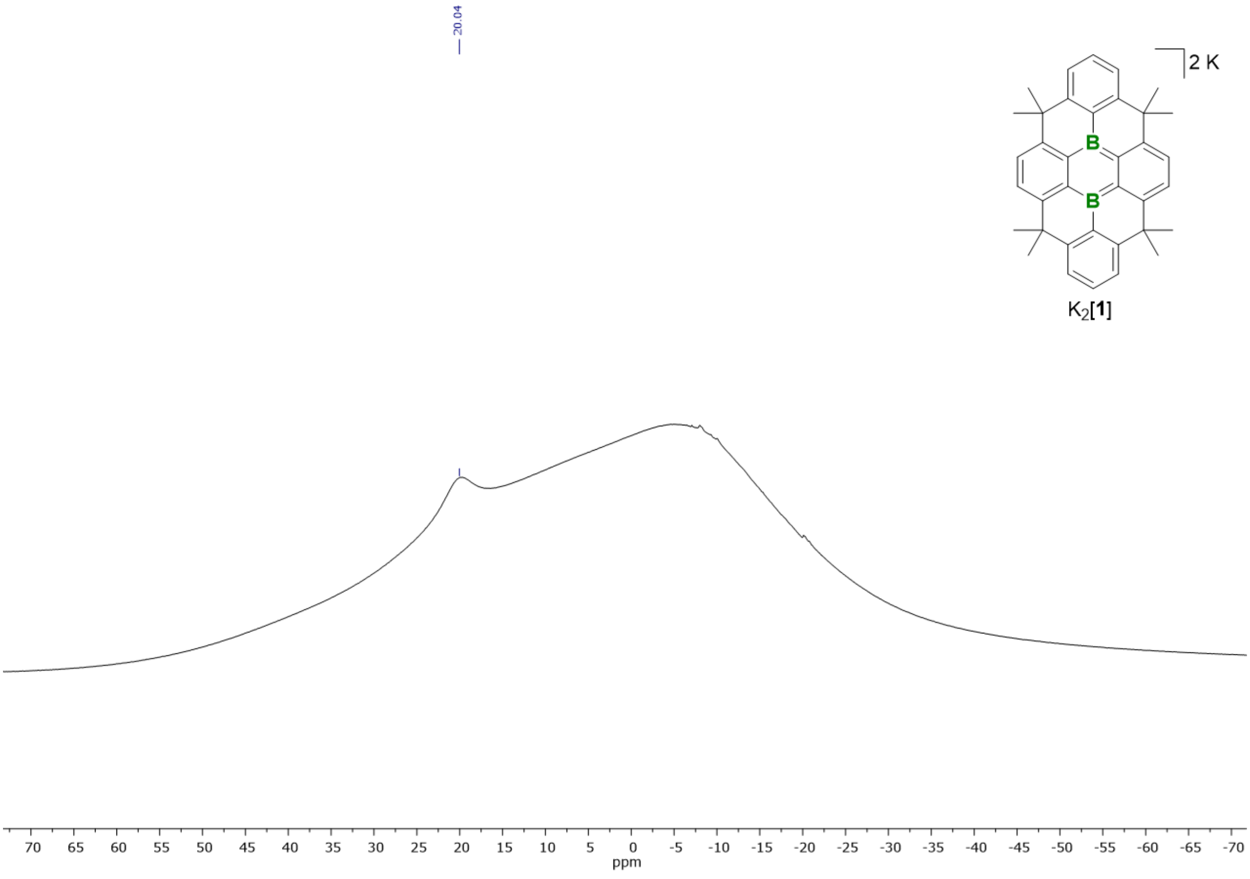

Figure S13: ^{11}B NMR spectrum (160.5 MHz, THF-d_8) of $K_2[\mathbf{1}]$.

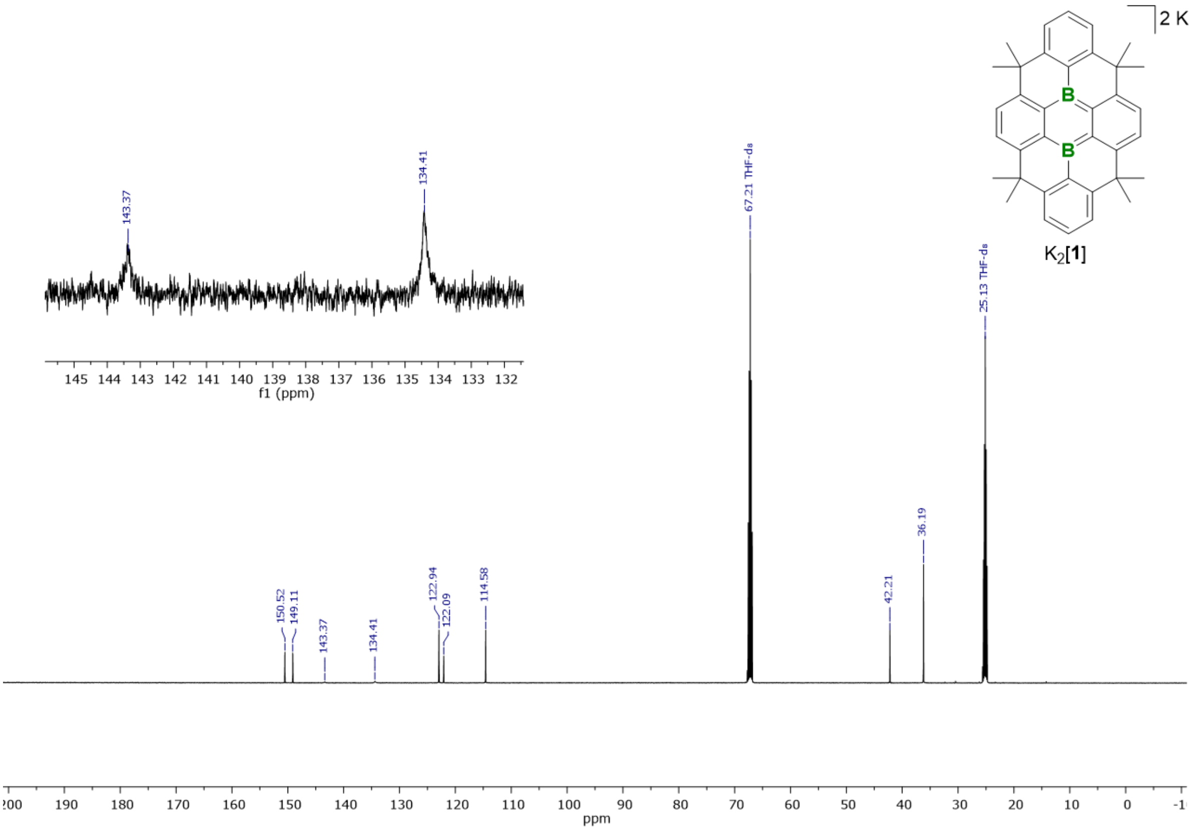

Figure S14: $^{13}C\{^{1}H\}$ NMR spectrum (125.8 MHz, THF-d_8) of K_2[**1**].

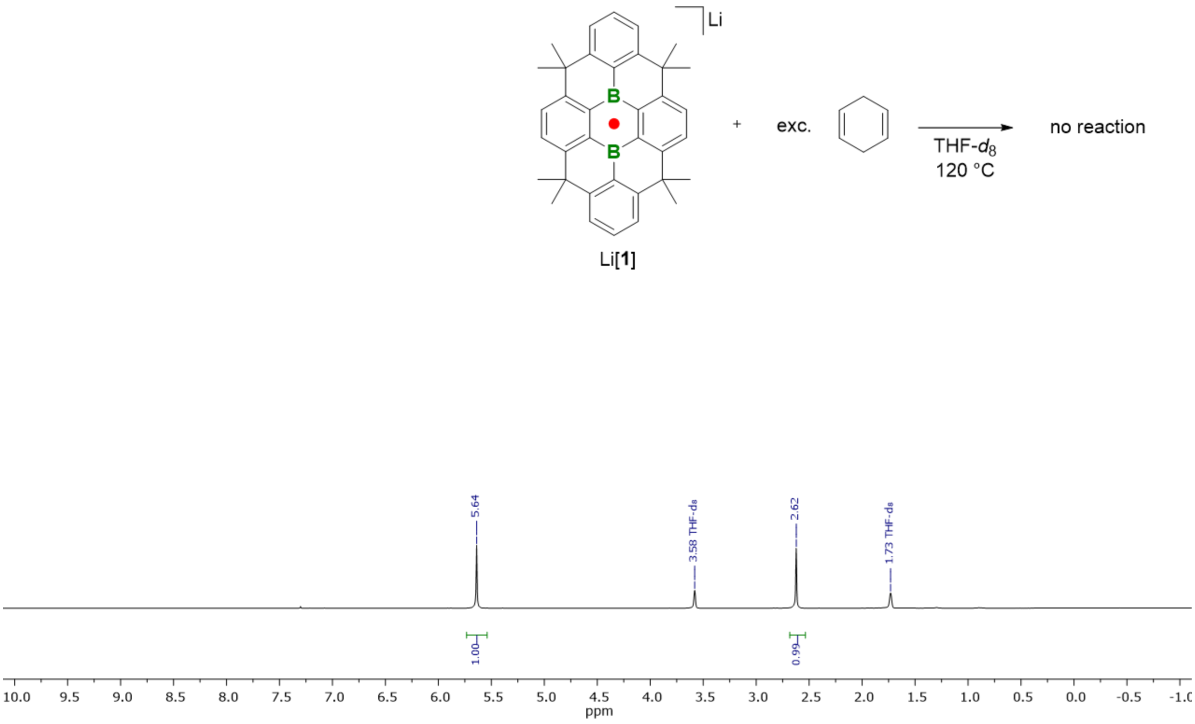

Figure S15: ^{1}H NMR spectrum (300.0 MHz, THF-d_8) of the reaction mixture containing Li[**1**] and an excess of 1,4-cyclohexadiene. No reaction could be observed after heating to 120 °C.

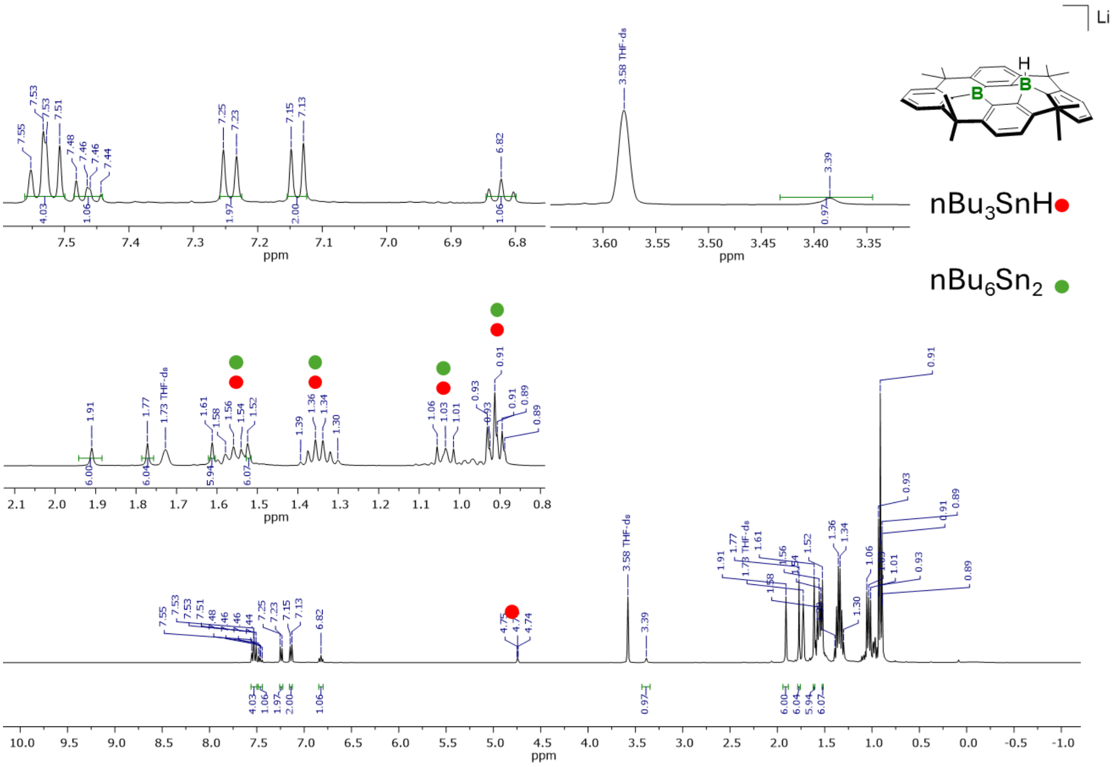

Figure S16: The $^{1}H\{^{11}B\}$ NMR spectrum (300.0 MHz, THF-*d*8) of the reaction mixture of Li[**1**] with *n*Bu3SnH after heating to 50 °C overnight shows the predominant formation of Li[**1**•H].

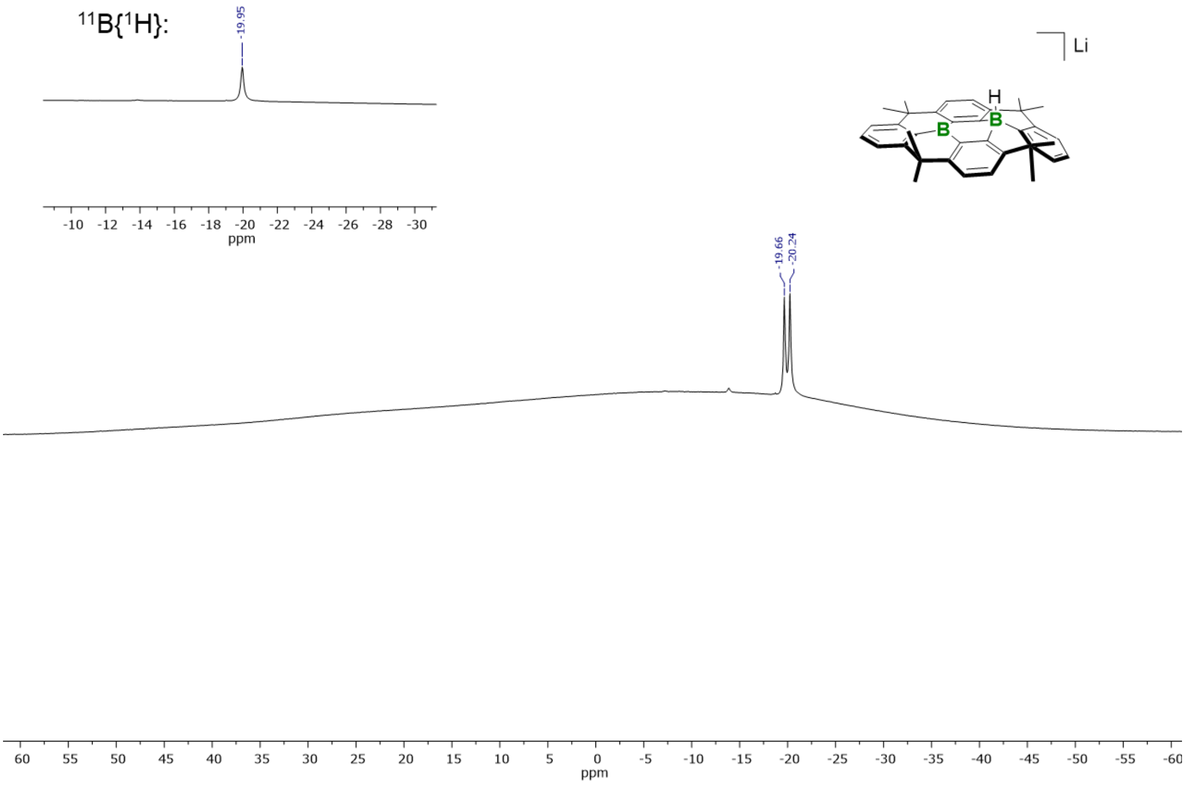

Figure S17: ^{11}B NMR spectrum (96.3 MHz, THF-*d*8) of the reaction mixture of Li[**1**] with *n*Bu3SnH after heating to 50 °C overnight. The enlarged region displays the corresponding $^{11}B\{^{1}H\}$ NMR spectrum, in which the observed doublet collapses to a singlet.

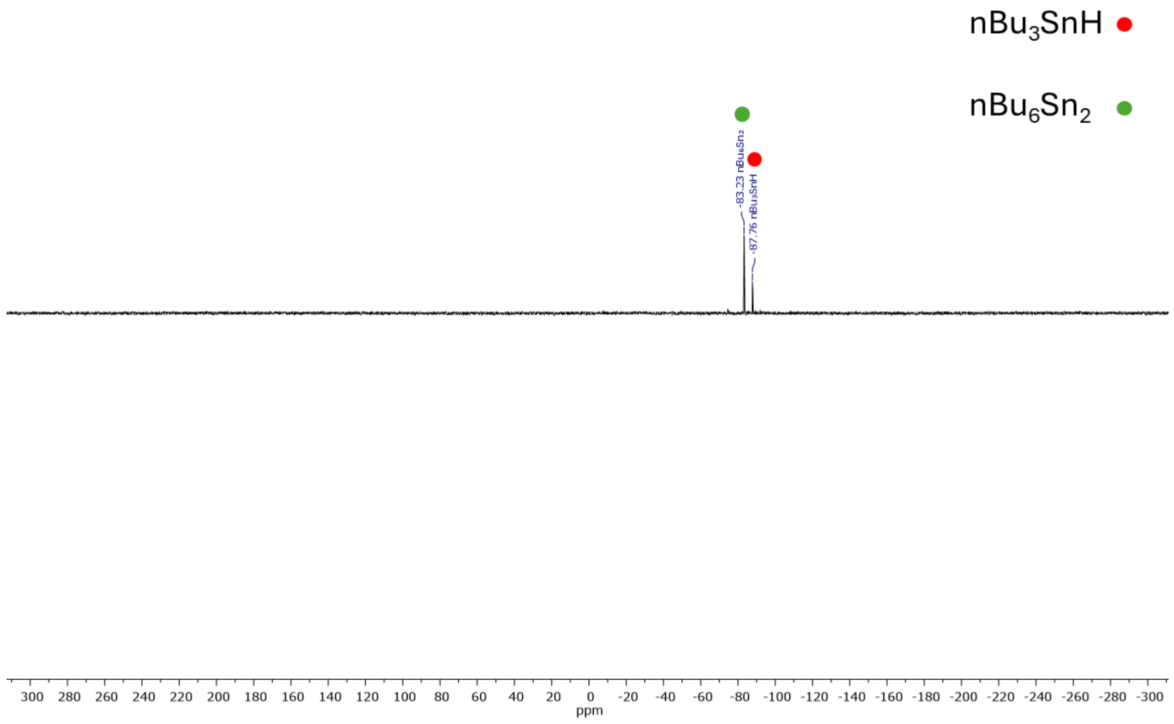

Figure S18: $^{119}Sn\{^1H\}$ NMR spectrum (149.3 MHz, THF-d_8) of the reaction mixture of Li[**1**] with nBu$_3$SnH after heating to 50 °C overnight.

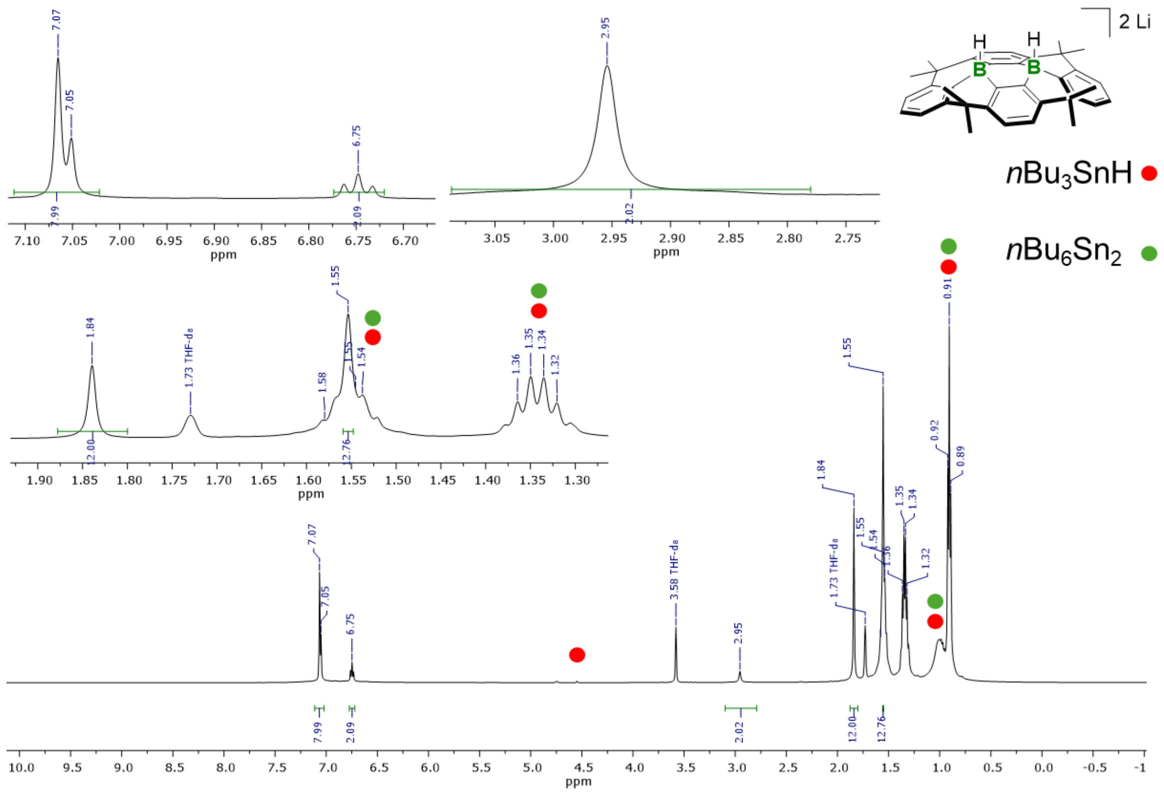

Figure S19: $^1H\{^{11}B\}$ NMR spectrum (300.0 MHz, THF-d_8) of Li$_2$[**1**•2H]. Residual nBu$_3$SnH and nBu$_6$Sn$_2$ could not be removed by crystallization; the spectra were therefore recorded on the crude reaction mixture.

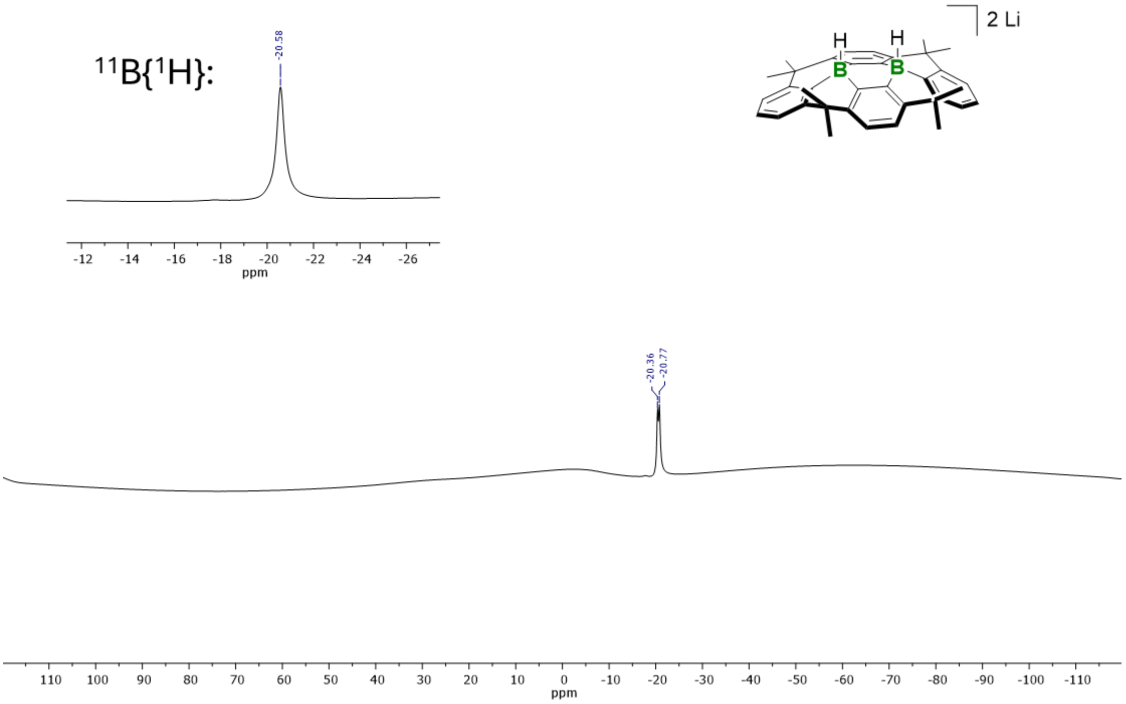

Figure S20: ^{11}B NMR spectrum (96.3 MHz, THF-d_8) of Li$_2$[**1**•2H]. The enlarged region displays the corresponding ^{11}B{^{1}H} NMR spectrum, in which the observed doublet collapses to a singlet.

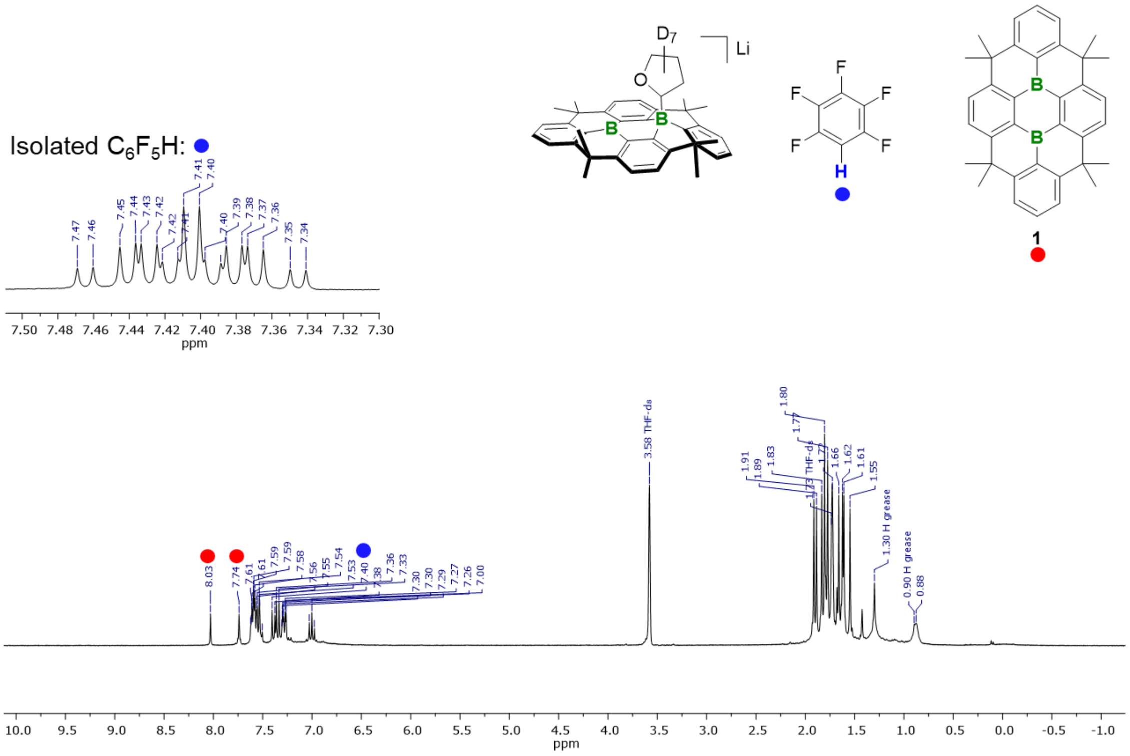

Figure S21: ^{1}H NMR spectrum (300.0 MHz, THF-d_8) of the reaction mixture of Li$_2$[**1**] with C_6F_6 after heating the reaction mixture to 50 °C for 12 h shows the formation of Li[**3^D**], compound **1** (red dots) and C_6F_5H (blue dots). The expanded region from 7.50 – 7.30 ppm shows the proton resonance of isolated C_6F_5H, which would otherwise be obscured by overlapping signals with Li[**3^D**] in the full spectrum.

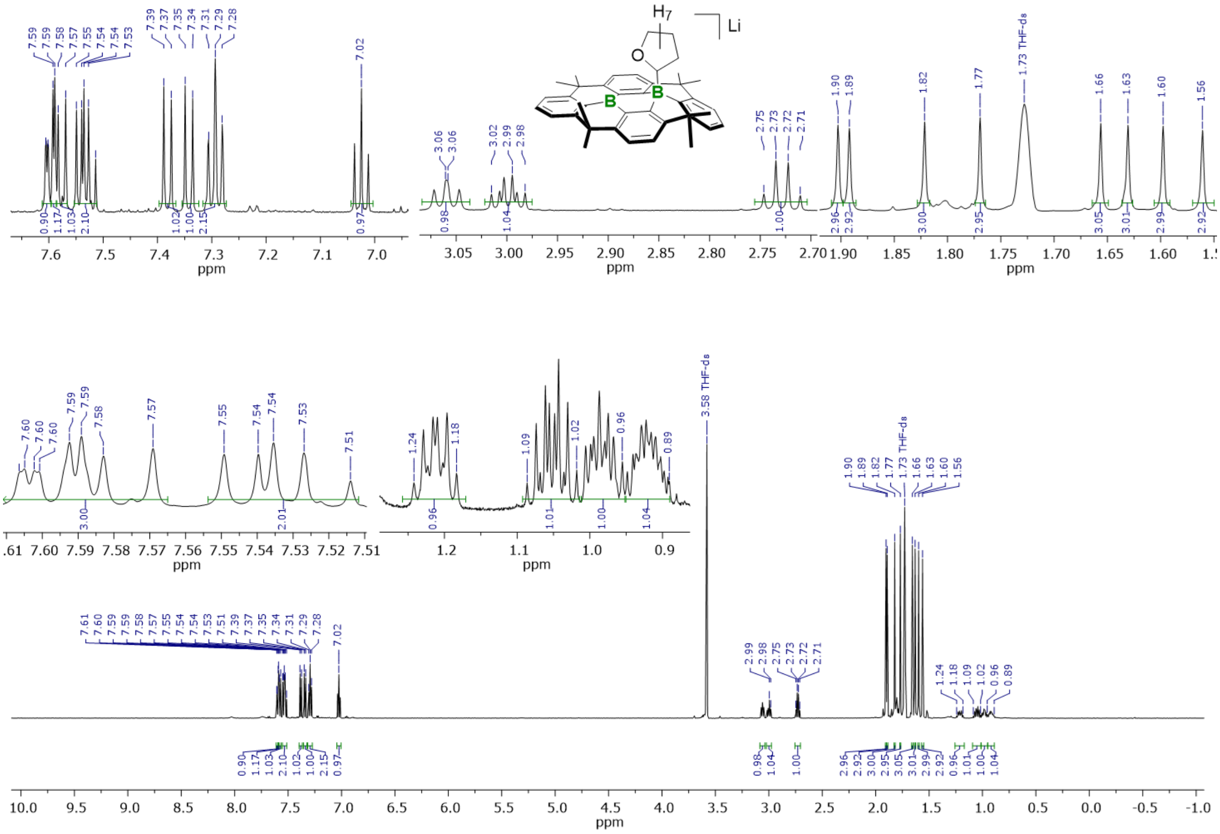

Figure S22: ^{1}H NMR spectrum (500.2 MHz, THF-d_8) of Li[**3**]. The expanded region from 7.61 to 7.51 ppm was recorded on an NMR spectrometer operating at a frequency of 600.0 MHz.

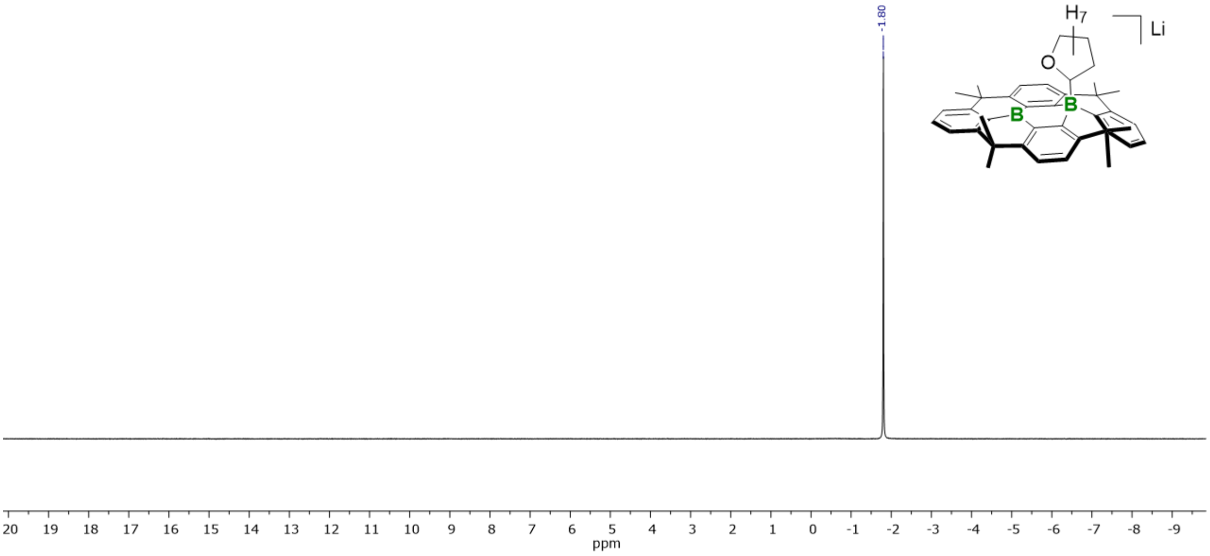

Figure S23: ^{7}Li NMR spectrum (194.4 MHz, THF-d_8) of Li[**3**].

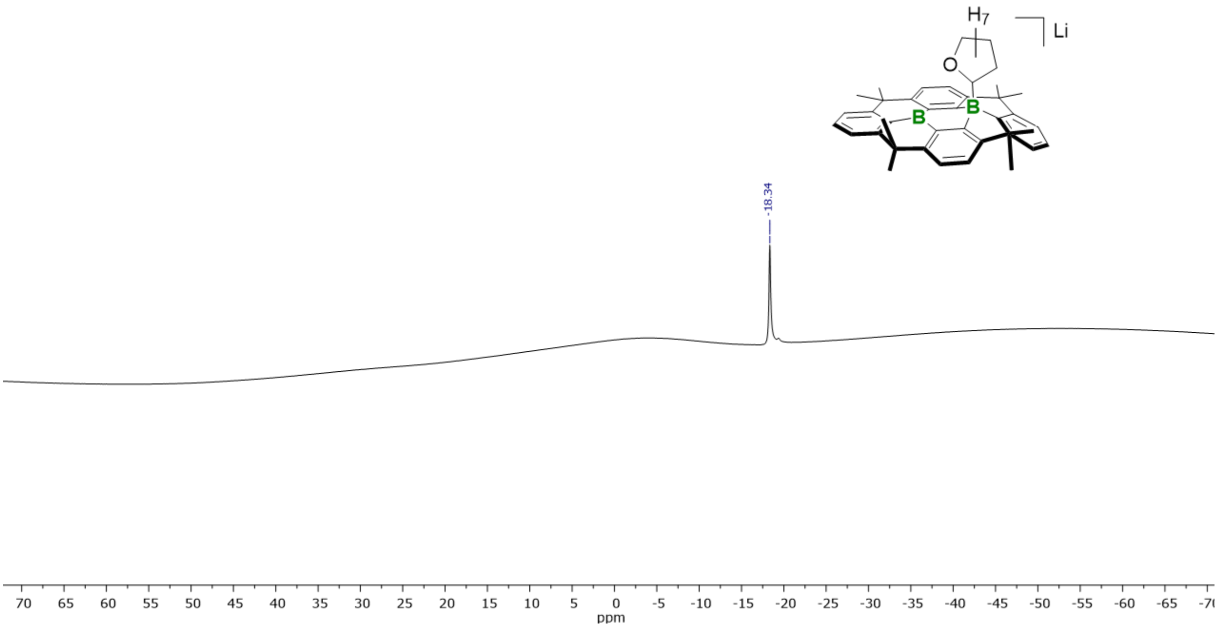

Figure S24: ^{11}B NMR spectrum (160.5 MHz, THF-d_8) of Li[**3**].

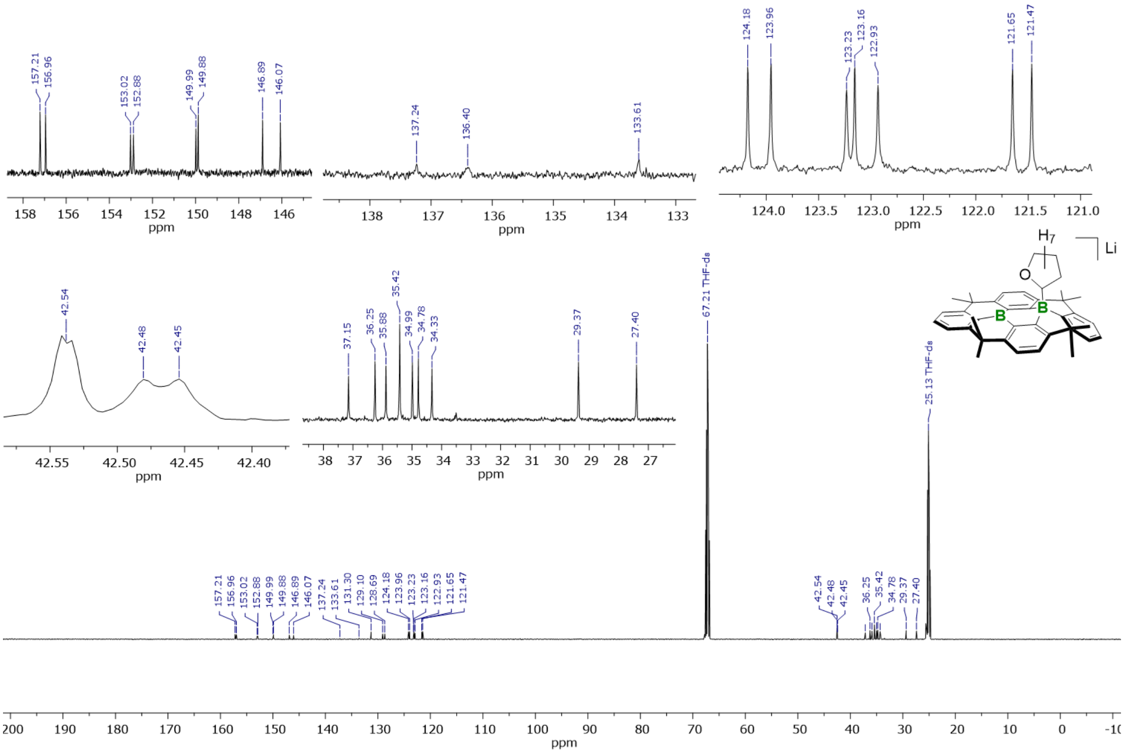

Figure S25: ^{13}C{^{1}H} NMR spectrum (125.8 MHz, THF-d_8) of Li[**3**].

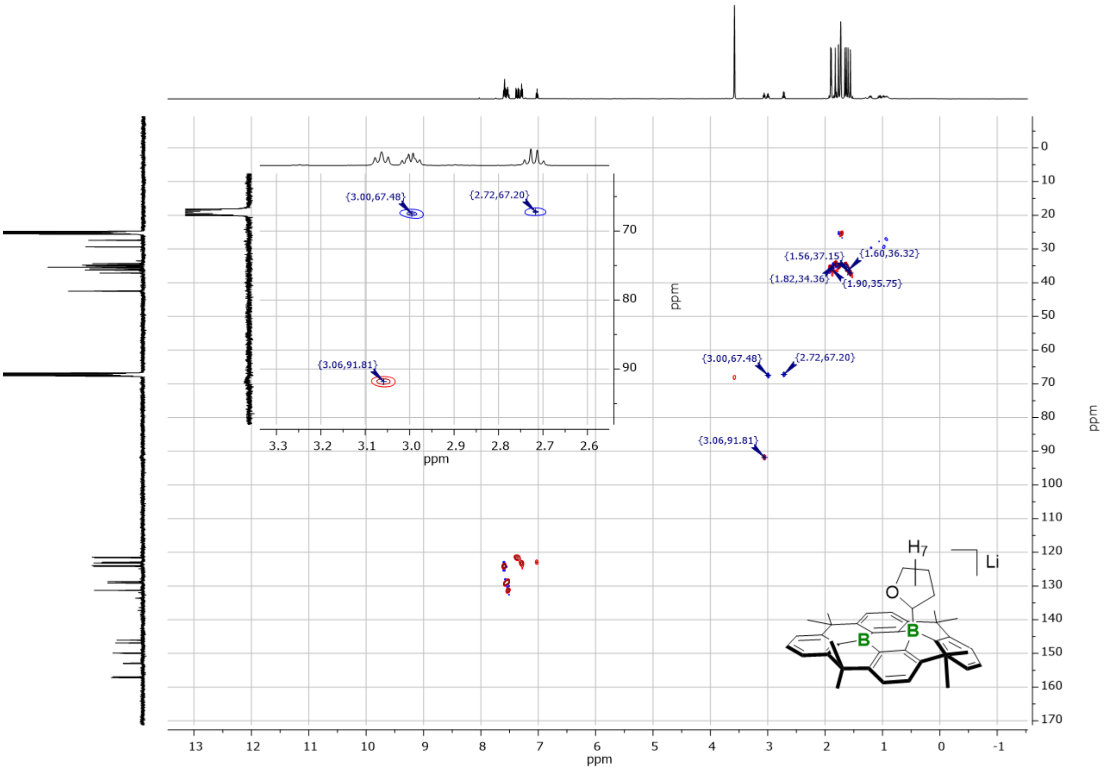

Figure S26: ^{1}H-^{13}C HSQC NMR spectrum (THF-d_8) of Li[**3**].

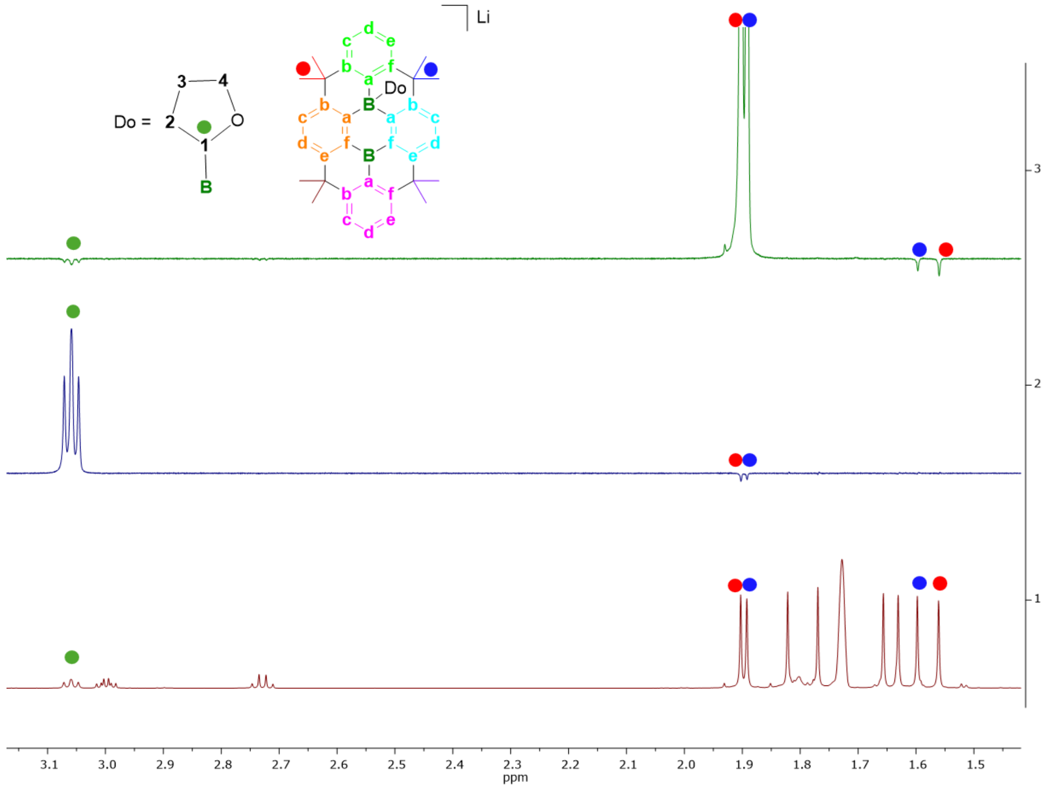

Figure S27: Expanded regions of the ^{1}H NMR and ^{1}H-^{1}H NOESY NMR spectra (500.2 MHz, THF-d_8) of Li[**3**]. Bottom: ^{1}H NMR spectrum (500.2 MHz, THF-d_8) of Li[**3**]. Middle: Selective excitation of H_1 (green resonance) of the C_4H_7O substituent shows that the signals at 1.90 (red) and 1.89 ppm (blue) can be assigned to the C*Me* groups positioned on the same side as the substituent. Top: Selective excitation of the C*Me* groups resonating at 1.90 (red) and 1.89 ppm (blue) shows interactions with the corresponding C*Me* groups at 1.60 (blue) and 1.56 ppm (red), where each red- and blue-marked pair is bonded to the same C atom. Additionally, interactions with H_1

of the C_4H_7O substituent are observed. *Note*: The assignment of blue- and red-marked C*Me*$_2$ pairs is based on the identical ^3J-couplings to aromatic quaternary C atoms observed in the ^{1}H-^{13}C-HMBC NMR spectrum.

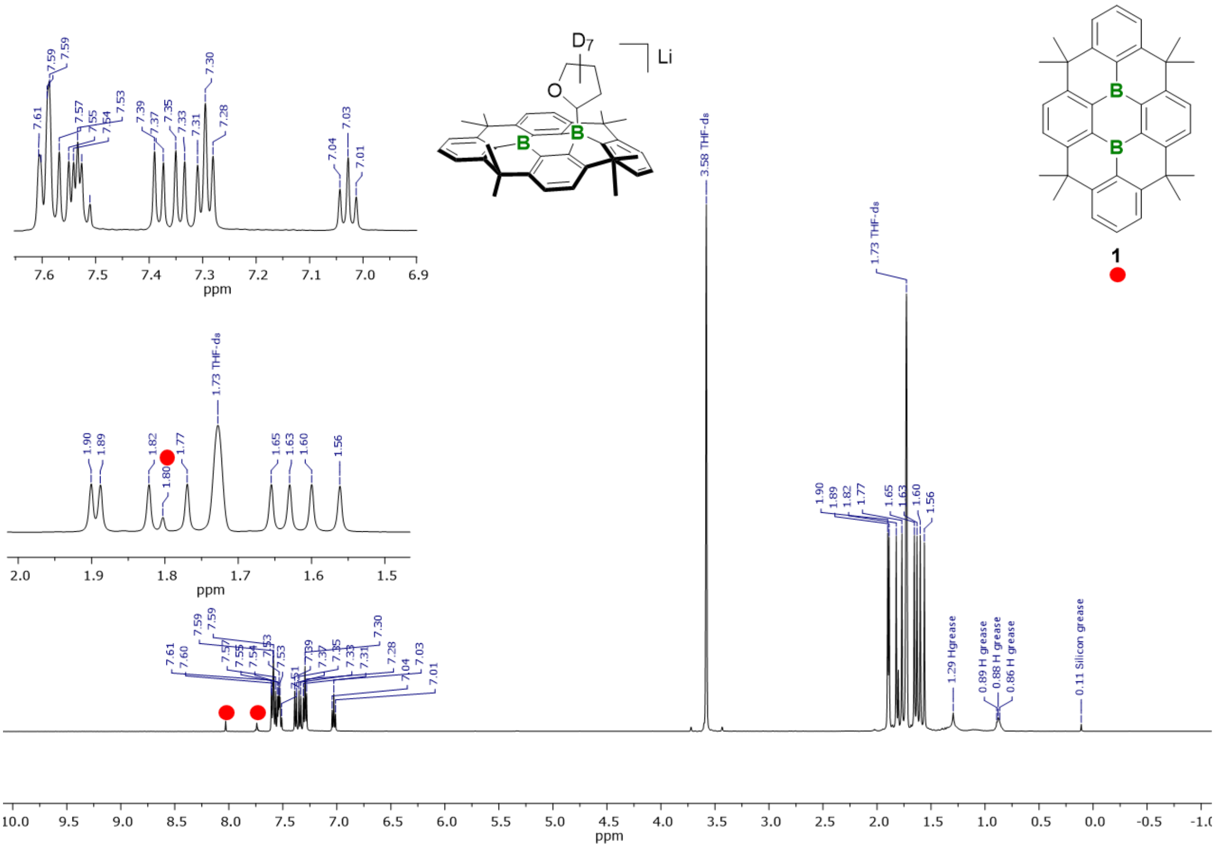

Figure S28: ^{1}H NMR spectrum (500.2 MHz, THF-d_8) of Li[**3**$^{\mathbf{D}}$] (with minor contaminations from compound **1** (red)).

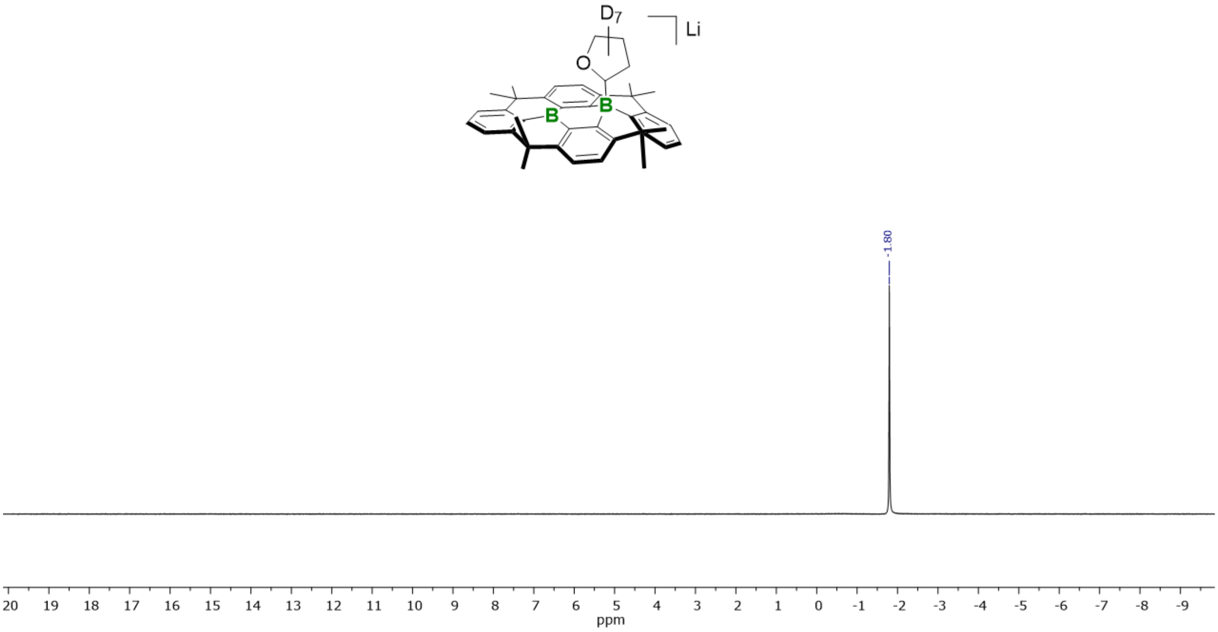

Figure S29: ^{7}Li NMR spectrum (194.4 MHz, THF-d_8) of Li[**3**$^{\mathbf{D}}$].

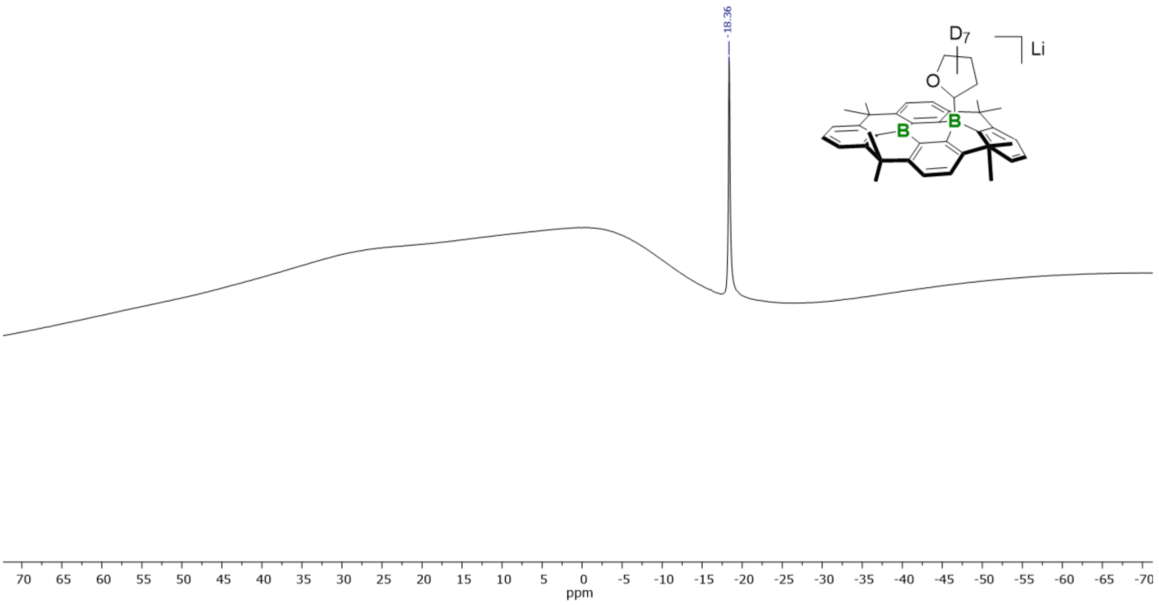

Figure S30: ^{11}B NMR spectrum (160.5 MHz, THF-d_8) of Li[**3^D**].

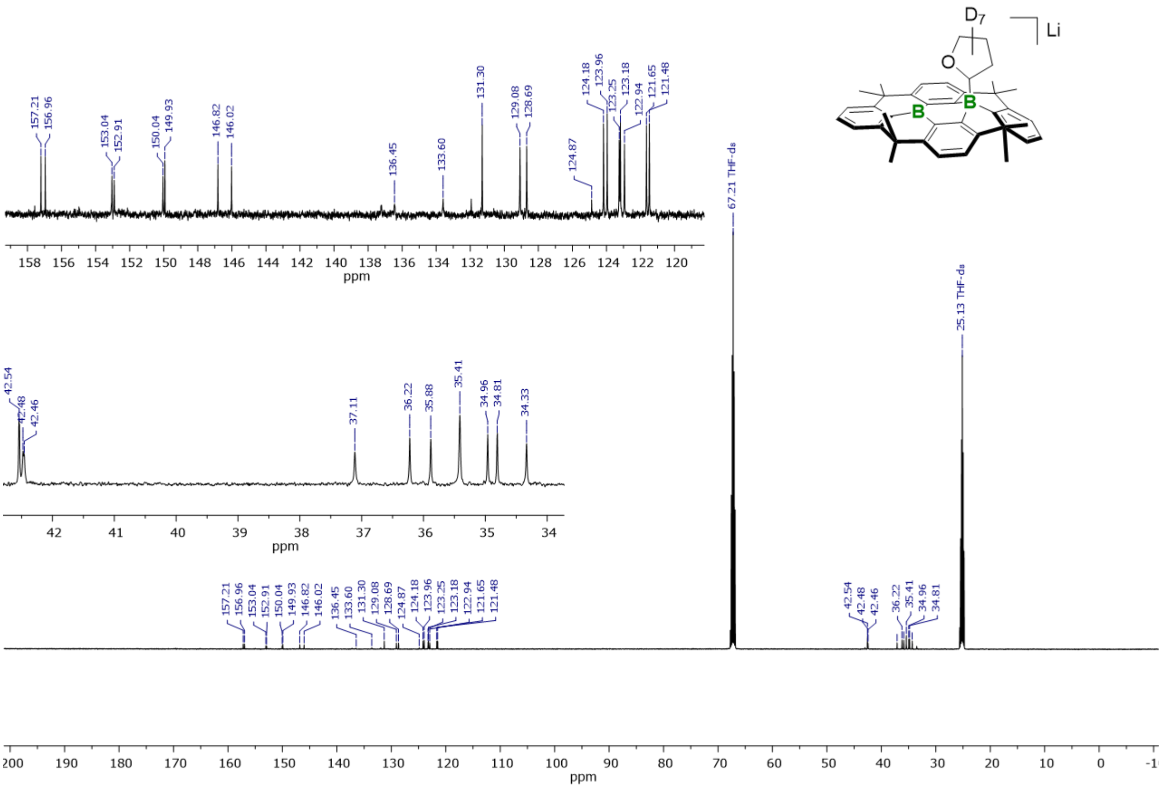

Figure S31: ^{13}C{^{1}H} NMR spectrum (125.8 MHz, THF-d_8) of Li[**3^D**].

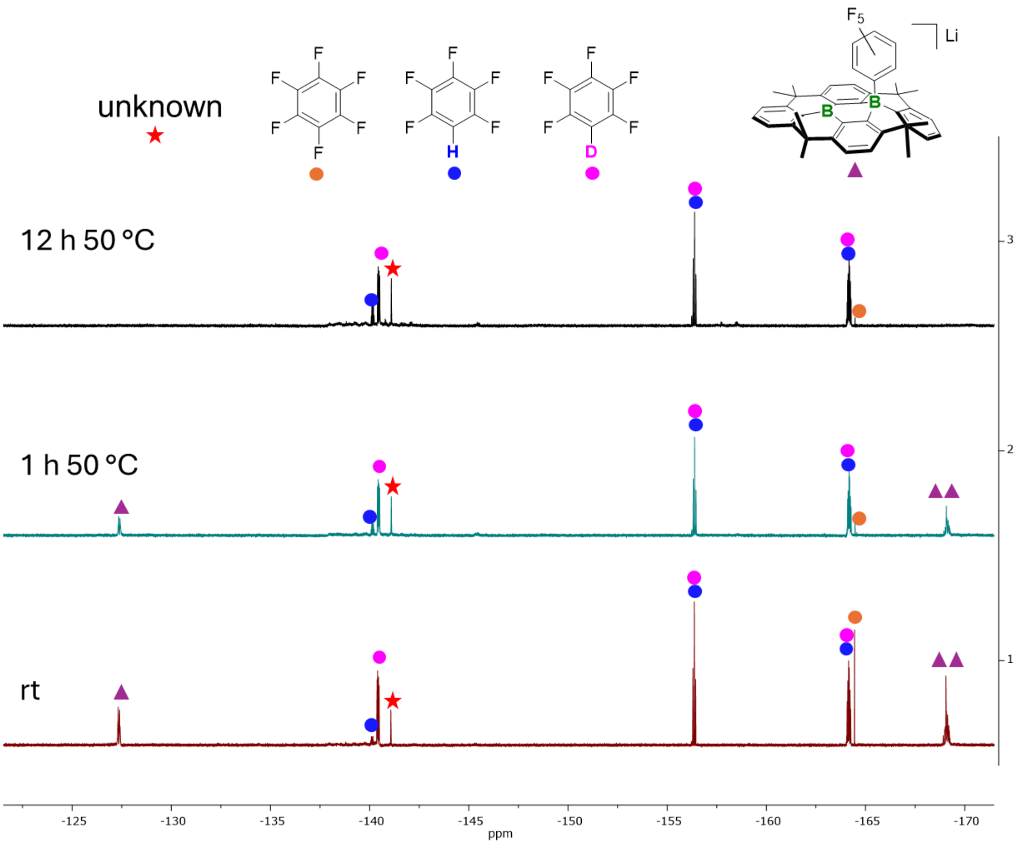

Figure S32: The ^{19}F NMR spectra (282.3 MHz, THF-d_8) of the reaction mixture of Li_2[**1**] and C_6F_6 at various temperatures indicate that the side product Li[**4**] (purple triangle) is thermolabile, resulting in the formation of C_6F_5H (blue) or C_6F_5D (magenta). Bottom: Reaction mixture at rt. Middle: Reaction mixture after heating to 50 °C for 1h. Top: Reaction mixture after heating to 50 °C for 12 h.

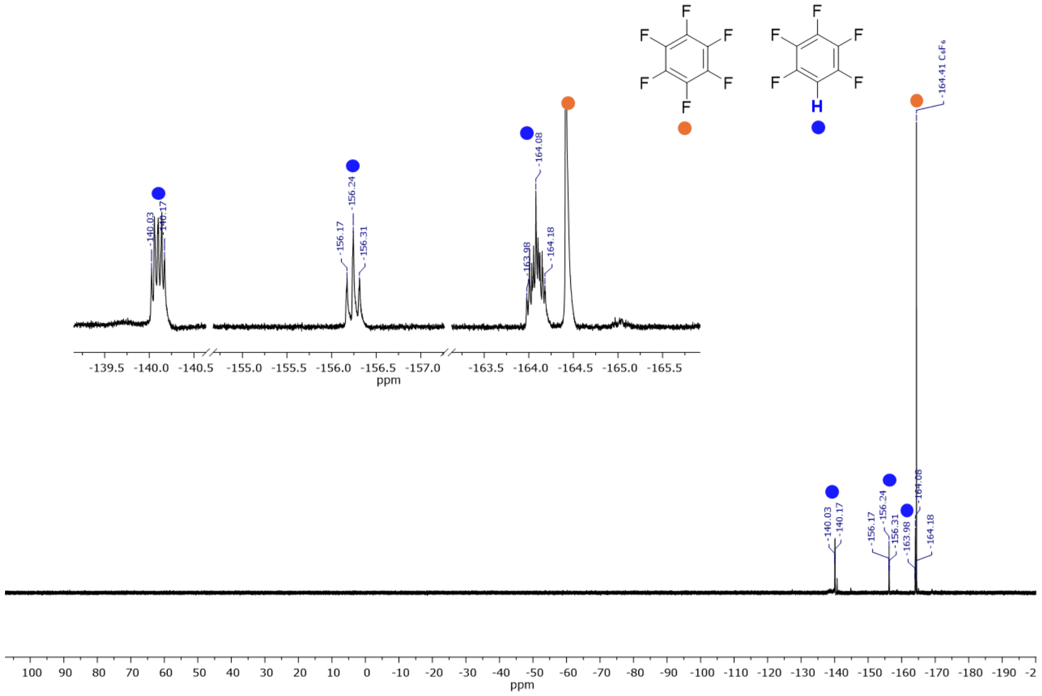

Figure S33: ^{19}F NMR spectrum (282.3 MHz, THF) of C_6F_5H, isolated after heating the reaction mixture of Li_2[**1**] and C_6F_6 to 50 °C. A small amount of C_6F_6 (orange dots) remains, due to the slight excess used in the reaction.

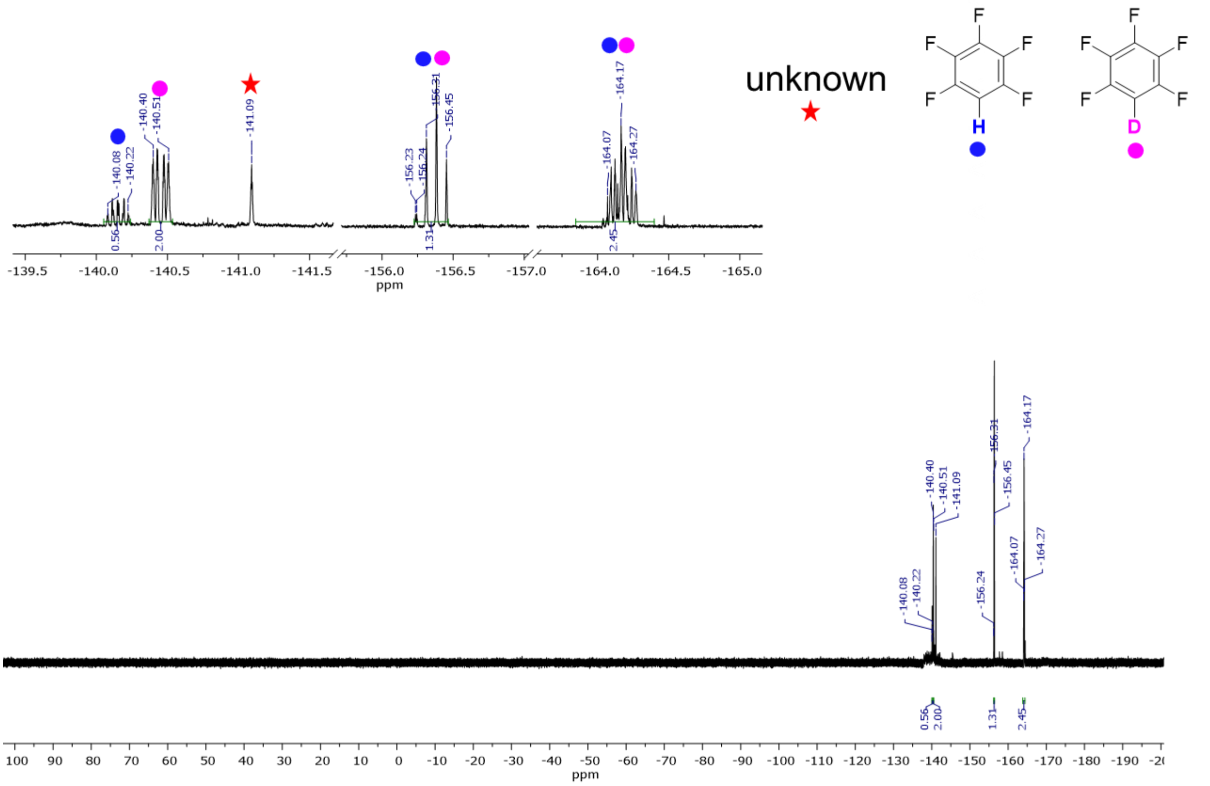

Figure S34: ^{19}F NMR spectrum (282.3 MHz, THF-d_8) of the reaction mixture of Li_2[**1**] and C_6F_6 after heating the reaction mixture to 50 °C. The spectrum shows the formation of a product mixture consisting of C_6F_5D and C_6F_5H. The singlet at −141.1 ppm (red star) corresponds to an as-yet unidentified compound.

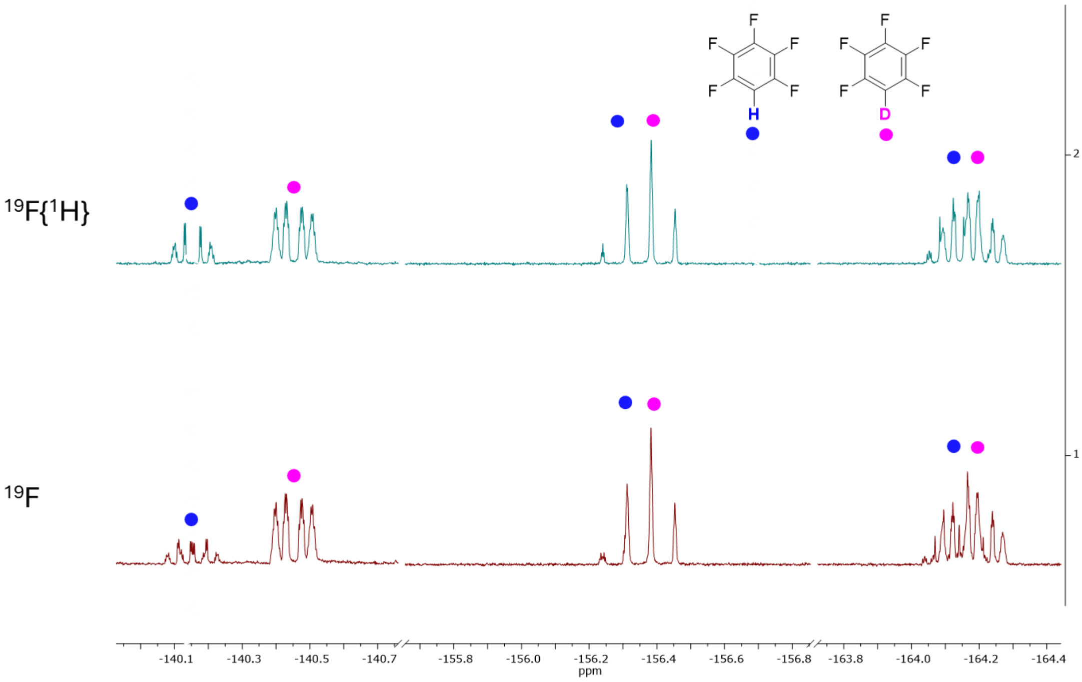

Figure S35: Expanded regions of ^{19}F (bottom) and ^{19}F{^{1}H} (top) NMR spectra (282.3 MHz, THF-d_8) of the mixture of C_6F_5D (pink dots) and C_6F_5H (blue dots) obtained after heating the reaction mixture to 50 °C. The ^{19}F{^{1}H} spectrum allows the assignment of the *o*-F signals: for C_6F_5H, the signal collapses into a simpler multiplet; for C_6F_5D, the signal remains unchanged compared to the ^{19}F NMR spectrum.

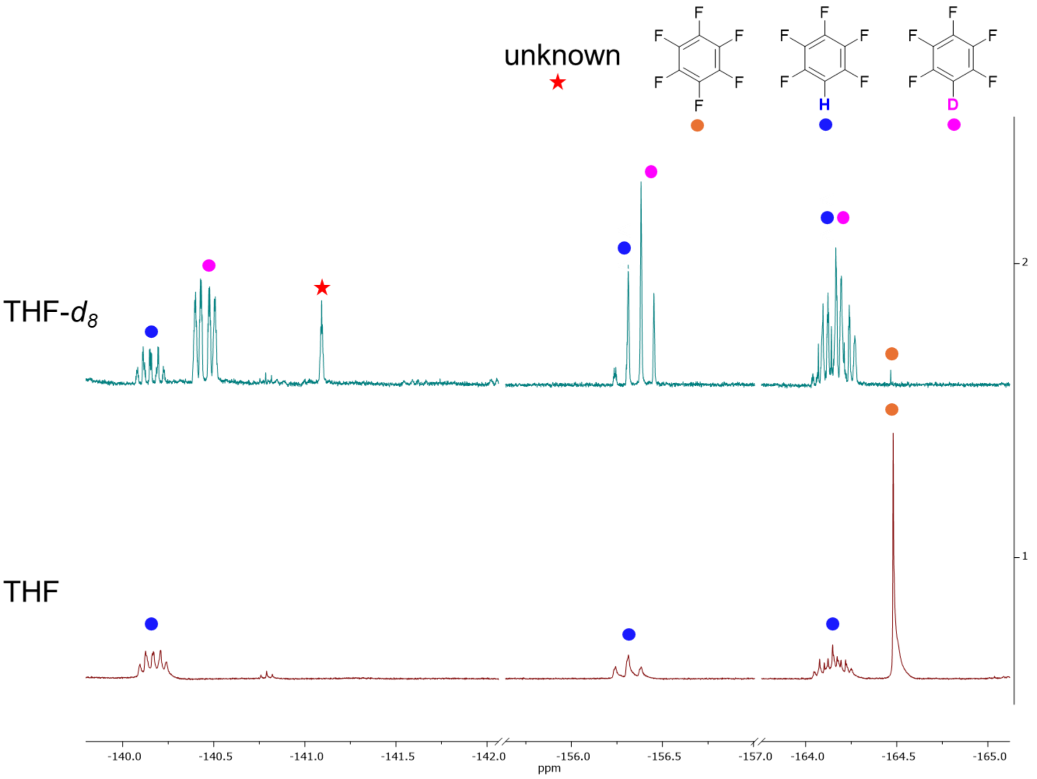

Figure S36: Expanded regions of ^{19}F NMR spectra (282.3 MHz, THF-d_8 or THF) of the reaction mixture of Li_2[**1**] and C_6F_6 in THF (bottom) or THF-d_8 (top). Conducting the reaction in THF, where no source of D atoms is present, results in the absence of any C_6F_5D signals. This allows the assignment of signals to C_6F_5H (blue dots) and C_6F_5D (pink dots). The singlet at −141.1 ppm (red star) corresponds to an as-yet unidentified compound.

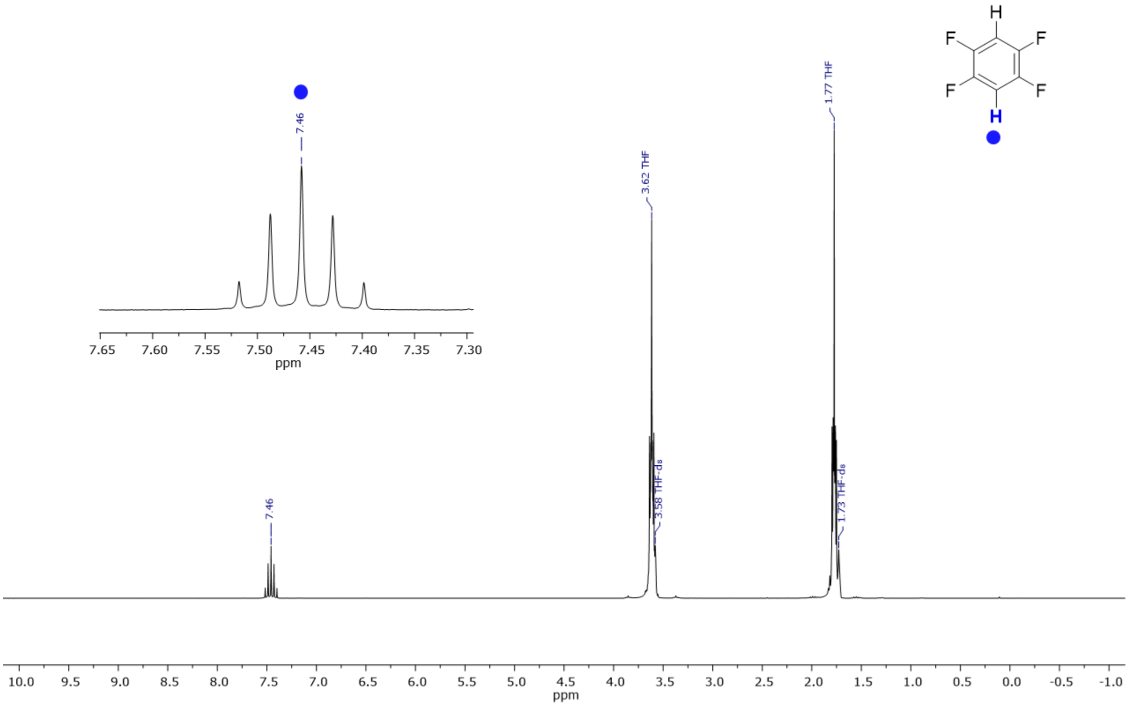

Figure S37: ^{1}H NMR spectrum (300.0 MHz, THF-d_8) of isolated 1,2,4,5-$C_6F_4H_2$. The THF originates from the reaction solution and could not be completely removed.

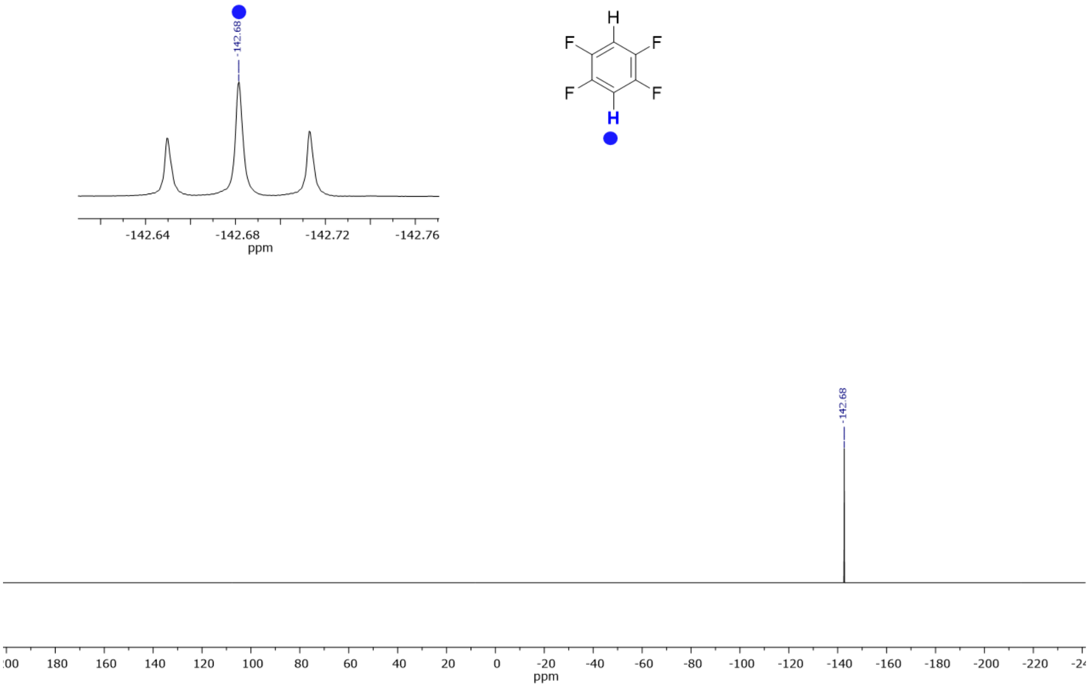

Figure S38: ^{19}F NMR spectrum (282.3 MHz, THF-d_8) of isolated 1,2,4,5-$C_6F_4H_2$.

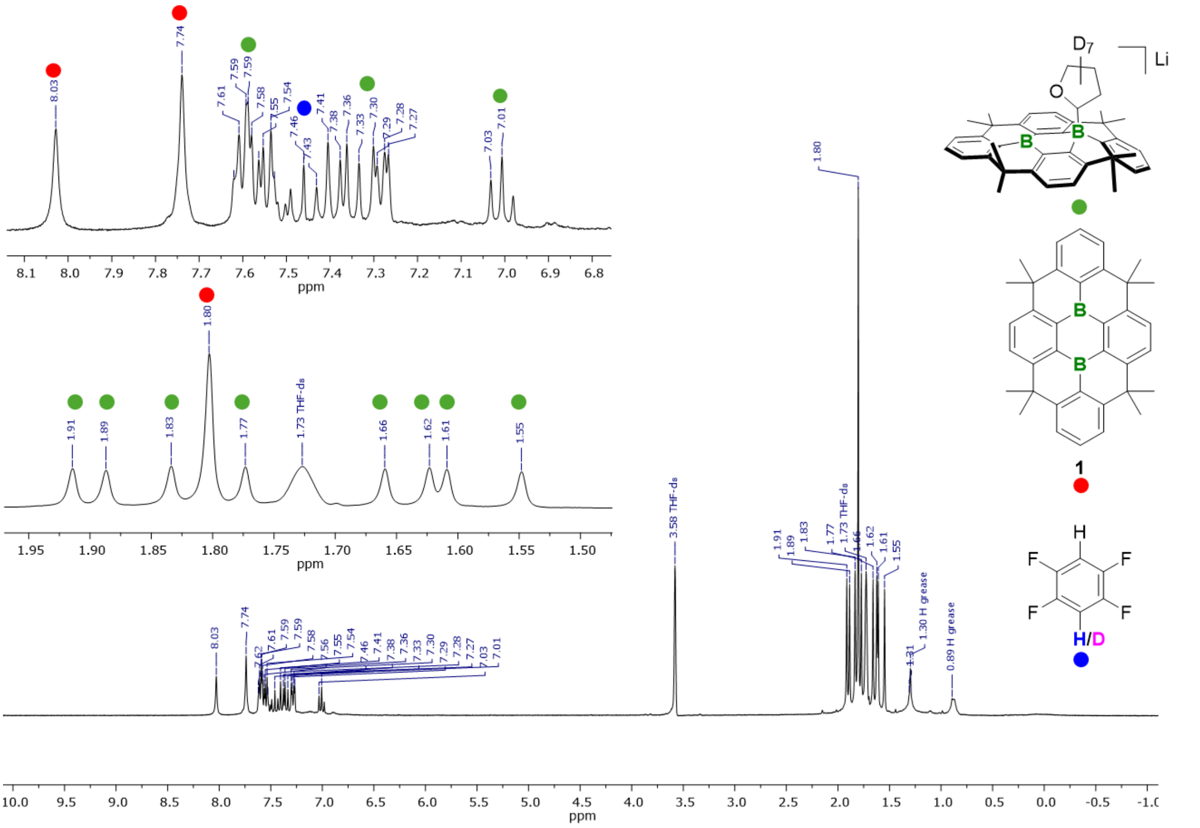

Figure S39: ^{1}H NMR spectrum (300.0 MHz, THF-d_8) of the reaction mixture of Li_2[**1**] with C_6F_5H after heating to 50 °C for 12 h. Compound **1** is formed from the thermolabile substitution product.

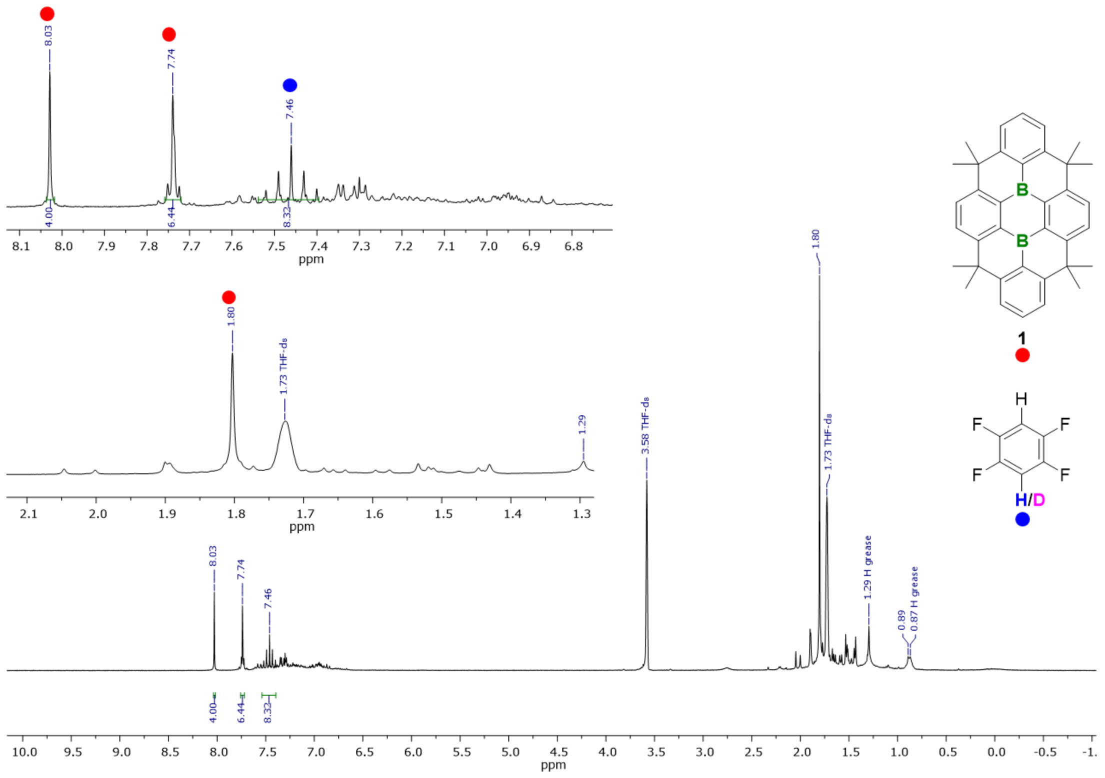

Figure 40: ^{1}H NMR spectrum (300.0 MHz, THF-d_8) of the reaction mixture of Li_2[**1**] with C_6F_5H after heating to 50 °C, followed by exposure of the sample to ambient air for 1 d. Since compound **1** is poorly soluble in THF and precipitates out of the solution, the integrals do not correspond to the expected 1:1 ratio with 1,2,4,5-$C_6F_4H_2$.

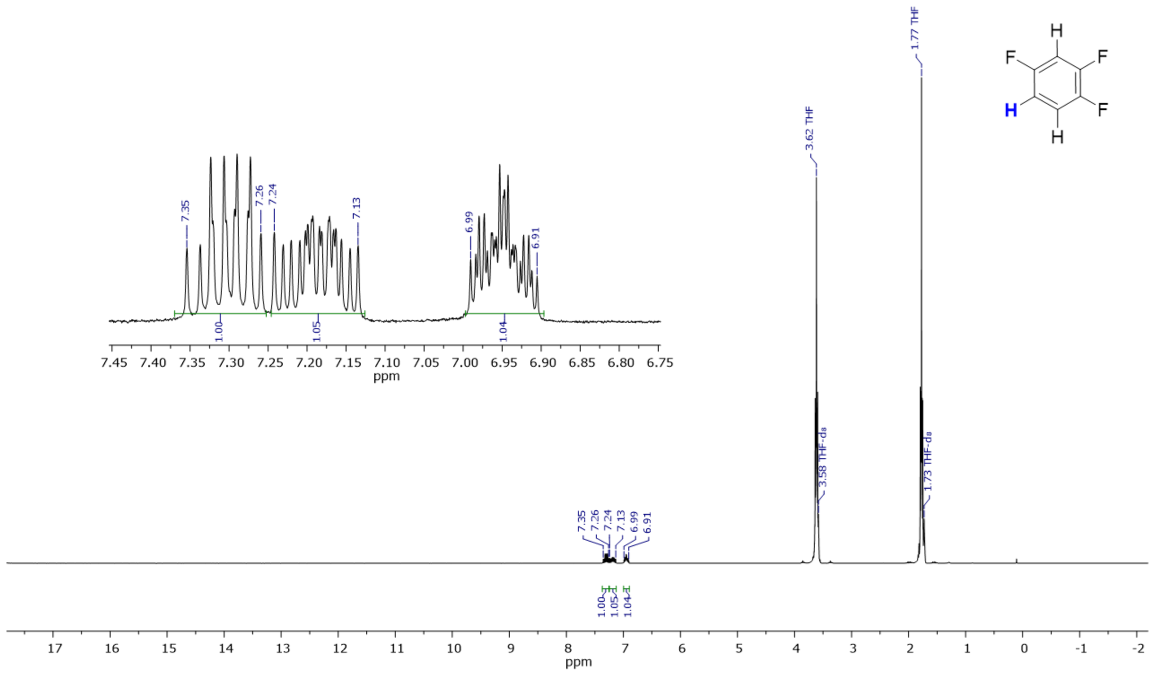

Figure S41: ^{1}H NMR spectrum (300.0 MHz, THF-d_8) of isolated 1,2,4-$C_6F_3H_3$. 1,2,4-$C_6F_3H_3$ can be isolated from the reaction of Li_2[**1**] with either 1 eq. 1,2,4,5-$C_6F_4H_2$ or 1,2,3,4-$C_6F_4H_2$ in THF. The THF originates from the reaction solution and could not be completely removed.

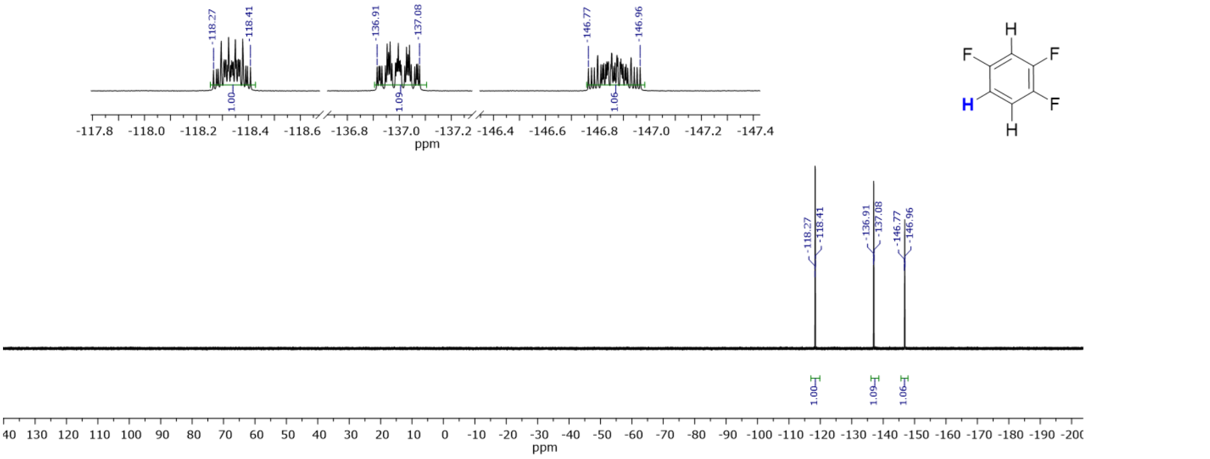

Figure S42: ^{19}F NMR spectrum (282.3 MHz, THF-d_8) of isolated 1,2,4-$C_6F_3H_3$. 1,2,4-$C_6F_3H_3$ can be isolated from the reaction of Li_2[**1**] with either 1 eq. 1,2,4,5-$C_6F_4H_2$ or 1,2,3,4-$C_6F_4H_2$ in THF.

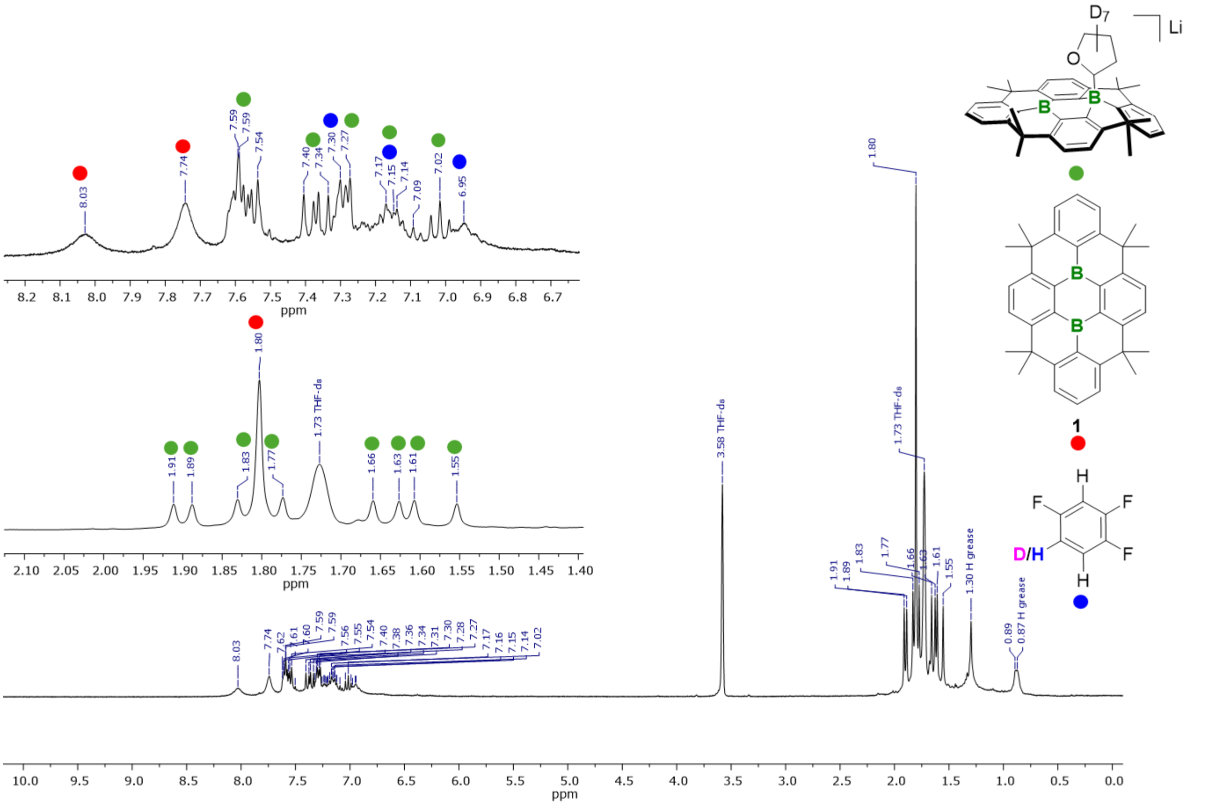

Figure S43: ^{1}H NMR spectrum (300.0 MHz, THF-d_8) of the reaction mixture of Li_2[**1**] with 1,2,4,5-$C_6F_4H_2$ after heating to 100 °C for 3 d. Compound **1** is formed from the thermolabile substitution product.

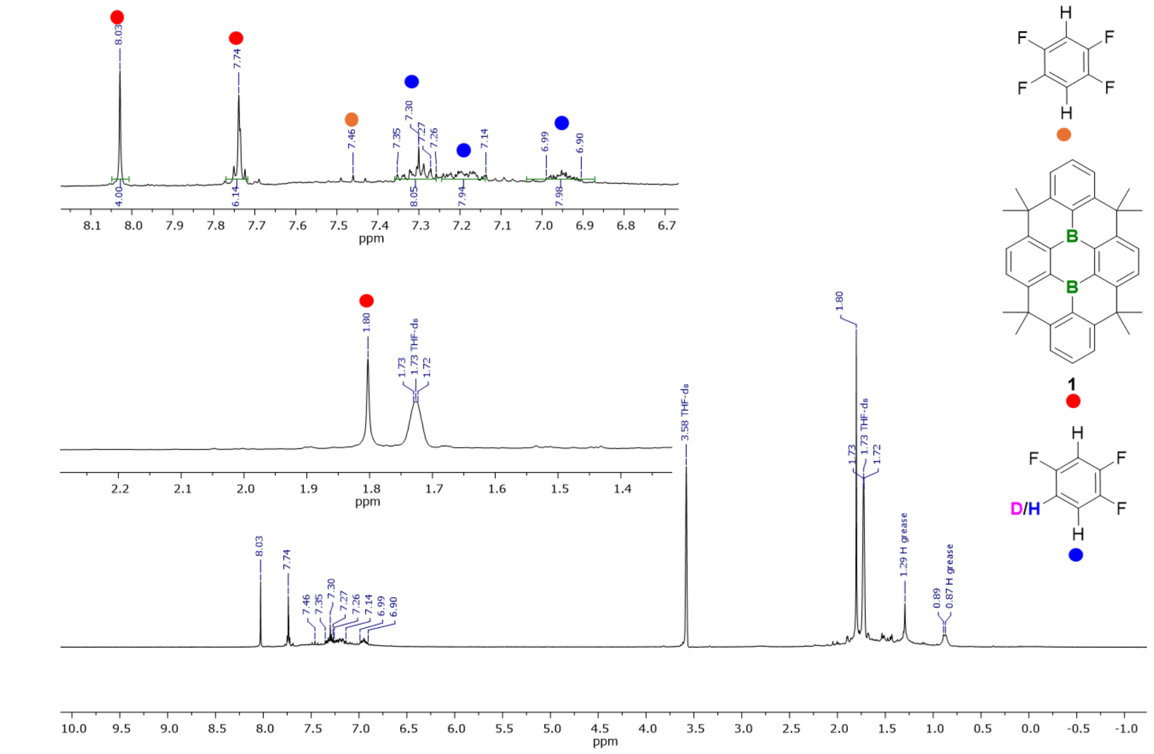

Figure S44: ^{1}H NMR spectrum (300.0 MHz, THF-d_8) of the reaction mixture of Li_2[**1**] with 1,2,4,5-$C_6F_4H_2$ after heating to 100 °C, followed by exposure of the sample to ambient air for 1 d. Since compound **1** is poorly soluble in THF and precipitates out of the solution, the integrals do not correspond to the expected 1:1 ratio with 1,2,4-$C_6F_3H_2$.

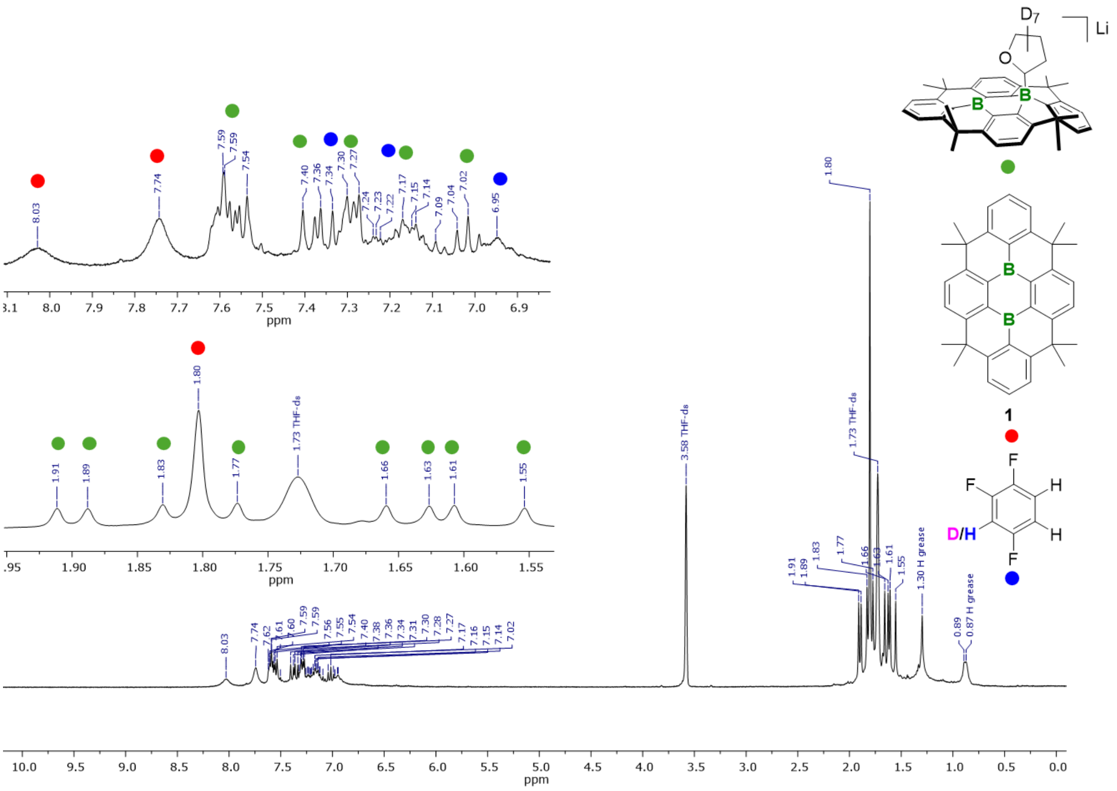

Figure 45: ^{1}H NMR spectrum (300.0 MHz, THF-d_8) of the reaction mixture of Li_2[**1**] with 1,2,3,4-$C_6F_4H_2$ after heating to 100 °C for 3 d. Compound **1** is formed from the thermolabile substitution product.

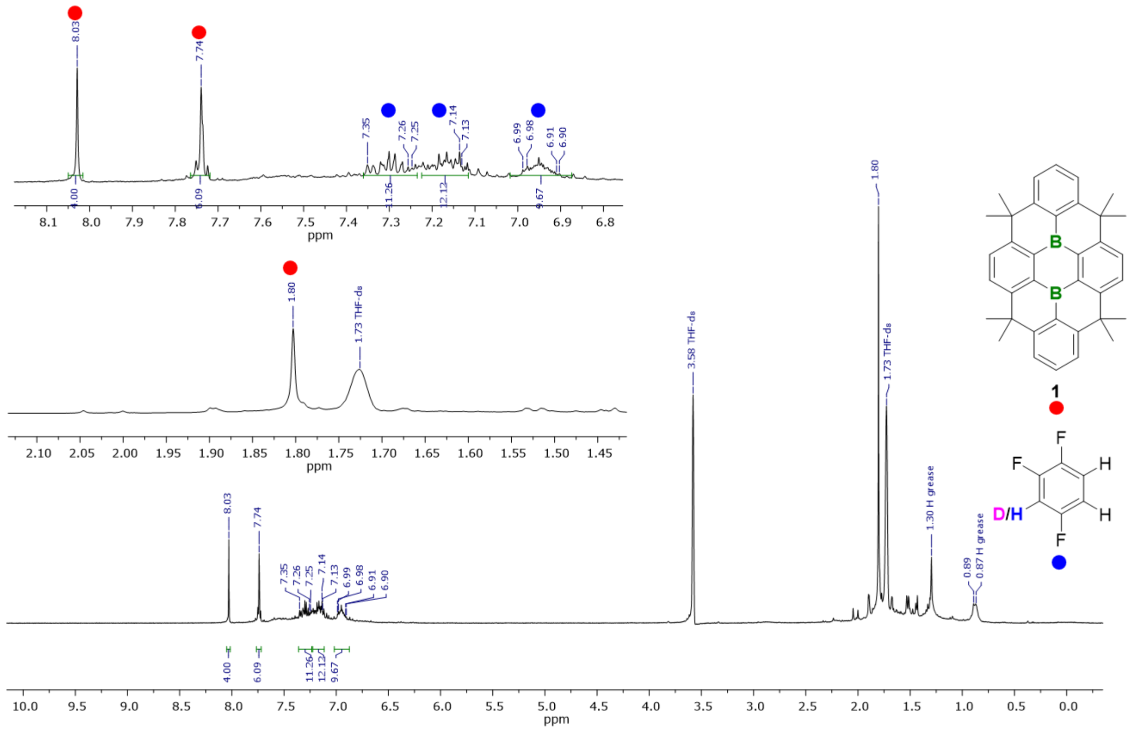

Figure S46: ^{1}H NMR spectrum (300.0 MHz, THF-d_8) of the reaction mixture of Li_2[**1**] with 1,2,3,4-$C_6F_4H_2$ after heating to 100 °C, followed by exposure of the sample to ambient air for 1 d. Since compound **1** is poorly soluble in THF and precipitates out of the solution, the integrals do not correspond to the expected 1:1 ratio with 1,2,4-$C_6F_3H_2$.

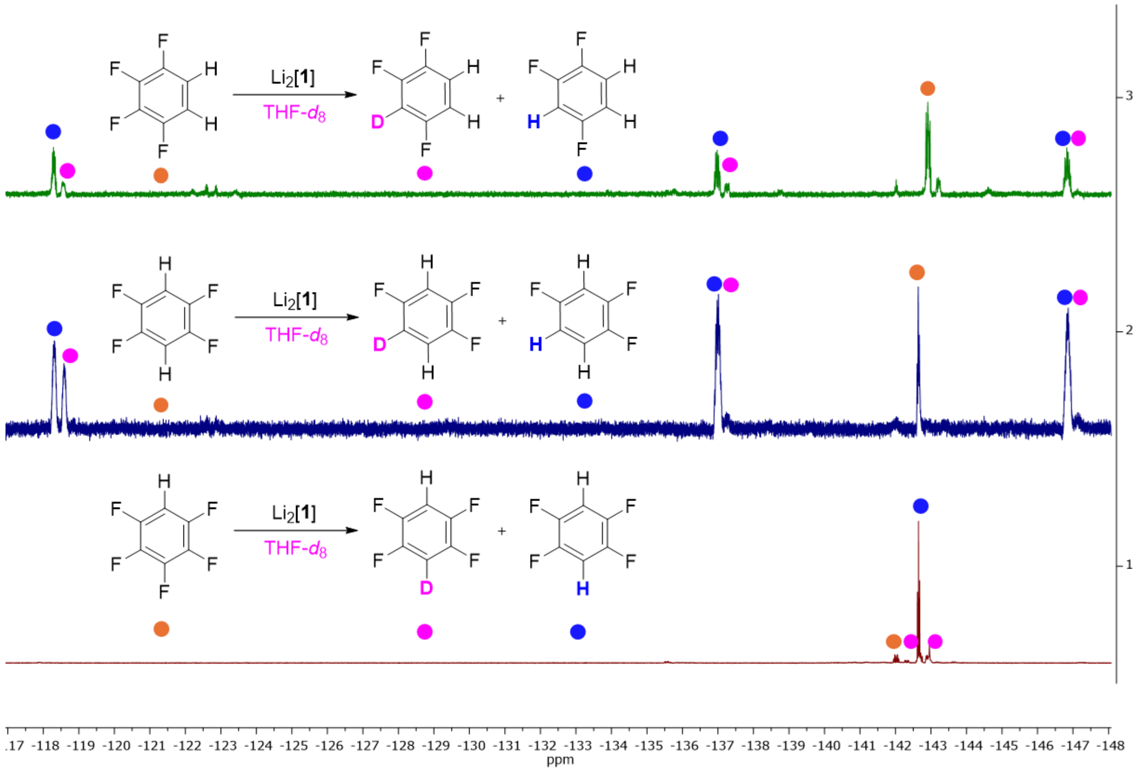

Figure S47: ^{19}F NMR spectra (282.3 MHz, THF-d_8) of the reactions of Li_2[**1**] with different fluorobenzenes in THF-d_8. Bottom: reaction with C_6F_5H; middle: reaction with 1,2,4,5-$C_6F_4H_2$; top: reaction with 1,2,3,4-$C_6F_4H_2$. Color code: orange – starting material; pink – product with D incorporation; blue – product with H incorporation.

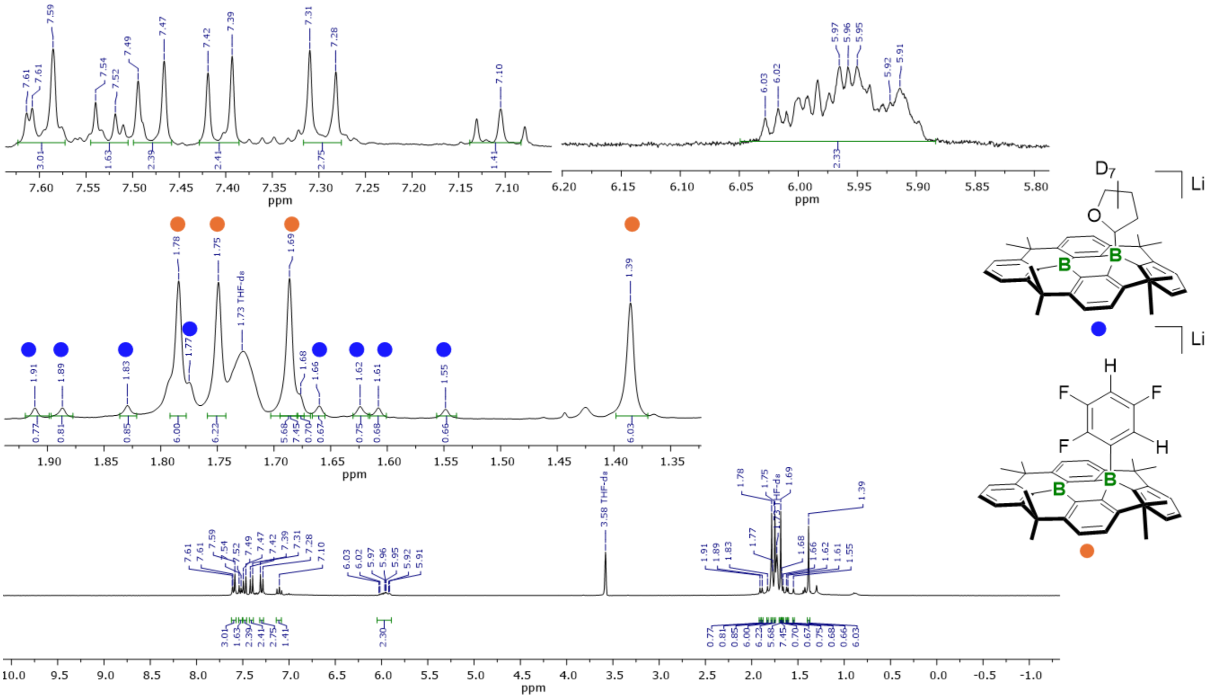

Figure S48: ^{1}H NMR spectrum (300.0 MHz, THF-d_8) of the reaction mixture of Li_2[**1**] with 1,2,3,5-$C_6F_4H_2$ after heating to 80 °C for 2 d. Li[**5**] (orange dots) forms as the main product, accompanied by Li[$\mathbf{3^D}$] (blue dots), a minor S_NAr side product, and hydrodefluorination products.

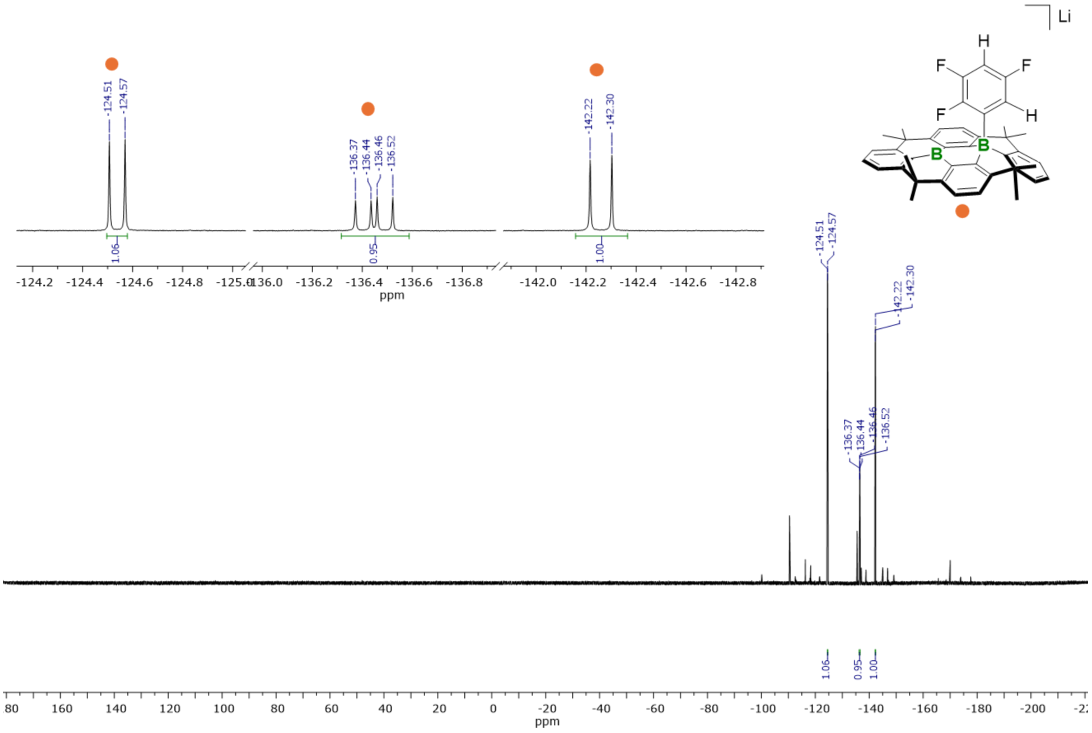

Figure S49: The $^{19}F\{^{1}H\}$ NMR spectrum (282.3 MHz, THF-d_8) of the reaction of Li_2[**1**] with 1,2,3,5-$C_6F_4H_2$ reveals the formation of Li[**5**] (orange dots) as the major product.

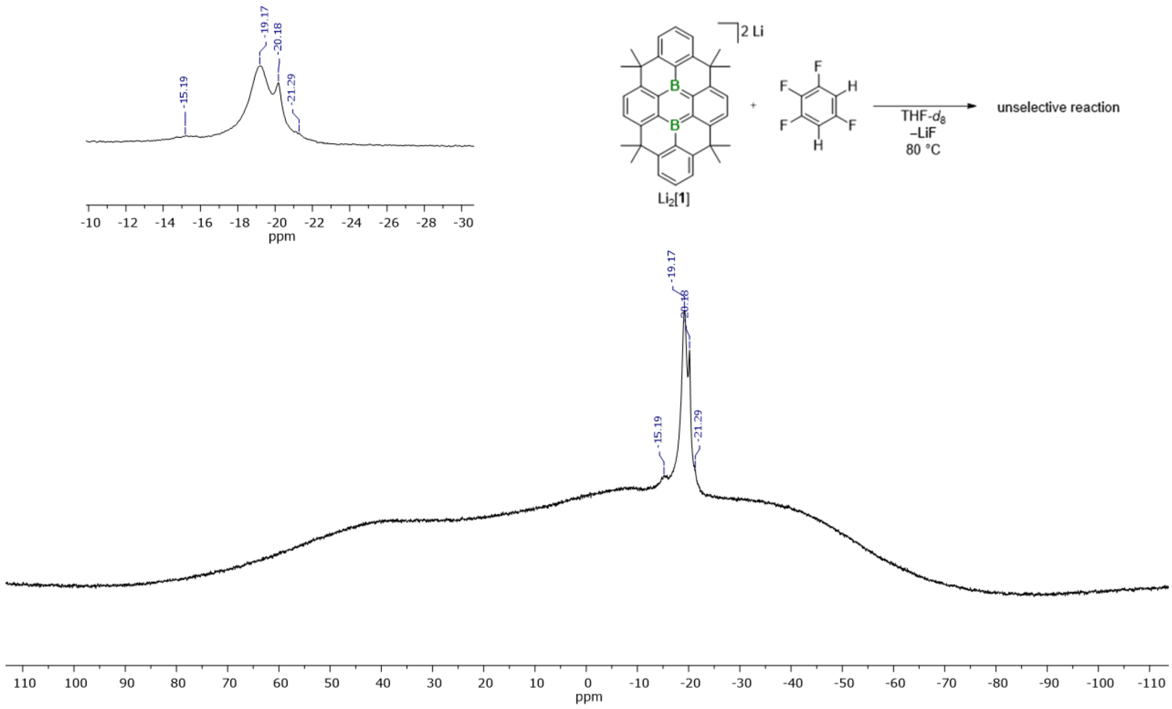

Figure S50: ^{11}B NMR spectrum (96.3 MHz, THF-d_8) of the reaction of Li_2[**1**] with 1,2,3,5-$C_6F_4H_2$.

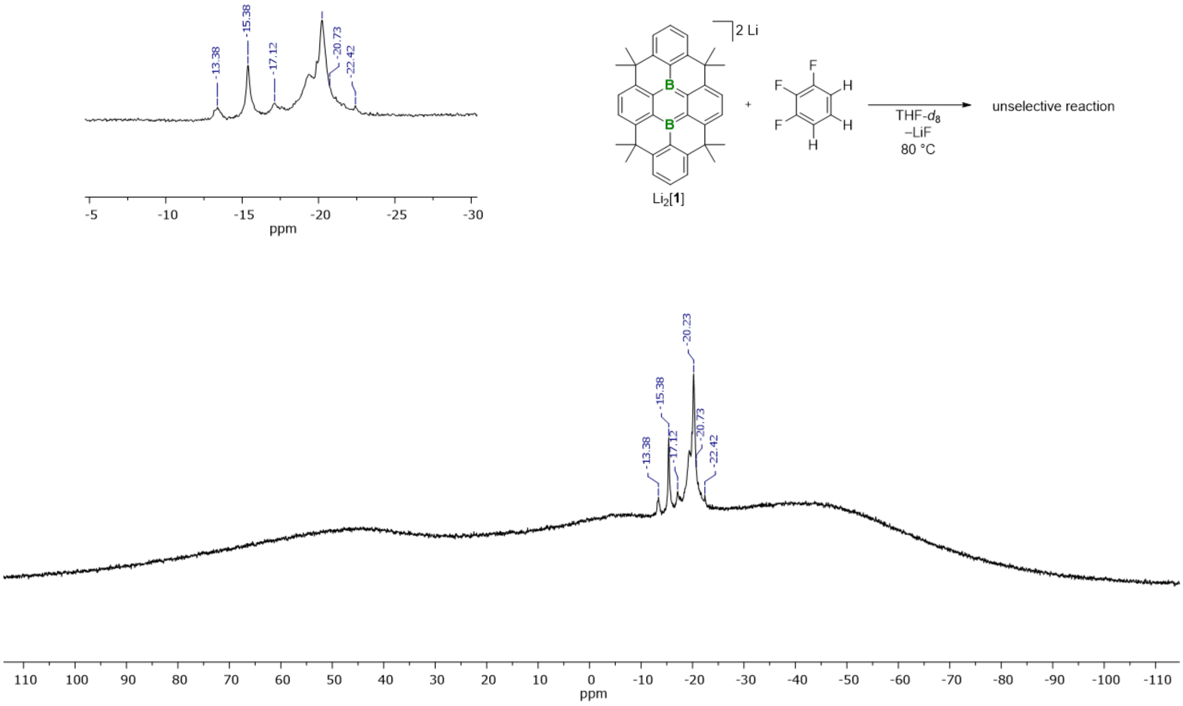

Figure S51: ^{11}B NMR spectrum (96.3 MHz, THF-d_8) of the reaction of Li_2[**1**] with 1,2,3-$C_6F_3H_3$.

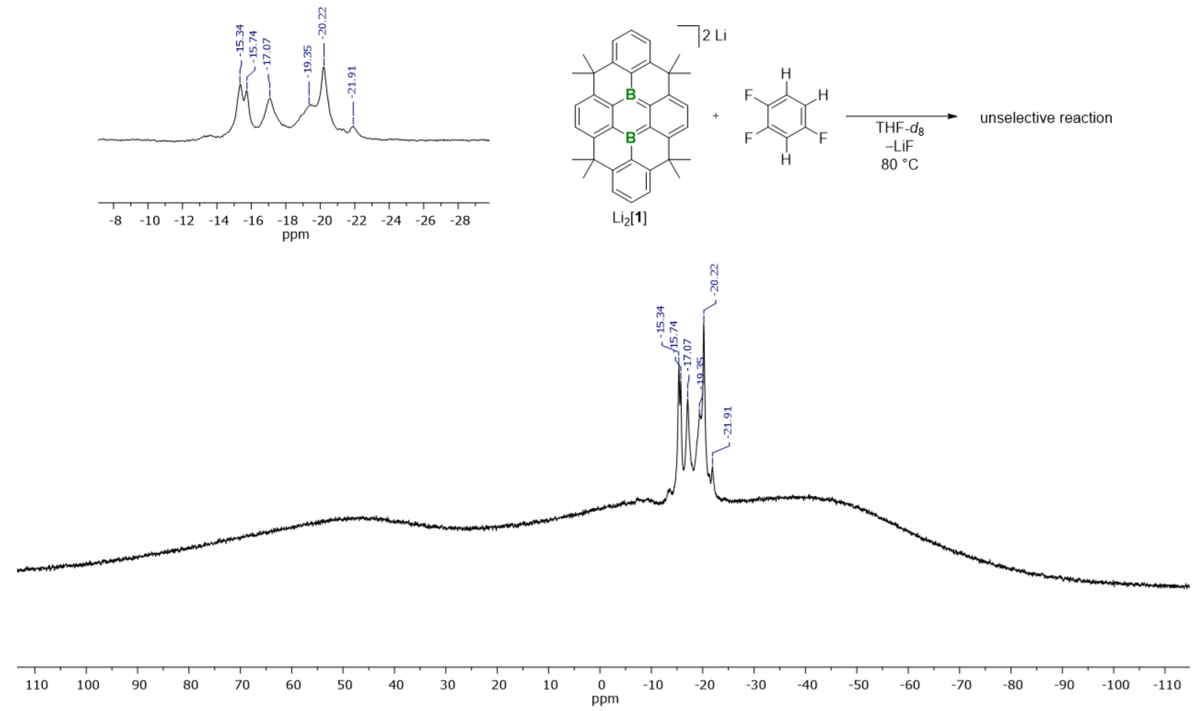

Figure S52: ^{11}B NMR spectrum (96.3 MHz, THF-d_8) of the reaction of Li_2[**1**] with 1,2,4-$C_6F_3H_3$.

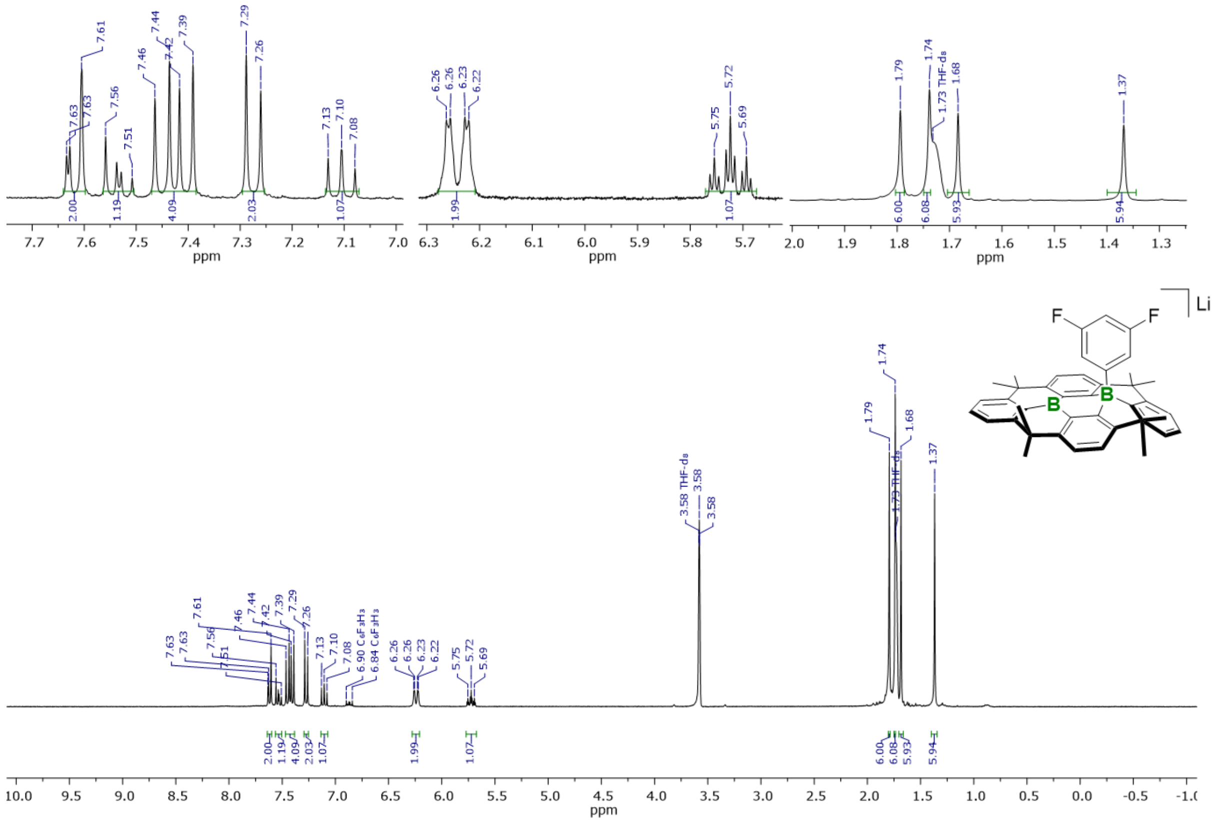

Figure S53: ^{1}H NMR spectrum (500.2 MHz, THF-d_8) of Li[**2**]. Contaminant: 1,3,5-$C_6F_3H_3$.

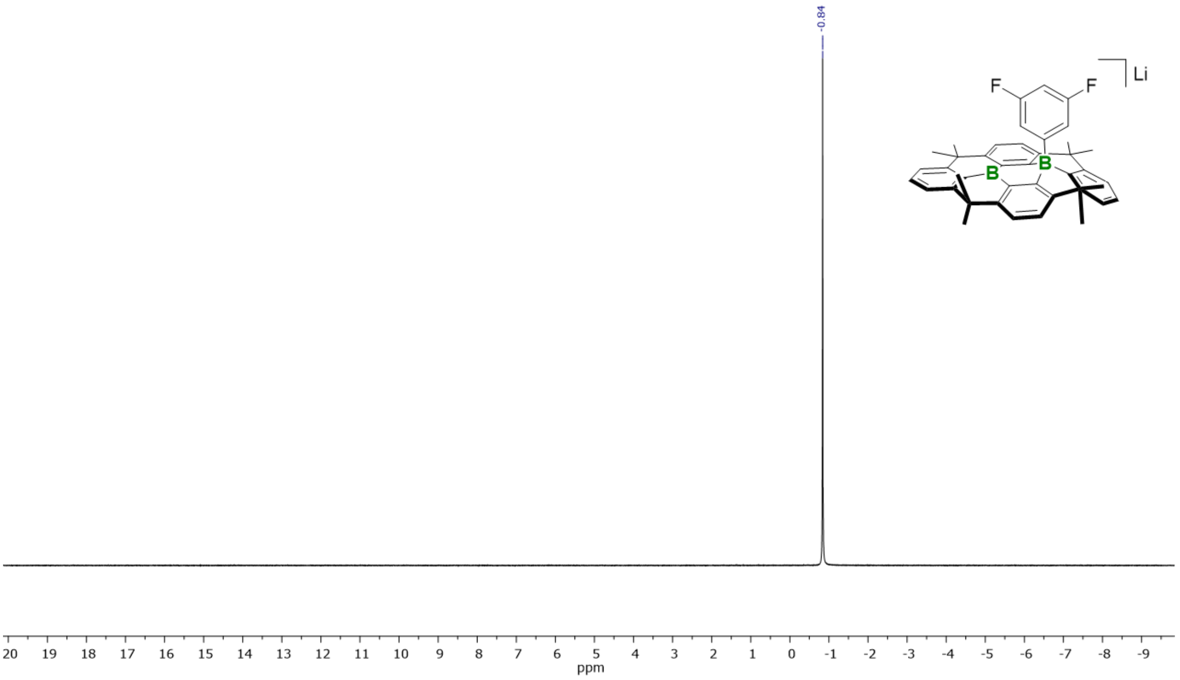

Figure S54: ^{7}Li NMR spectrum (194.4 MHz, THF-d_8) of Li[**2**].

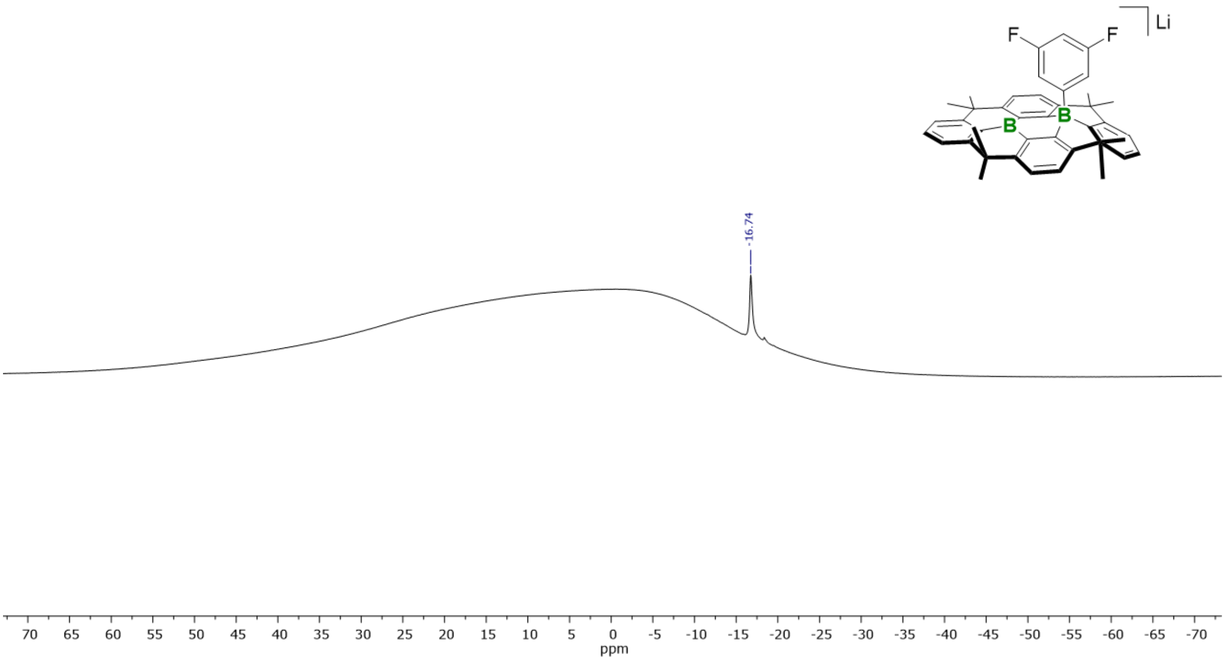

Figure S55: ^{11}B NMR spectrum (160.5 MHz, THF-d_8) of Li[**2**].

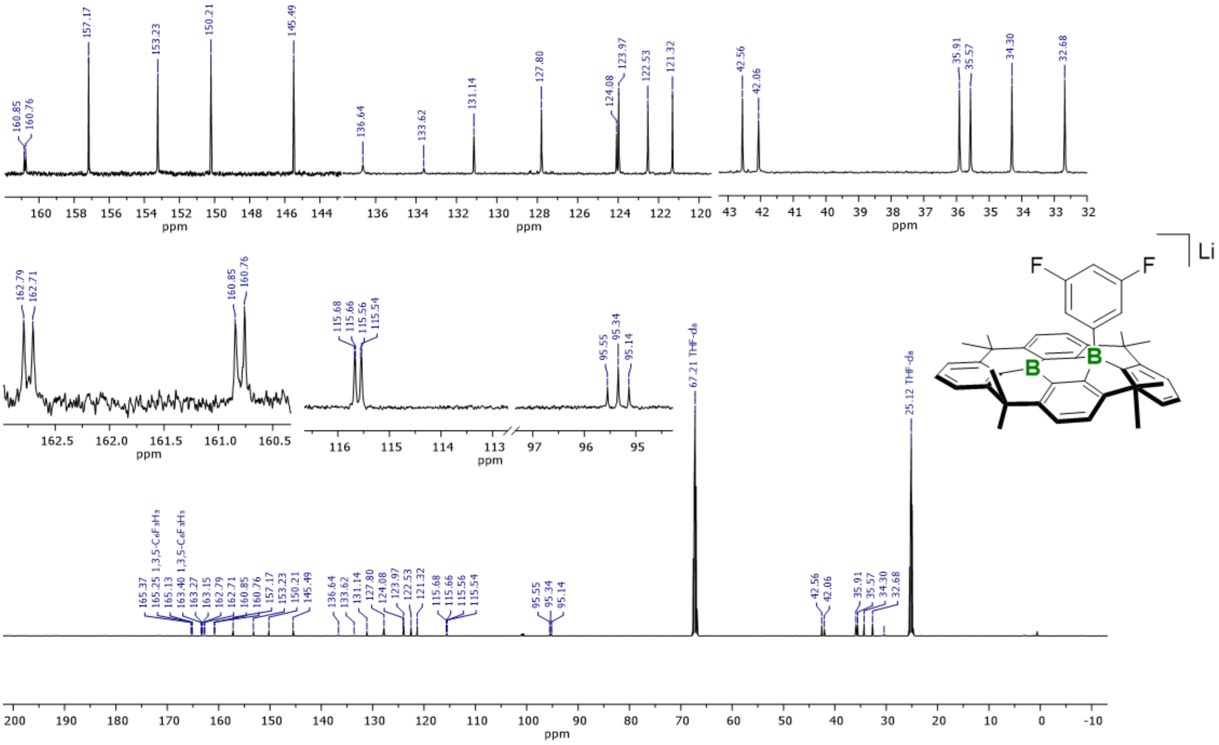

Figure S56: $^{13}C\{^1H\}$ NMR spectrum (125.8 MHz, THF-d_8) of Li[**2**]. Contaminant: 1,3,5-$C_6F_3H_3$.

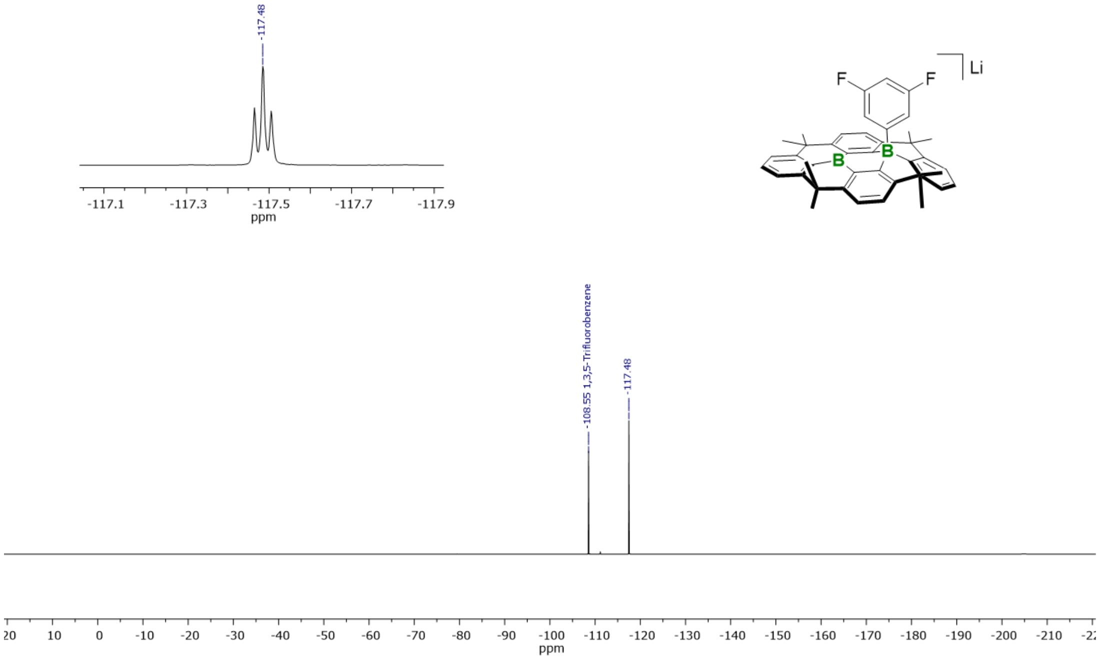

Figure S57: ^{19}F NMR spectrum (470.6 MHz, THF-d_8) of Li[**2**]. Contaminant: 1,3,5-$C_6F_3H_3$.

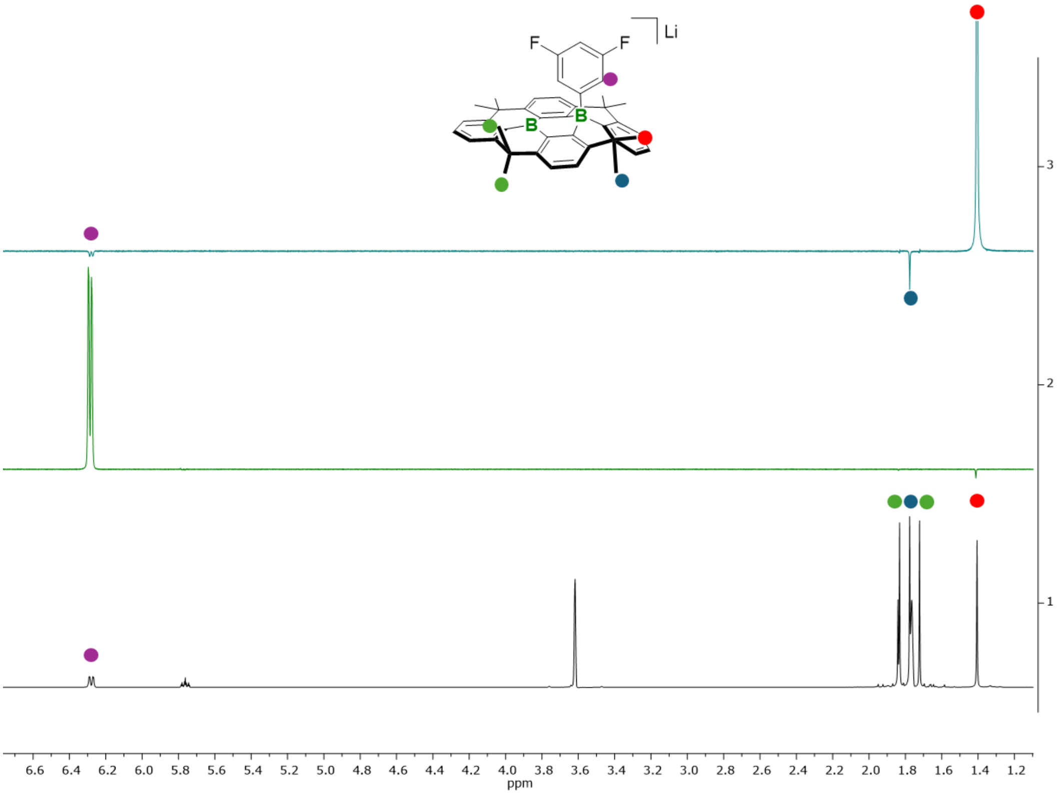

Figure S58: Expanded regions of the ^{1}H NMR and ^{1}H-^{1}H NOESY NMR spectra (500.2 MHz, THF-d_8) of Li[**2**]. Bottom: ^{1}H NMR spectrum of Li[**2**]. Middle: Selective excitation of a proton resonance of the $C_6F_2H_3$ substituent (6.24 ppm, purple) shows that the signal at 1.37 ppm (red) can be assigned to the C*Me* groups positioned on the same side as the substituent. Top: Selective excitation of the C*Me* groups resonating at 1.37 ppm (red) shows interaction with the respective C*Me* groups resonating at 1.74 ppm (blue), where the red- and blue-marked pair is bonded to the same C atom. Additionally, interactions with protons of the $C_6F_2H_3$ substituent (6.24 ppm, purple) are observed.

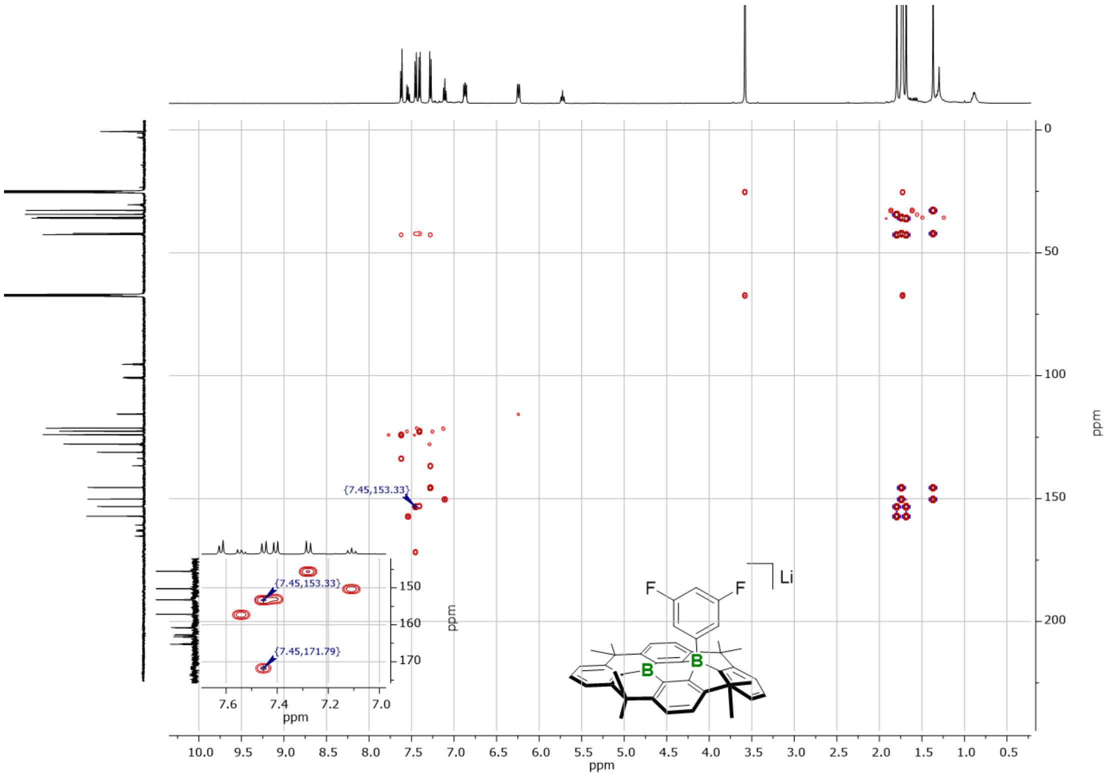

Figure S59: ^{1}H-^{13}C HMBC NMR (THF-d_8) spectrum of Li[**2**].

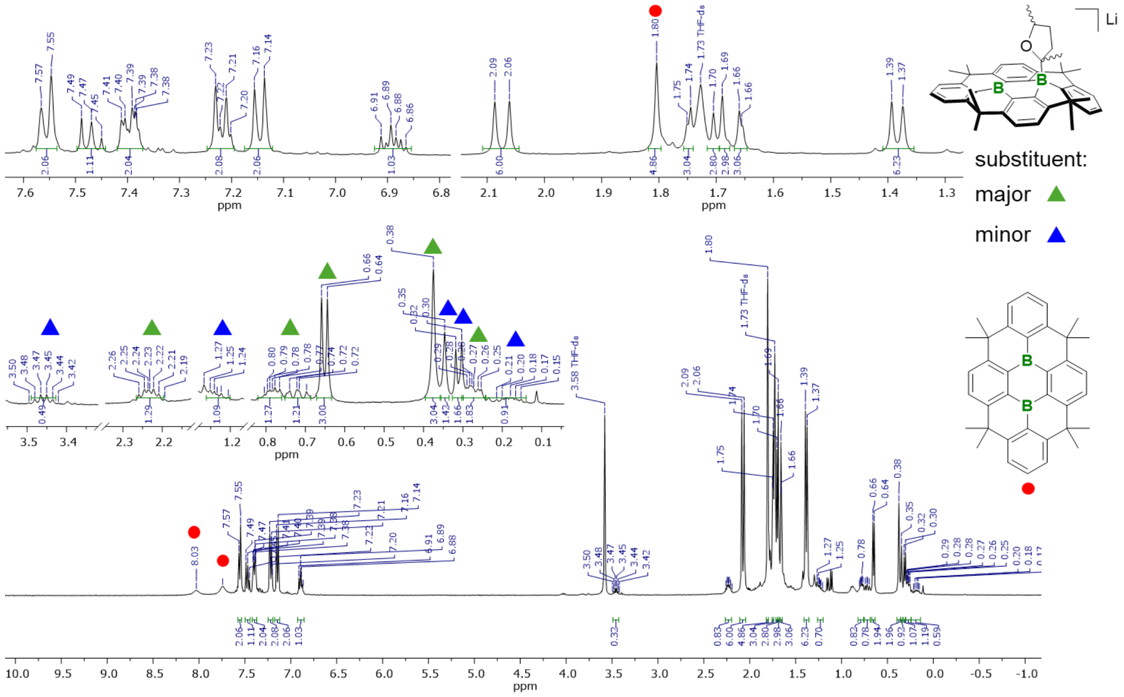

Figure S60: The ^{1}H-NMR spectrum (400.2 MHz, THF-d_8) of the reaction mixture of Li_2[**1**] with C_6F_6 in THF-Me_2 reveals the formation of diastereomeric Li[**3^{Me}**] as the predominant product, accompanied by the formation of **1** (red dots). The diastereomeric pairs can be distinguished for the B-bonded substituent (major isomer: green triangle; minor isomer: blue triangle). The other resonances of the diastereomeric pairs could not be distinguished, due to significant signal overlap. *Note*: In the top and bottom spectra, the resonances of the individual CH_3 groups of the DBA fragment have been normalized to 3H; in the middle spectrum, the resonance at 0.65 ppm, assignable to one CH_3 group of the substituent of the major diastereomer, has been normalized to 3H.

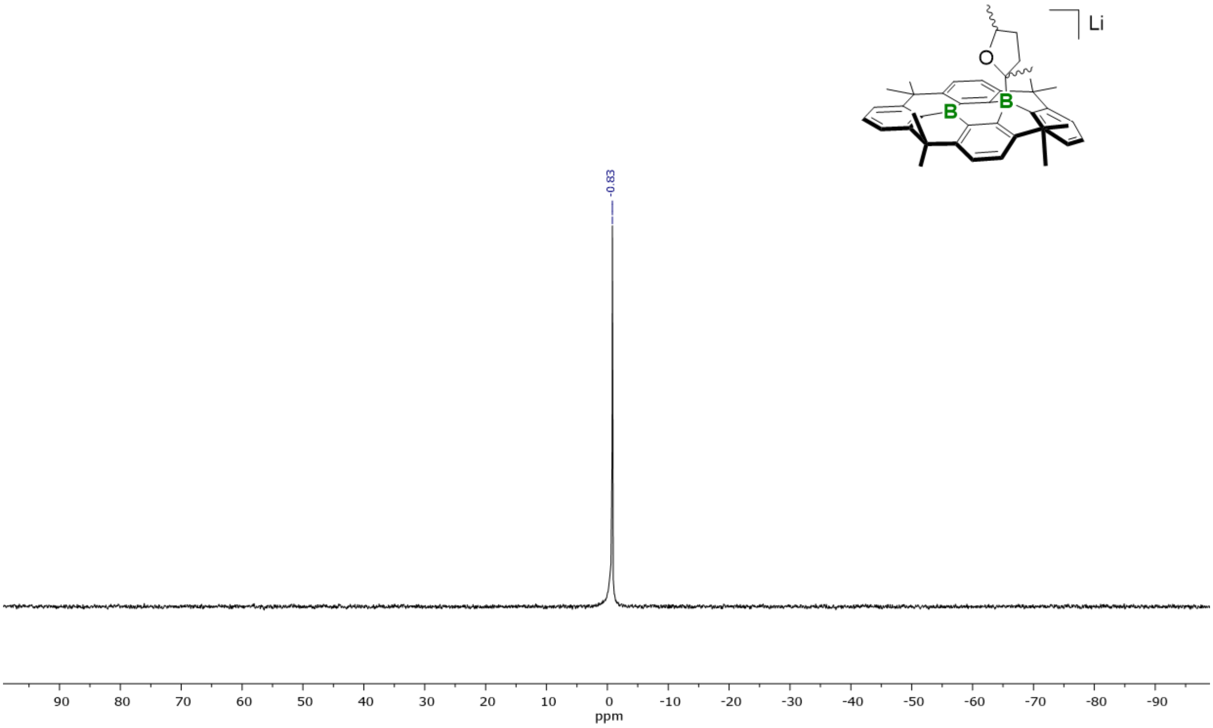

Figure S61: ^{7}Li NMR (155.5 MHz, THF-d_8) of Li[**3^{Me}**].

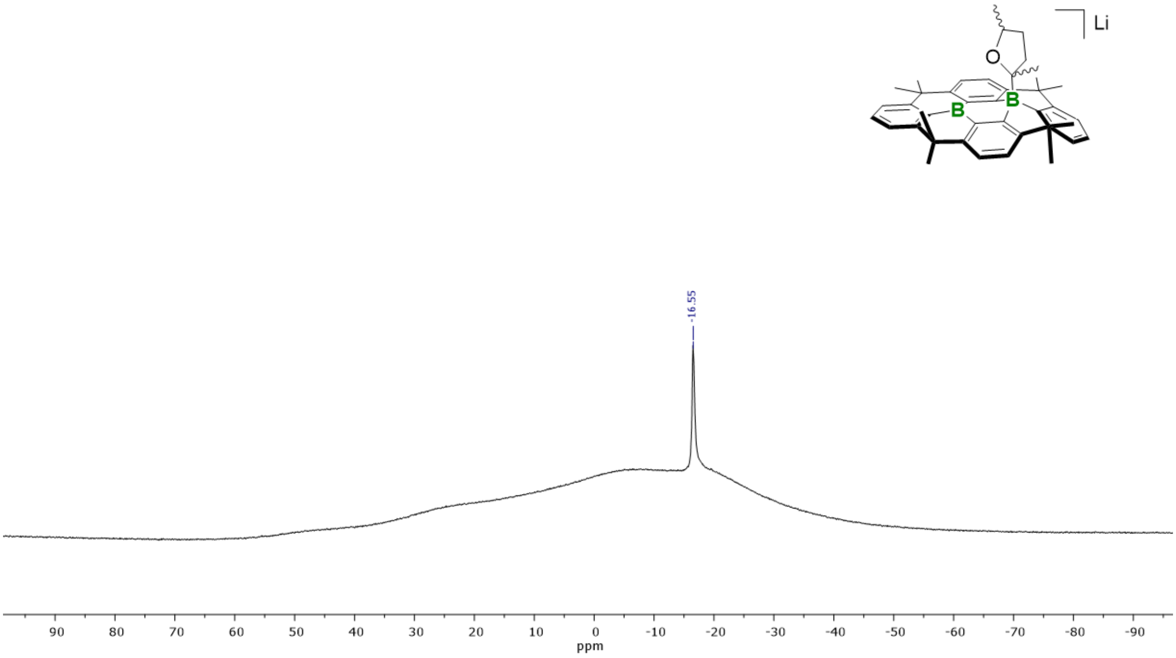

Figure S62: ^{11}B NMR (128.4 MHz, THF-d_8) of Li[**3^{Me}**].

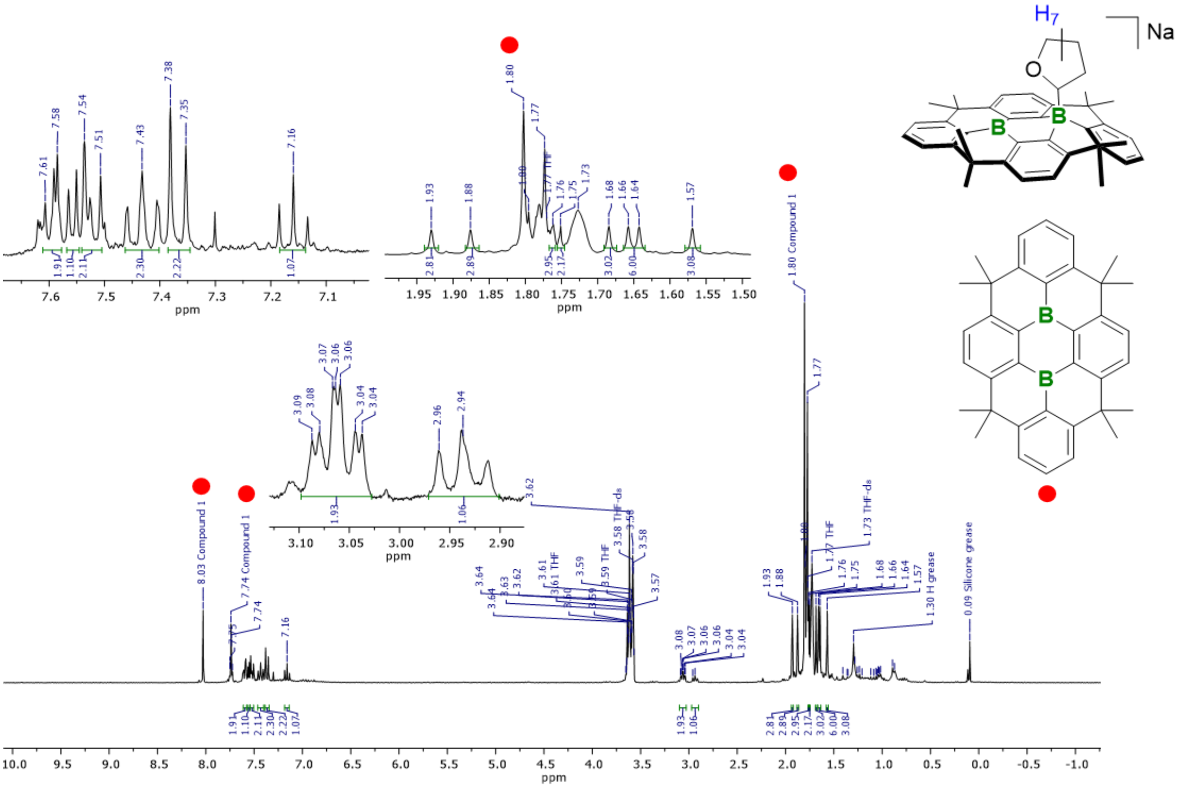

Figure S63: The ^{1}H-NMR spectrum (300.0 MHz, THF-d_8) of the reaction mixture of Na_2[**1**] with C_6F_6 in THF after heating to 50 °C for 12 h shows the formation of Na[**3**]. Contaminants: compound **1,** H grease and silicone grease.

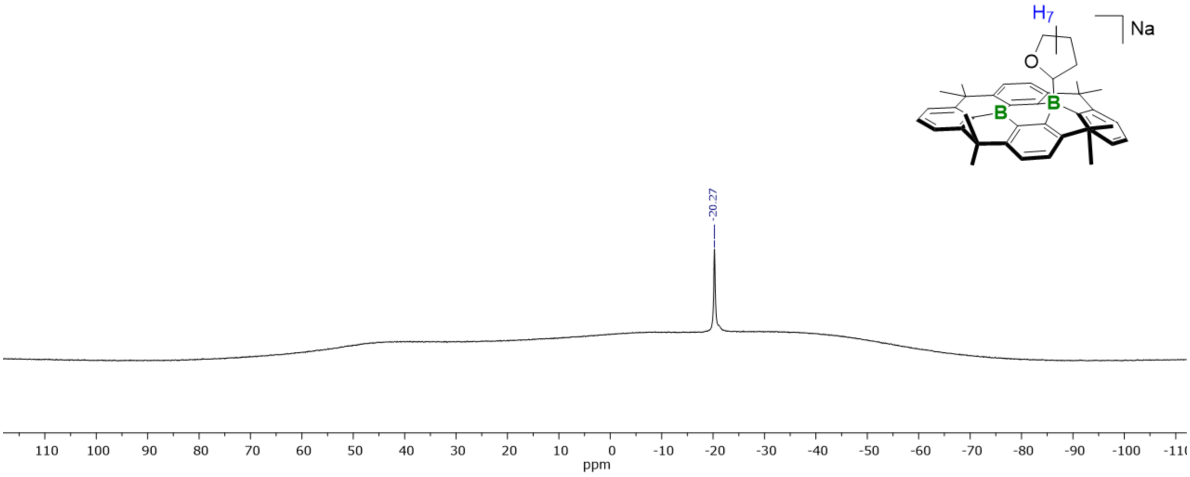

Figure S64: ^{11}B NMR spectrum (96.3 MHz, THF-d_8) of the reaction mixture of Na_2[**1**] with C_6F_6.

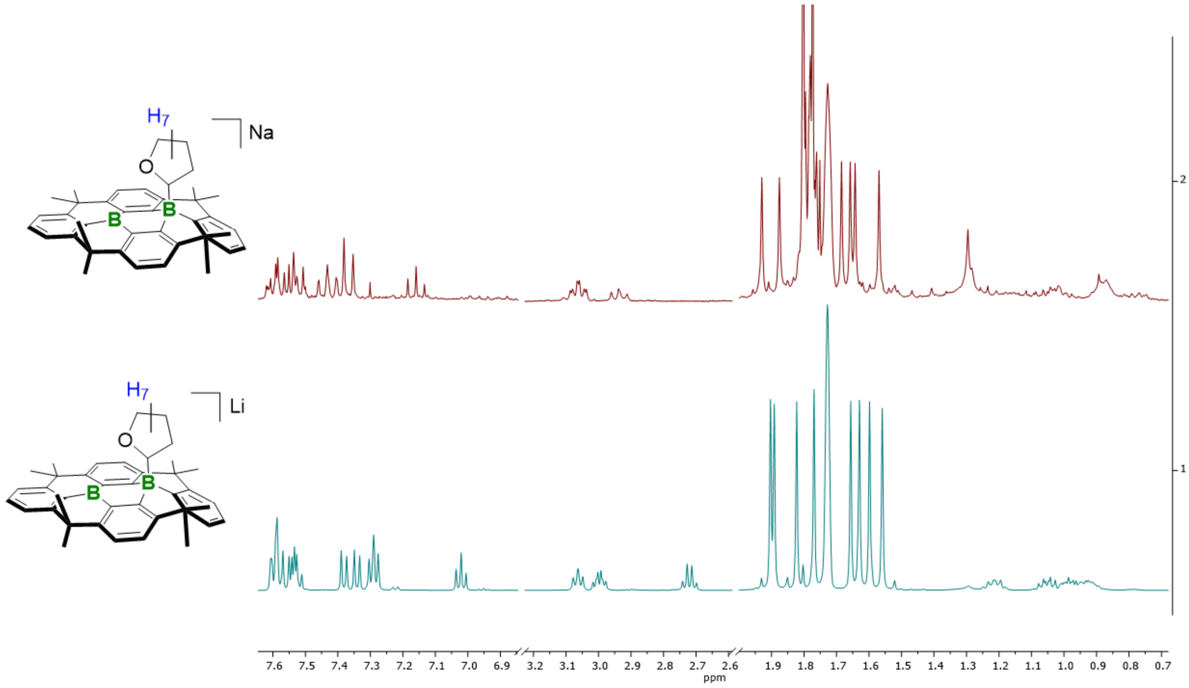

Figure S65: Comparison of expanded regions of the ^1H NMR spectra (500.2 MHz, THF-d_8 (bottom); 300.0 MHz, THF-d_8 (top)) of Li[**3**] (bottom) and of the reaction mixture of Na_2[**1**] with C_6F_6 (top). The purpose of this comparison is to demonstrate that, in the reaction of Na_2[**1**] with C_6F_6, the structurally similar Na[**3**] forms.

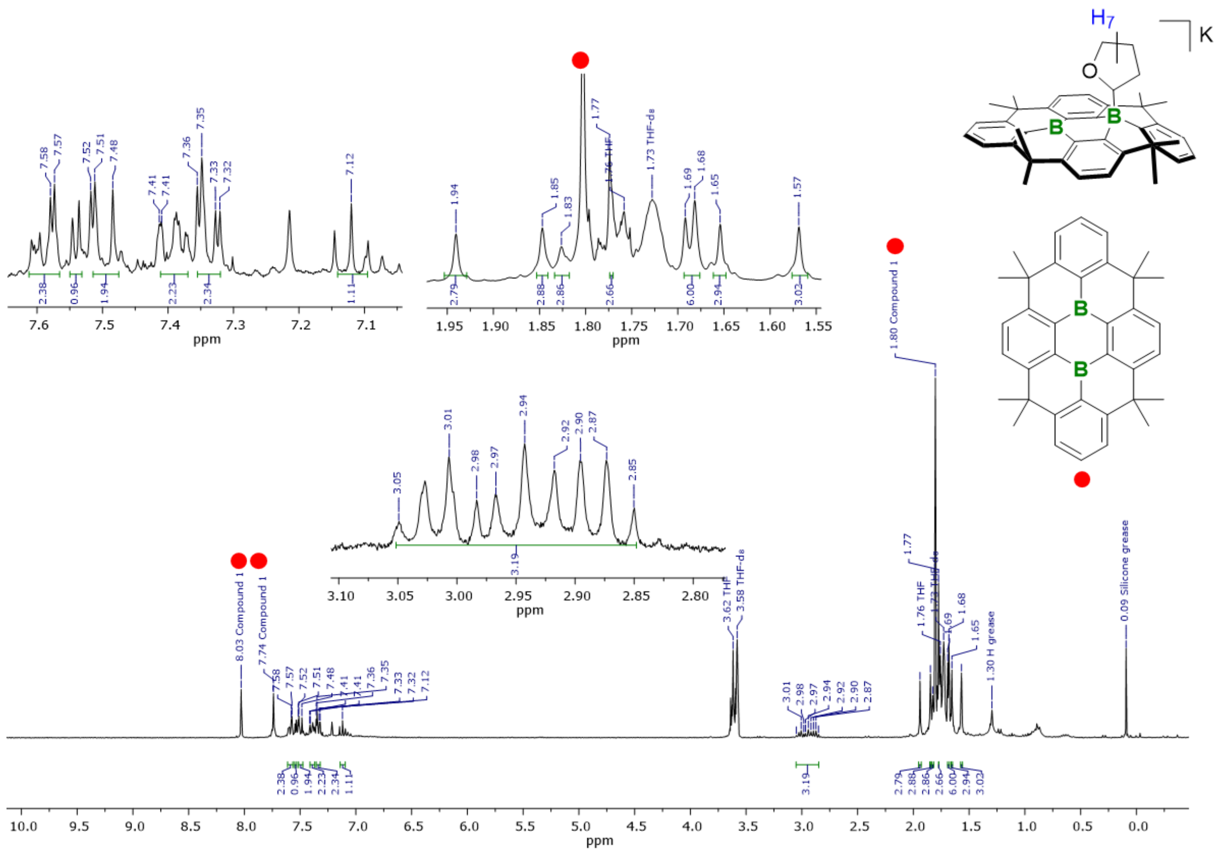

Figure S66: The ^{1}H-NMR spectrum (300.0 MHz, THF-d_8) of the reaction mixture of K_2[**1**] with C_6F_6 in THF after heating to 50 °C for 12 h, shows the formation of K[**3**]. Contaminants: compound **1,** H grease and silicone grease.

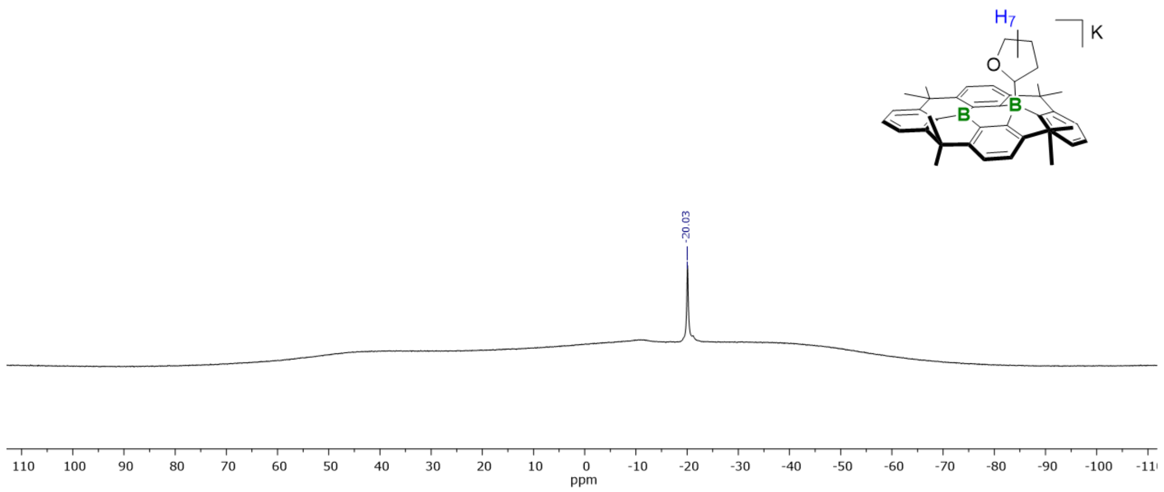

Figure S67: ^{11}B NMR spectrum (96.3 MHz, THF-d_8) of the reaction mixture of K_2[**1**] with C_6F_6.

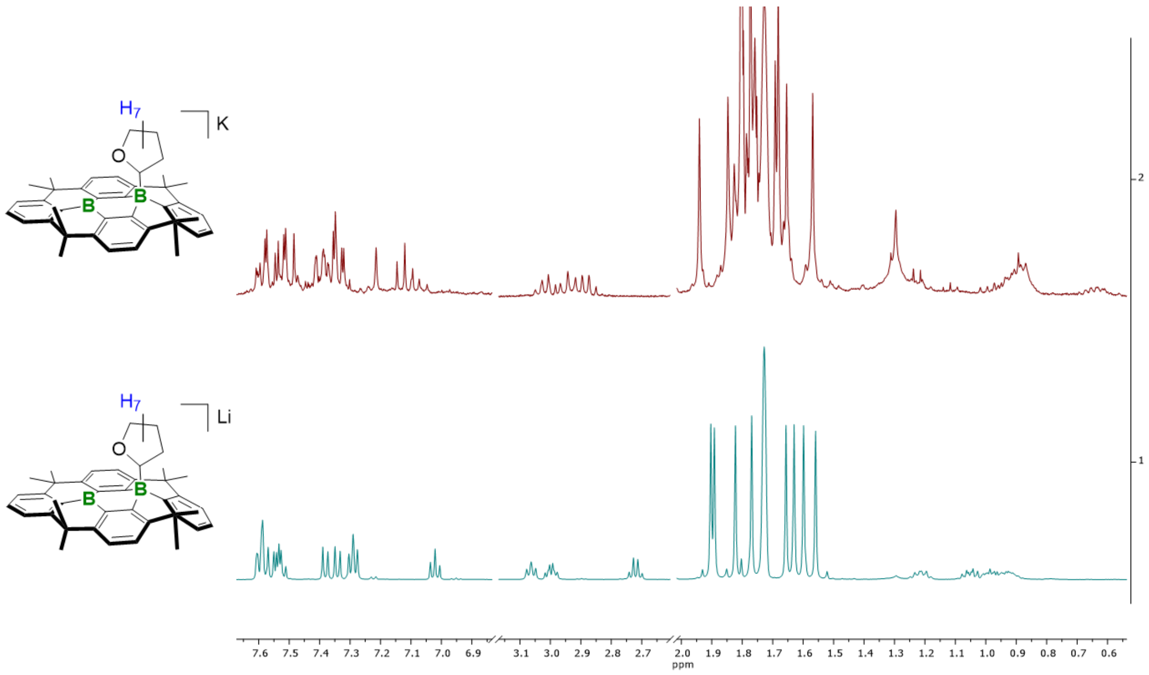

Figure S68: Comparison of expanded regions of the ^{1}H NMR spectra (500.2 MHz, THF-d_8 (bottom); 300.0 MHz, THF-d_8 (top)) of Li[**3**] (bottom) and of the reaction mixture of K_2[**1**] with C_6F_6 (top). The purpose of this comparison is to demonstrate that, in the reaction of K_2[**1**] with C_6F_6, the structurally similar K[**3**] forms.

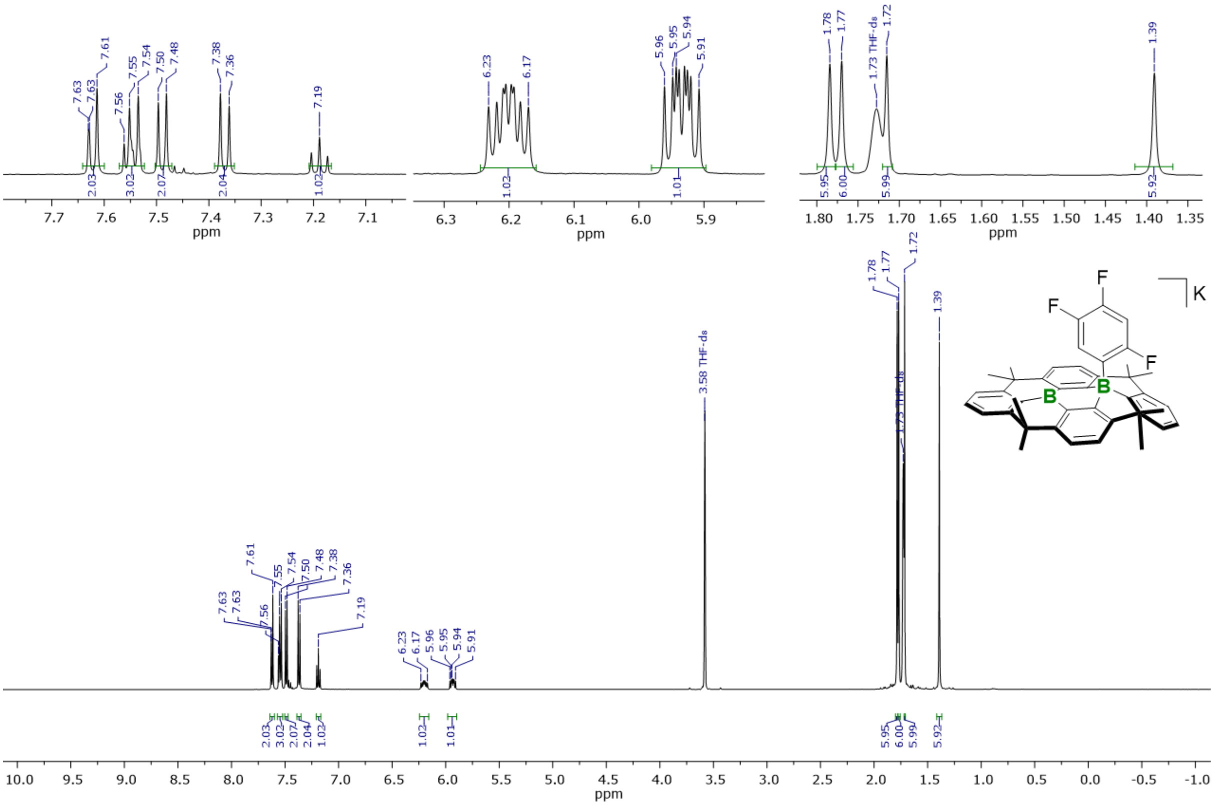

Figure S69: ^{1}H NMR spectrum (500.2 MHz, THF-d_8) of K[**6**].

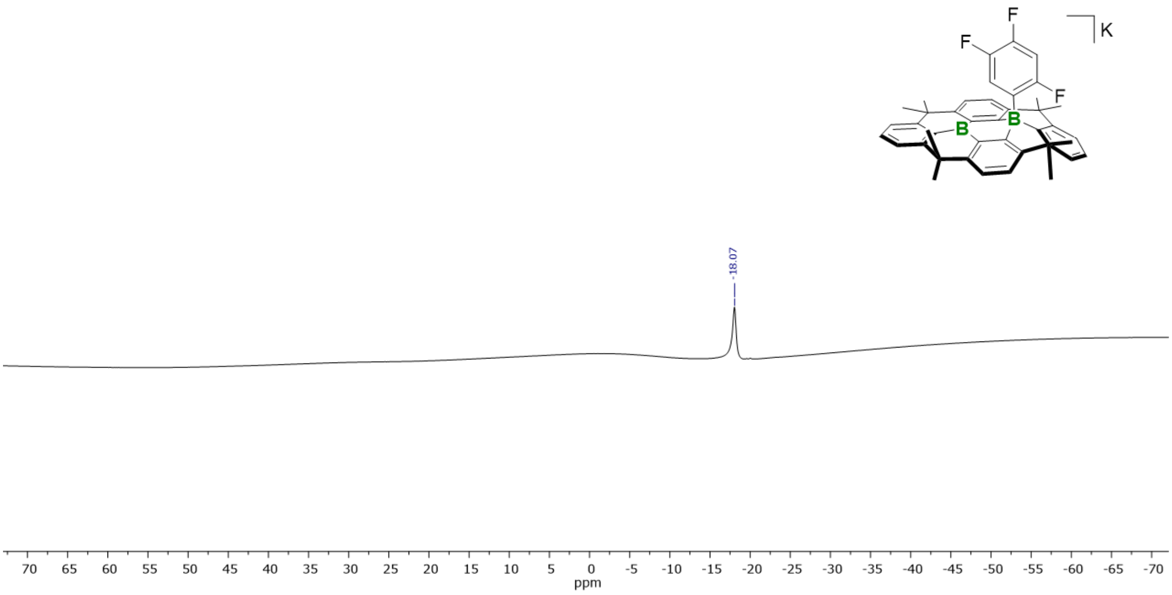

Figure S70: ^{11}B NMR spectrum (160.5 MHz, THF-d_8) of K[**6**].

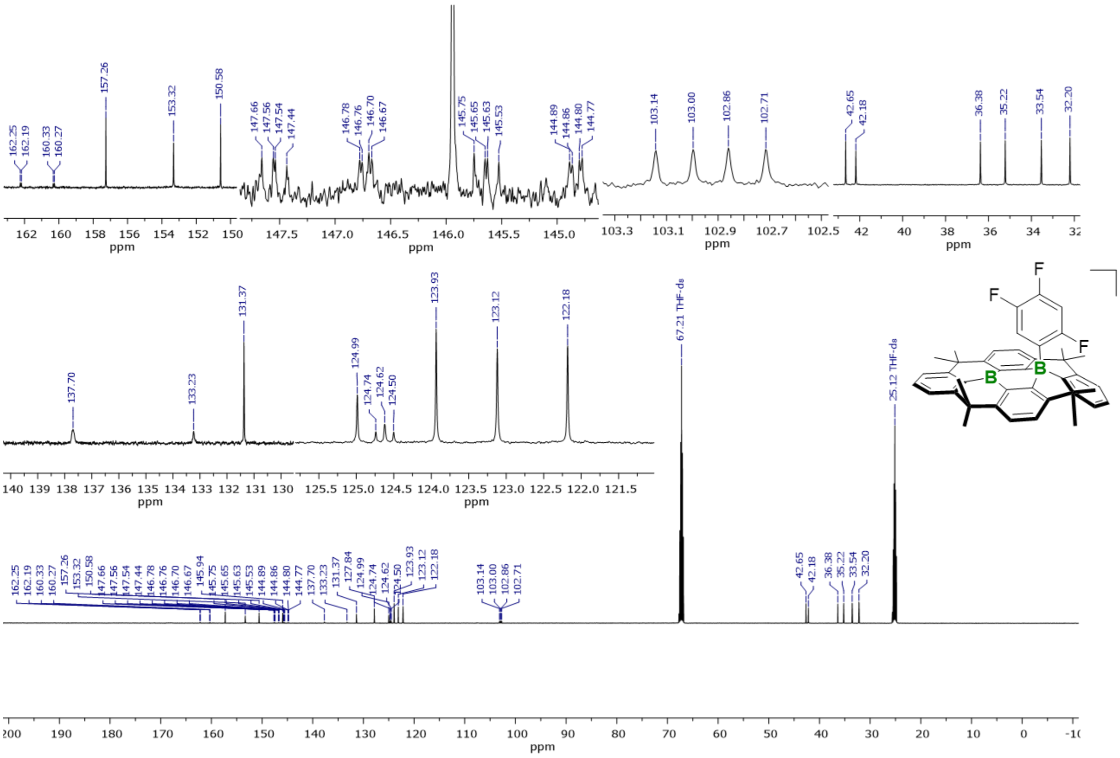

Figure S71: $^{13}C\{^1H\}$ NMR spectrum (125.8 MHz, THF-d_8) of K[**6**].

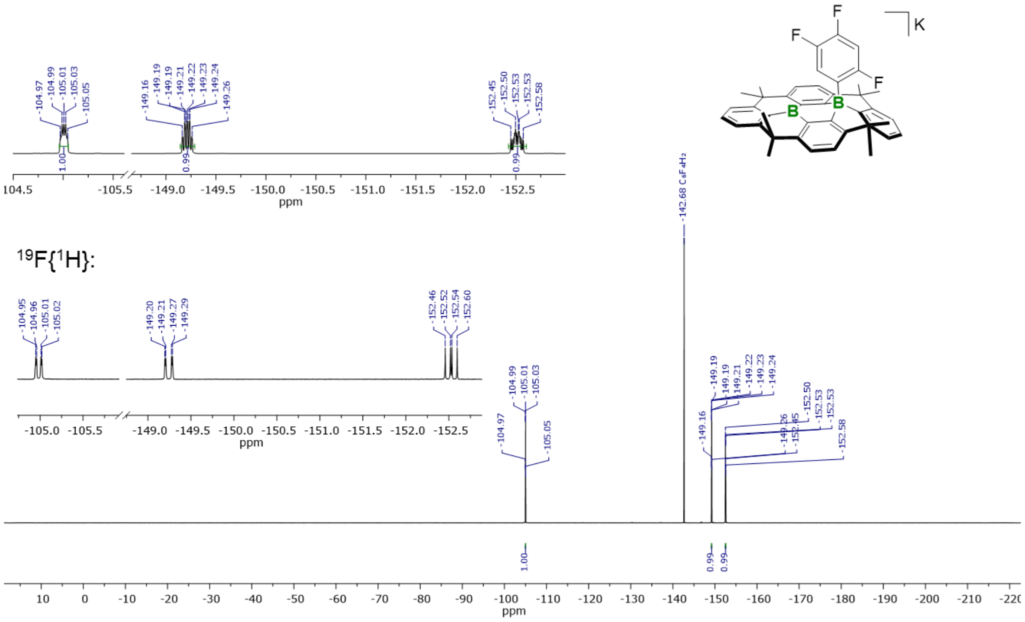

Figure S72: ^{19}F NMR spectrum (470.6 MHz, THF-d_8) of K[**6**]. The second enlarged region shows the $^{19}F\{^1H\}$ NMR spectrum (470.6 MHz, THF-d_8) of K[**6**]. Contaminant: 1,2,4,5-$C_6F_4H_2$.

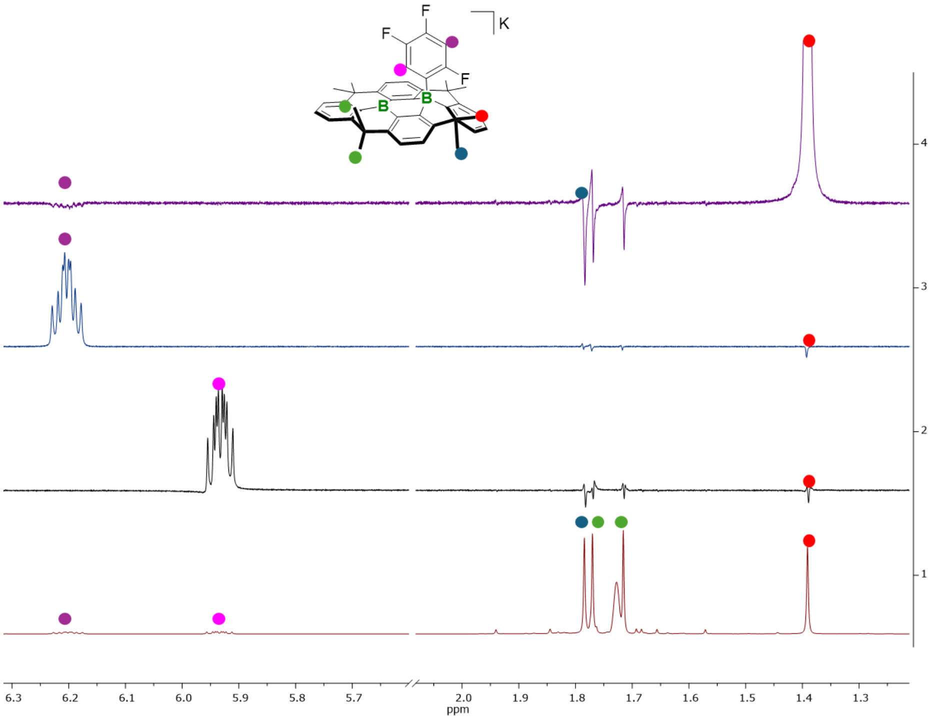

Figure S73: Expanded regions of the ^1H NMR and ^1H-^1H NOESY NMR spectra (500.2 MHz, THF-d_8) of K[**6**]. Bottom: ^1H NMR spectrum of K[**6**]. Black and blue spectrum: excitation of a proton resonance of the $C_6F_3H_2$ substituent (black spectrum: 5.96 – 5.91, pink dot ≙ H_2; blue spectrum: 6.23 – 6.17 ppm, purple dot ≙ H_5) shows that the signal at 1.39 ppm (red) can be assigned to the C*Me* groups positioned on the same side as the substituent. The signals within this spectrum, which exhibit both positive and negative intensities, can be attributed to artifacts of the NOESY NMR spectrum and/or exchange phenomena. Consequently, only the C*Me* resonance marked with a red dot represents a genuine interaction with the $C_6F_3H_2$ substituent. Top: Selective excitation of the C*Me* groups resonating at 1.39 ppm (red) shows an interaction with the respective C*Me* groups resonating at 1.78 ppm (blue), where the red- and blue-marked pair is bonded to the same C atom. Additionally, interactions with a proton of the $C_6F_3H_2$ substituent (6.23 – 6.17 ppm, purple ≙ H_5) are observed.

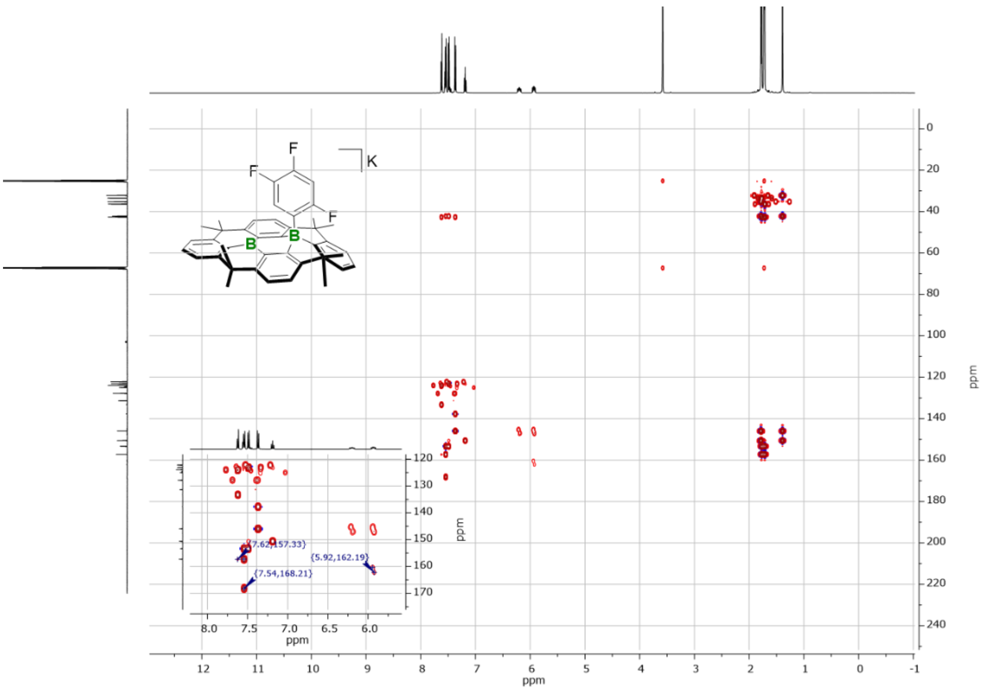

Figure S74: ^{1}H-^{13}C HMBC NMR spectrum (THF-d_8) of K[**6**].

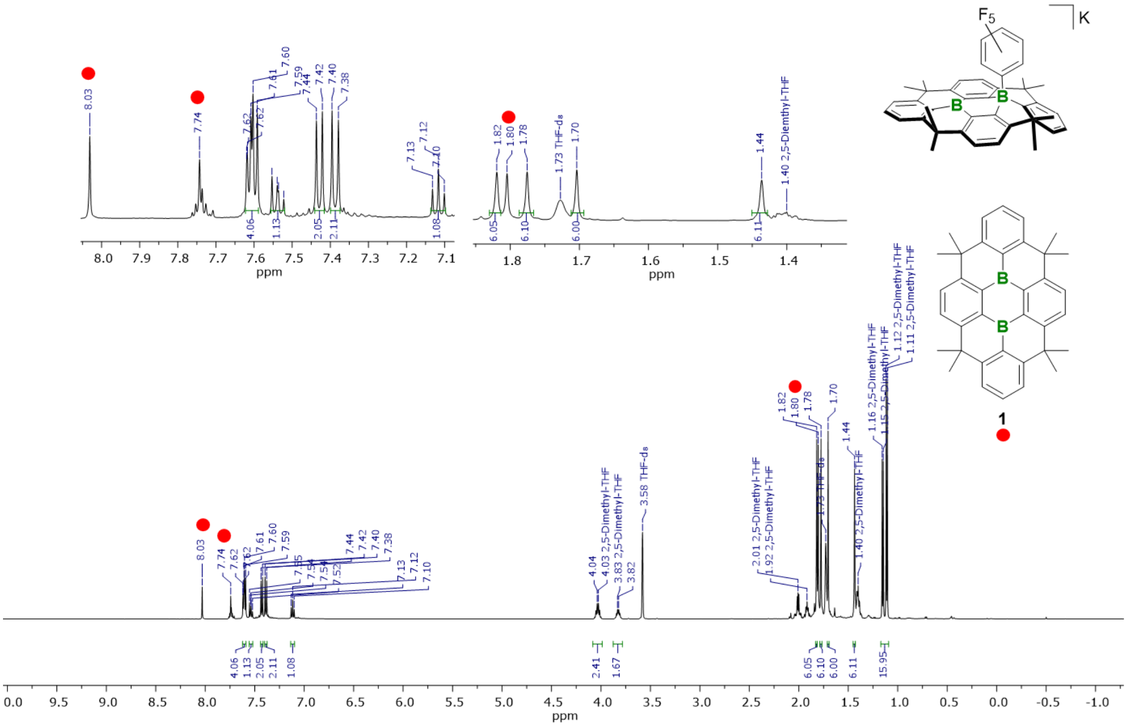

Figure S75: ^{1}H-NMR spectrum (500.2 MHz, THF-d_8) of K[**4**]·(2,5-Me$_2$-THF)$_{2.5}$. Contaminant: compound **1**.

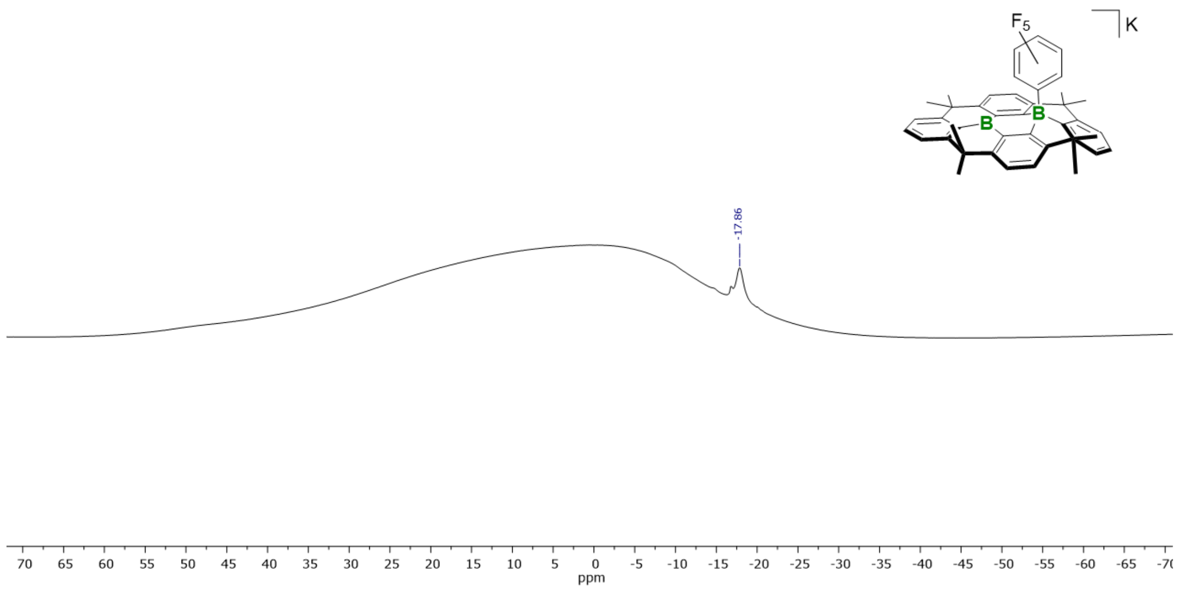

Figure S76: ^{11}B NMR spectrum (160.5 MHz, THF-d_8) of K[**4**]·(2,5-Me$_2$-THF)$_{2.5}$.

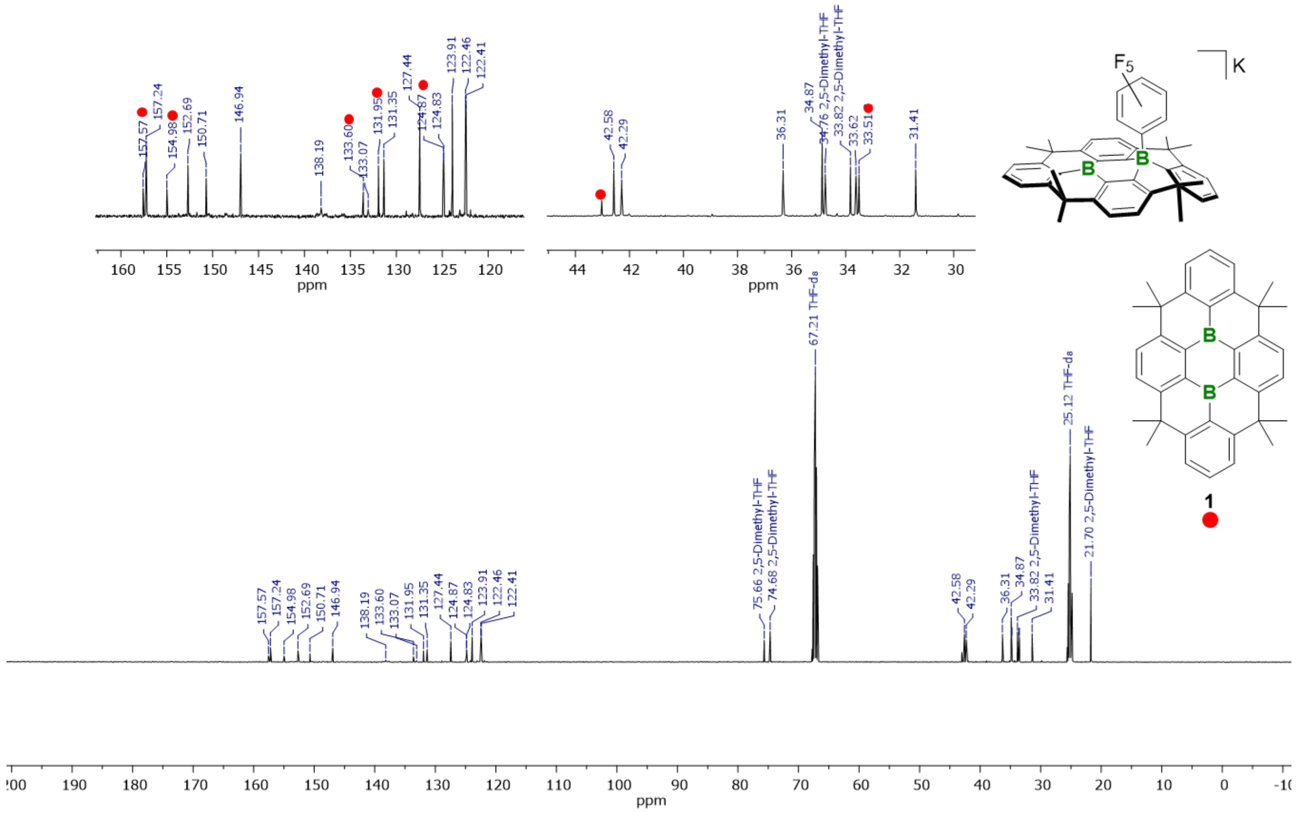

Figure S77: ^{13}C{^{1}H} NMR spectrum (125.8 MHz, THF-d_8) of K[**4**]·(2,5-Me$_2$-THF)$_{2.5}$. Contaminant: compound **1** (red dots).

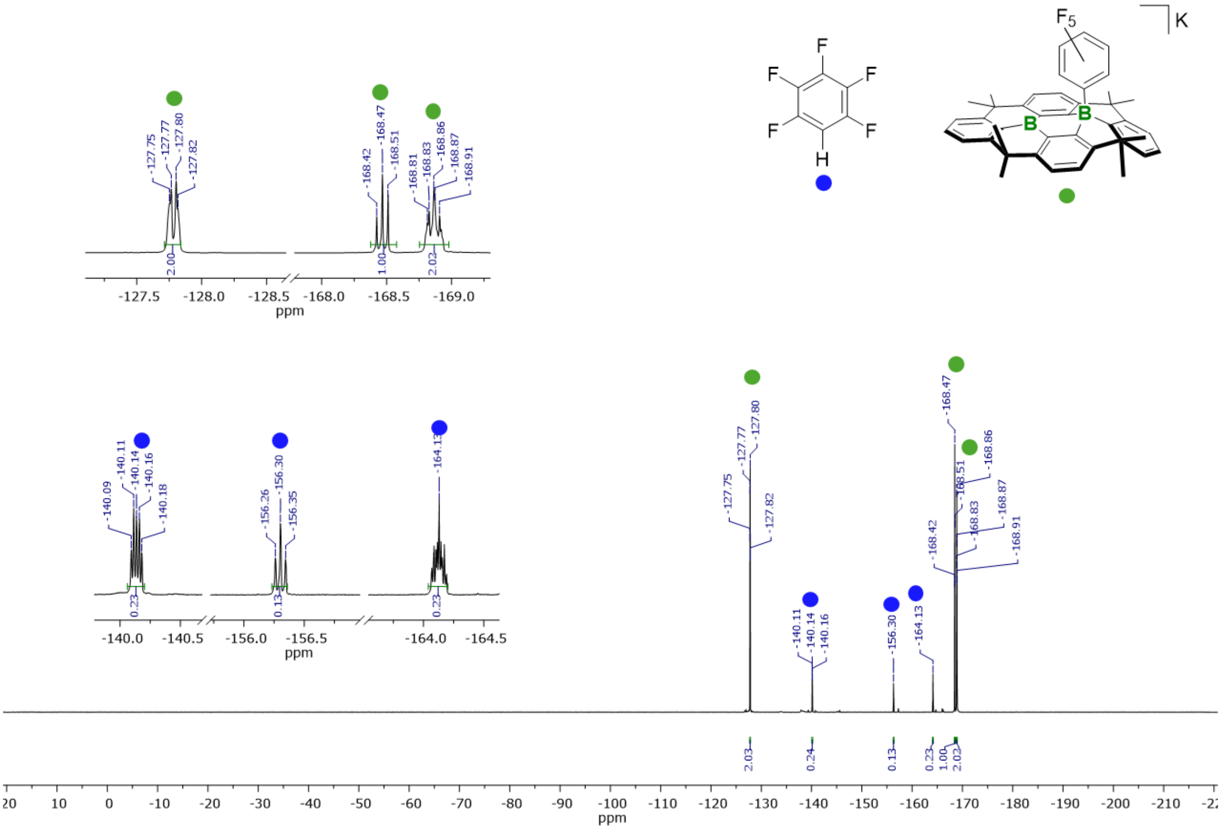

Figure S78: ^{19}F NMR spectrum (470.6 MHz, THF-d_8) of K[**4**]·(2,5-Me_2-THF)$_{2.5}$. Contaminant: C_6F_5H (blue dots).

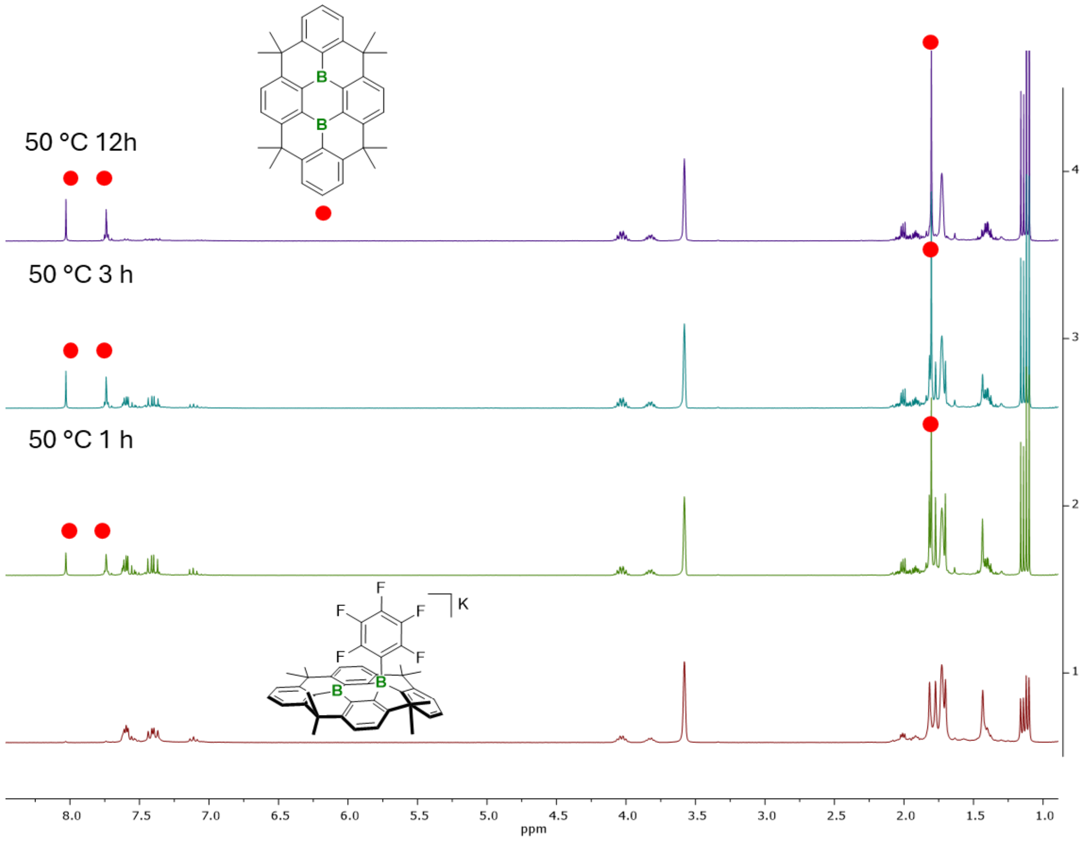

Figure S79: ^{1}H NMR spectra (300.0 MHz, THF-d_8) of K[**4**]·(2,5-Me_2-THF)$_{2.5}$ before (bottom) and after heating to 50 °C for 1 h (green spectrum), 3 h (blue spectrum) and 12 h (purple spectrum).

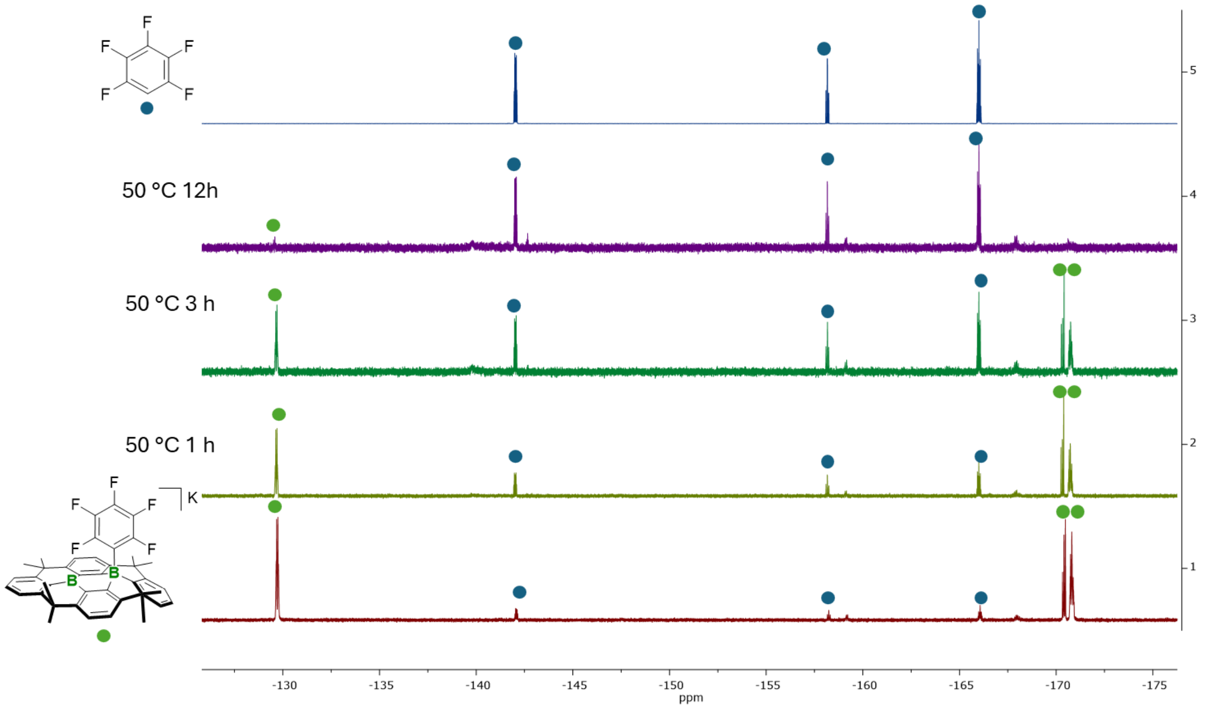

Figure S80: ^{19}F NMR spectra (282.3 MHz, THF-d_8) of K[**4**]·(2,5-Me_2-THF)$_{2.5}$ (bottom) and after heating to 50 °C for 1 h (light green spectrum), 3 h (dark green spectrum) and 12 h (purple spectrum). Top: Authentic sample of C_6F_5H.

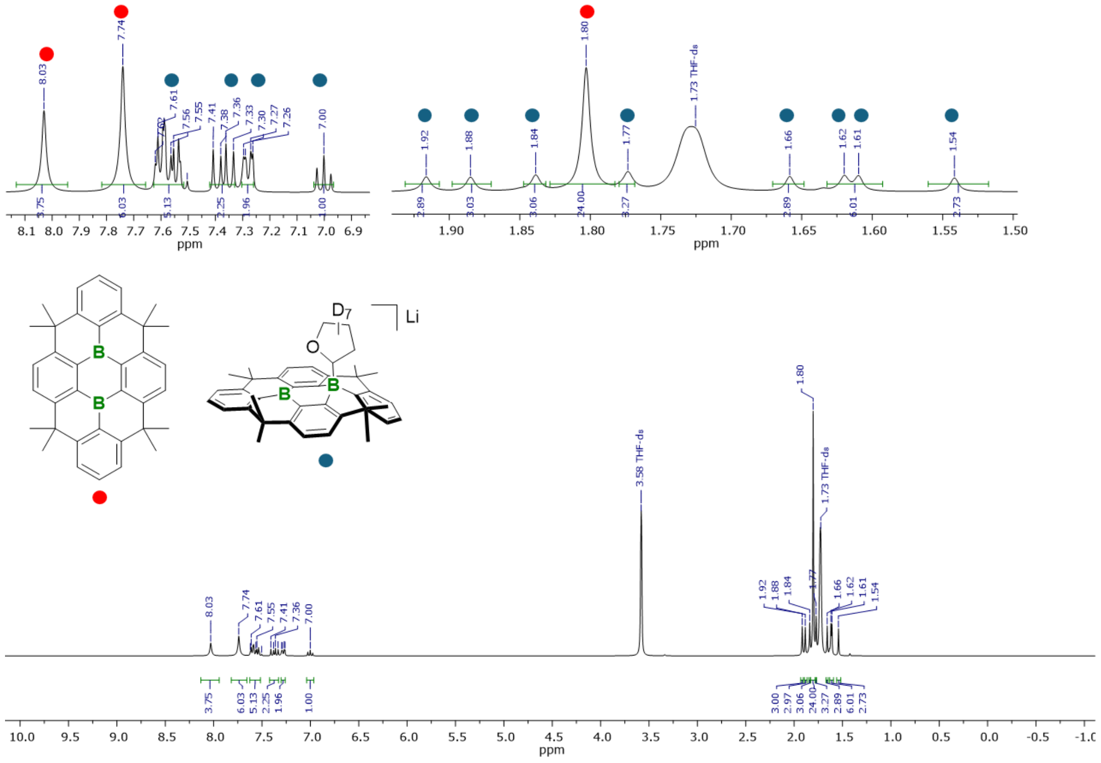

Figure S81: ^{1}H NMR spectrum (300.0 MHz, THF-d_8) of the reaction mixture of Li[**1**] with C_6F_6.

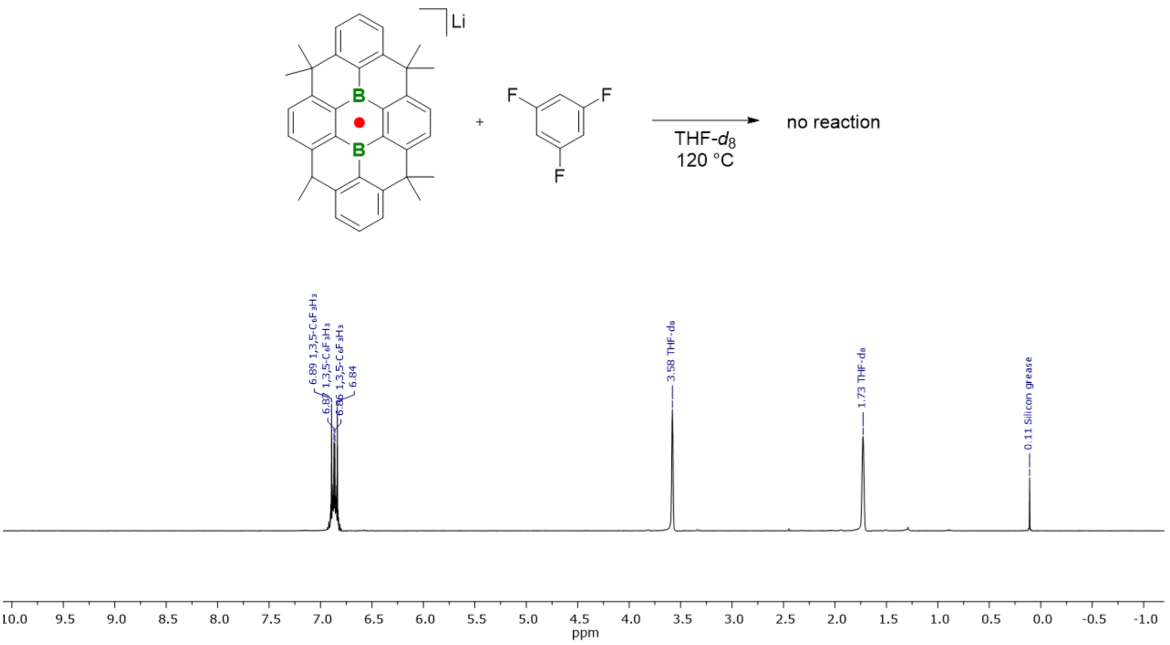

Figure S82: The ^{1}H NMR spectrum (300.0 MHz, THF-d_8) of Li[**1**] and 1,3,5-$C_6F_3H_3$ after heating to 120 °C still shows only the NMR signals of 1,3,5-$C_6F_3H_3$ and of the solvent, indicating Li[**1**] (an NMR silent radical) did not react with 1,3,5-$C_6F_3H_3$.

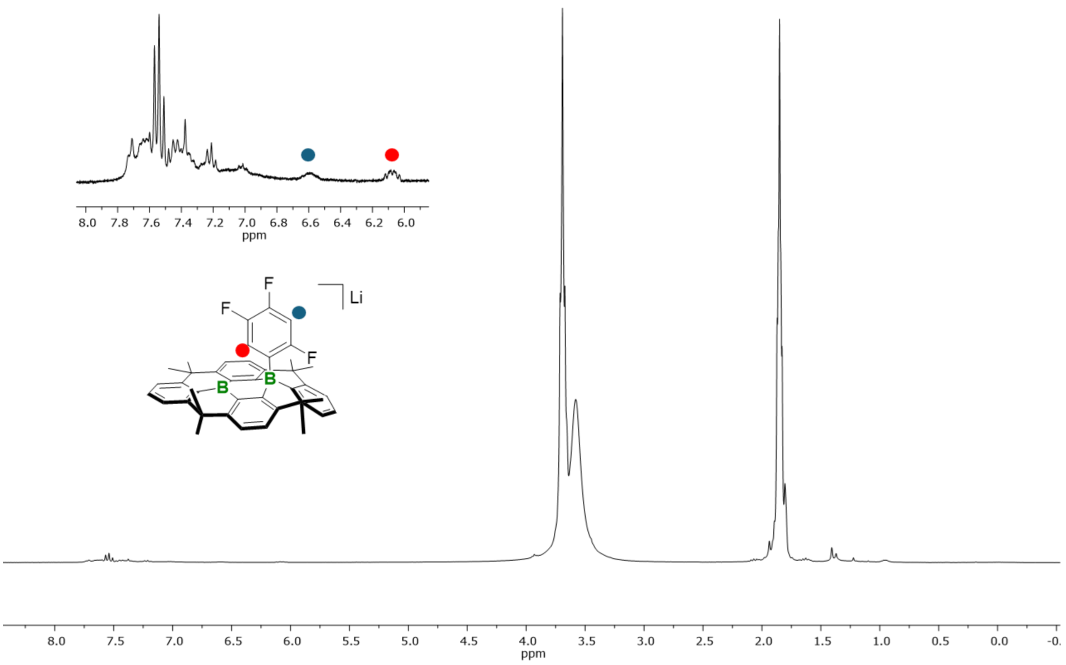

Figure S83: ^{1}H NMR spectrum (300.0 MHz, THF-d_8) of the crude reaction mixture of Li_2[**1**] with 1,2,4,5-$C_6F_4H_2$ in the presence of 12-c-4.

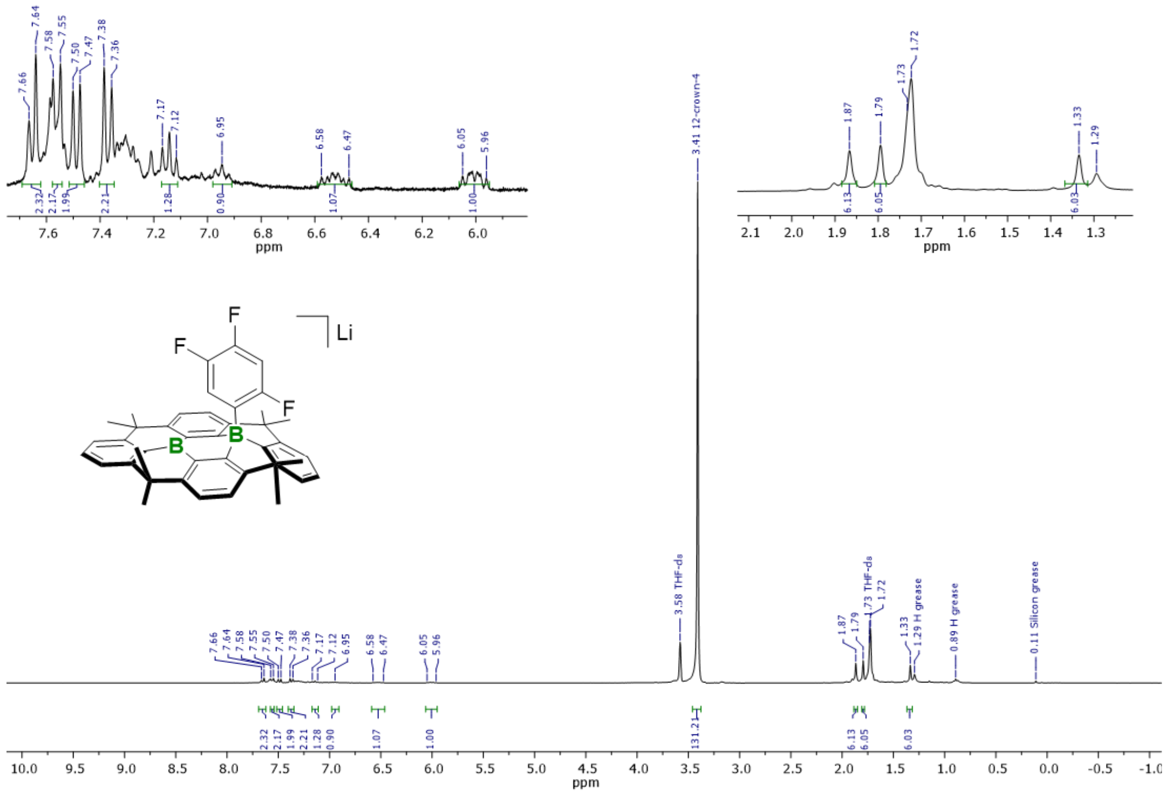

Figure S84: ^{1}H NMR spectrum (300.0 MHz, THF-d_8) of the reaction mixture of Li_2[**1**] with 1,2,4,5-$C_6F_4H_2$ in the presence of 12-c-4 after removing volatile components under reduced pressure.

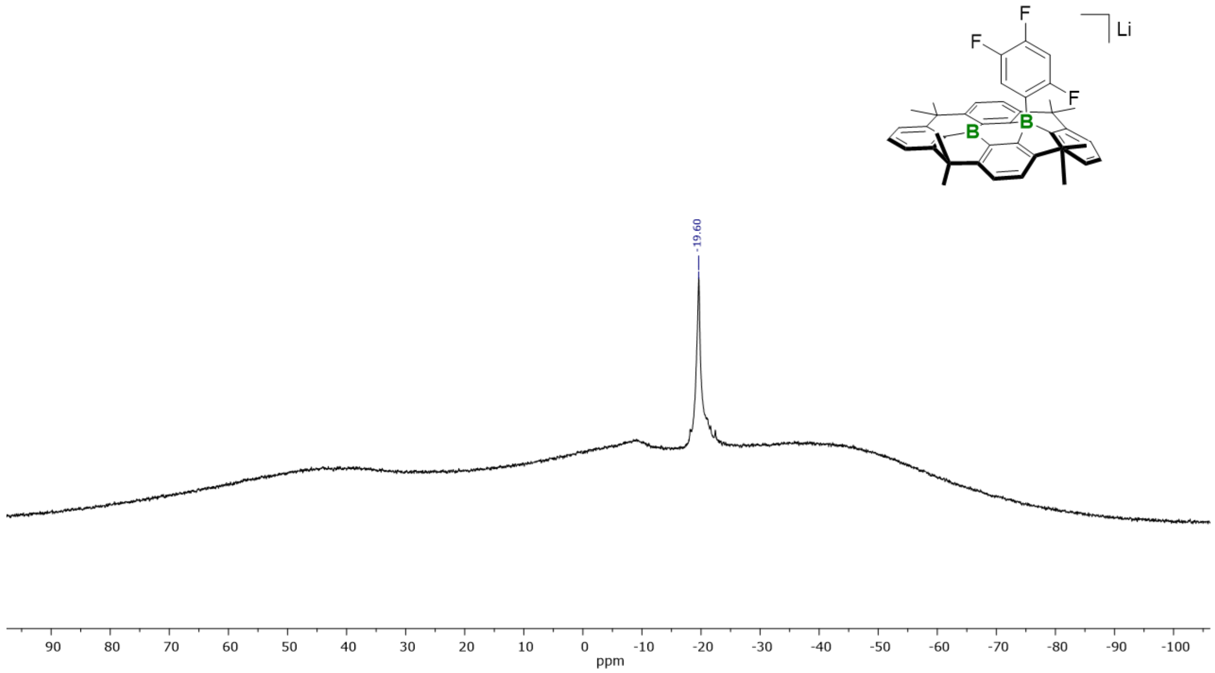

Figure S85: ^{11}B NMR spectrum (96.3 MHz, THF-d_8) of the reaction mixture of Li_2[**1**] with 1,2,4,5-$C_6F_4H_2$ in the presence of 12-c-4.

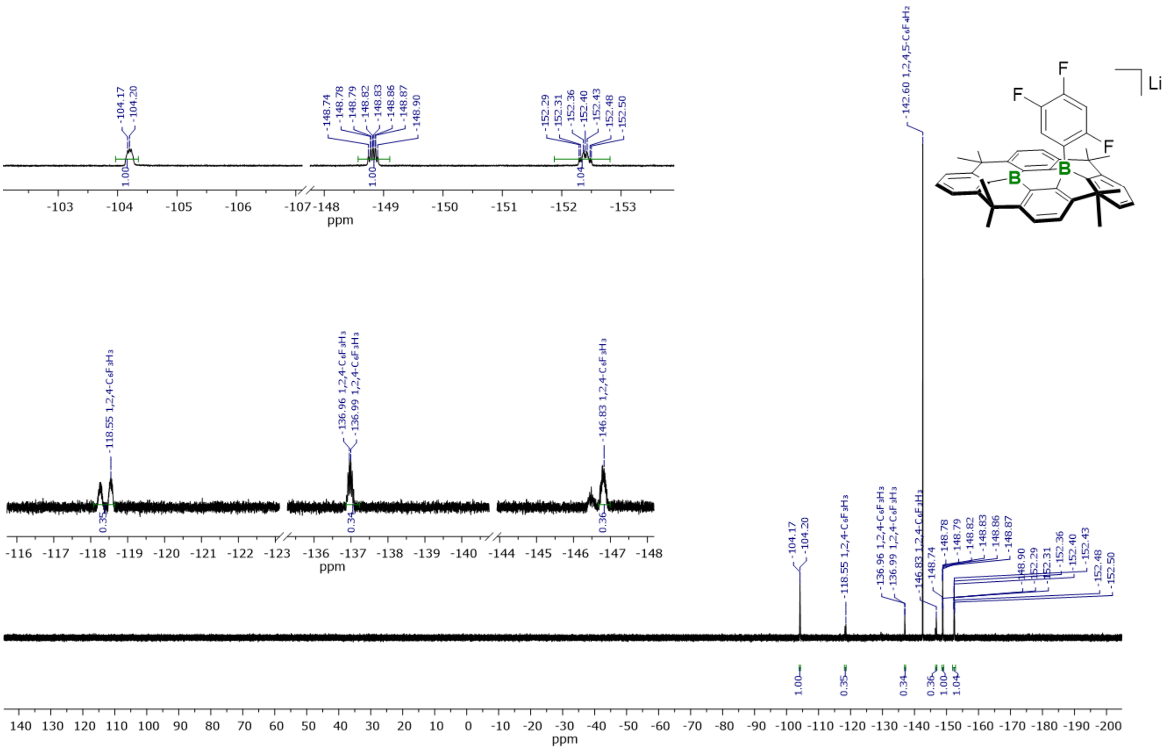

Figure S86: ^{19}F NMR spectrum (282.3 MHz, THF-d_8) of the reaction mixture of Li_2[**1**] with 1,2,4,5-$C_6F_4H_2$ in the presence of 12-c-4. Contaminant: 1,2,4-$C_6F_3H_3$ and 1,2,4-F_3-5-D-C_6H_2.

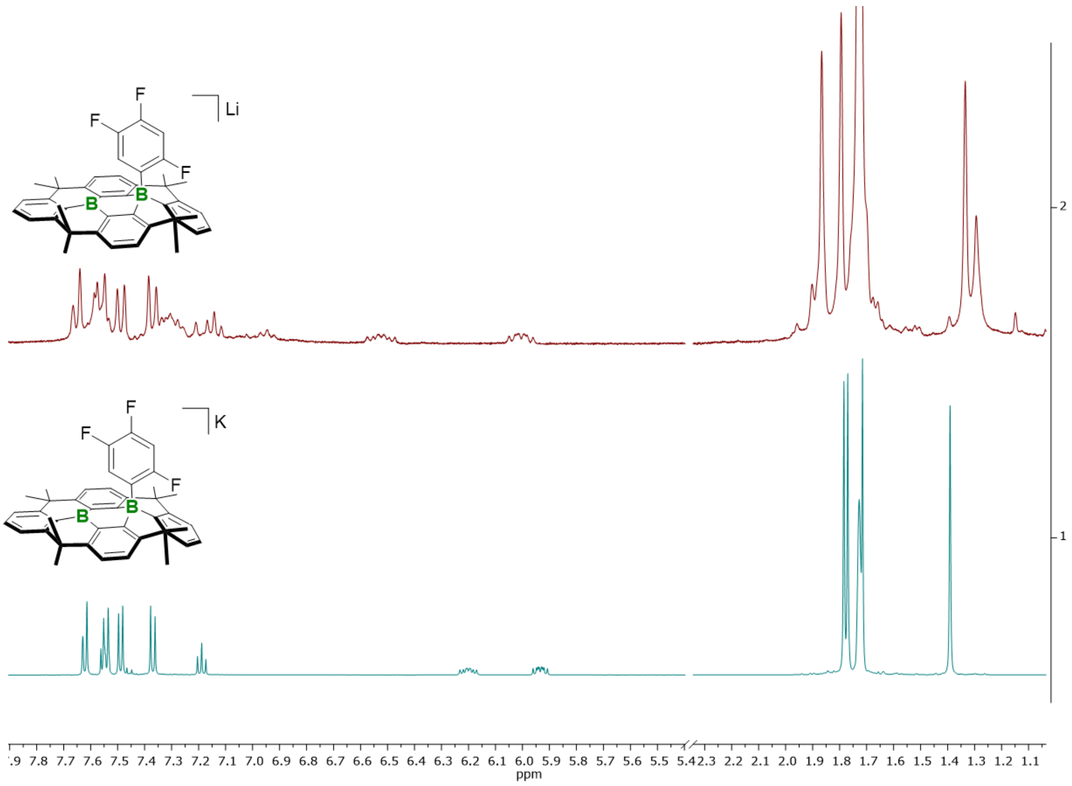

Figure S87: Expanded regions of ^{1}H NMR spectra (300.0 MHz, THF-d_8) of K[**6**] (bottom) and Li[**6**]·(12-c-4)$_8$ (top). The comparison of the NMR spectra confirms the structural identity of both compounds.

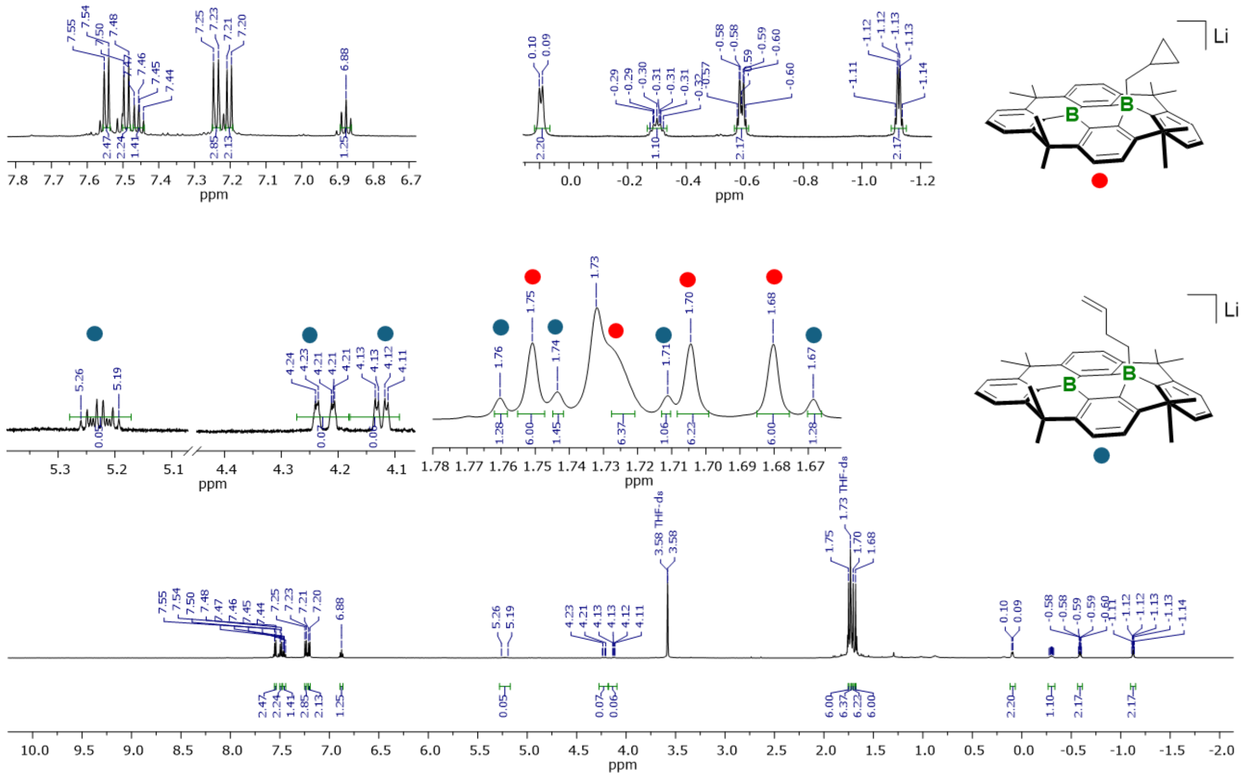

Figure S88: The ^{1}H NMR spectrum (600.0 MHz, THF-d_8) of the reaction mixture of Li_2[**1**] with (bromomethyl)cyclopropane shows the formation of Li[**7**] (red dots) as the main product (85 %). Li[**8**] (blue dots) formed as a side product in this reaction (15 %) and could not be separated by crystallization.

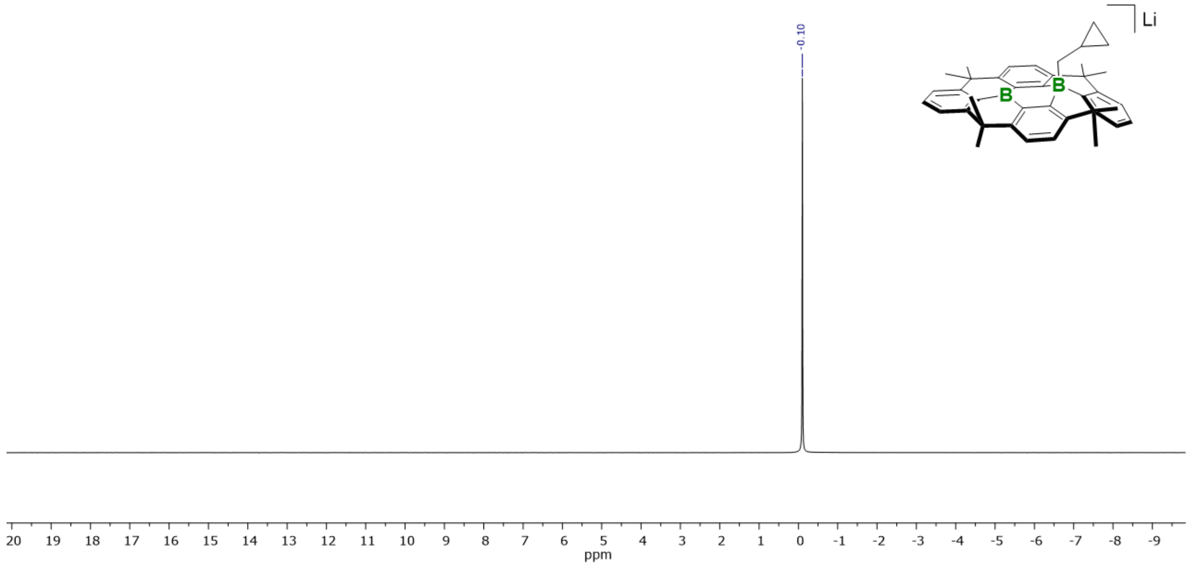

Figure S89: ^{7}Li NMR spectrum (194.4 MHz, THF-d_8) of the reaction mixture of Li_2[**1**] with (bromomethyl)cyclopropane.

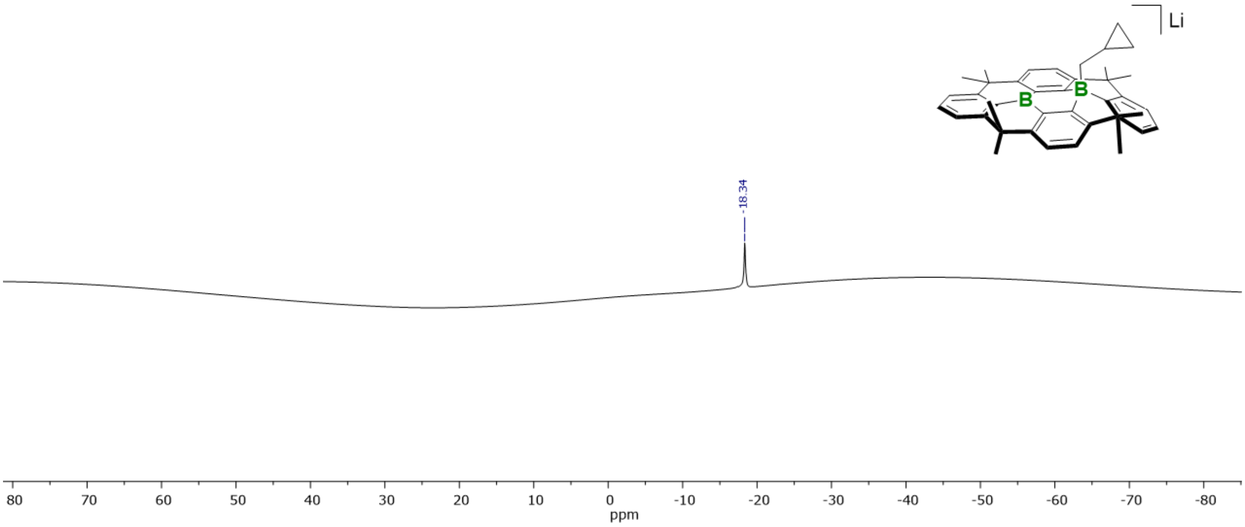

Figure S90: ^{11}B NMR spectrum (160.5 MHz, THF-d_8) of the reaction mixture of Li_2[**1**] with (bromomethyl)cyclopropane.

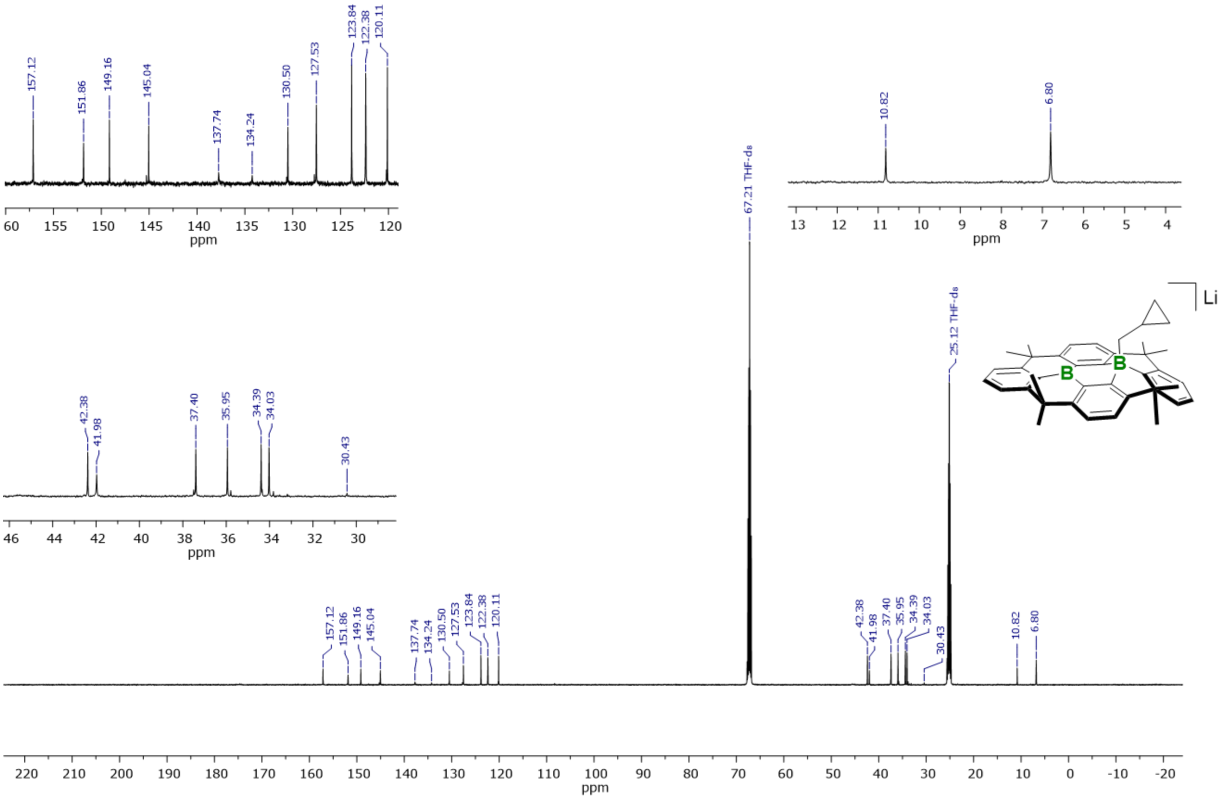

Figure S91: ^{13}C{^{1}H} NMR spectrum (125.8 MHz, THF-d_8) of the reaction mixture of Li_2[**1**] with (bromomethyl)cyclopropane.

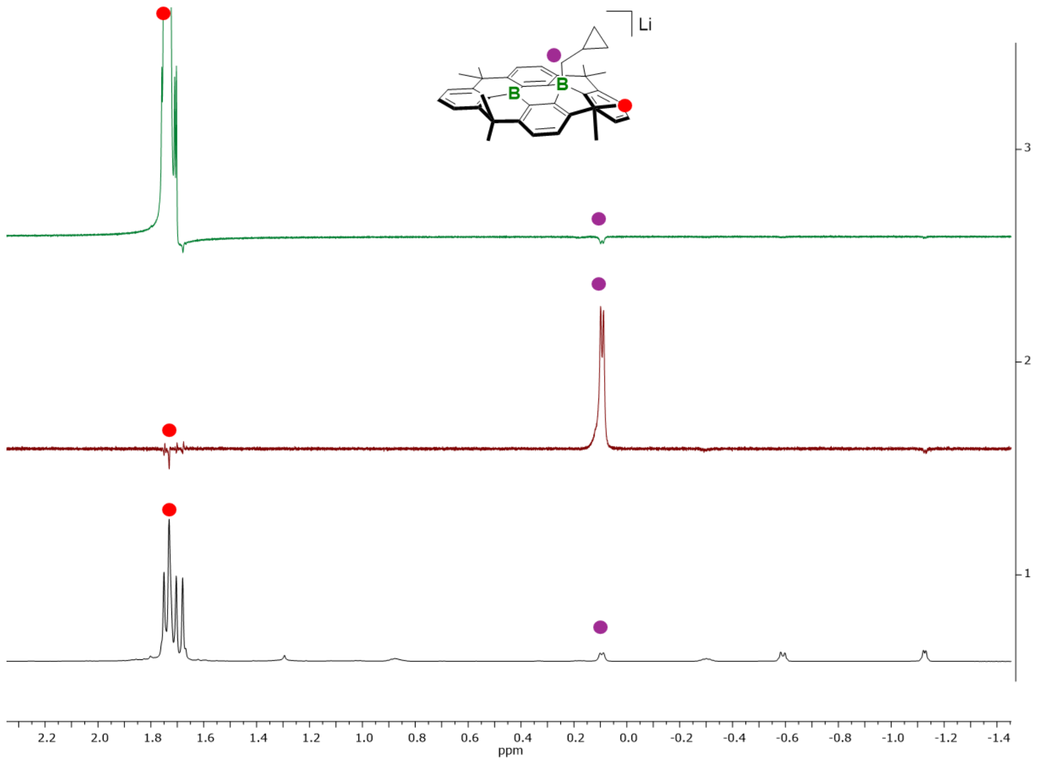

Figure S92: Expanded regions of the ^{1}H NMR and ^{1}H-^{1}H NOESY NMR spectra (500.2 MHz, THF-d_8) of Li[**7**]. Bottom: ^{1}H NMR spectrum of Li[**7**]. Middle: Selective excitation of the B-bonded CH_2 group (0.10 ppm; purple dot) shows that the signal at 1.73 (red dot) can be assigned to the adjacent Me groups situated on the same side as the substituent. Top: Excitation of the Me groups at 1.73 ppm (red dot) shows an interaction with the CH_2 group of the substituent (0.10 ppm; purple dot).

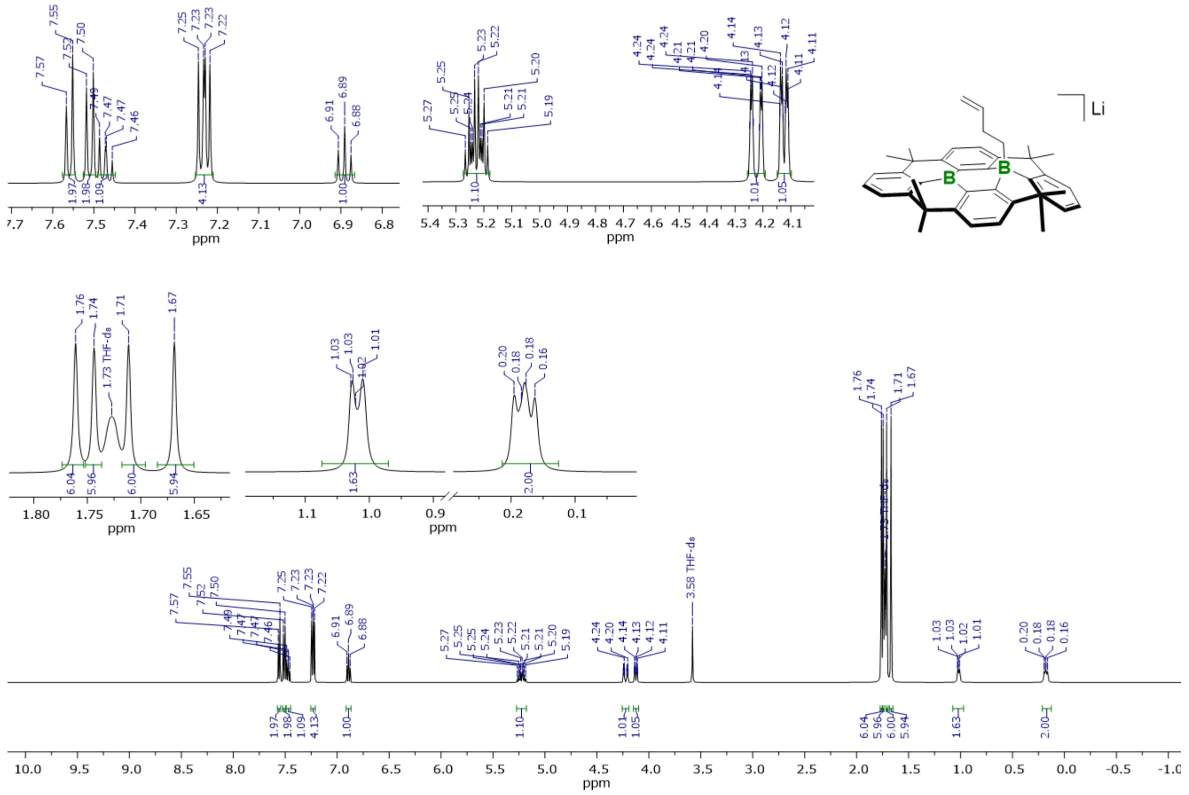

Figure S93: ^{1}H NMR spectrum (500.2 MHz, THF-d_8) of Li[**8**].

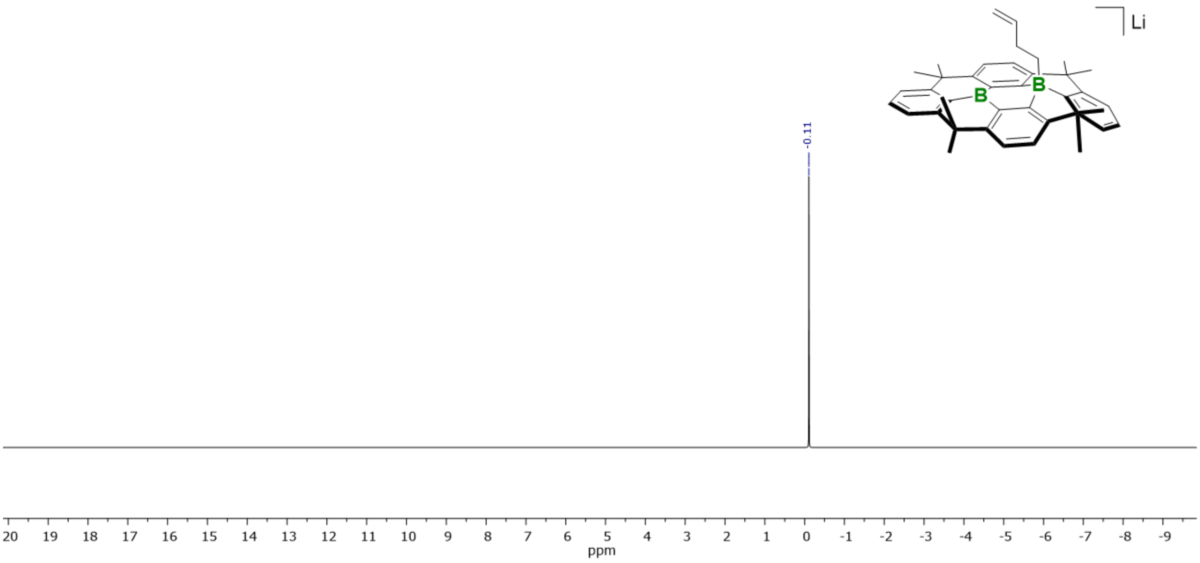

Figure S94: ^{7}Li NMR spectrum (194.4 MHz, THF-d_8) of Li[**8**].

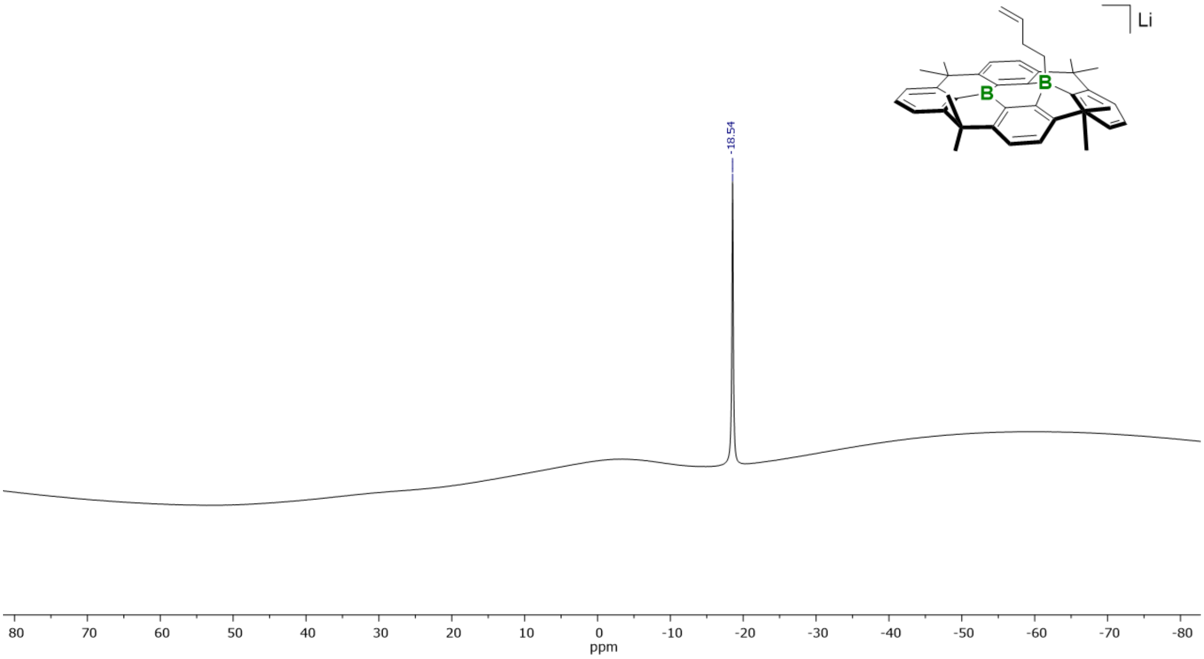

Figure S95: ^{11}B NMR spectrum (160.5 MHz, THF-d_8) of Li[**8**].

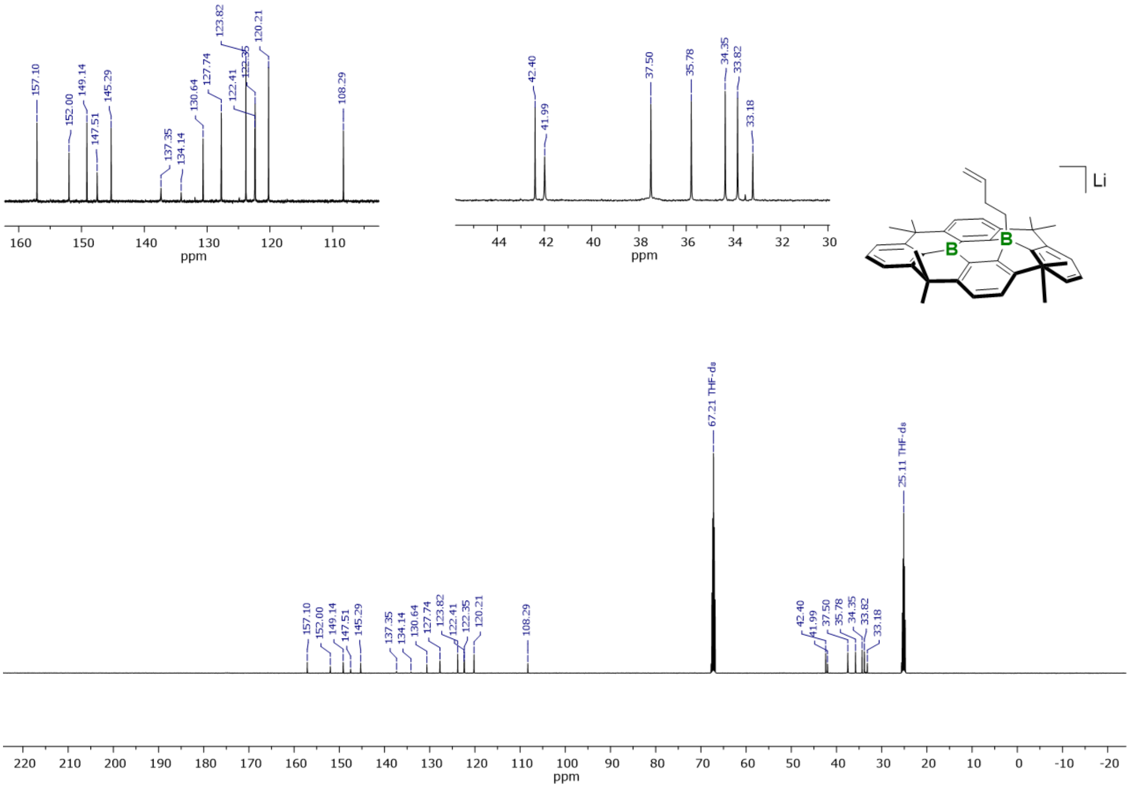

Figure S96: $^{13}C\{^{1}H\}$ NMR spectrum (125.8 MHz, THF-d_8) of Li[**8**].

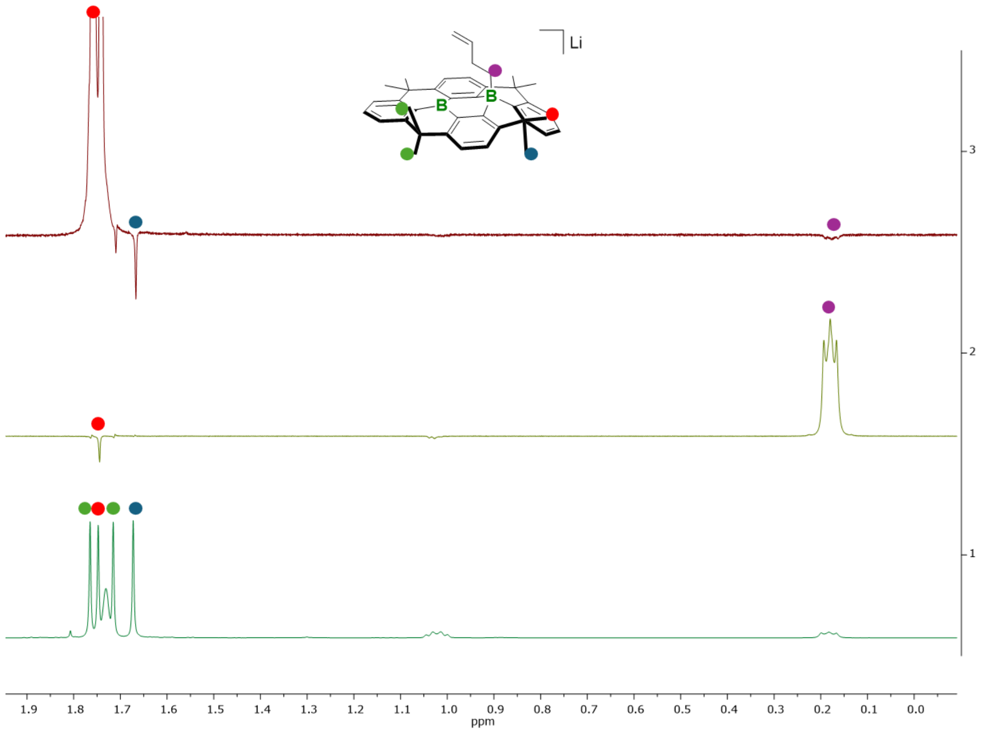

Figure S97: Expanded regions of the ^{1}H NMR and ^{1}H-^{1}H NOESY NMR spectra (500.2 MHz, THF-d_8) of Li[**8**]. Bottom: ^{1}H NMR spectrum of Li[**8**]. Middle: Selective excitation of the B-bonded CH_2 group (0.20-0.16 ppm, purple dot) shows that the signal at 1.74 ppm (red dot) can be assigned to the adjacent Me groups that are situated on the same side as the substituent. Top: Excitation of the Me groups resonating at 1.74 ppm (red dot) shows an interaction with the CH_2 group of the substituent (0.20-0.16 ppm; purple dot). Additionally, an interaction with the C*Me* groups resonating at 1.67 ppm is observed, indicating that the red- and blue-marked pair is bonded to the same C atom.

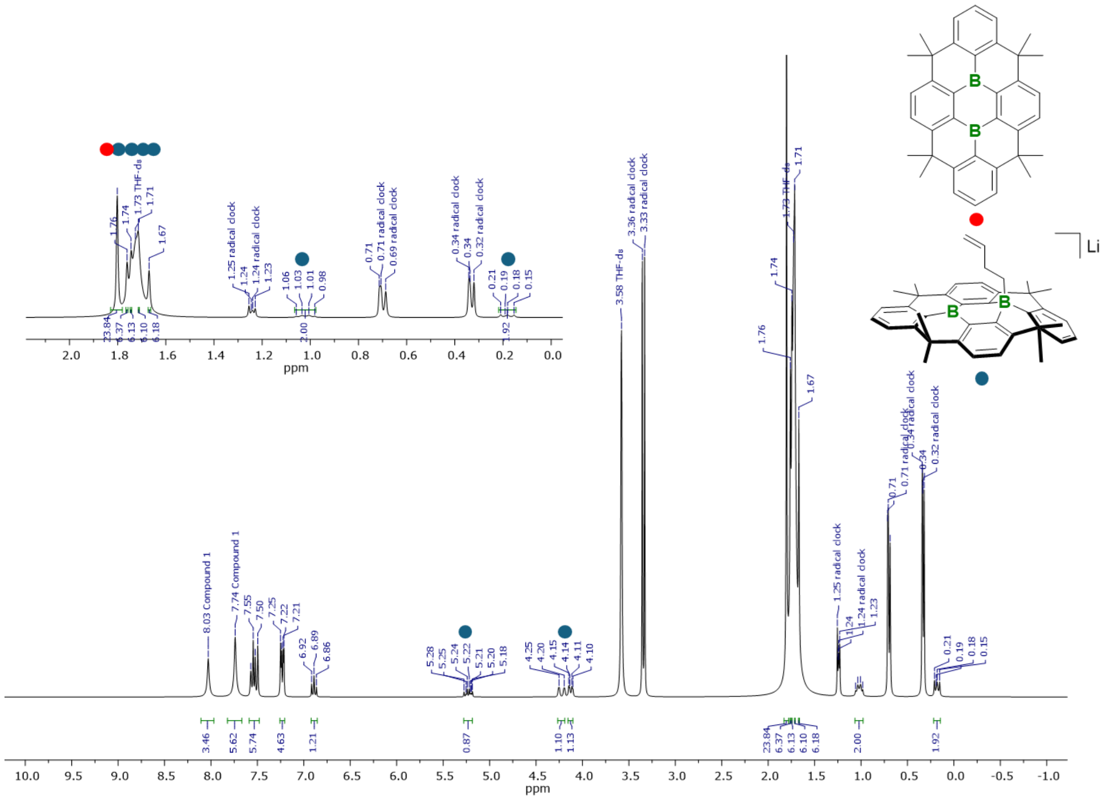

Figure S98: ^{1}H NMR spectrum (300.0 MHz, THF-d_8) of the reaction mixture of Li[**1**] with (bromomethyl)cyclopropane.

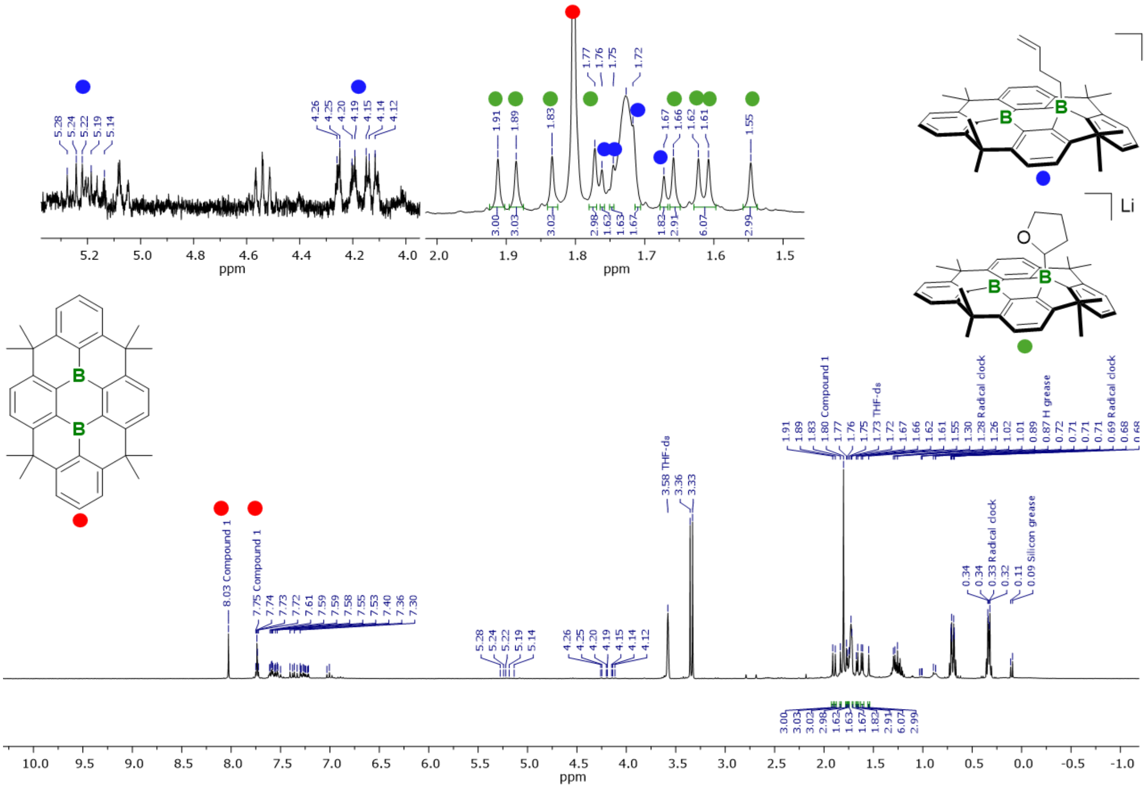

Figure S99: ^{1}H NMR spectrum (300.0 MHz, THF-d_8) of the reaction mixture of Li_2[**1**] with C_6F_6 and (bromomethyl)cyclopropane.

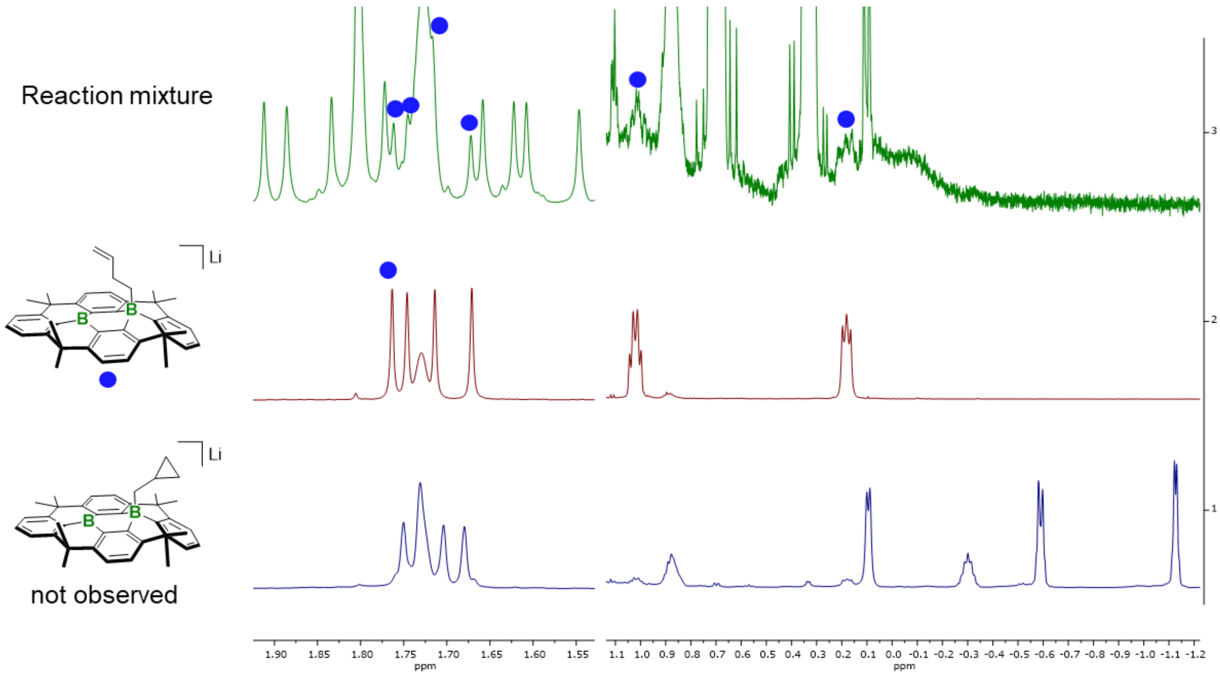

Figure S100: Comparison of the alkyl regions of the ^{1}H NMR spectra (500.0 MHz (bottom and middle), 300.0 MHz (top); THF-d_8) of Li[**7**] (bottom), Li[**8**] (middle), and of the reaction mixture Li_2[**1**]/C_6F_6/(bromomethyl)cyclopropane (top). This comparison demonstrates that Li[**7**] is not observed in the reaction mixture, suggesting that radicals are involved in the reaction.

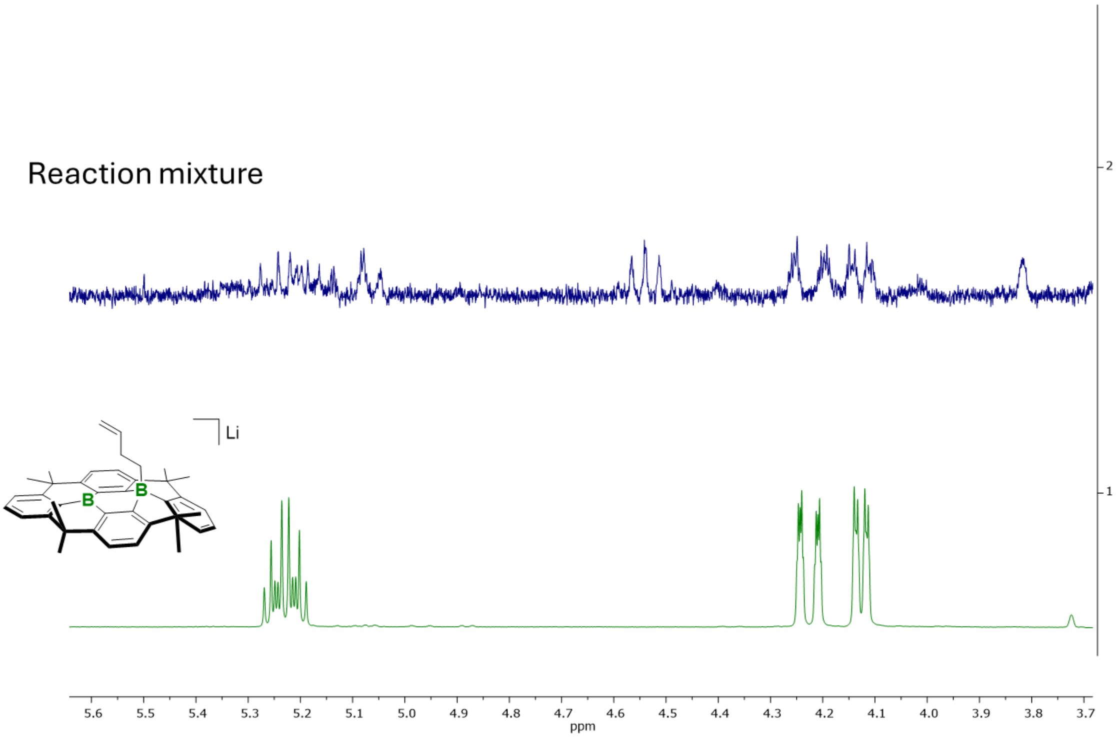

Figure S101: Comparison of the allyl regions of the ^{1}H NMR spectra (500.0 MHz (bottom), 300.0 MHz (top); THF-d_8) of Li[**8**] (bottom) and of the reaction mixture of Li_2[**1**]/C_6F_6/(bromomethyl)cyclopropane (top).

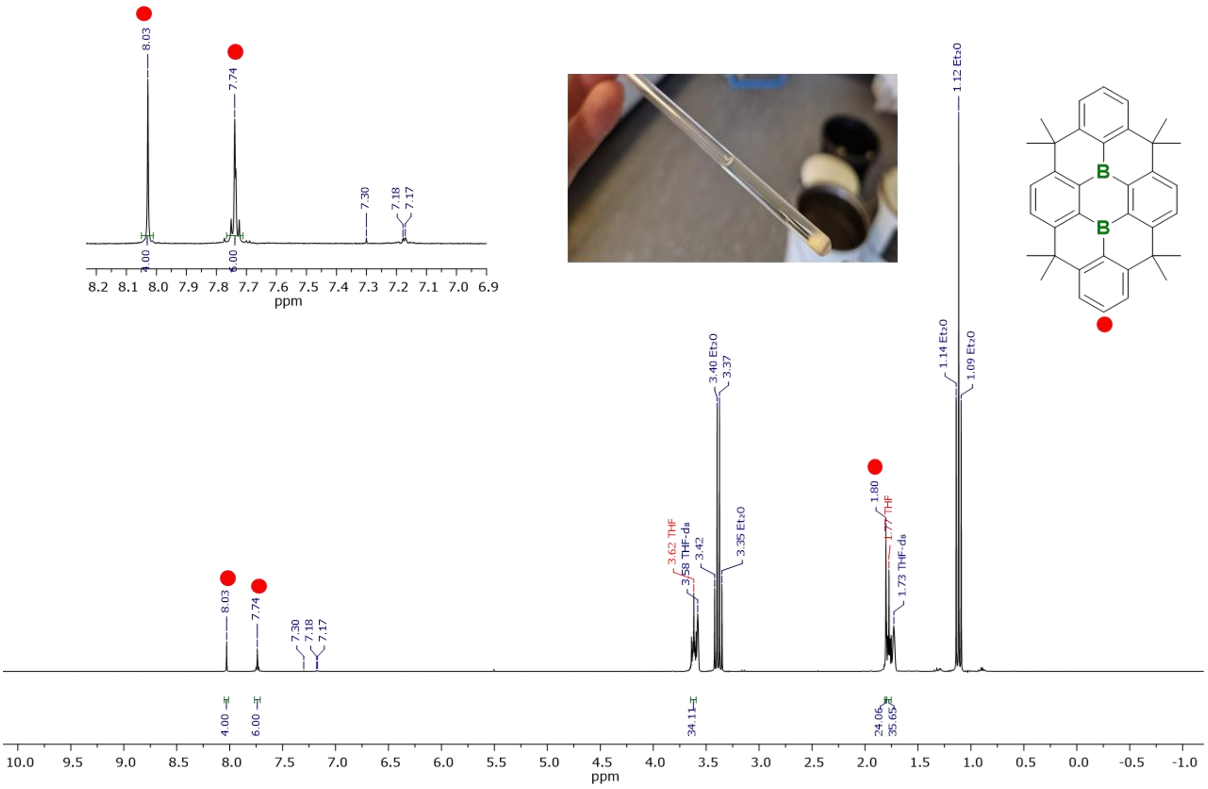

Figure S102: ^{1}H NMR spectrum (300.0 MHz, THF-d_8) of the reaction mixture of Li[**3**] with ethereal HCl.

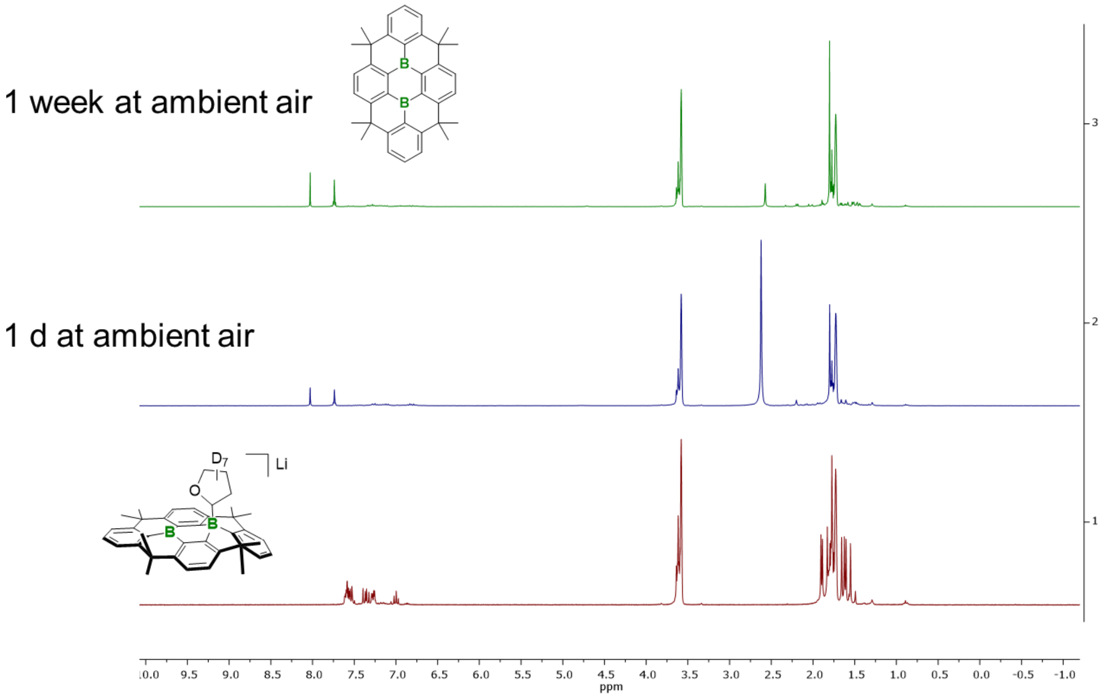

Figure S103: ^{1}H NMR spectra (300.0 MHz, THF-d_8) of Li[**3**^{D}] (bottom), of Li[**3**^{D}] after exposure to air for 1 d (middle), and of Li[**3**^{D}] after exposure to air for one week (top).

3 General procedure for UV-vis experiments

Upon visual observation of the reaction between Li_2[**1**] or K_2[**1**] and C_6F_6, a distinct color change was immediately apparent: green to blue to colorless for Li_2[**1**], and purple to blue to colorless for K_2[**1**].

Given that the blue color is a characteristic feature of the radical anion $[\mathbf{1}]^{\bullet-}$, C−F activation was performed with simultaneous monitoring the reaction mixture using UV-vis spectroscopy to confirm the presence of the radical anion $[\mathbf{1}]^{\bullet-}$ as a reaction intermediate.

The THF used for UV-vis spectroscopy required special purification: When the THF was dried over Na/benzophenone and then distilled, a color change was observed in the solutions of Li_2[**1**] and K_2[**1**] in the cuvette within minutes, even in the absence of C_6F_6. This phenomenon is attributable to trace amounts of residual benzophenone and/or the stabilizing agent dibutylhydroxytoluene (BHT). Due to the high absorbance of the dianions, highly diluted solutions were required to record the UV-vis spectra. The molar concentrations in these solutions were so low that even trace amounts of benzophenone or BHT were sufficient to oxidize the dianions to a significant extent, resulting in the formation of the radical anion $[\mathbf{1}]^{\bullet-}$. To avoid this issue, THF was distilled over an extended Vigreux column, then dried over Na/K alloy, and re-distilled. The purified THF was then stored over Na/K alloy, where a slight blue coloration was observed, prompting an additional distillation step. Finally, the THF was degassed through four freeze-pump cycles. UV-vis measurements performed with this rigorously purified THF enabled the acquisition of clean spectra of the dianion salts Li_2[**1**] and K_2[**1**], without detectable contamination by Li[**1**] or K[**1**]. To complement these findings, also the radical anion salts Li[**1**] and K[**1**] were freshly prepared, allowing for the collection of their authentic spectra in THF, too.

Since the UV-vis spectral differences between K[**1**] and K_2[**1**] are more pronounced than those between Li[**1**] and Li_2[**1**] (Figures S105 and S106), reaction monitoring with C_6F_6 was performed exclusively with the K^+ salts, which exhibit the same reactivity toward C_6F_6 as the Li^+ salts.

Following the addition of C_6F_6 to K_2[**1**] in THF, a delay of approximately 30−40 s was unavoidable during the transfer of the cuvette containing the reaction mixture from the glovebox to the UV-vis spectrometer. Consequently, progress of the reactions could only be monitored from this point in time.

3.1 Procedure for recording UV-vis spectra of the compounds Li_2[**1**], Li[**1**], K_2[**1**], and K[**1**]

Li_2[**1**], Li[**1**], K_2[**1**], and K[**1**] were prepared by following the previously described protocols. The quantities of **1** used for each reduction, along with the corresponding solvent volumes and molar amounts, are compiled in Table S10. An aliquot of each solution was further diluted with an additional 3 mL of THF to prepare the final sample for investigation.

Table S10: Quantities used for the preparation of samples of compounds Li_2[**1**], Li[**1**], K_2[**1**], and K[**1**] for UV-vis measurements.

**Compound**	**Amount of 1**	**Volume of THF used for reduction**	**Volume used for sample preparation**	**Final concentration of the sample**
Li_2[**1**]	1.0 mg, 2.1 µmol	0.5 mL	25 µL	35 µmol/L
Li[**1**]	2.2 mg, 4.5 µmol	1 mL	60 µL	90 µmol/L
K_2[**1**]	1.0 mg, 2.1 µmol	0.5 mL	25 µL	35 µmol/L
K[**1**]	2.0 mg, 4.1 µmol	1 mL	60 µL	80 µmol/L

The spectra obtained were normalized to an OD of 1 for the respective maximum. (see Figures S104, S105 and S106).

3.2 Procedure for reaction monitoring of K_2[**1**] and C_6F_6 by UV-vis spectroscopy

1 (1.0 mg, 2.1 µmol) was reduced to K_2[**1**] in THF (0.5 mL) in a glovebox using the previously described method. 25 µL of the respective deep purple solution was transferred to a cuvette and THF (3 mL) was added. The cuvette was sealed airtight and wrapped with parafilm. A UV-vis spectrum was recorded on this sample of K_2[**1**]. The cuvette was then returned to the glovebox and a THF/C_6F_6 solution (0.1 mol/L, 1.2 µL, 1.1 eq.) was added. The cuvette was again sealed airtight and wrapped with parafilm. The progress of the reaction was monitored by recording a series of UV-vis spectra (Figure S107).

3.3 Procedure for reaction monitoring of K_2[**1**] and 1,2,4,5-$C_6F_4H_2$ by UV-vis spectroscopy

1 (1.0 mg, 2.1 µmol) was reduced to K_2[**1**] in THF (0.5 mL) in a glovebox using the previously described method. 25 µL of the respective deep purple solution was transferred to a cuvette and THF (3 mL) was added. The cuvette was sealed airtight and wrapped with parafilm. A UV-vis spectrum was recorded on this sample of K_2[**1**]. The cuvette was then returned to the glovebox and 1,2,4,5-$C_6F_4H_2$ (2.5 µL, exc.) was added. The cuvette was again sealed airtight and wrapped with parafilm. The progress of the reaction was monitored by recording a series of UV-vis spectra (Figure S108).
Note: A substantially larger excess of 1,2,4,5-$C_6F_4H_2$ was employed, as the reaction rate is significantly smaller compared to reactions involving C_6F_6 and the reaction would otherwise be too slow to monitor using UV-vis spectroscopy.

4 Plots of UV-vis spectra

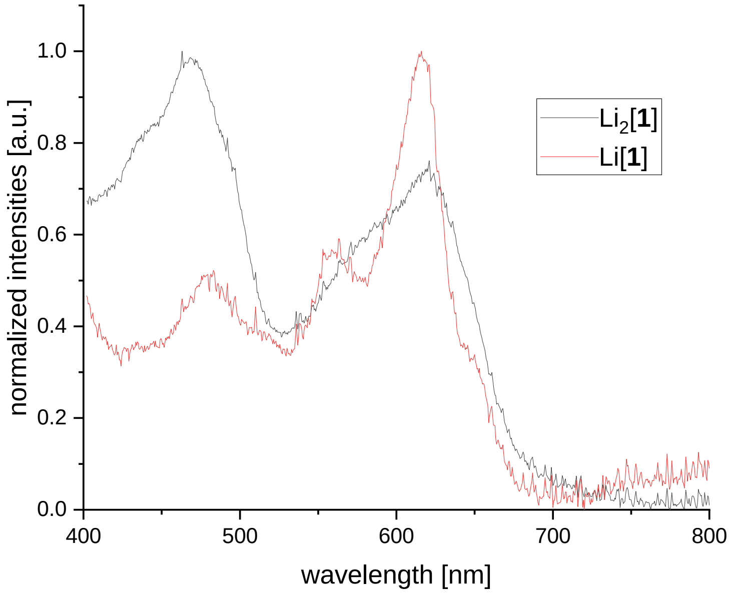

Figure S104: Normalized UV-vis absorption spectra of Li_2[**1**] (black) and Li[**1**] (red) in THF, recorded using a fiber-optic device inside an Ar-filled glovebox.

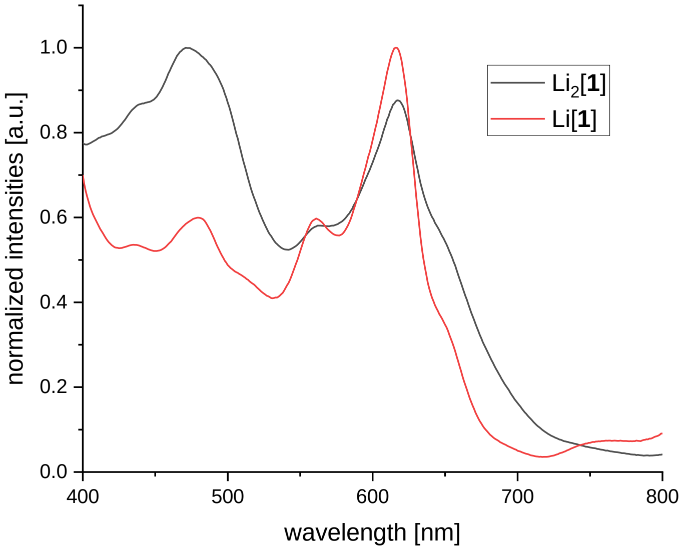

Figure S105: Normalized UV-vis absorption spectra of Li_2[**1**] (black) and Li[**1**] (red) in THF, recorded on samples inside airtight cuvettes outside the Ar-filled glovebox.

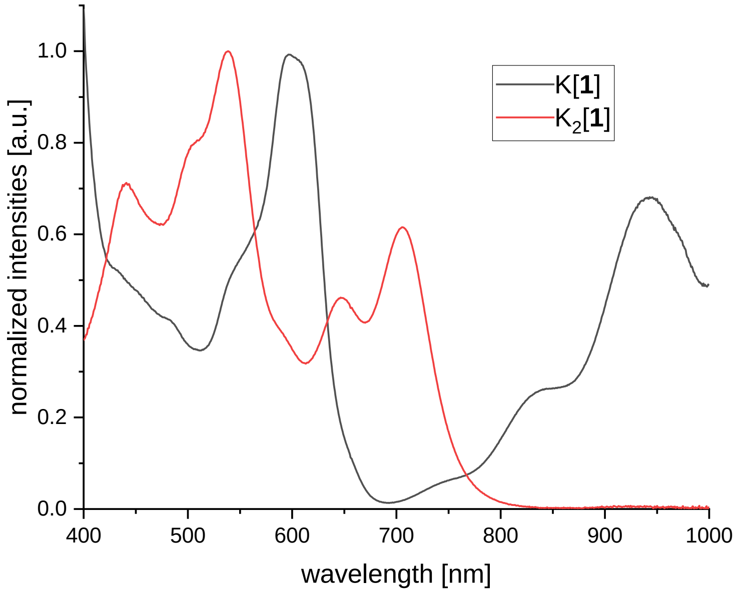

Figure S106: UV-vis absorption spectra of K[**1**] (black) and K_2[**1**] (red) in THF, recorded on samples inside airtight cuvettes outside the Ar-filled glovebox.

Table S11: Selected UV-vis absorption maxima of Li_2[**1**], Li[**1**] (400−800 nm); K_2[**1**], and K[**1**] (400−1000 nm) in THF.

Compound	λ^{abs}(THF) [nm]
Li_2[**1**]	438, 470, 618
Li[**1**]	435, 480, 560, 616
K_2[**1**]	440, 505, 540, 645, 705
K[**1**]	483, 598, 610, 840, 940

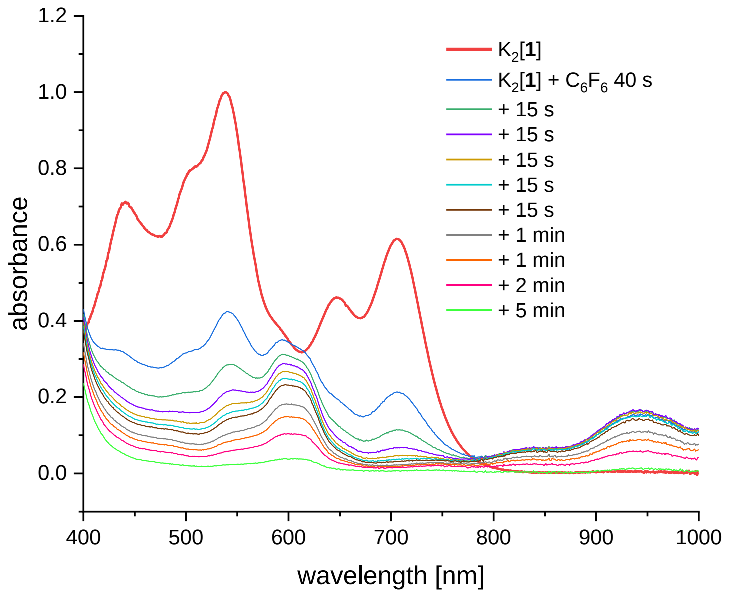

Figure S107: Monitoring the reaction of K_2[**1**] with C_6F_6 in THF by UV-vis spectroscopy at various time points (airtight cuvette; for technical reasons, the red trace was measured only 40 s after the reactants had been combined).

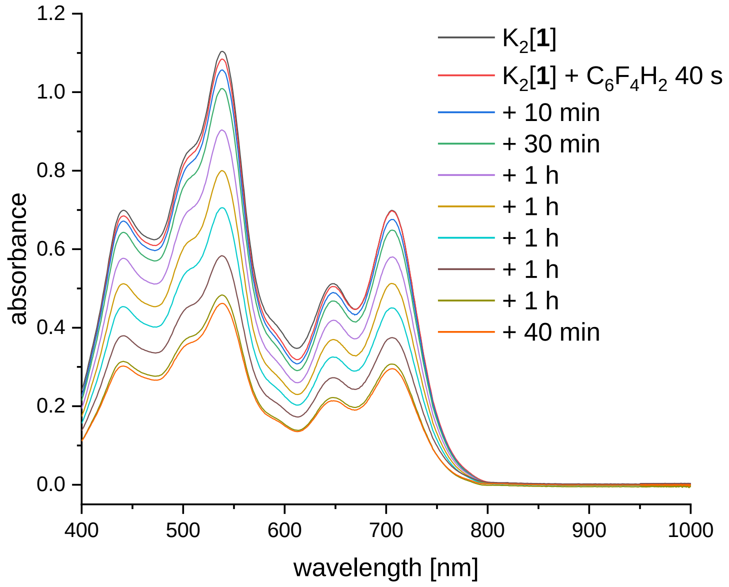

Figure S108: Monitoring the reaction of K_2[**1**] with 1,2,4,5-$C_6F_4H_2$ in THF by UV-vis spectroscopy at various time points (airtight cuvette; for technical reasons, the red trace was measured only 40 s after the reactants had been combined).

5 EPR spectrum of Li[**1**]

In an Ar-filled glove box, **1** (1.0 mg, 2.1 µmol) was reduced with Li granules (10 mg, exc.) in THF (0.5 mL) overnight. The THF solution of Li_2[**1**] was transferred to a vial charged with another equivalent of **1** (1.0 mg, 2.1 µmol). A color change from deep green to deep blue was observed immediately after **1** was fully dissolved. The resulting solution of Li[**1**] (4.2 µmol) was diluted with THF (total volume of the sample after diluting: 8.5 mL) to obtain a concentration of 0.49 mmol L^{-1}. An aliquot (0.3 mL) of the solution was transferred to a 3 mm EPR quartz tube (inner diameter: 2 mm) by using a syringe. The tube was flame-sealed under vacuum and a CW-EPR spectrum was recorded at rt on a Bruker EMXnano tabletop EPR spectrometer using its Xenon controlling/recording software. Parameters of the EPR spectrum shown below: microwave frequency = 9.6381 GHz; power = 2 mW; field modulation = 0.05 G; recording time = 100 min. The EPR spectrum was simulated and plotted with the program EasySpin 5.2.33, which runs in MATLAB 9.9 (R2020b) (Figure S109).

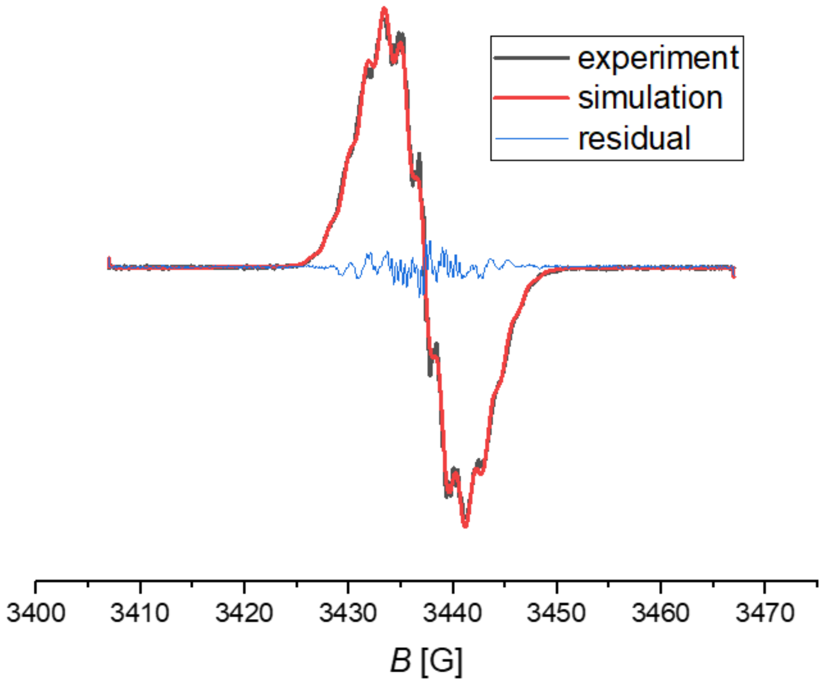

Figure S109: Experimental (black) and simulated (red) EPR spectra of Li[**1**] in THF (c = 0.49 mmol L^{-1}) at rt. The difference between the experimental and simulated spectra likely arises from couplings of Me protons that could not be included into the simulation. g: 2.0033; linewidth: 0.8 G; RMSD: 0.019; Hyperfine couplings: 2 x B: 0.16 mT (1.6 G); 4 x H: 0.20 mT (2.0 G); 4 x H: 0.15 mT (1.5 G); 2 x H: 0.07 mT (0.7 G).

Note: For the computed spin density of $[\mathbf{1}]^{\bullet-}$, confirming the α-spin density at the C*Me* groups, see chapter 7.1.1.

6 X-ray crystal structure determinations

Single-crystal diffraction data were collected at −100 °C on a *STOE IPDS II* two-circle diffractometer equipped with a *Genix 3D HS* microfocus Mo*K*α X-ray source (λ = 0.71073 Å). The finalization of the data, including empirical absorption corrections, was done using the *CrysAlisPro* software v.1.171.42.43a (Rigaku Oxford Diffraction, 2022). The structures were solved using the SHELXS or SHELXT programs and refined against $|F|^2$ with full-matrix least-squares techniques using the program *SHELXL*-2018/3.[S6–S8] All H atoms were located geometrically and refined riding on the pivot atom.

For compounds $[Li(12\text{-}c\text{-}4)_2]$[**7**]$_{0.79}$[**8**]$_{0.21}$•2THF and $[Li(thf)_4]$[**3**$^{\mathbf{Me}}$], the diffraction data was collected at T = 100(2) K on the *P24* beamline of the *DESY PETRA III* synchrotron, equipped with a four-circle *HUBER* diffractometer with Eulerian geometry and X-*Spectrum Lambda 7.5M CdTe* ($[Li(12\text{-}c\text{-}4)_2]$[**7**]$_{0.79}$[**8**]$_{0.21}$•2THF) or Pilatus3 CdTe 1M ($[Li(thf)_4]$[**3**$^{\mathbf{Me}}$]) photon-counting detectors. Radiation with wavelengths of λ = 0.5 Å (24.797 keV) and 0.56002 Å (22.139 keV) was used for ($[Li(12\text{-}c\text{-}4)_2]$[**7**]$_{0.79}$[**8**]$_{0.21}$•2THF and $[Li(thf)_4]$[**3**$^{\mathbf{Me}}$], respectively. For ($[Li(thf)_4]$[**3**$^{\mathbf{Me}}$]) the data from two single crystals were merged to gain data completeness. The data was acquired by two runs at two different detector 2Θ positions, 0° and −10°, to compensate the data loss from the gaps between the detector chips. Each run was done by 360° ϕ rotation with a 0.5° scan width and an exposure time of 2 s ($[Li(12\text{-}c\text{-}4)_2]$[**7**]$_{0.79}$[**8**]$_{0.21}$•2THF) or 1.5 s ($[Li(thf)_4]$[**3**$^{\mathbf{Me}}$]) per frame.

Due to severe conformational disorder, the THF molecules were refined in most cases with SADI restraints to the C−C and C−O distances. The crystal of $[Li(thf)_3]$[**3**]•THF chosen for the diffraction study was found to be twinned by pseudo-merohedry (m*P* → o*P*) with the twin law of (−1 0 0 / 0 1 0 / 0 0 1) and the relative domain weights of 0.679(2)/0.321. Analogously, the crystal $[K(thf)_{2.5}]_2$[**1**] was also twinned by pseudo-merohedry (m*P* → o*C*) with the twin law of (0 0 -1 / 0 1 0 / −1 0 0) and the relative domain weights of 0.594(2):0.406. Both structures were refined using the *SHELXL* TWIN instruction.

CIF files containing the crystallographic information are deposited with the Cambridge Crystallographic Data Centre under the deposition codes CCDC2434035-2434046 and CCDC2448823 and can be obtained free of charge via www.ccdc.cam.ac.uk/data_request/cif. Crystallographic data and parameters of the diffraction experiments are given in Tables S12 – S18.

Acknowledgements:

Parts of this research were carried out on the P24 beamline (projects I-20230286 and I-20220865) at PETRA III at DESY, a member of the Helmholtz Association (HGF).

Table S12: Selected crystallographic data for **1**•THF and $[Li(thf)_2]_2$[**1**].

**Parameter**	**1**•THF	$[Li(thf)_2]_2$[**1**]
**Chemical formula**	$C_{36}H_{34}B_2{\cdot}C_4H_8O$	$C_{52}H_{66}B_2Li_2O_4$
M_r	560.35	790.54
**Crystal system, space group**	Triclinic, $P\overline{1}$	Triclinic, $P\overline{1}$
**Temperature (K)**	173	173
***a*, *b*, *c* (Å)**	9.7389(12), 12.0632(15), 14.759(3)	10.4604(6), 10.9592(6), 20.3977(12)
***α*, *β*, *γ* (°)**	106.940(14), 106.819(14), 90.195(10)	98.965(5), 99.984(5), 99.906(5)
***V* (Å^3)**	1580.5(4)	2226.7(2)
Z	2	2
***F*(000)**	600	852
D_x **(Mg m^{-3})**	1.177	1.179
**Radiation type**	Mo *K*α	Mo *K*α
***μ* (mm^{-1})**	0.07	0.07
**Crystal shape**	Block	Prism
**Color**	Colorless	Dark green
**Crystal size (mm)**	0.29 × 0.20 × 0.18	0.29 × 0.27 × 0.12
**Absorption correction**	Multi-scan	Multi-scan
T_{min}, T_{max}	0.159, 1.000	0.845, 1.000
**No. of measured, independent and observed [*I* > 2*σ*(*I*)] reflections**	9020, 9020, 3820	17259, 8876, 4263
R_{int}	0.058	0.051
θ_{max} **(°)**	27.1	26.4
**Range of *h*, *k*, *l***	*h* = -12→12, *k* = -15→15, *l* = -18→18	*h* = -12→13, *k* = -13→13, *l* = -25→25
$R[F^2 > 2\sigma(F^2)]$, $wR(F^2)$, S	0.061, 0.160, 0.82	0.051, 0.125, 0.85
**No. of reflections**	9020	8876
**No. of parameters**	415	643
**No. of restraints**	52	0
$\Delta\rho_{max}$, $\Delta\rho_{min}$ **(e Å^{-3})**	0.54, -0.32	0.32, -0.24

Computer programs: *X-AREA* (Stoe & Cie, 2001), *CrysAlis PRO* 1.171.42.43a (Rigaku OD, 2022), *SHELXS* (G. M. Sheldrick, 1997), *SHELXT* (G. M. Sheldrick, 2015), *SHELXL2018*/3 (Sheldrick, 2018).

Table S13: Selected crystallographic data for $[K(thf)_{2.5}]_2$[**1**] and $[Li(12\text{-}c\text{-}4)_2]$[**1**].

**Parameter**	$[K(thf)_{2.5}]_2$[**1**]	$[Li(12\text{-}c\text{-}4)_2]$[**1**]
**Chemical formula**	$C_{56}H_{74}B_2K_2O_5$	$C_{16}H_{32}LiO_8 \cdot C_{36}H_{34}B_2$
M_r	926.97	847.60
**Crystal system, space group**	Monoclinic, $P2_1/n$	Orthorhombic, *Cmca*
**Temperature (K)**	173	173
***a*, *b*, *c* (Å)**	24.485(3), 18.4613(15), 24.567(3)	18.104(2), 12.074(2), 20.313(2)
**α, β, γ (°)**	90, 114.208(15), 90	90, 90, 90
**V (Å^3)**	10128(2)	4440.2(10)
Z	8	4
***F*(000)**	3984	1820
**D_x (Mg m^{-3})**	1.216	1.268
**Radiation type**	Mo *K*α	Mo *K*α
**μ (mm^{-1})**	0.23	0.08
**Crystal shape**	Plate	Plate
**Color**	Black	Black
**Crystal size (mm)**	0.36 × 0.34 × 0.18	0.26 × 0.18 × 0.05
**Absorption correction**	Multi-scan	Multi-scan
T_{min}, T_{max}	0.111, 1.000	0.294, 1.000
**No. of measured, independent and observed [$I > 2\sigma(I)$] reflections**	34790, 10603, 7848	8767, 1650, 986
R_{int}	0.120	0.128
**θ_{max} (°)**	20.8	23.3
**Range of *h*, *k*, *l***	$h = -22 \rightarrow 24$, $k = -18 \rightarrow 18$, $l = -24 \rightarrow 24$	$h = -20 \rightarrow 20$, $k = -13 \rightarrow 13$, $l = -22 \rightarrow 21$
$R[F^2 > 2\sigma(F^2)]$, $wR(F^2)$, S	0.079, 0.217, 1.03	0.053, 0.116, 0.99
**No. of reflections**	10603	1650
**No. of parameters**	1177	205
**No. of restraints**	39	6
**$\Delta\rho_{max}$, $\Delta\rho_{min}$ (e Å^{-3})**	0.78, -0.39	0.16, -0.18

Computer programs: *X-AREA* (Stoe & Cie, 2001), *CrysAlis PRO* 1.171.42.43a (Rigaku OD, 2022), *SHELXS* (G. M. Sheldrick, 1997), *SHELXT* (G. M. Sheldrick, 2015), *SHELXL2018*/3 (Sheldrick, 2018).

Table S14: Selected crystallographic data for [Li(thf)$_3$][**3**]•THF and [Li(thf)$_4$][**3^{Me}**].

**Parameter**	[Li(thf)$_3$][**3**]•THF	[Li(thf)$_4$][**3^{Me}**]
**Chemical formula**	$C_{52}H_{65}B_2LiO_4 \cdot C_4H_8O$	$C_{16}H_{32}LiO_4 \cdot C_{42}H_{45}B_2O$
M_r	854.70	882.75
**Crystal system, space group**	Monoclinic, $P2_1/c$	Triclinic, $P\overline{1}$
**Temperature (K)**	173	100
***a*, *b*, *c* (Å)**	11.1656(9), 19.9415(18), 21.488(2)	12.78309(18), 13.5780(2), 16.77186(18)
**α, β, γ (°)**	90, 90.079(9), 90	74.4388(12), 89.3841(10), 63.6623(15)
V **(Å^3)**	4784.5(8)	2493.42(7)
Z	4	2
F**(000)**	1848	956
D_x **(Mg m^{-3})**	1.187	1.176
**Radiation type**	Mo *K*α	Synchrotron, λ = 0.56002 Å
**μ (mm^{-1})**	0.07	0.05
**Crystal shape**	Block	Plate
**Color**	Colorless	Colorless
**Crystal size (mm)**	0.26 × 0.13 × 0.13	0.20 × 0.15 × 0.05
**Absorption correction**	Multi-scan	Multi-scan
T_{min}, T_{max}	0.558, 1.000	0.777, 1.000
**No. of measured, independent and observed [$I > 2\sigma(I)$] reflections**	54880, 9084, 6264	72967, 13854, 12604
R_{int}	0.161	0.036
θ_{max} **(°)**	25.7	24.5
**Range of *h*, *k*, *l***	h = -13→13, k = -24→24, l = -26→26	h = -18→18, k = -20→19, l = -24→24
$R[F^2 > 2\sigma(F^2)]$, $wR(F^2)$, S	0.076, 0.221, 1.04	0.056, 0.160, 1.02
**No. of reflections**	9084	12203
**No. of parameters**	622	625
**No. of restraints**	75	12
$\Delta\rho_{max}$, $\Delta\rho_{min}$ **(e Å^{-3})**	0.40, -0.36	0.42, -0.26

Computer programs: *X-AREA* (Stoe & Cie, 2001), *CrysAlis PRO* 1.171.42.43a (Rigaku OD, 2022), *SHELXS* (G. M. Sheldrick, 1997), *SHELXT* (G. M. Sheldrick, 2015), *SHELXL2018*/3 (Sheldrick, 2018).

Table S15: Selected crystallographic data for [Li(thf)$_4$][**5**]•0.4 THF and [Li(thf)$_4$][**2**]•0.2 THF.

**Parameter**	[Li(thf)$_4$][**5**]•0.4 THF	[Li(thf)$_4$][**2**]•0.2 THF
**Chemical formula**	$C_{42}H_{36}B_2F_3 \cdot C_{16}H_{30}LiO_4 \cdot 0.4(C_4H_8O)$	$C_{42}H_{37}B_2F_2 \cdot C_{16}H_{32}LiO_4 \cdot 0.2(C_4H_8O)$
M_r	943.52	911.11
**Crystal system, space group**	Orthorhombic, *Pbca*	Orthorhombic, *Pbca*
**Temperature (K)**	173	173
***a*, *b*, *c* (Å)**	22.4440(12), 18.1193(10), 26.9981(14)	22.3572(9), 18.1609(8), 26.8714(13)
**α, β, γ (°)**	90, 90, 90	90, 90, 90
V **(Å^3)**	10979.3 (10)	10910.5(8)
Z	8	8
F**(000)**	4032	3904
D_x **(Mg m^{-3})**	1.142	1.109
**Radiation type**	Mo *K*α	Mo *K*α
**μ (mm^{-1})**	0.08	0.07
**Crystal shape**	Plate	Plate
**Color**	Colorless	Colorless
**Crystal size (mm)**	0.56 × 0.52 × 0.12	0.50 × 0.45 × 0.18
**Absorption correction**	Multi-scan	Multi-scan
T_{min}, T_{max}	0.061, 1.000	0.455, 1.000
**No. of measured, independent and observed [$I > 2\sigma(I)$] reflections**	56120, 6690, 4841	39747, 6634, 3982
R_{int}	0.097	0.077
θ_{max} **(°)**	22.0	22.0
**Range of *h*, *k*, *l***	$h = -23 \rightarrow 23$, $k = -17 \rightarrow 19$, $l = -28 \rightarrow 28$	$h = -23 \rightarrow 23$, $k = -19 \rightarrow 19$, $l = -26 \rightarrow 28$
$R[F^2 > 2\sigma(F^2)]$, $wR(F^2)$, S	0.092, 0.297, 1.17	0.107, 0.306, 1.08
**No. of reflections**	6690	6634
**No. of parameters**	745	714
**No. of restraints**	109	152
$\Delta\rho_{max}$, $\Delta\rho_{min}$ **(e Å^{-3})**	0.97, -0.28	1.07, -0.36

Computer programs: *X-AREA* (Stoe & Cie, 2001), *CrysAlis PRO* 1.171.42.43a (Rigaku OD, 2022), *SHELXS* (G. M. Sheldrick, 1997), *SHELXT* (G. M. Sheldrick, 2015), *SHELXL2018*/3 (Sheldrick, 2018).

Table S16: Selected crystallographic data for [K(thf)$_4$][**6**] and [K(thf)$_4$][**4**].

**Parameter**	[K(thf)$_4$][**6**]	[K(thf)$_4$][**4**]
**Chemical formula**	$C_{42}H_{36}B_2F_3{\cdot}C_{16}H_{32}KO_4$	$C_{42}H_{34}B_2F_5{\cdot}C_{16}H_{32}KO_4$
M_r	946.84	982.82
**Crystal system, space group**	Monoclinic, $P2_1/n$	Monoclinic, $P2_1/n$
**Temperature (K)**	173	173
***a*, *b*, *c* (Å)**	10.7053(2), 22.0669(4), 20.8583(4)	11.0570(9), 21.9190(15), 20.8223(11)
**α, β, γ (°)**	90, 90.6246(17), 90	90, 90.676(6), 90
V **(Å^3)**	4927.11(16)	5046.1(6)
Z	4	4
***F*(000)**	2016	2080
D_x **(Mg m^{-3})**	1.276	1.294
**Radiation type**	Mo *K*α	Mo *K*α
**μ (mm^{-1})**	0.17	0.17
**Crystal shape**	Prism	elongated plate
**Color**	Colorless	Colorless
**Crystal size (mm)**	0.41 × 0.30 × 0.25	0.36 × 0.11 × 0.05
**Absorption correction**	Multi-scan	Multi-scan
T_{min}, T_{max}	0.695, 1.000	0.432, 1.000
**No. of measured, independent and observed [$I > 2\sigma(I)$] reflections**	69301, 11736, 9715	17377, 6106, 3814
R_{int}	0.055	0.073
θ_{max} **(°)**	27.9	22.0
**Range of *h*, *k*, *l***	$h = -13 \rightarrow 14$, $k = -29 \rightarrow 29$, $l = -27 \rightarrow 27$	$h = -11 \rightarrow 8$, $k = -23 \rightarrow 23$, $l = -21 \rightarrow 21$
$R[F^2 > 2\sigma(F^2)]$, $wR(F^2)$, S	0.064, 0.185, 1.02	0.076, 0.240, 1.04
**No. of reflections**	11736	6106
**No. of parameters**	657	666
**No. of restraints**	70	60
$\Delta\rho_{max}$, $\Delta\rho_{min}$ **(e Å^{-3})**	0.56, -0.68	0.51, -0.56

Computer programs: *X-AREA* (Stoe & Cie, 2001), *CrysAlis PRO* 1.171.42.43a (Rigaku OD, 2022), *SHELXS* (G. M. Sheldrick, 1997), *SHELXT* (G. M. Sheldrick, 2015), *SHELXL2018*/3 (Sheldrick, 2018).

Table S17: Selected crystallographic data for [Li(12-c-4)$_2$][**7**]$_{0.79}$[**8**]$_{0.21}$•2THF and [Li(thf)$_4$][**8**].

**Parameter**	[Li(12-c-4)$_2$][**7**]$_{0.79}$[**8**]$_{0.21}$•2THF	[Li(thf)$_4$][**8**]
**Chemical formula**	$C_{40}H_{41}B_2{\cdot}C_{16}H_{32}LiO_8{\cdot}2(C_4H_8O)$	$C_{40}H_{41}B_2{\cdot}C_{16}H_{32}LiO_4$
M_r	1046.91	838.70
**Crystal system, space group**	Monoclinic, $P2_1/c$	Monoclinic, $P2_1/n$
**Temperature (K)**	173	173
***a*, *b*, *c* (Å)**	11.6785(3), 24.0333(4), 20.2939(4)	13.5981(5), 19.0541(8), 19.1246(8)
**α, β, γ (°)**	90, 91.4389(17), 90	90, 101.552(4), 90
V (Å^3)	5694.12(19)	4854.8(3)
Z	4	4
F(000)	2264	1816
D_x (Mg m^{-3})	1.221	1.147
**Radiation type**	Synchrotron, λ= 0.500 Å	Mo $K\alpha$
**μ (mm^{-1})**	0.04	0.07
**Crystal shape**	Plate	Plate
**Color**	Colorless	Colorless
**Crystal size (mm)**	0.10 × 0.05 × 0.03	0.55 × 0.52 × 0.14
**Absorption correction**	Multi-scan	Multi-scan
T_{min}, T_{max}	0.841, 1.000	0.450, 1.000
**No. of measured, independent and observed [$I > 2\sigma(I)$] reflections**	58106, 10758, 7295	59466, 9547, 7051
R_{int}	0.036	0.070
θ_{max} (°)	17.8	26.0
**Range of *h*, *k*, *l***	h = -14→14, k = -29→29, l = -24→24	h = -16→16, k = -23→20, l = -23→23
$R[F^2 > 2\sigma(F^2)]$, $wR(F^2)$, S	0.058, 0.176, 0.99	0.065, 0.197, 1.04
**No. of reflections**	10758	9547
**No. of parameters**	765	631
**No. of restraints**	54	138
$\Delta\rho_{max}$, $\Delta\rho_{min}$ (e Å^{-3})	0.56, -0.55	0.44, -0.40

Computer programs: *X-AREA* (Stoe & Cie, 2001), *CrysAlis PRO* 1.171.42.43a (Rigaku OD, 2022), *SHELXS* (G. M. Sheldrick, 1997), *SHELXT* (G. M. Sheldrick, 2015), *SHELXL2018*/3 (Sheldrick, 2018).

Table S18: Selected crystallographic data for [K(2.2.2crypt)][**1**]•2THF

**Parameter**	[K(2.2.2crypt)][**1**]•2THF
**Chemical formula**	$C_{36}H_{34}B_2 \cdot C_{18}H_{36}KN_2O_6 \cdot 2(C_4H_8O)$
M_r	1048.04
**Crystal system, space group**	Triclinic, $P\overline{1}$
**Temperature (K)**	173
***a*, *b*, *c* (Å)**	11.3888(6), 13.5383(6), 20.6945(10)
**α, β, γ (°)**	85.009(4), 76.891(4), 68.512(4)
***V* (Å^3)**	2891.6(3)
Z	2
***F*(000)**	1130
D_x **(Mg m^{-3})**	1.204
**Radiation type**	Mo *K*α
**μ (mm^{-1})**	0.15
**Crystal shape**	Prism
**Color**	Dark blue
**Crystal size (mm)**	0.45 × 0.35 × 0.30
**Absorption correction**	Multi-scan
T_{min}, T_{max}	0.636, 1.000
**No. of measured, independent and observed [$I > 2\sigma(I)$] reflections**	29201, 12217, 9776
R_{int}	0.055
θ_{max} **(°)**	26.7
**Range of *h*, *k*, *l***	$h = -14 \rightarrow 14$, $k = -15 \rightarrow 17$, $l = -26 \rightarrow 26$
$R[F^2 > 2\sigma(F^2)]$, $wR(F^2)$, S	0.052, 0.147, 1.02
**No. of reflections**	12217
**No. of parameters**	711
**No. of restraints**	57
$\Delta\rho_{max}$, $\Delta\rho_{min}$ **(e Å^{-3})**	0.40, -0.37

Computer programs: *X-AREA* (Stoe & Cie, 2001), *CrysAlis PRO* 1.171.42.43a (Rigaku OD, 2022), *SHELXS* (G. M. Sheldrick, 1997), *SHELXT* (G. M. Sheldrick, 2015), *SHELXL2018*/3 (Sheldrick, 2018).

6.1 Single-crystal X-ray structure analysis of **1**•THF

The compound **1**•THF crystallizes as a THF solvate in the triclinic space group $P\bar{1}$ (No. 2), with two crystallographically unique, nearly identical DBA-based molecules located on inversion centers and one conformationally disordered THF solvent molecule occupying the general position. (Fig. S110) resulting in one THF molecule per one (two halves) DBA-derivative.

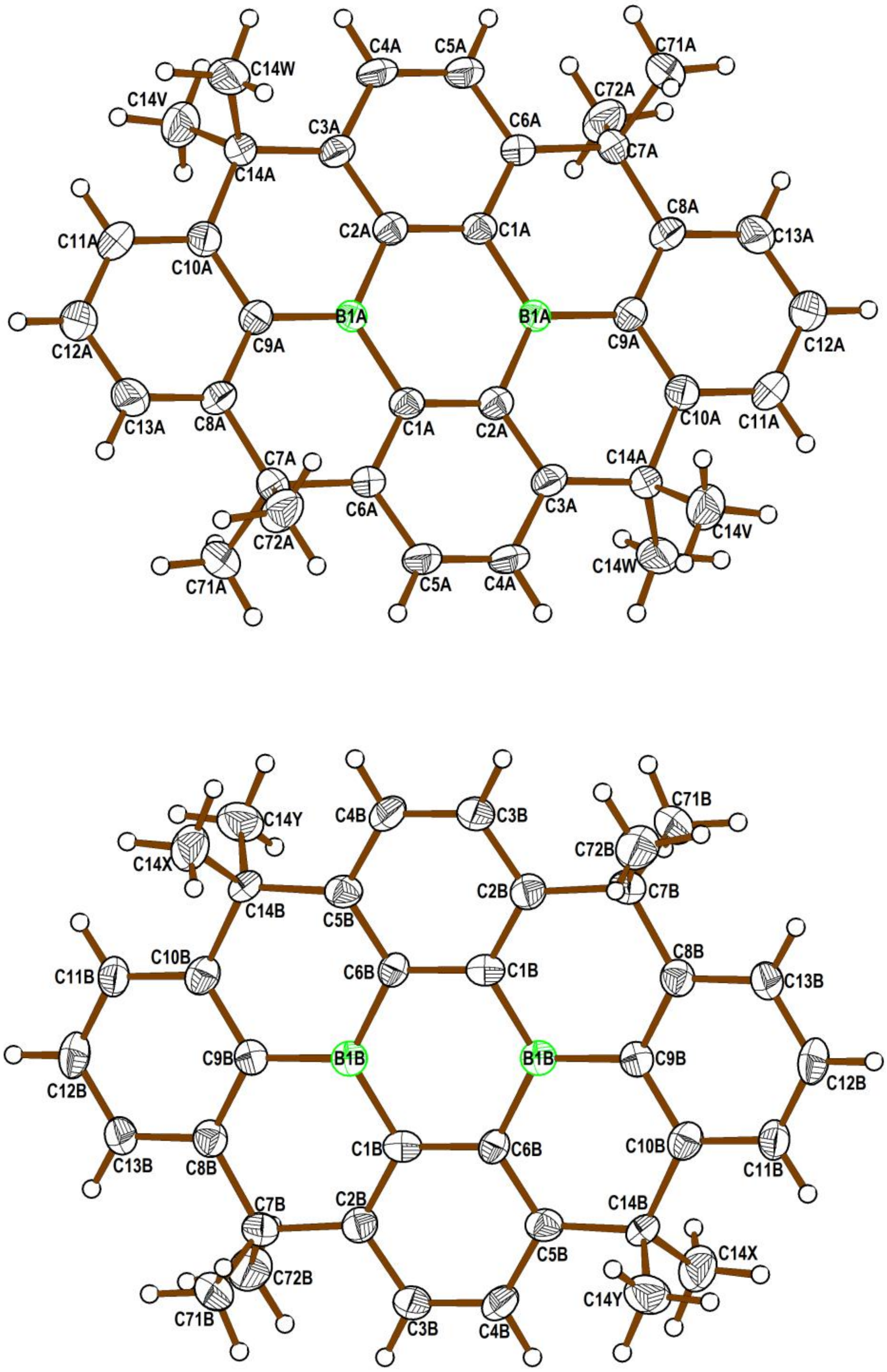

a

b

Figure S110: Structure of the two crystallographically unique molecules (a and b) of **1**•THF (atomic displacement ellipsoids at the 50% probability level). Disordered solvent molecules are omitted for clarity.

6.2 Single-crystal X-ray structure analysis of [Li(thf)$_2$]$_2$[**1**]

The compound [Li(thf)$_2$]$_2$[**1**] crystallizes in the triclinic space group $P\bar{1}$ (No. 2). The $[DBA]^{2-}$ anion is coordinated on both sides by $[Li(thf)_2]^{+}$ complex cations (Fig. S111), which occupy general positions. The Li···C distances vary from 2.411(4) to 2.481(4) Å.

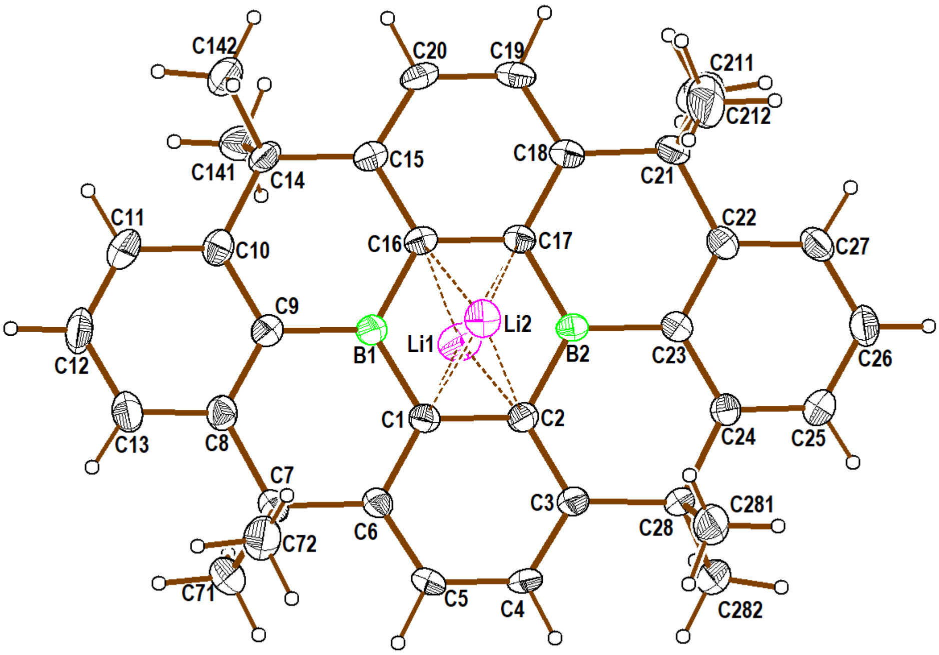

Figure S111: Structure of the neutral moiety of [Li(thf)$_2$]$_2$[**1**] (atomic displacement ellipsoids at the 50% probability level). The thf ligands are omitted for clarity.

6.3 Single-crystal X-ray structure analysis of [K(thf)$_{2.5}$]$_2$[**1**]

The compound [K(thf)$_{2.5}$]$_2$[**1**] crystallizes in the monoclinic space group $P2_1/n$ (No. 14) with two crystallographically unique DBA-based anions, two K^+ cations and nine THF molecules occupying general crystallographic positions (Fig. S112 a,b). In the crystal, the K^+ cations are coordinated by DBA fragments and partly disordered THF molecules, resulting in the formation of K_4(*DBA-derivative*)$_2$(THF)$_{10}$ neutral tetramers (Fig. S112 c,d). At that, K···C(DBA) distances ranging from 2.951(7) to 3.327(7) Å.

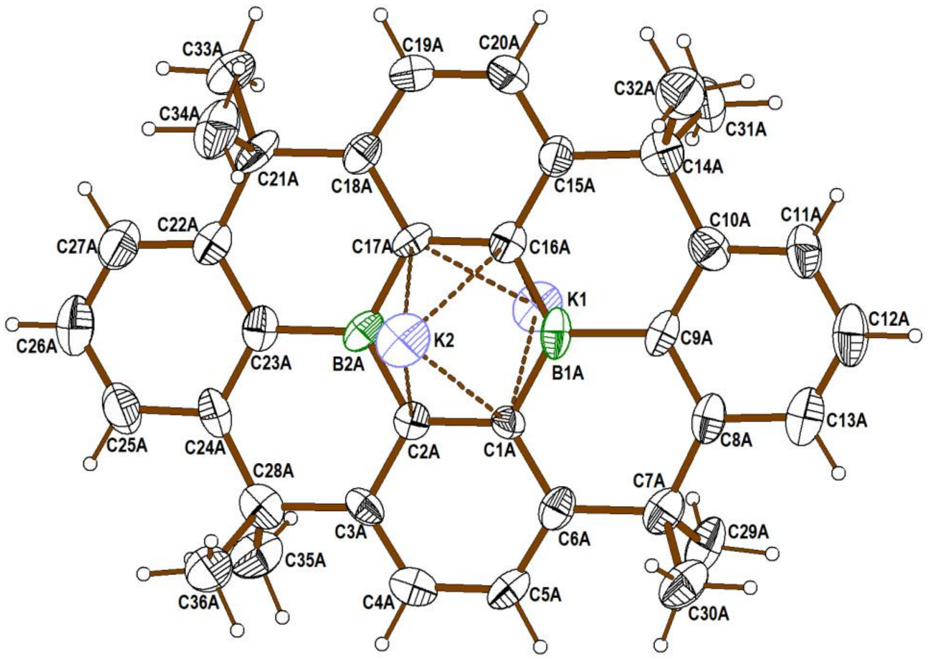
C33A
C19A
C20A
C32A
C34A
C31A
C21A
C18A
C15A
C14A
C22A
C10A
C11A
C27A
C17A
C16A
K1
C12A
K2
C26A
C9A
C23A
B2A
B1A
C13A
C2A
C1A
C8A
C25A
C24A
C28A
C7A
C29A
C3A
C6A
C35A
C36A
C4A
C5A
C30A

a

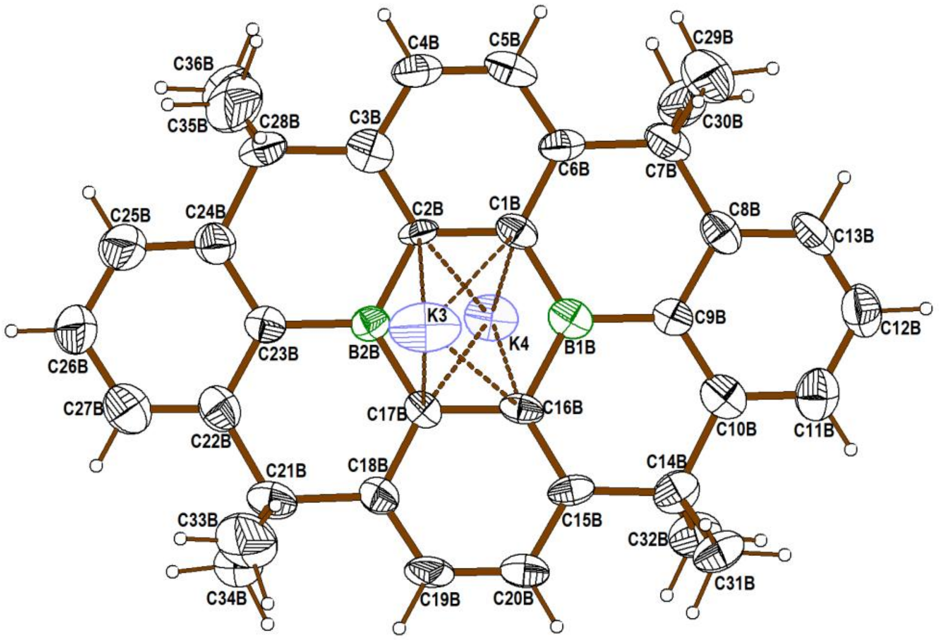
C36B
C4B
C5B
C29B
C35B
C28B
C3B
C30B
C6B
C7B
C25B
C24B
C2B
C1B
C8B
C13B
K3
K4
C23B
C9B
C12B
C26B
B2B
B1B
C17B
C16B
C27B
C22B
C10B
C11B
C21B
C18B
C14B
C33B
C15B
C32B
C31B
C34B
C19B
C20B

b

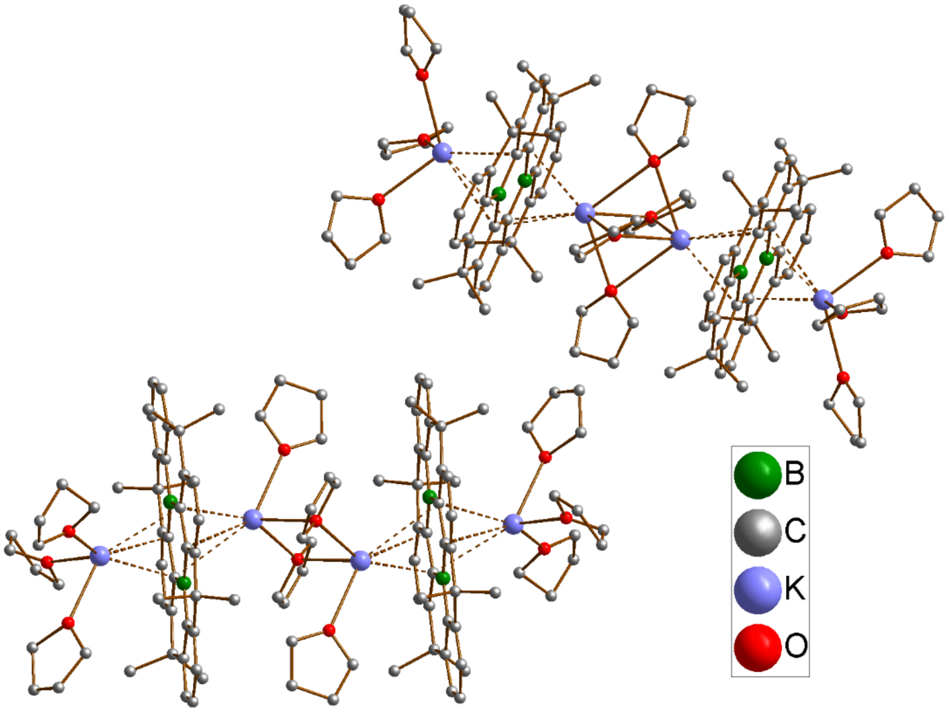

c

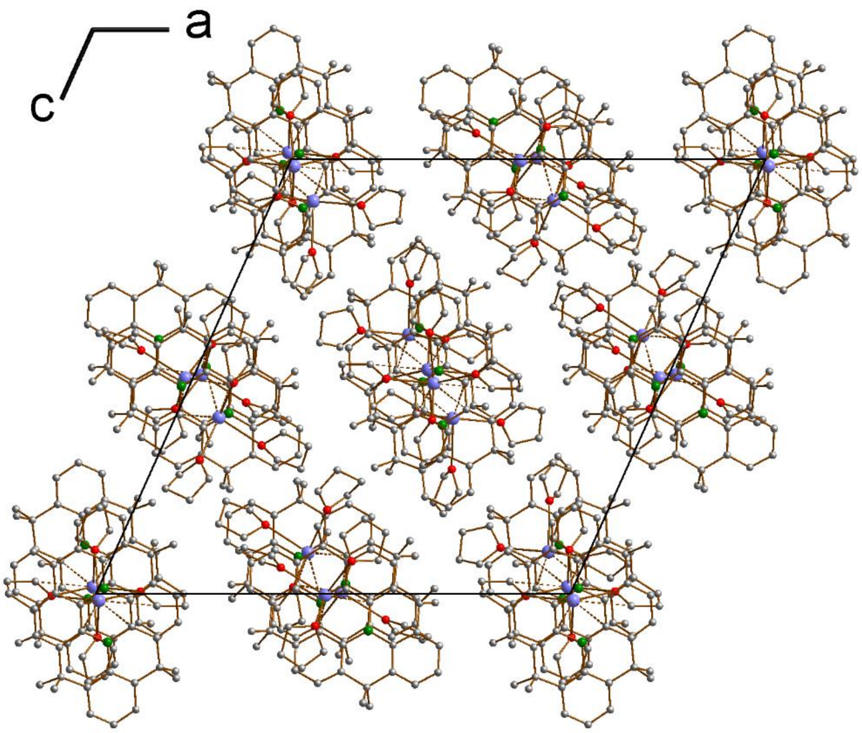

d

Figure S112: *a*, *b*) Fragments of the crystal structures of [K(thf)$_{2.5}$]$_2$[**1**], where the THF molecules are omitted for clarity (atomic displacement ellipsoids at the 50% probability level). *c*) Cation-anion tetramers in the crystal, *d*) crystal packing, H atoms are omitted for clarity.

6.4 Single-crystal X-ray structure analysis of [Li(12-c-4)$_2$][**1**]

The ionic compound [Li(12-c-4)$_2$][**1**] crystallizes solvent-free in the orthorhombic space group *Cmca* (*Cmce* in modern notation, No. 64). The [Li(12-*crown*-4)$_2$]$^+$ cation is located on an inversion center, while the DBA-containing anion (Fig. S113) lies on the 2/*m* special position.

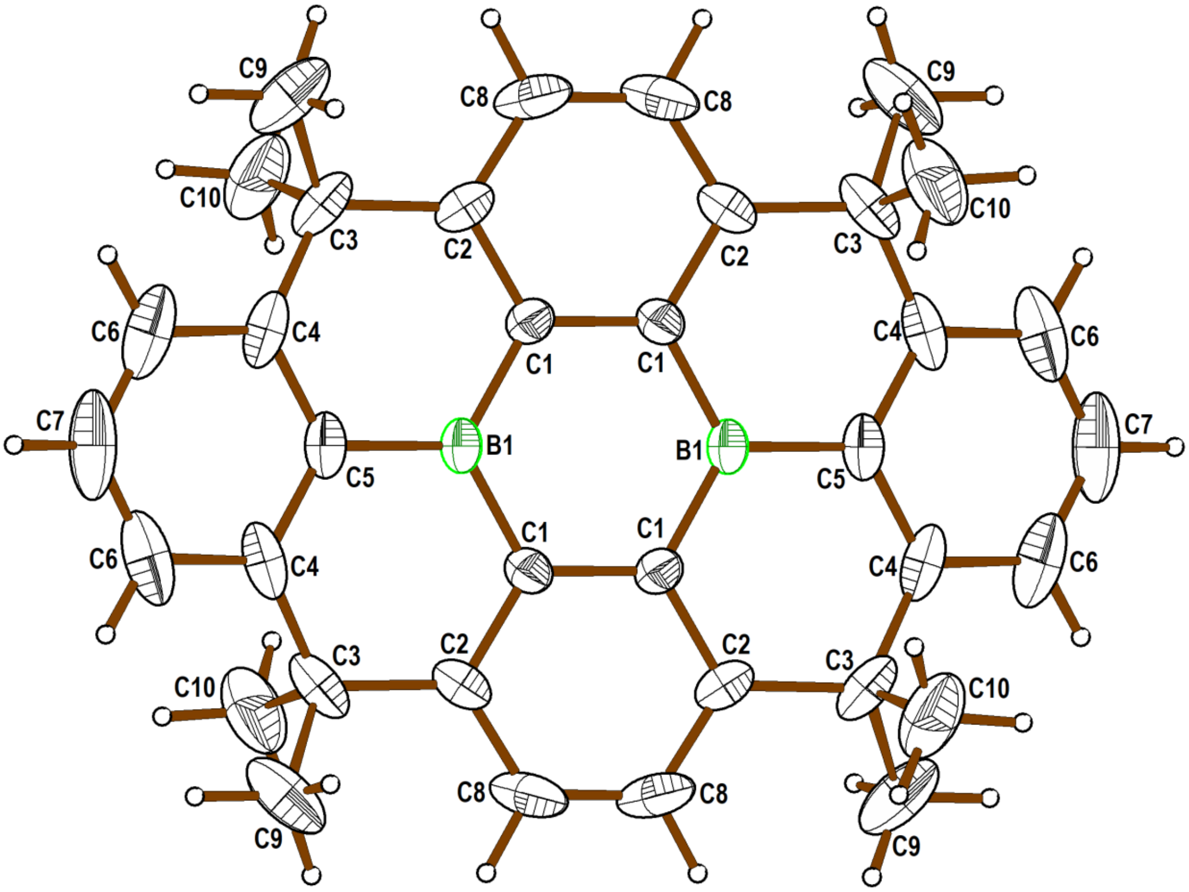

Figure S113: Structure of the anion in [Li(12-c-4)$_2$][**1**], atomic displacement ellipsoids at 50% probability level. The [Li(12-*crown*-4)$_2$]$^+$ cation is omitted for clarity.

6.5 Single-crystal X-ray structure analysis of [Li(thf)$_3$][**3**]•THF

Compound [Li(thf)$_3$][**3**]•THF (Fig. S114) crystallizes with one THF solvent molecule per formula unit in the monoclinic space group *P*2$_1$/*c* (No. 14). All structural moieties occupy general positions. The solvent molecule exhibits severe conformational disorder. The Li^+ cation is coordinated by the THF substituent *via* its O atom, and its coordination sphere is completed by three additional THF molecules.

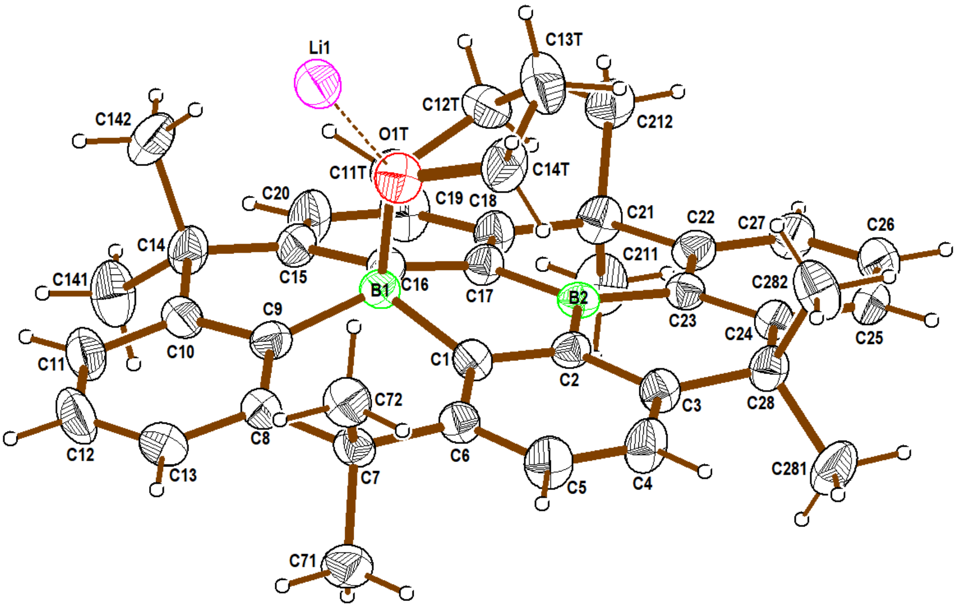

Figure S114: Structure of the anion in [Li(thf)$_3$][**3**]•THF, atomic displacement ellipsoids at 50% probability level. Coordinating thf ligands and disordered THF solvent molecules are omitted for clarity.

6.6 Single-crystal X-ray structure analysis of $[Li(thf)_4]$[**3**^{Me}]

The ionic compound $[Li(thf)_4]$[**3**^{Me}] crystallizes in the triclinic space group $P\bar{1}$ (No. 2) with both the $[Li(THF)_4]^+$ cation and the DBA-based anion occupying general positions (Fig. S115). The deprotonated 2,5-Me_2-THF fragment at B(1) is conformationally disordered over two positions with relative occupancies of 67:33%.

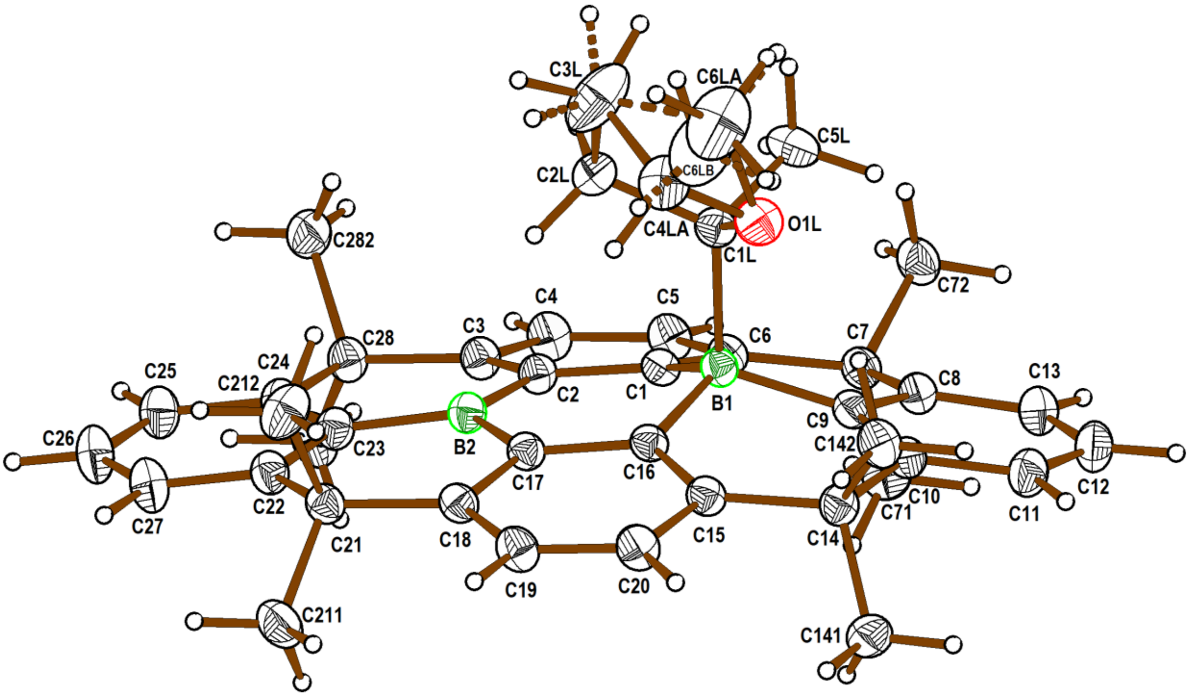

Figure S115: Structure of the anion in $[Li(thf)_4]$[**3**^{Me}], atomic displacement ellipsoids at 50% probability level. The $[Li(THF)_4]^+$ cation is omitted for clarity.

6.7 Single-crystal X-ray structure analysis of [Li(thf)$_4$][**5**]•0.4 THF

Compound [Li(thf)$_4$][**5**]•0.4 THF crystallizes as a THF solvate in the orthorhombic space group *Pbca* (No. 61), with the [Li(THF)$_4$]$^+$ cation, the DBA-based anion, and the THF solvent molecule occupying general positions. (Fig. S116). All three structural moieties exhibit disorder. Specifically, two of the four coordinated THF molecules in the cation, as well as the solvent molecule, are positionally and conformationally disordered. In the DBA-based anion (Fig. S116a), four of five terminal positions on the phenyl substituent are statistically shared between F and H atoms in complimentary ratios, while one position, F(2), is fully occupied by fluorine (Fig. S116b). Refinement of the site occupancy factors leads to the total of 3 F atoms (within refinement accuracy) disordered over 4 positions.

The co-crystallization of the 2,3,5-F_3-4,6-H_2 and 2,4,6-F_3-3,5-H_2 isomers of the $C_6F_3H_2$ substituent at the boron atom leads to varying F/H distributions, with both isomers sharing the same crystallographic positions within the crystal. In the solid state, the 2,3,5-F_3-4,6-H_2 isomer predominates (81%), while the 2,4,6-F_3-3,5-H_2 isomer accounts for only 19%. This distribution may differ for each individual crystal and is influenced by the relative solubility of the isomers, as the composition in solution can differ from that observed in the solid state.

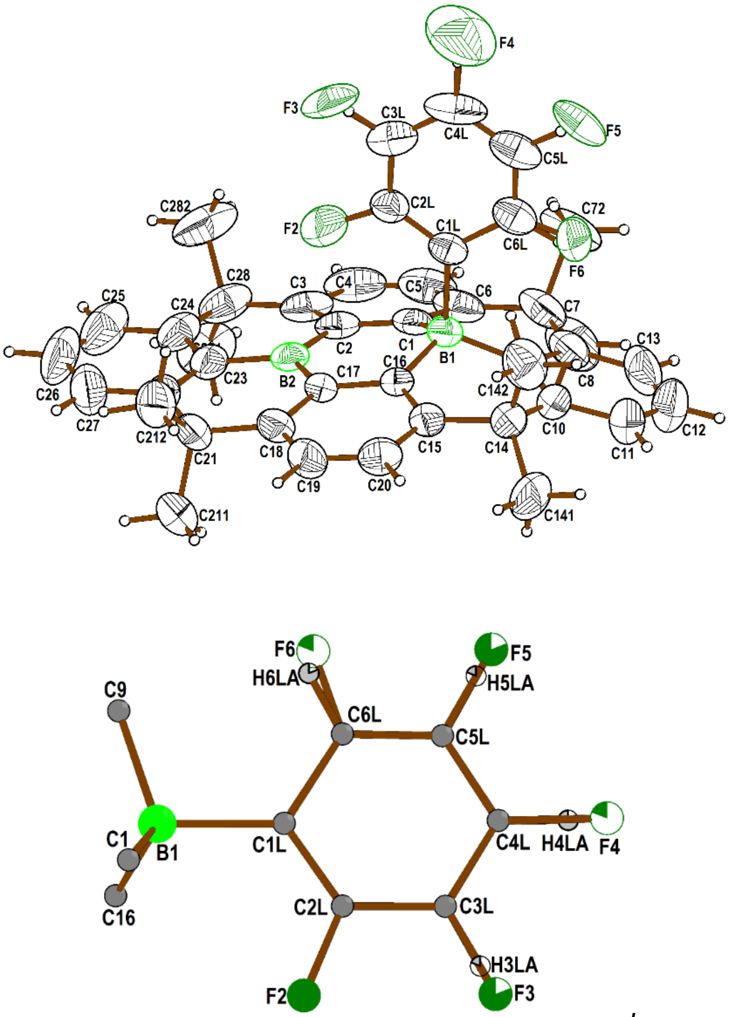

a

b

Figure S116: *a*) Structure of the anion in [Li(thf)$_4$][**5**]•0.4 THF, atomic displacement ellipsoids at 50% probability level. The [Li(THF)$_4$]$^+$ cation is omitted for clarity; *b*) the F/H disorder in the fluorinated phenyl ring depicted by sectors.

6.8 Single-crystal X-ray structure analysis of [Li(thf)$_4$][**2**]•0.2 THF

The ionic compound [Li(thf)$_4$][**2**]•0.2 THF (Fig. S117) crystallizes with one [Li(THF)$_4$]$^+$ cation and a THF solvent molecule in the orthorhombic space group *Pbca* (No. 61). All structural units occupy general positions, with the THF solvent molecule showing an occupancy of 20%.

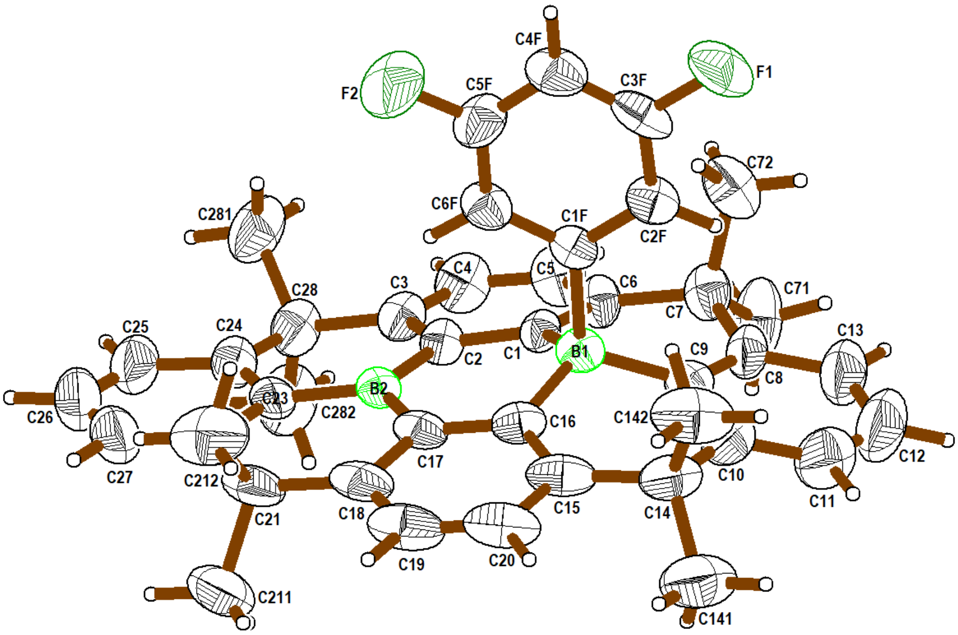

Figure S117: Structure of the anion in [Li(thf)$_4$][**2**]•0.2 THF, atomic displacement ellipsoids at 50% probability level. The [Li(THF)$_4$]$^+$ cation is omitted for clarity.

6.9 Single-crystal X-ray structure analysis of [K(thf)$_4$][**6**]

Compound [K(thf)$_4$][**6**], like the isostructural [K(thf)$_4$][**4**], crystallizes in the monoclinic space group *P*2$_1$/*n* (No. 14), with all structural units occupying general positions. Similar to [K(thf)$_4$][**4**], the [K(THF)$_4$]$^+$ cationic fragment, which exhibits slight conformational disorder, coordinates to the DBA with K…C distances ranging from 3.3040(17) – 3.3637(16) Å (Fig. S118).

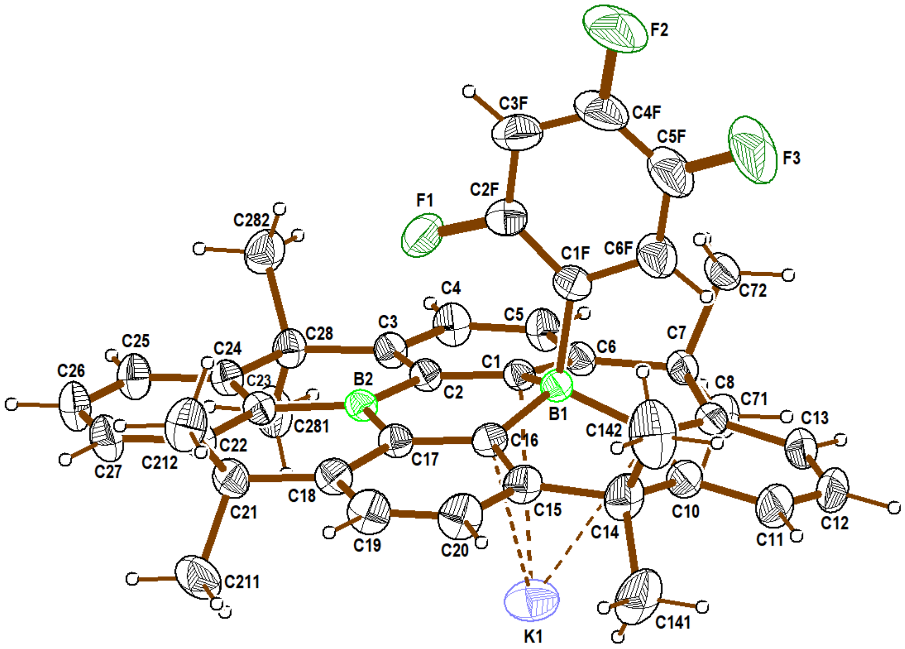

Figure S118: Structure of the anion in [K(thf)$_4$][**6**], atomic displacement ellipsoids at 50% probability level. The disordered thf ligands are omitted for clarity.

6.10 Single-crystal X-ray structure analysis of [K(thf)$_4$][**4**]

Compound [K(thf)$_4$][**4**], like the isostructural [K(thf)$_4$][**6**], crystallizes in the monoclinic space group $P2_1/n$ (No. 14), with all structural units occupying general positions. As in [K(thf)$_4$][**6**], the [K(THF)$_4$]$^+$ cationic fragment, which exhibits slight conformational disorder, coordinates to the DBA fragment with K…C distances ranging from of 3.181(5) – 3.470(4) Å (Fig. S119).

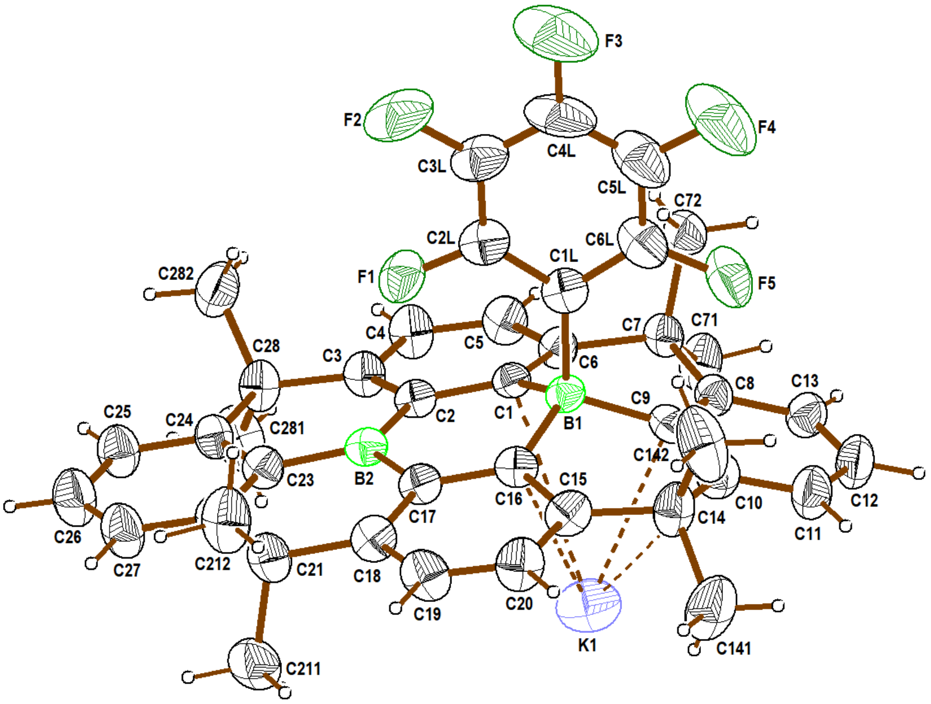

Figure S119: Structure of the anion in [K(thf)$_4$][**4**], atomic displacement ellipsoids at 50% probability level. The disordered thf ligands are omitted for clarity.

6.11 Single-crystal X-ray structure analysis of [Li(12-c-4)$_2$][**7**]$_{0.79}$[**8**]$_{0.21}$•2THF

The ionic compound [Li(12-c-4)$_2$][**7**]$_{0.79}$[**8**]$_{0.21}$•2THF crystallizes in the monoclinic space group *P*2$_1$/*c* (No. 14), with both the DBA-based anion and the [Li(12-crown-4)$_2$]$^+$ cation occupying general positions. The reaction produces two distinct products, which share ~~a~~ structurally identical DBA fragment but differ in the substituents bound to the boron atom B(1): CH_2(cyclo-C_3H_5) and n-but-3-en. These products co-crystallize within the same crystal lattice so that these fragments overlap in the unique part of the crystal structure. Detailed analysis of the diffraction pattern showed and no sign for larger unit cell and therefore no superstructural ordering. The residual electron density near the C2R atom reveals that the disordered fragments share very close crystallographic positions (Fig. S120). High-quality diffraction data allowed for the unconstrained refinement of both fragments, resulting in a solid-state distribution of 79.0(7)% CH_2(cyclo-C_3H_5) and 21% *n*-but-3-en.

In solution, however, ^{1}H NMR spectroscopy indicates a slightly different distribution, with a ratio of 85:15% CH_2(cyclo-C_3H_5):*n*-but-3-en. This discrepancy suggests that the relative solubility of the two products influences their content in the solid state.

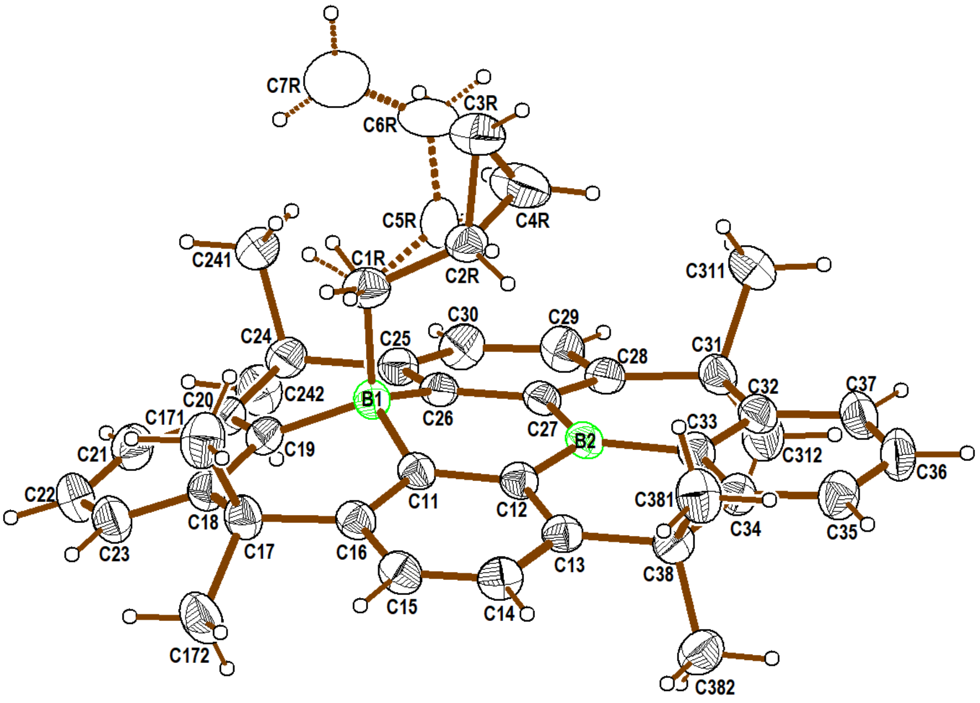

Figure S120: Structure of the anion in [Li(12-c-4)$_2$][**7**]$_{0.79}$[**8**]$_{0.21}$•2THF, atomic displacement ellipsoids at 50% probability level. The minor component of the disorder is shown by empty ellipsoids and dashed lines. The [Li(12-*crown*-4)$_2$]$^+$ cation is omitted for clarity.

6.12 Single-crystal X-ray structure analysis of [Li(thf)$_4$][**8**]

The ionic compound [Li(thf)$_4$][**8**] crystallizes in the monoclinic space group $P2_1/c$ (No. 14). Both the conformationally disordered tetrahedral [Li(THF)$_4$]$^+$ cation and the DBA-based anion occupy general positions (Fig. S121).

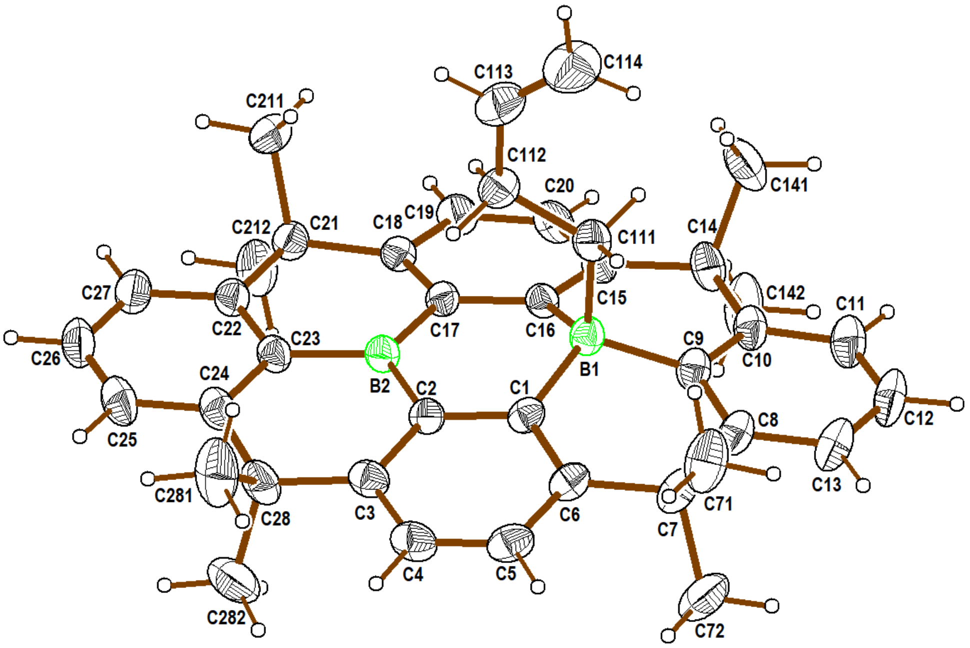

Figure S121: Structure of the anion in [Li(thf)$_4$][**8**], atomic displacement ellipsoids at 50% probability level. The disordered [Li(thf)$_4$]$^+$ cation is omitted for clarity.

6.13 Single-crystal X-ray structure analysis of [K(2.2.2crypt)][**1**]•2THF

The ionic compound [K(2.2.2crypt)][**1**]•2THF crystallizes with one [K(2.2.2-*crypt*)]$^+$ cation and two THF solvent molecules in the triclinic space group $P\overline{1}$ (No. 2). Two crystallographically unique DBA-radicals [**1**]$^{\bullet-}$ are positioned at the inversion centers of the space group (Fig. S122). One of the THF solvent molecules is conformationally disordered. Both radicals have almost identical geometrical parameters. Despite the fact that the [K(2.2.2crypt)][**1**]•2THF crystal structure is determined with much better accuracy than [Li(12-c-4)$_2$][**1**], the corresponding B−C and C−C bond lengths in both structures are very close to each other. The respective standard deviations of the corresponding bonds are comparable in both structural determinations. However, the conformation of [**1**]$^{\bullet-}$ in the K$^+$ salt differs slightly from that observed in the Li$^+$ salt, [Li(12-c-4)$_2$][**1**] (Fig. S123). This fact can be explained by crystal packing effects.

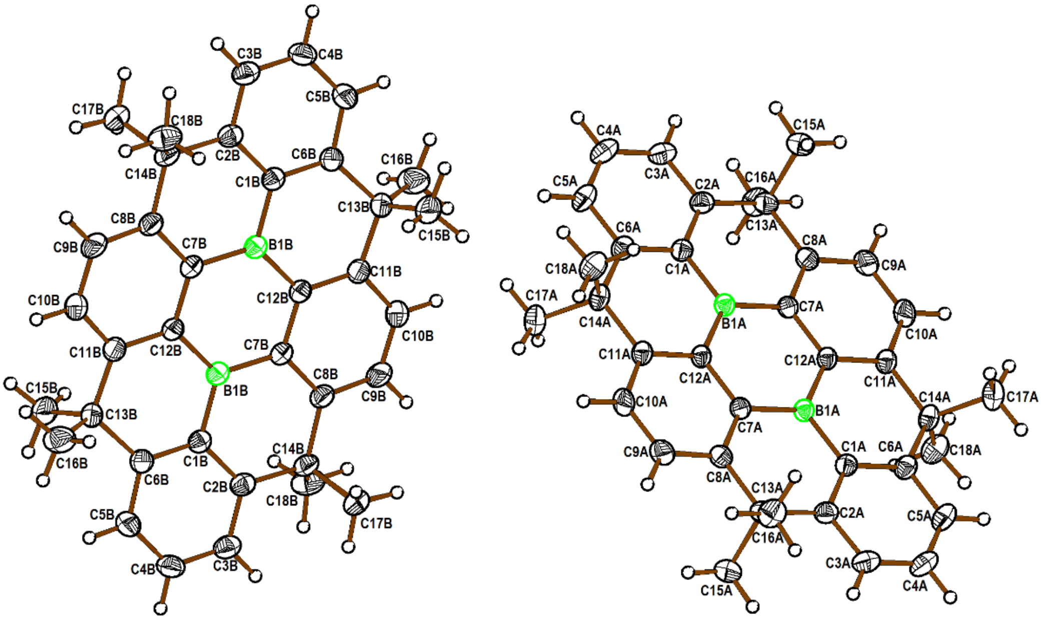

Figure S122: Molecular structure of two crystallographically unique DBA-radicals in [K(2.2.2crypt)][**1**]•2THF (atomic displacement parameters at 50% probability level; countercations omitted for clarity).

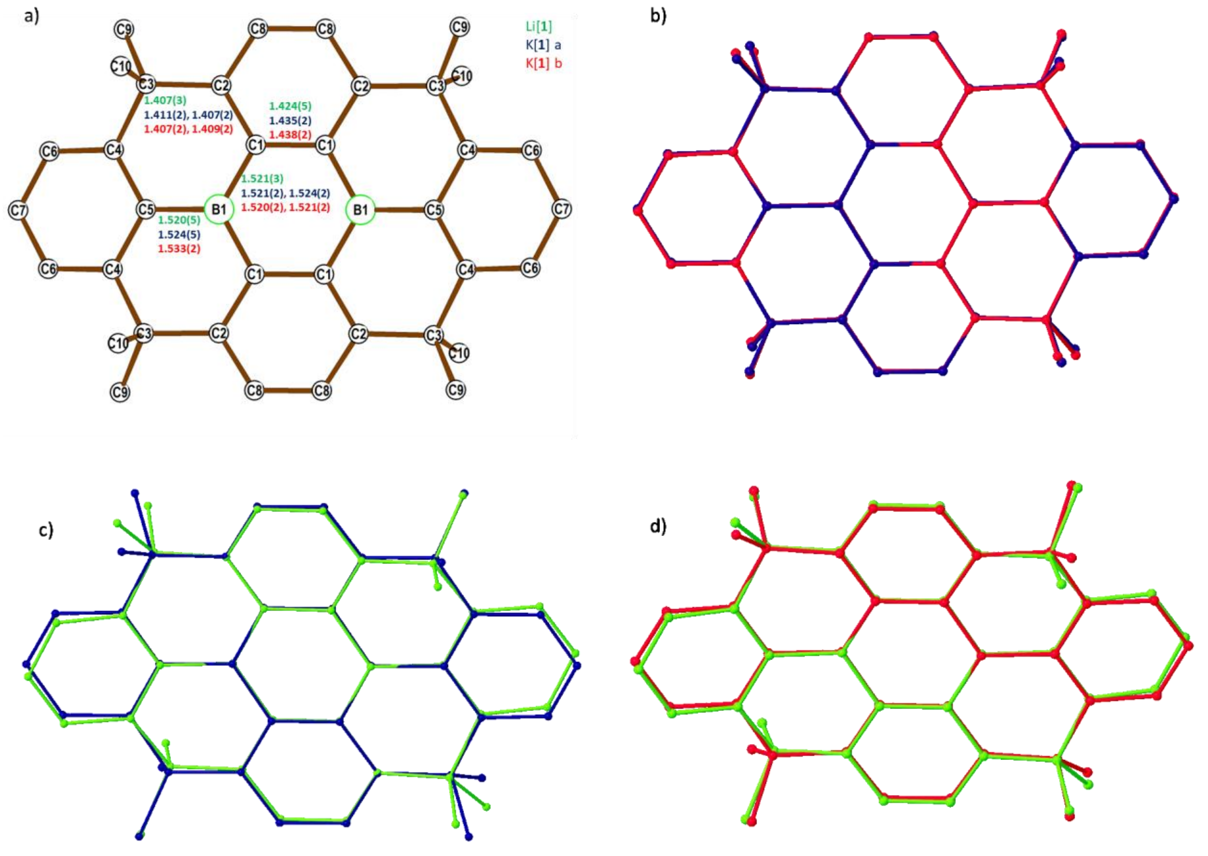

Figure S123: Interatomic distances and molecular conformations of $[\mathbf{1}]^{\bullet-}$ in $[Li(12\text{-}c\text{-}4)_2][\mathbf{1}]$ (green) and in the two crystallographically unique radicals $[\mathbf{1}^{a}]^{\bullet-}$ (blue) and $[\mathbf{1}^{b}]^{\bullet-}$ (red) in [K(2.2.2crypt)][**1**]•2THF. (a) Key interatomic distances. Overlays of (b) $[\mathbf{1}^{a}]^{\bullet-}$ (blue) and $[\mathbf{1}^{b}]^{\bullet-}$ (red), (c) $[\mathbf{1}^{a}]^{\bullet-}$ (blue) and $[\mathbf{1}]^{\bullet-}$ (green), (d) $[\mathbf{1}^{b}]^{\bullet-}$ (red) and $[\mathbf{1}]^{\bullet-}$ (green).

7 Computational Details

All DFT calculations were performed using *Gaussian 16, Revision B.01*.[S16] Graphical representations of molecular geometries were produced with the *CYLview20* software.[S17] Graphical representations of frontier orbitals (HOMO/LUMO) and natural bond orbitals were produced with GaussView 6.0.16.[S18]

To determine appropriate methods for optimizing the structures investigated herein, we performed geometry optimizations of Li_2[**1**] considering five distinct DFT functionals including implicit solvation by the solvent model based on density (SMD; solvent = THF; ε = 7.4257)[S20], namely B3LYP[S20]-D3(BJ)[S21], BP86[S23,S24]-D3(BJ), M062X[S24]-D3, PBE0[S25,26]-D3(BJ) and ωB97X-D.[S27] The Ahlrich's def2-SVP basis set was used in these calculations.[S28] The optimized results were compared to the respective X-ray crystal structures [Li_2(thf)$_4$][**1**]. A comparison of the root-mean-square deviation (RMSD) values, which were obtained excluding hydrogen atoms, are shown in Table S19. Overall, SMD(THF)/ωB97X-D/def2-SVP performed best among the tested theory levels. Optimized geometries were confirmed to be the desired minimum energy structures or transition states by vibrational frequency analysis.

Table S19: DFT functionals used for the benchmarking and the respective RMSD values derived by comparison with the crystal structure of Li_2[**1**].

Compound	B3LYP-D3(BJ)	BP86-D3(BJ)	M062X-D3	PBE0-D3(BJ)	ωB97X-D
Li_2[**1**]	0.2254	0.2287	0.2293	0.2292	0.2228

Single-point energy calculations at the SMD(THF)/ωB97X-D/def2-QZVP level were conducted to compute the free energies of the species along the proposed mechanistic pathway. This functional was selected to maintain consistency with the level of theory used for geometry optimization, and because ωB97X-D is known to perform well for systems with similar characteristics.[S6,S30,S31] All free energy values were calculated for the corresponding experimental temperature (25 °C) and included a concentration correction accounting for the change in standard states going from gas phase to condensed phase.[S32,S33]
Note: Geometry optimization of the radical anion $[C_6F_6]^{\bullet-}$ using the best-performing method from the benchmark studies resulted in a pseudo-Jahn-Teller distorted structure with asymmetric distortion (C_s). In this structure, the two C–F bonds protruding from the plane adopt different tilt angles. In contrast, the literature-reported structure exhibits C_{2v} symmetry with equal tilt angles (chapter 7.1.2).[S34,S35] Since the previously published calculations were performed almost exclusively to model the gas phase, we omitted the solvent model in our best-performing theory level and afterwards reproduced the literature-reported symmetric structure. Furthermore, this symmetric structure was also reproduced using other computational methods reported in the literature.[S34,S35] The energy difference between the two structures is well below 1 kcal/mol, supporting our continued use of the benchmarked method for calculating the entire hydrodefluorination mechanism and the free enthalpies (ΔG). To ensure completeness and to demonstrate that the electronic structure remains qualitatively unchanged, the NBO analysis of the radical anion $[C_6F_6]^{\bullet-}$ was conducted for both the literature-reported C_{2v}-symmetric and the C_S-symmetric structure (see chapter 7.2.4).

7.1 Frontier orbitals of **1**, [**1**]$^{\bullet-}$, [**1**]$^{2-}$, fluorobenzenes and fluorobenzene radical anions

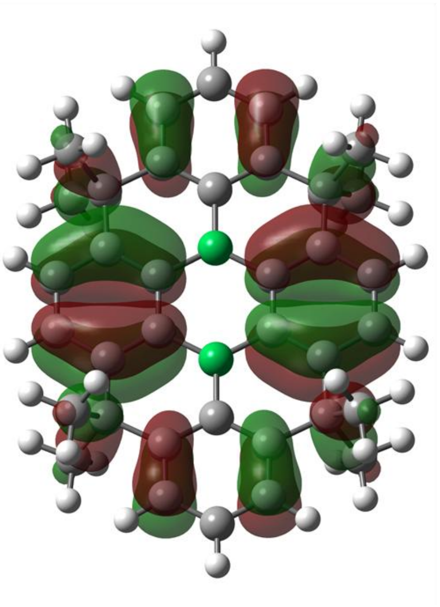

HOMO **1**

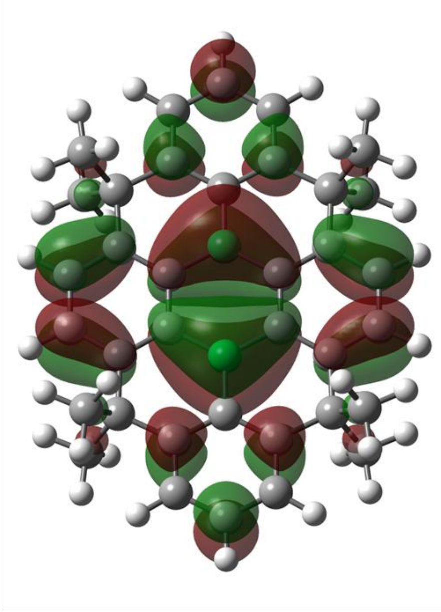

LUMO **1**

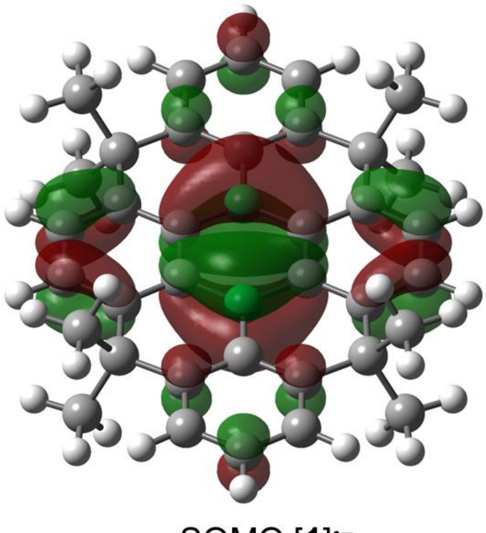

SOMO [**1**]$^{\bullet-}$

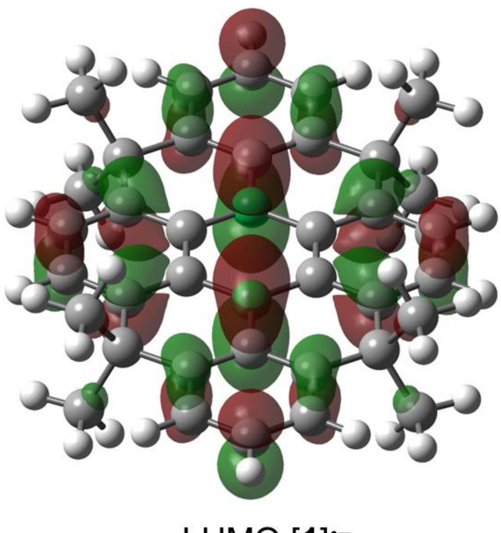

LUMO [**1**]$^{\bullet-}$

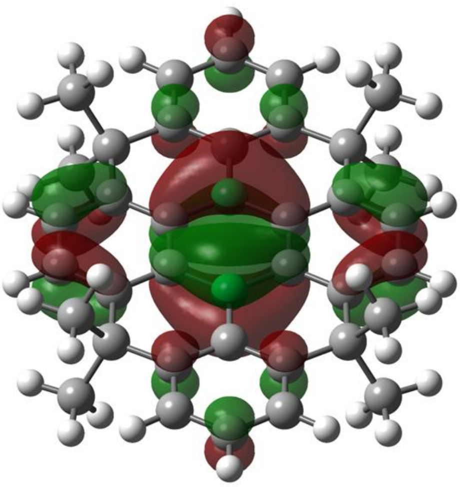

HOMO [**1**]$^{2-}$

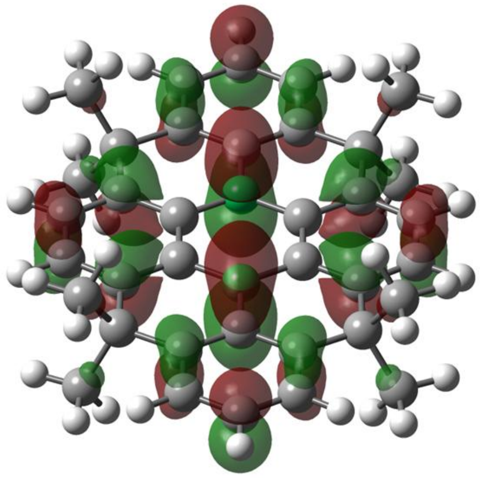

LUMO [**1**]$^{2-}$

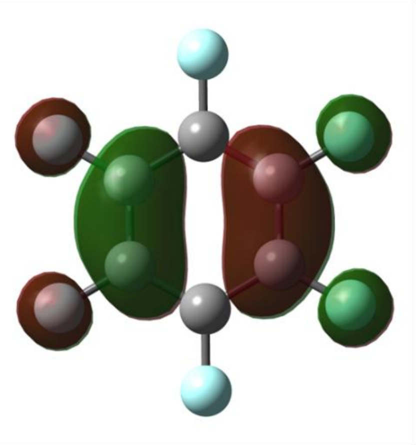

HOMO C_6F_6

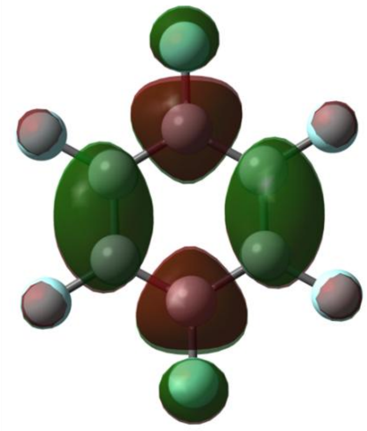

LUMO C_6F_6

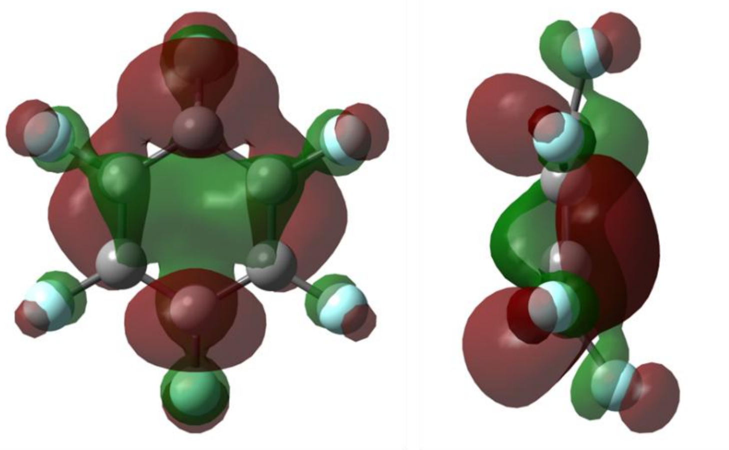

SOMO $[C_6F_6]^{\bullet-}$

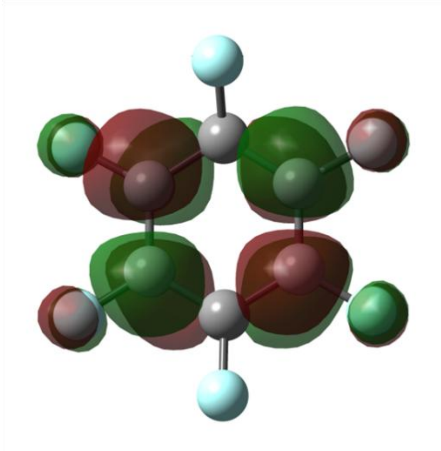

LUMO $[C_6F_6]^{\bullet-}$

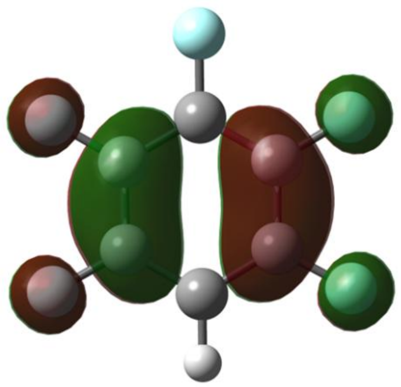

HOMO C_6F_5H

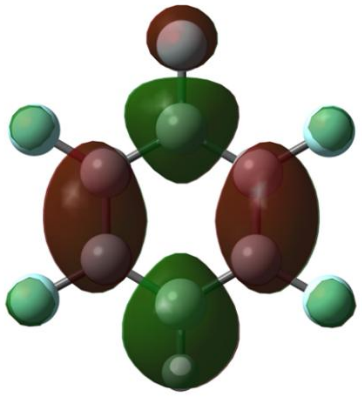

LUMO C_6F_5H

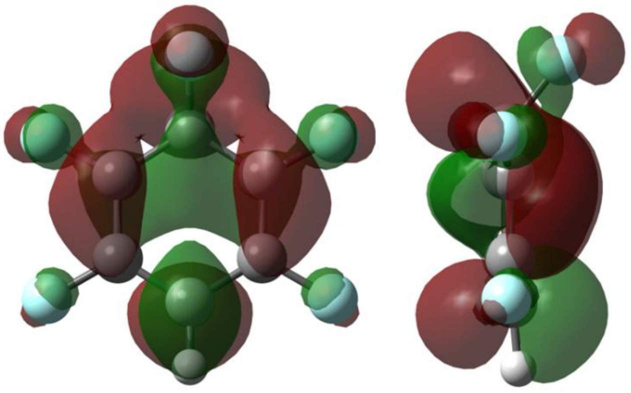

SOMO $[C_6F_5H]^{\bullet -}$

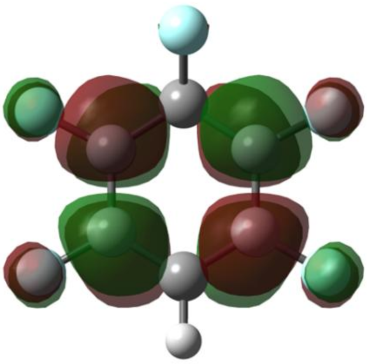

LUMO $[C_6F_5H]^{\bullet -}$

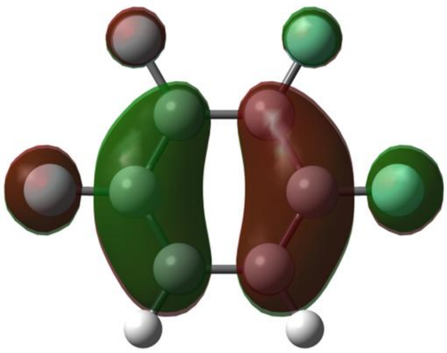

HOMO 1,2,3,4-$C_6F_4H_2$

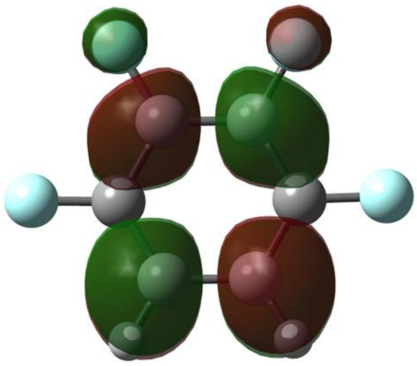

LUMO 1,2,3,4-$C_6F_4H_2$

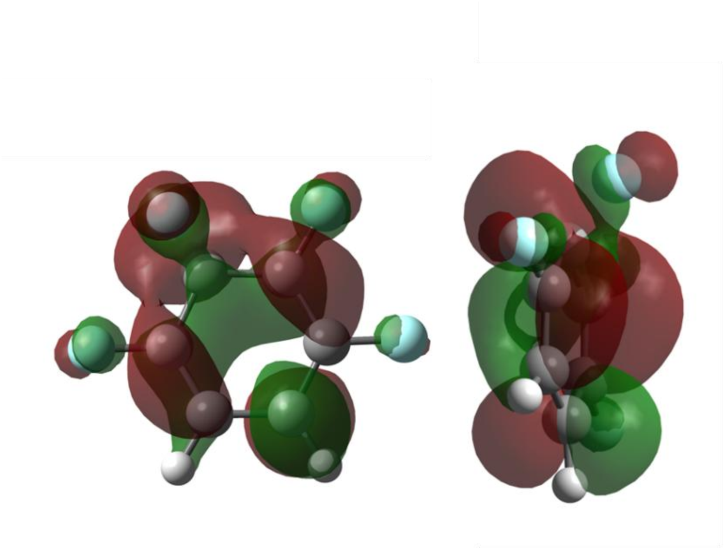

SOMO $[1,2,3,4\text{-}C_6F_4H_2]^{\bullet-}$

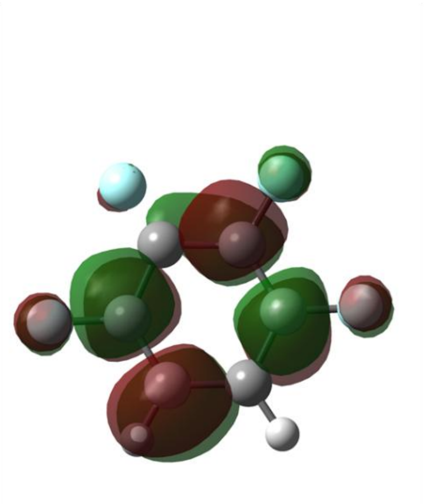

LUMO $[1,2,3,4\text{-}C_6F_4H_2]^{\bullet-}$

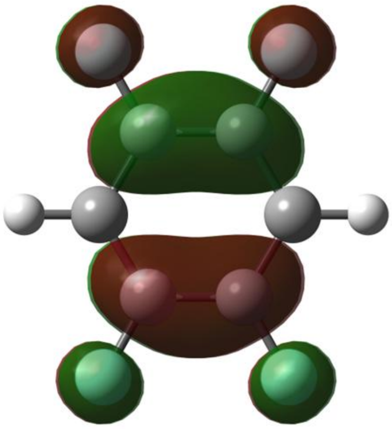

HOMO 1,2,4,5-$C_6F_4H_2$

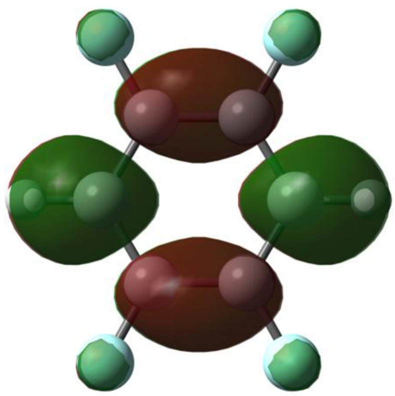

LUMO 1,2,4,5-$C_6F_4H_2$

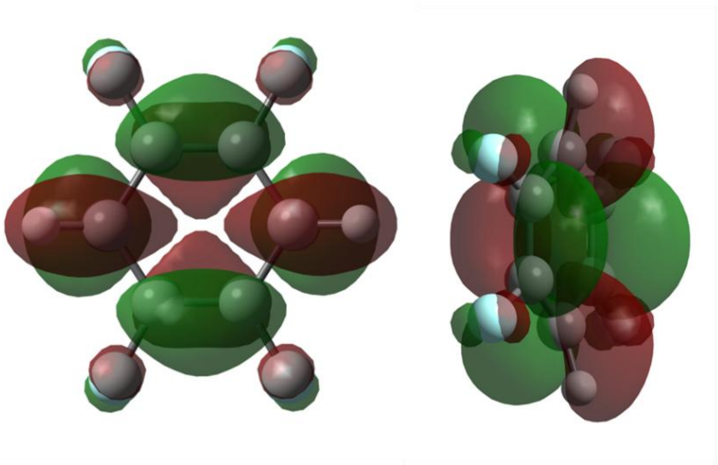

SOMO $[1,2,4,5\text{-}C_6F_4H_2]^{\bullet-}$

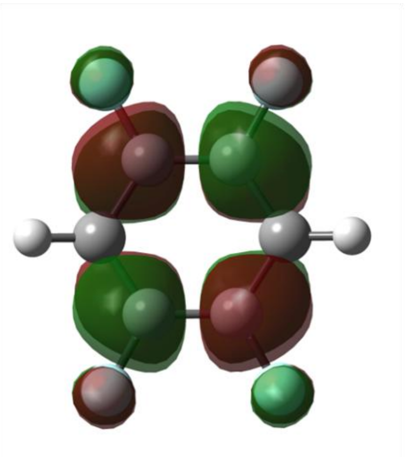

LUMO $[1,2,4,5\text{-}C_6F_4H_2]^{\bullet-}$

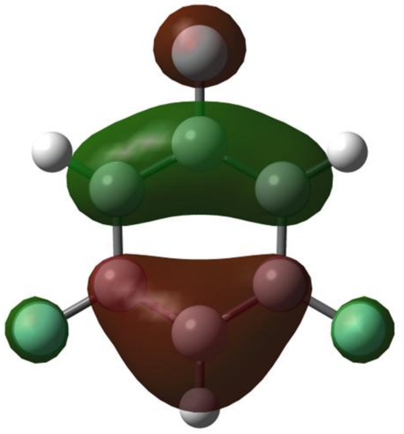

HOMO 1,3,5-$C_6F_3H_3$

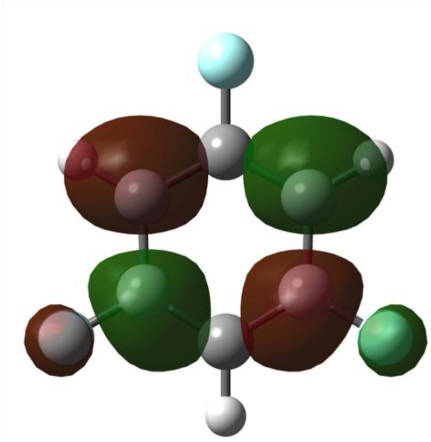

LUMO 1,3,5-$C_6F_3H_3$

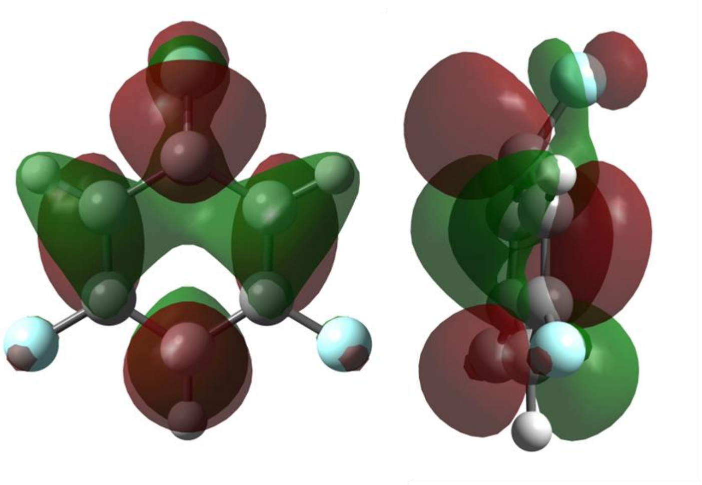

SOMO [1,3,5-$C_6F_3H_3$]$^{\bullet-}$

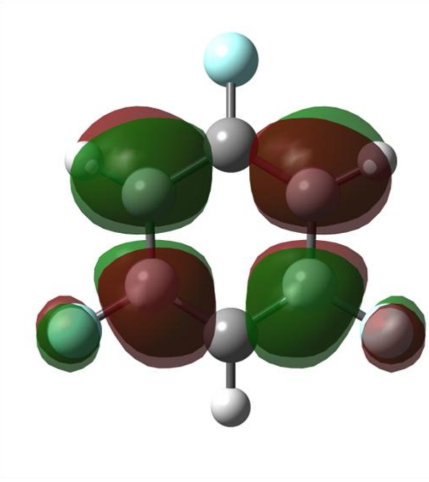

LUMO [1,3,5-$C_6F_3H_3$]$^{\bullet-}$

7.1.1 Spin density of [**1**]$^{•−}$

The spin density (α − β SCF density) of the radical [**1**]$^{•−}$ is shown below, visualized at an isovalue of 0.0006 *e* a_0^{-3}. The distribution of unpaired electron density is represented using two distinct colors: green corresponds to regions of excess α density, while blue represents regions of excess β density. The odd electron is delocalized throughout the entire framework of [**1**]$^{•−}$. Small contributions of α spin density at the methyl groups indicate that coupling to the methyl H atoms (as observed in the EPR spectrum) is indeed possible.

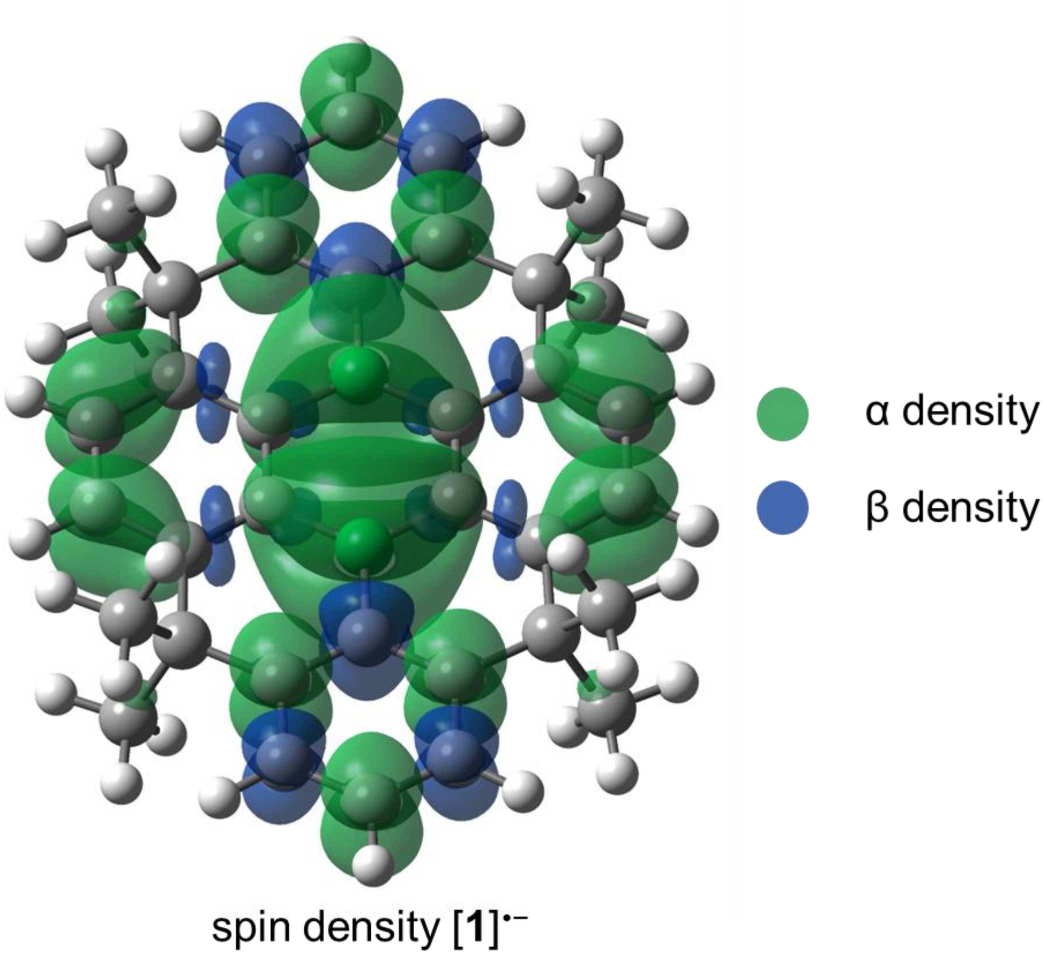

Figure S124: Computed spin density of the radical anion [**1**]$^{•−}$. Level of theory: SMD(THF)/ωB97X-D/def2-SVP; isovalue = 0.0006 *e* a_0^{-3}.

7.1.2 Distorted structures of fluorobenzene radical anions

All fluorobenzene radical anions relevant to this work exhibit a geometric distortion, which is characteristic of a pseudo-Jahn-Teller effect.[S35] Both side and top views of the respective anions are presented below.

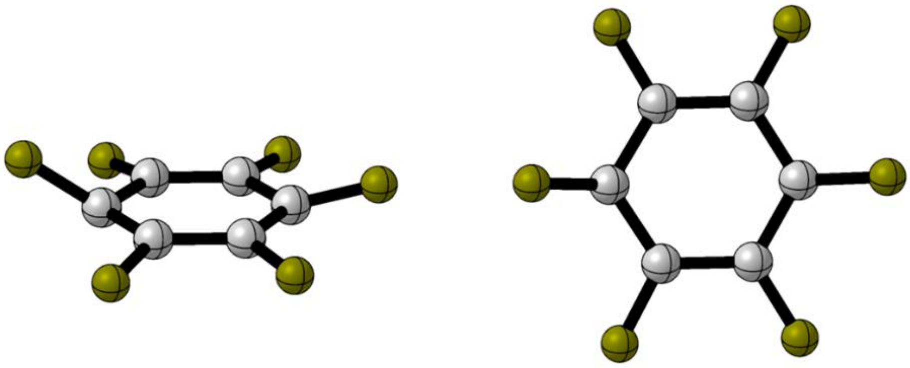

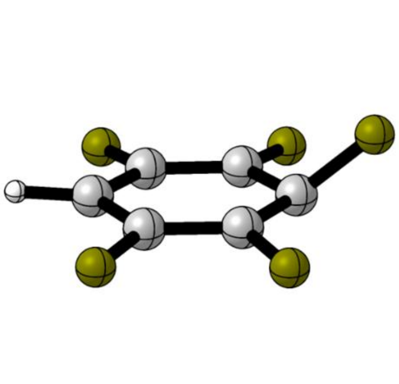

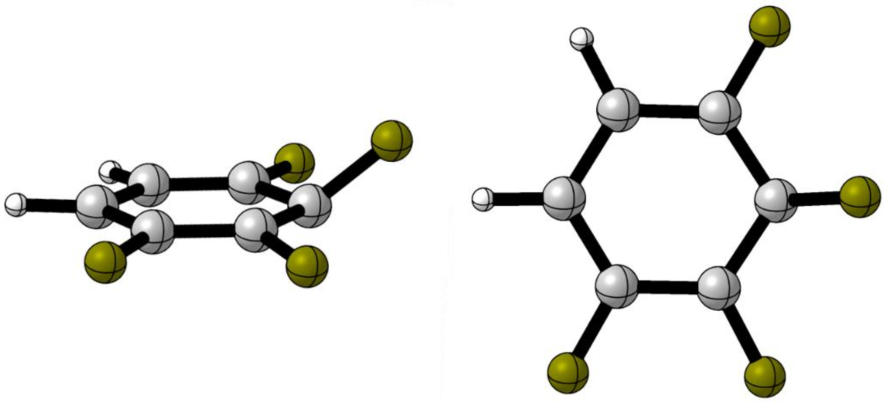

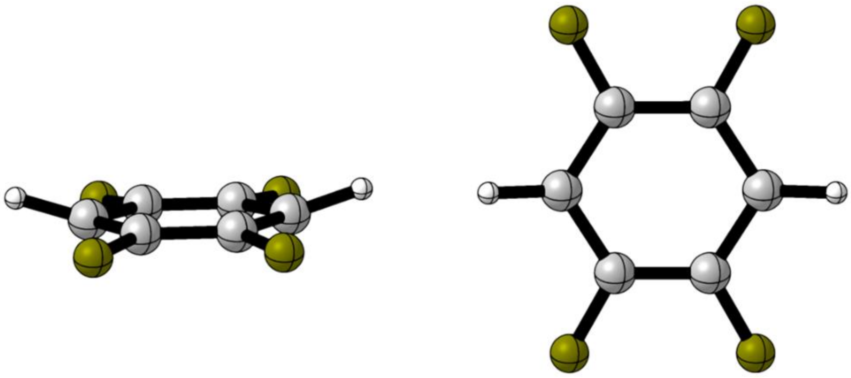

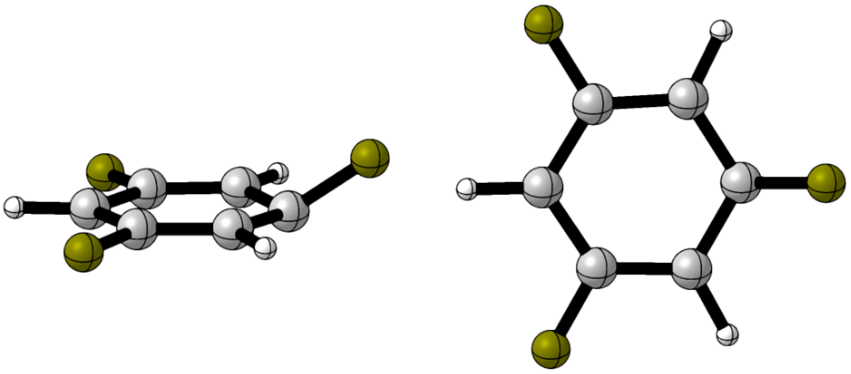

7.2 Mechanism of the hydrodefluorination reaction

7.2.1 Gibbs free energy values (ΔG_{SET}) for the SET from [**1**]$^{2-}$ to $C_6F_nH_{6-n}$

To investigate the observed trend of the reactivity change from open to closed shell as a function of the degree of fluorination in more detail, the free enthalpy of the first step was calculated. The reactants ([**1**]$^{2-}$ and fluorobenzene) were set to the relative energy 0.0 kcal/mol and the ΔG_{SET} of a hypothetical single-electron transfer (SET) to the respective fluorobenzene was calculated. This confirmed the trend found in the experiments: The formation of the π-radicals is strongly exergonic, with ΔG_{SET} values of −6.5 kcal/mol for C_6F_6 and −4.3 kcal/mol for C_6F_5H, which aligns well with the observed reactions at room temperature. In the case of 1,2,3,4- and 1,2,4,5-$C_6F_4H_2$, the electron transfer proceeds with ΔG_{SET} values of 0.5 kcal/mol and 4.0 kcal/mol, respectively. The reaction of [**1**]$^{2-}$ with 1,2,3,5-$C_6F_4H_2$ is endergonic (4.7 kcal/mol), and the formation of the radical anion of 1,3,5-$C_6F_3H_3$ is the most energetically unfavorable among the systems investigated, with a ΔG_{SET} of +9.5 kcal/mol. In these cases, the reactivity shifts to nucleophilic aromatic substitution.

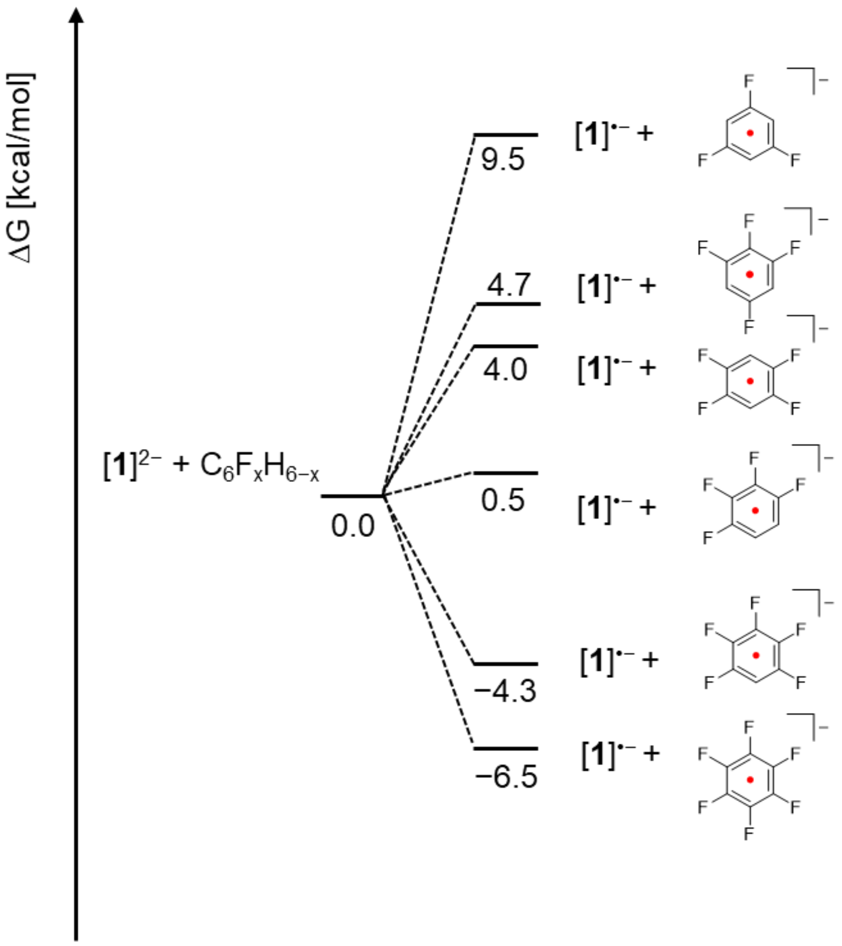

Figure S125: Comparison of ΔG_{SET} values for the SET proceeding from [**1**]$^{2-}$ to different fluorobenzenes.

7.2.4 Natural bond orbital calculations of $[C_6F_6]^{\bullet-}$

We performed natural bond orbital (NBO) calculations[S37–S39] to gain deeper insights into the electronic structure of the $[C_6F_6]^{\bullet-}$ radical anion. As previously mentioned, our benchmarked method gave a slightly different structure for $[C_6F_6]^{\bullet-}$ compared to the one reported in the literature.[S35,S40] The published structure exhibits a symmetric pseudo-Jahn-Teller distortion, whereas our computed structure is asymmetrically distorted (C_{2v} vs. C_S). Since the energy difference between both structures is less than 1 kcal/mol, we performed NBO calculations for both to ensure a comprehensive analysis.

In both cases, analysis of the α-spin density revealed a C_6 ring with two π bonds and two nonbonding SOMOs with lone pair character (Schemes S3 and S4). The two nonbonding SOMOs are located on the C atoms that carry the out-of-plane tilted C−F bonds, indicating sp^3 hybridization. Both NBO analyses show that the pseudo-Jahn-Teller distortion facilitates electron delocalization through hyperconjugation: (i) between the two nonbonding SOMOs and the π* orbitals of the formal C=C double bonds, and (ii) from the π orbitals into the σ* C−F antibonding orbitals. The greater the out-of-plane tilt of the two C−F bonds, the stronger the hyperconjugative interaction with the σ* orbital (cf. Tables S19 and S20). These results suggest that (a) the odd electron in the C_6F_6 radical anion is fully delocalized, and (b) the pseudo-Jahn-Teller distortion enables electron transfer into the σ* C−F bonds, ultimately facilitating fluoride elimination in the subsequent step.

7.2.4.1 Symmetrically distorted structure of $[C_6F_6]^{\bullet-}$: NBO calculations

The literature known structure of $[C_6F_6]^{\bullet-}$ was reproduced.[S35,S40] The respective NBO analysis was performed at the UωB97X-D/def2-TZVPP level of theory.

We found a C_6 ring containing two π bonds (C_2=C_3 and C_5=C_6) and two nonbonding SOMOs with lone pair character (SOMO C_1 and C_4) (Scheme S3). The C_1−F_1 and C_4−F_4 bonds are tilted out of the C_6 plane by the same angle, facilitating the overlap of the respective σ* orbitals with the two π bonds. Both lone pairs exhibit hyperconjugation into the respective π* bonds. The energy values associated with these hyperconjugations are provided in Table S19.

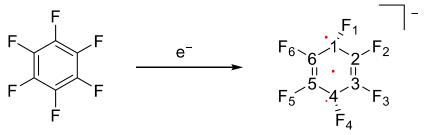

Scheme S3: Structure of the symmetrically distorted $[C_6F_6]^{\bullet-}$ based on the NBO calculations.

Table S20: E(2) values for donor acceptor interactions derived from NBO calculations on $[C_6F_6]^{\bullet-}$. Level of theory: UωB97X-D/def2-TZVPP.

donor	acceptor	E(2) [kcal/mol]
SOMO C_1	π* C_2=C_3	21
SOMO C_1	π* C_5=C_6	21
SOMO C_4	π* C_2=C_3	21
SOMO C_4	π* C_5=C_6	21
π C_2=C_3	σ* C_1−F_1	5
π C_5=C_6	σ* C_1−F_1	5
π C_2=C_3	σ* C_4−F_4	5
π C_5=C_6	σ* C_4−F_4	5

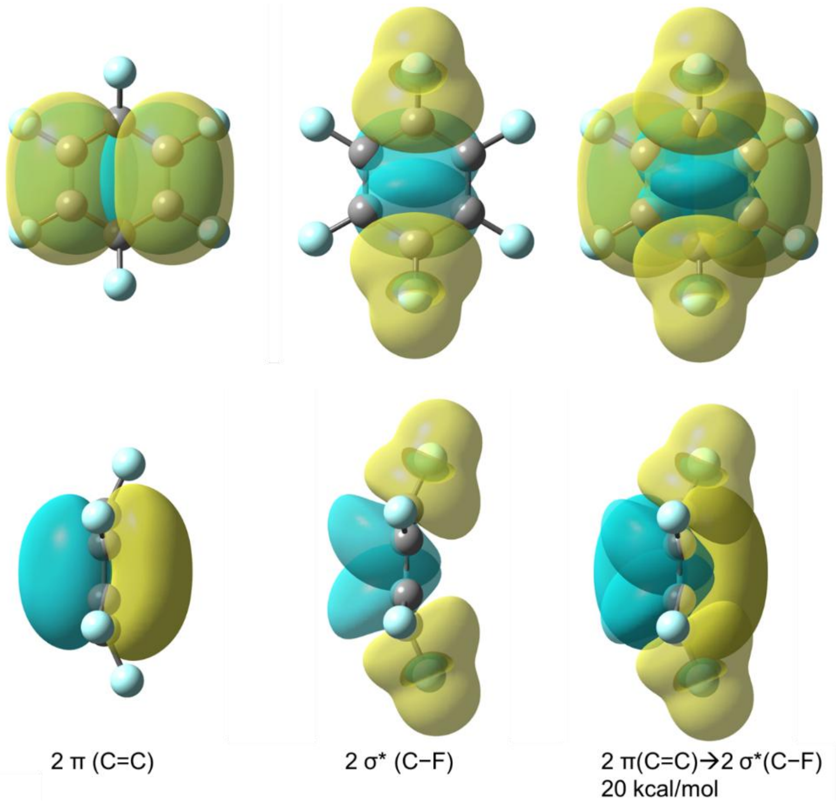

Figure S126: Top and side views of the donor (left; π C_2=C_3 and C_5=C_6) and acceptor orbitals (middle; σ* C_1−F_1 and C_4−F_4), as well as the corresponding donor-acceptor interaction (right), derived from Natural Bond Orbital (NBO) analysis.

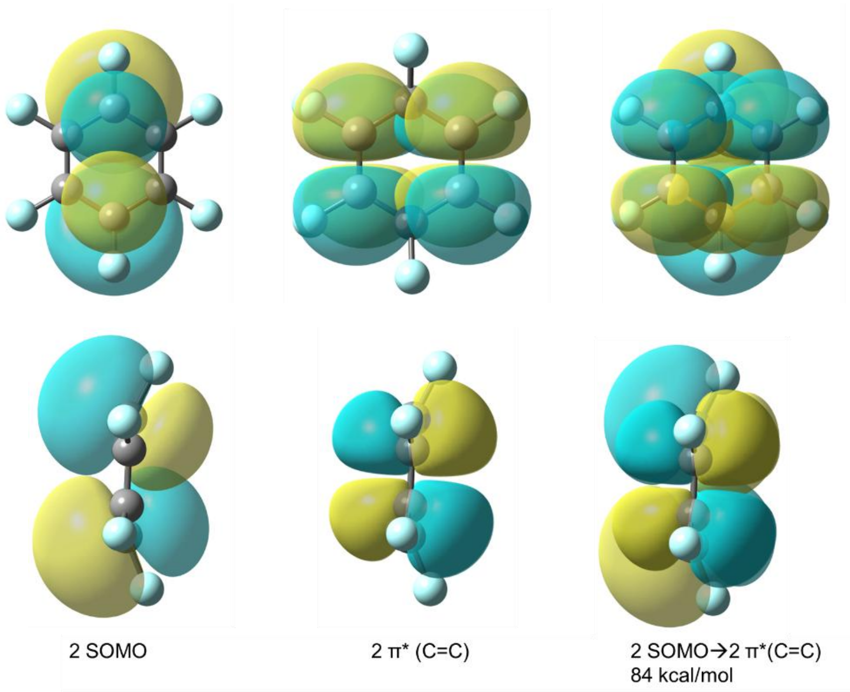

Figure S127: Top and side views of the donor (left; nonbonding SOMOs) and acceptor orbitals (middle; π* C_2=C_3 and C_5=C_6), as well as the corresponding donor-acceptor interaction (right), derived from Natural Bond Orbital (NBO) analysis.

7.2.4.2 Asymmetrically distorted structure of $[C_6F_6]^{\bullet-}$: NBO calculations

This NBO analysis was performed at the UωB97X-D/def2-SVP level of theory.

We found a C_6 ring containing two π bonds (C_2=C_3 and C_5=C_6), a doubly occupied lone pair (LP C_1) and a nonbonding SOMO with lone pair character (SOMO C_4) (Scheme S4). This explains why the two fluorine atoms tilt out of the C_6 plane to different extents, as the doubly occupied lone pair at C_1 occupies considerably more space than the nonbonding SOMO with lone pair character at C_4. Since the C_1−F_1 bond is therefore tilted further out of the plane, the overlap of its σ* orbital with the π orbitals is much better than the overlap between the π orbitals and the C_4−F_4 σ* orbital.

The energy values associated with these hyperconjugations are provided in Table S20.

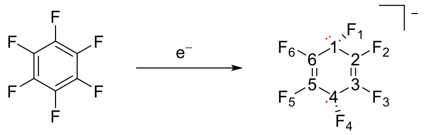

Scheme S4: Structure of the assymetrically distorted $[C_6F_6]^{\bullet-}$ based on the NBO calculations.

Table S21: E(2) values for donor acceptor interactions derived from NBO calculations on $[C_6F_6]^{\bullet-}$. Level of theory: UωB97XD/def2-SVP.

donor	acceptor	E(2) [kcal/mol]
LP C_1 (doubly occupied)	π* C_2=C_3	11
LP C_1 (doubly occupied)	π* C_5=C_6	11
SOMO C_4	π* C_2=C_3	42
SOMO C_4	π* C_5=C_6	42
π C_2=C_3	σ* C_1−F_1	8
π C_5=C_6	σ* C_1−F_1	8
π C_2=C_3	σ* C_4−F_4	2
π C_5=C_6	σ* C_4−F_4	2

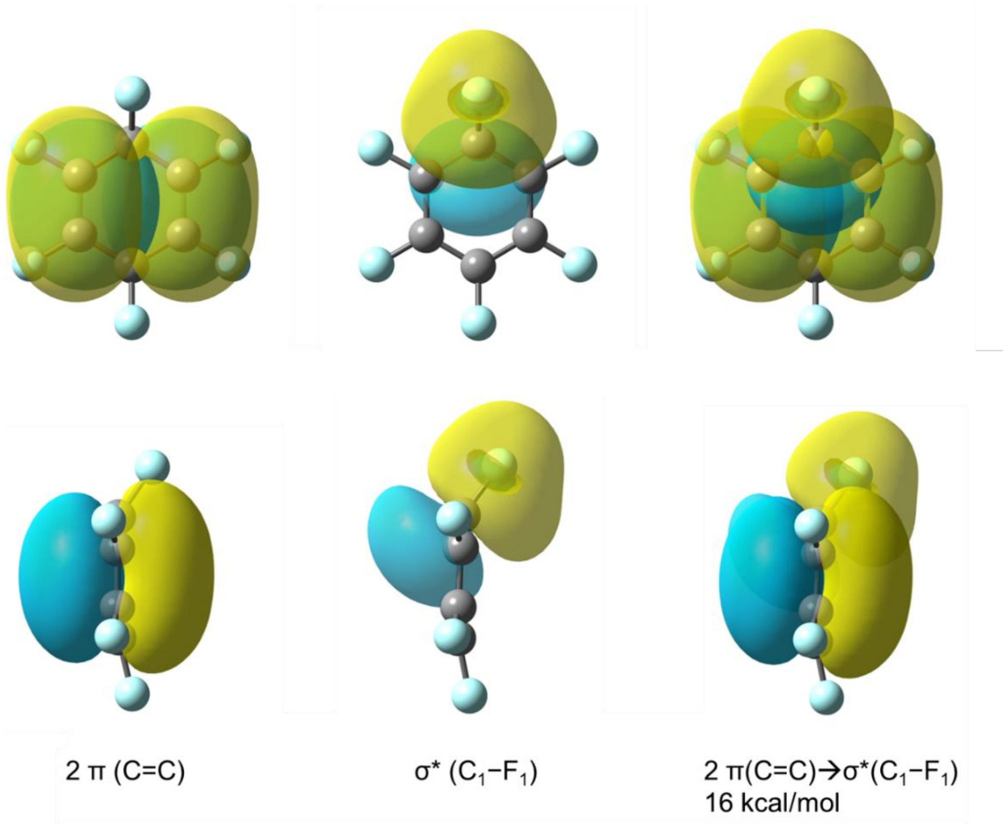

Figure S128: Top and side views of the donor (left; π C_2=C_3 and C_5=C_6) and acceptor orbitals (middle; σ* C_1−F_1), as well as the corresponding donor-acceptor interaction (right), derived from Natural Bond Orbital (NBO) analysis.

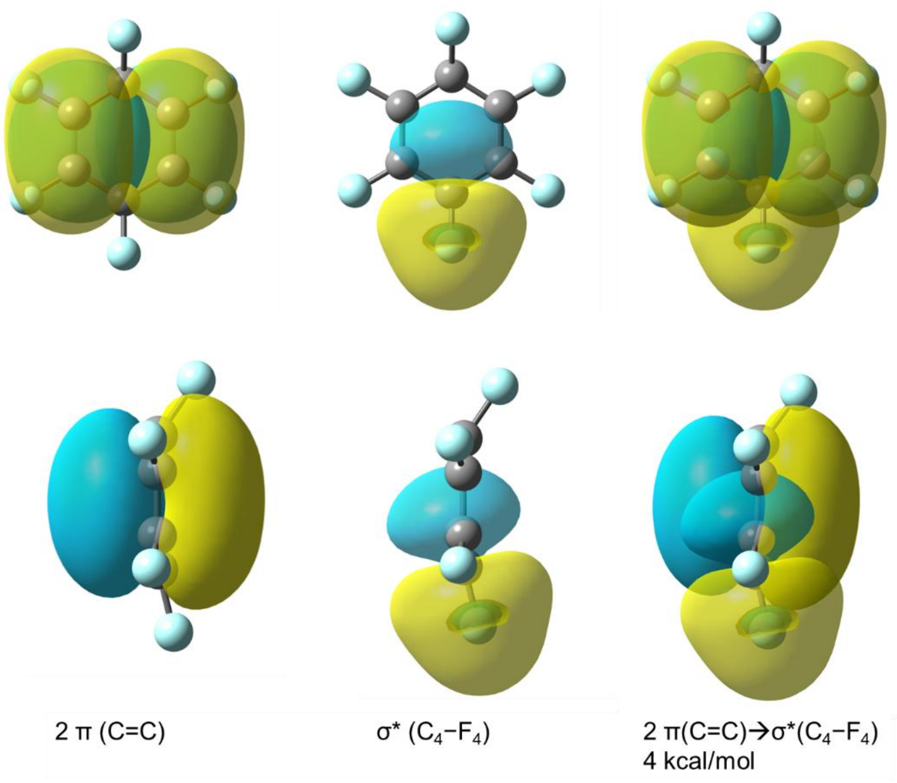

Figure S129: Top and side views of the donor (left; π C_2=C_3 and C_5=C_6) and acceptor orbitals (middle; σ* C_4−F_4), as well as the corresponding donor-acceptor interaction (right), derived from Natural Bond Orbital (NBO) analysis.

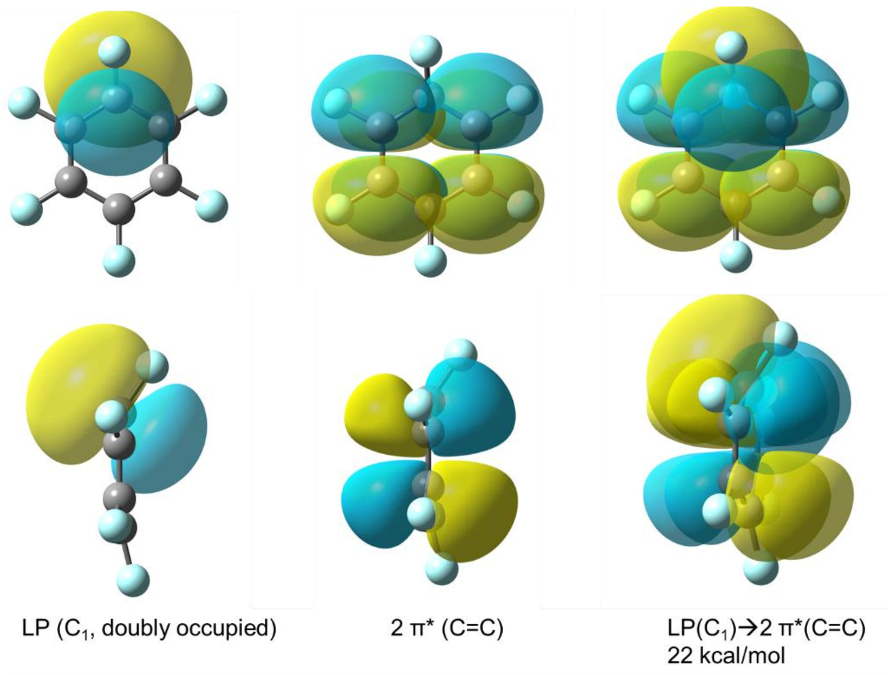

Figure S130: Top and side views of the donor (left; LP C_1) and acceptor orbitals (middle; π* C_2=C_3 and C_5=C_6), as well as the corresponding donor-acceptor interaction (right), derived from Natural Bond Orbital (NBO) analysis.

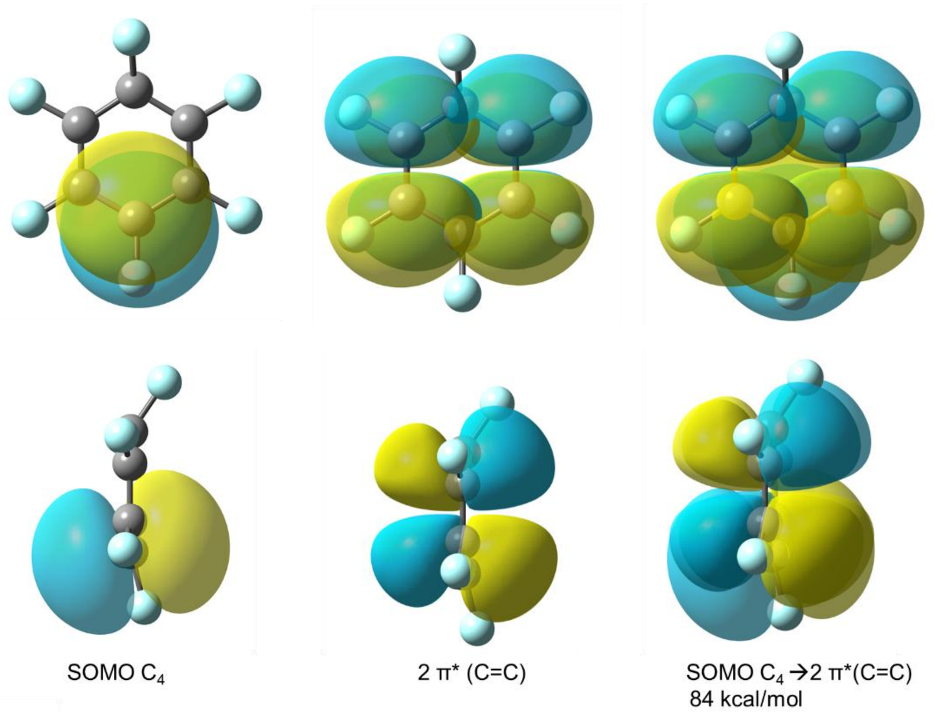

Figure S131: Top and side views of the donor (left; nonbonding SOMO) and acceptor orbitals (middle; π* C_2=C_3 and C_5=C_6), as well as the corresponding donor-acceptor interaction (right), derived from Natural Bond Orbital (NBO) analysis.

7.2.5 LiF-elimination

The next step of the mechanism involves LiF elimination from Li[C_6F_6] to obtain the neutral C_6F_5 sp^2 radical.

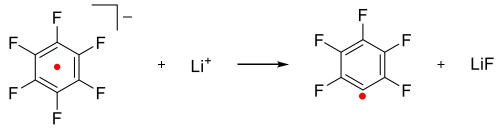

Scheme S5: The C_6F_6 radical anion interacts with a lithium cation under LiF elimination and the formation of a neutral sp$_2$ radical.

To achieve this, the preoptimized structure of [C_6F_6]$^{\bullet-}$ was used and a Li^+ cation was subsequently added at varying distances from the central ring. After geometry optimization, LiF was already eliminated. We then performed a systematic potential energy scan, varying the position of Li^+, and were able to locate a transition state (TS). A frequency analysis of the LiF elimination step revealed a TS with a large imaginary frequency (=134.5*i* cm^{-1}; Fig. S125). The resulting transition state (TS 1) was identified at a lower Gibbs free energy value ($\Delta G^{‡}$ = −15.8 kcal/mol) than the initial reactants, indicating the presence of a submerged TS.[S41,S42] However, this result aligns with the expectations, as the lithium cation stabilizes the anionic structure through strong Li^+···F interactions observed in the TS.

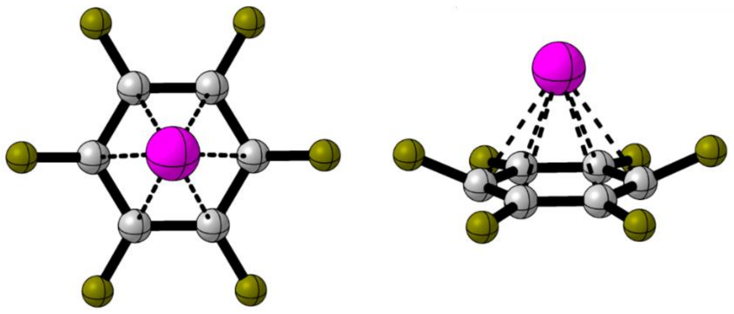

Figure S132: Optimized transition state for the LiF elimination. The imaginary frequency moves the Lithium cation from the left fluorine to the right fluorine, already indicating strong Li^+ - Fluorine interactions.

We then performed intrinsic reaction coordinate (IRC) calculations[S42] on the identified TS. For both the TS optimization and the IRC calculations, the use of an ultra-fine grid was necessary. Interestingly, the transition state found is bifurcated.[S42] Starting from the optimized transition state, we discovered that the same minimum structure is optimized regardless of whether the reaction coordinate is in the positive or negative direction. This indicates that the LiF elimination is facile, but that Li[C_6F_6] eliminates LiF on opposite sides (Fig. S126), leading to the formation of the same C_6F_5 sp^2 radical in either direction. This bifurcation highlights a unique aspect of the reaction pathway, suggesting that the elimination process can proceed via two equivalent but opposite routes, both converging to the same product.

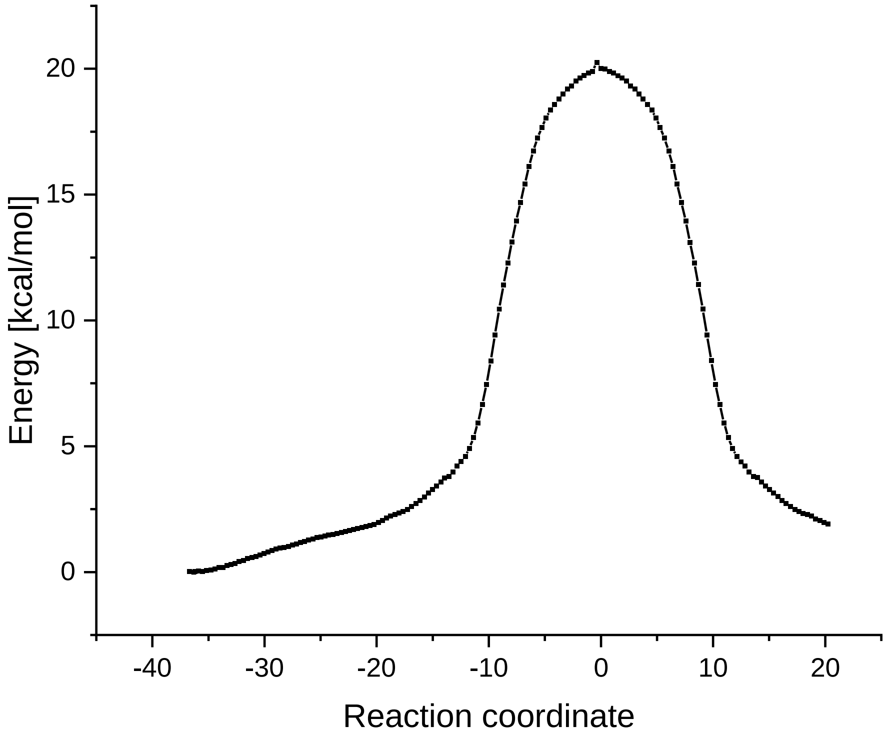

Figure S133: The IRC scan starting from the optimized transition state, yields LiF elimination on both sides respectively.

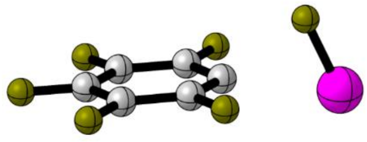

Figure S134: LiF elimination yields the planarized C_6F_5 sp^2 radical.

7.2.6 H·-abstraction

The following step involves hydrogen atom abstraction from the highly reactive C_6F_5 sp^2 radical using THF as an effective hydrogen atom transfer reagent and generating C_6F_5H and the resonance stabilized THF radical.[S33,S34]

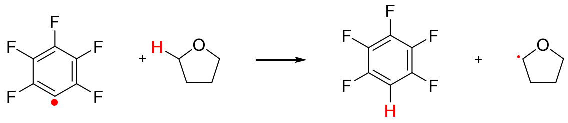

Scheme S6: The C_6F_5 radical abstracts an H atom from the solvent THF.

The preoptimized C_6F_5 radical and a THF molecule were placed in close proximity and optimized under constraints, giving the structure from which the TS for hydrogen atom abstraction could be identified after reoptimization. Using an ultra-fine grid was again crucial. The TS exhibits a high imaginary frequency of 189.1*i* cm^{-1}.

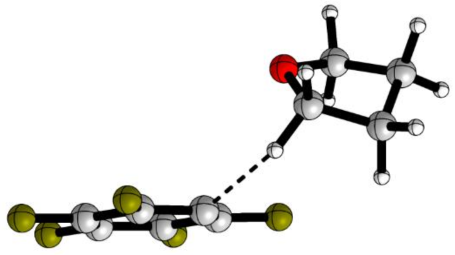

Figure S135: Transition state for hydrogen abstraction

Performing the IRC scan starting from the optimized transition state reveals the migration of a hydrogen atom located in ortho position to THF's oxygen atom (Figure S131). This process ultimately yields the desired THF radical and C_6F_5H as the energetic minimum of this reaction.

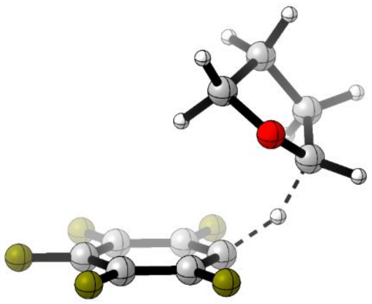

Figure S136: The C_6F_5 sp^2 radical abstracts a hydrogen atom from a THF molecule.

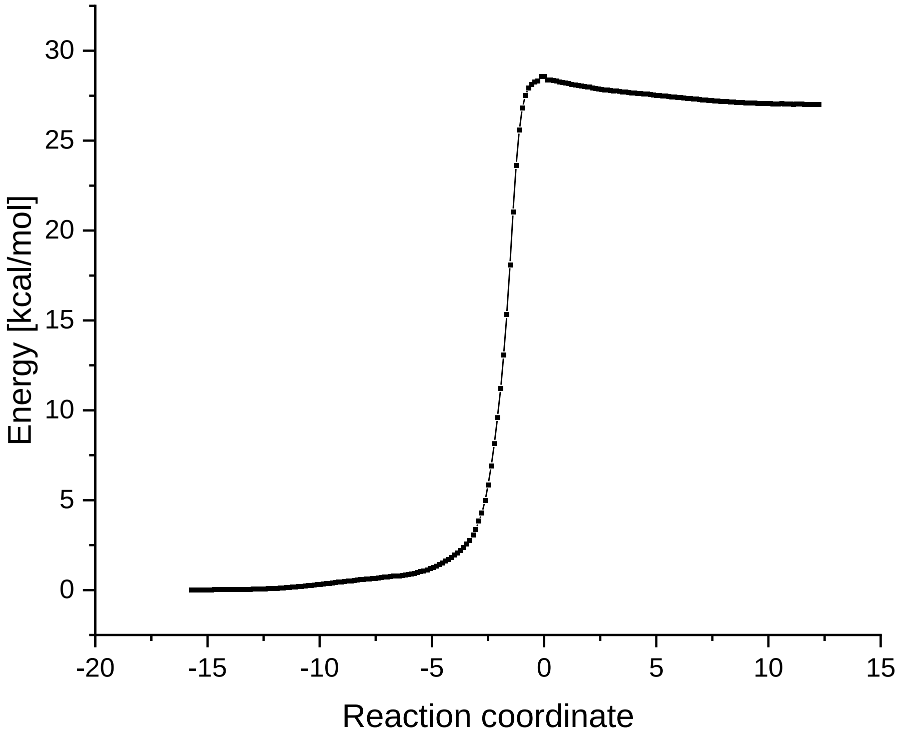

Figure S137: IRC scan for the hydrogen abstraction by the C_6F_5 radical.

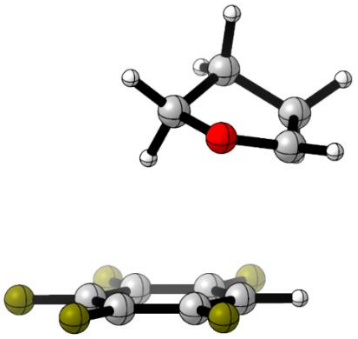

Figure S138: Energetic minimum for the performed IRC scan.

7.2.7 Recombination of the THF radical with [**1**]$^{\bullet-}$ or [**1**]$^{2-}$

The last step involves the recombination of the THF radical with either [**1**]$^{\bullet-}$ or [**1**]$^{2-}$ to yield [**3**]$^{-}$/[**3**]$^{\bullet 2-}$ in either its mono or dianionic form. To determine which recombination is more likely, the free enthalpies of the reactions were calculated. This helps to describe the tendency of each recombination pathway, provided that the two pathways differ significantly in terms of energy.

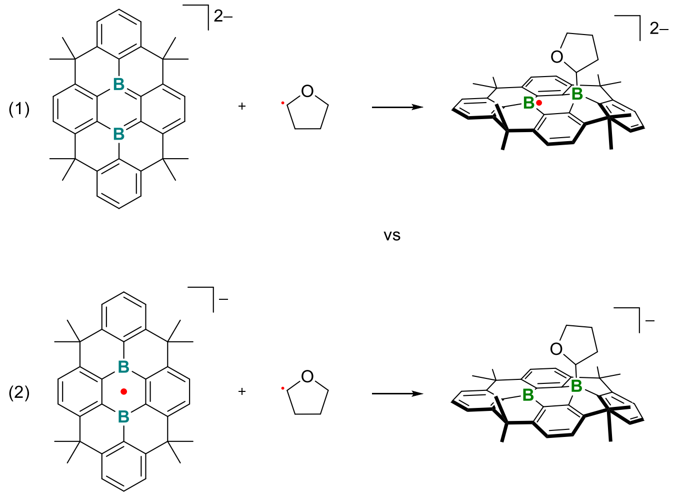

Figure S139: Two possible recombinations of the THF radical with either [**1**]$^{2-}$ or [**1**]$^{\bullet-}$.

The comparison of the ΔG values for both reactions showed that they are both exergonic:

(1) ΔG = −19.6 kcal/mol
(2) ΔG = −30.3 kcal/mol

However, the formation of the monoanionic DBA-*o*THF^{-} adduct however is favored by −11.7 kcal/mol. Therefore, it can be assumed that this is the preferred pathway for the reaction.

7.3 Summarized mechanism

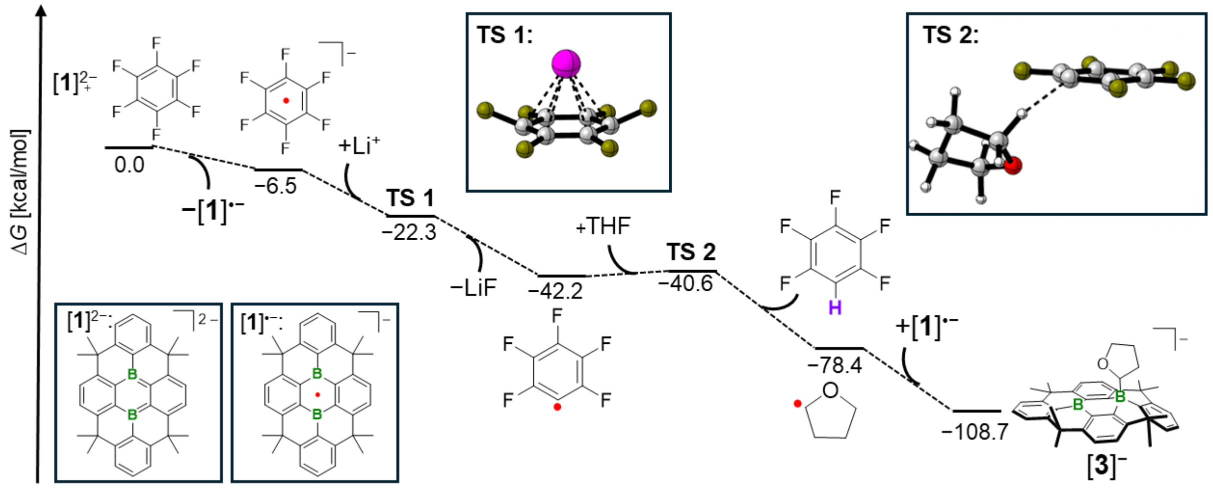

Figure S140: Summarized mechanism for the hydrodefluorination and the formation of [**3**]$^{-}$.

7.4 Computed structures and corrected free energies

1
***G*₂₉₈** = −1441.719073 Hartree

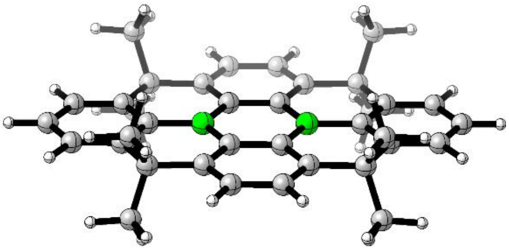

$[\mathbf{1}]^{\bullet-}$
$\boldsymbol{G_{298}}$ = −1441.807062 Hartree

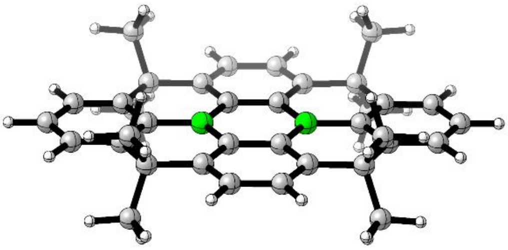

$[\mathbf{1}]^{2-}$
$\boldsymbol{G_{298}}$ = −1441.867048 Hartree

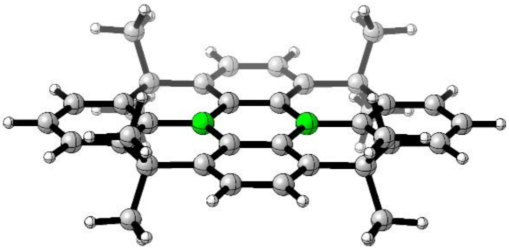

C_6F_6
$\boldsymbol{G_{298}}$ = −827.7540453 Hartree

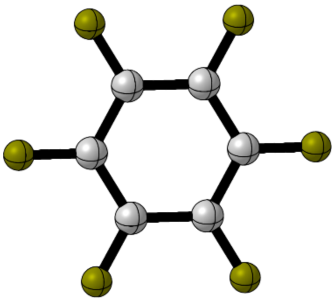

C_6F_5H
$\boldsymbol{G_{298}}$ = −728.502723 Hartree

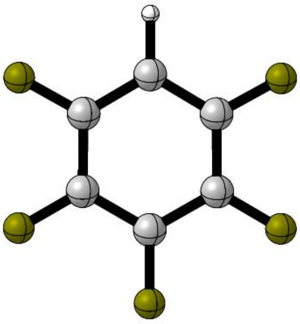

1,2,4,5-$C_6F_4H_2$
$\boldsymbol{G_{298}}$ = −629.2492167 Hartree

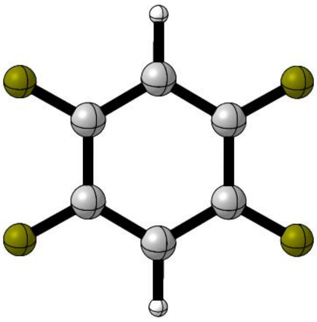

1,2,3,4-$C_6F_4H_2$
$\boldsymbol{G}_{298}$ = −629.2444253 Hartree

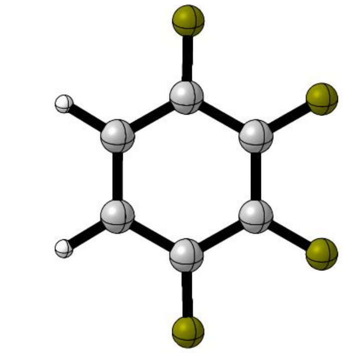

1,3,5-$C_6F_3H_3$
$\boldsymbol{G}_{298}$ = −529.9958916 Hartree

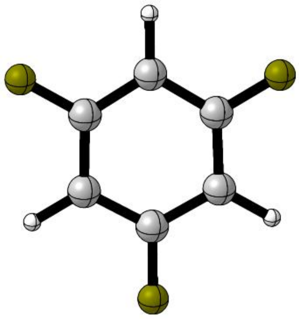

$[C_6F_6]^{\bullet-}$
$\boldsymbol{G}_{298}$ = −827.824432 Hartree

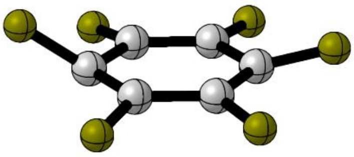

$[C_6F_5H]^{\bullet-}$
$\boldsymbol{G}_{298}$ = −728.5695089 Hartree

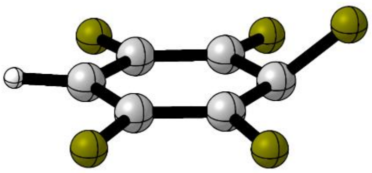

[1,2,4,5-$C_6F_4H_2]^{\bullet-}$
$\boldsymbol{G}_{298}$ = −629.3027744 Hartree

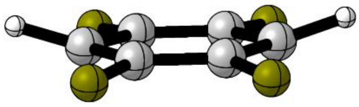

[1,2,3,4-$C_6F_4H_2]^{\bullet-}$
$\boldsymbol{G}_{298}$ = −629.3036921 Hartree

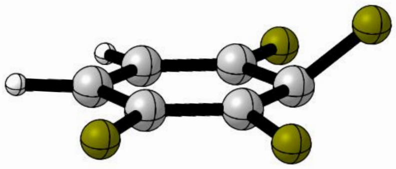

$[1,3,5\text{-}C_6F_3H_3]^{\bullet-}$
$\boldsymbol{G}_{298}$ = −530.040734 Hartree

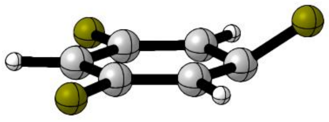

$[C_6F_5]^{\bullet}$
$\boldsymbol{G}_{298}$ = −727.7590297 Hartree

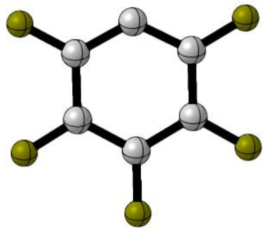

LiF
$\boldsymbol{G}_{298}$ = −107.4978396 Hartree

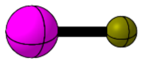

Li^{+}
$\boldsymbol{G}_{298}$ = −7.427403584 Hartree

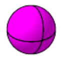

TS 1
$\boldsymbol{G}_{298}$ = −835.2771073 Hartree

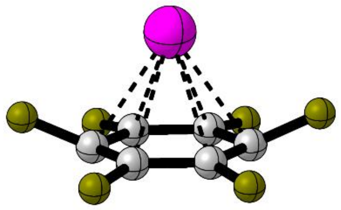

TS 2
$\boldsymbol{G}_{298}$ = −960.199856 Hartree

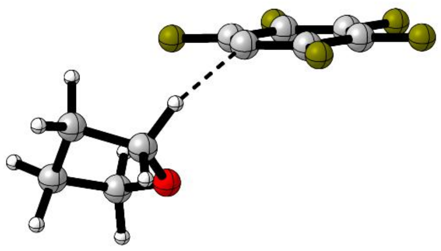

THF
$\boldsymbol{G}_{298}$ = −232.3972183 Hartree

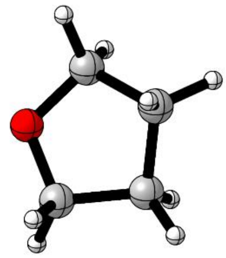

[THF]$^{\bullet}$
$\boldsymbol{G}_{298}$ = −231.7510931 Hartree

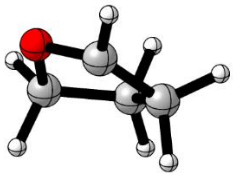

[3]$^{-}$
$\boldsymbol{G}_{298}$ = −1673.606519 Hartree

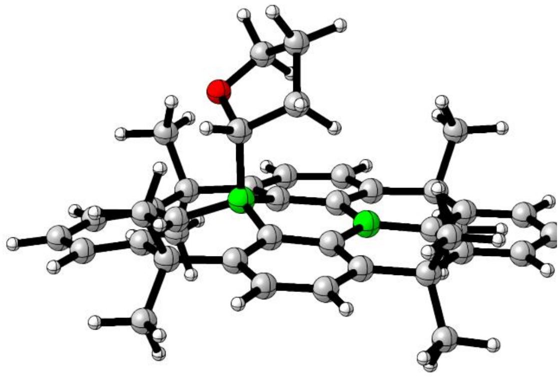

[3]$^{2-}$
$\boldsymbol{G}_{298}$ = −1673.649311 Hartree

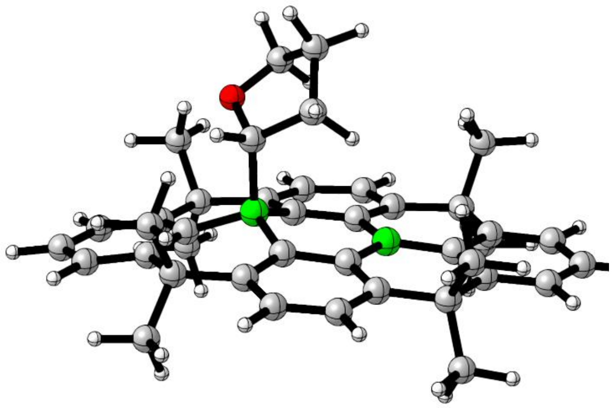

8 References

(S1) Byrne, F.; Forier, B.; Bossaert, G.; Hoebers, C.; Farmer, T. J.; Clark, J. H.; Hunt, A. J. 2,2,5,5-Tetramethyltetrahydrofuran (TMTHF): A Non-Polar, Non-Peroxide Forming Ether Replacement for Hazardous Hydrocarbon Solvents. *Green Chem.* **2017**, *19* (15), 3671–3678. https://doi.org/10.1039/c7gc01392b.

(S2) Zhou, Z.; Wakamiya, A.; Kushida, T.; Yamaguchi, S. Planarized Triarylboranes: Stabilization by Structural Constraint and Their Plane-to-Bowl Conversion. *J. Am. Chem. Soc.* **2012**, *134* (10), 4529–4532. https://doi.org/10.1021/ja211944q.

(S3) Fulmer, G. R.; Miller, A. J. M.; Sherden, N. H.; Gottlieb, H. E.; Nudelman, A.; Stoltz, B. M.; Bercaw, J. E.; Goldberg, K. I. NMR Chemical Shifts of Trace Impurities: Common Laboratory Solvents, Organics, and Gases in Deuterated Solvents Relevant to the Organometallic Chemist. *Organometallics* **2010**, *29* (9), 2176–2179. https://doi.org/10.1021/om100106e.

(S4) (a) Hoffend, C.; Diefenbach, M.; Januszewski, E.; Bolte, M.; Lerner, H.-W.; Holthausen, M. C.; Wagner, M. Effects of Boron Doping on the Structural and Optoelectronic Properties of 9,10-Diarylanthracenes. *Dalt. Trans.* **2013**, *42* (38), 13826–13837. https://doi.org/10.1039/c3dt51035b. For the assessment of the electronic structure by ^{13}C NMR spectroscopy, see: (b) O'Brien, D. H.; Hart, A. J.; Russell, C. R. Carbon-13 Magnetic Resonance of Allyl, Pentadienyl, and Arylmethyl Carbanions. Empirical Calculation of π-Electron Densities. J. Am. Chem. Soc. **1975**, *97* (15), 4410–4412. https://doi.org/10.1021/ja00848a054; (c) Mills, N. S.; Burns, E. E.; Hodges, J.; Gibbs, J.; Esparza, E.; Malandra, J. L.; Koch, J. Dications of Fluorenylidenes. Electronic Effects on the Paratropicity/Antiaromaticity of 2,7-Disubstituted Fluorenyl Cations. J. Org. Chem. **1998**, *63* (9), 3017–3022. https://doi.org/10.1021/jo972202s; (d) Shenhar, R.; Beust, R.; Hoffman, R. E.; Willner, I.; Bronstein, H. E.; Scott, L. T.; Rabinovitz, M. Lithium Reduction of the Bowl-Shaped C_{60} Fragment Di-indeno[1,2,3,4-defg; 1‘,2‘,3‘,4‘-mnop]Chrysene: An Interplay between Experiment and Calculation. J. Org. Chem. **2001**, *66* (18), 6004–6013. https://doi.org/10.1021/jo010103f. For references discussing changes in ^{13}C NMR shifts and/or bond lengths upon one- or two-electron reduction of B-PAHs, see: (e) Gilmer, J.; Budy, H.; Kaese, T.; Bolte, M.; Lerner, H. W.; Wagner, M. The 9H-9-Borafluorene Dianion: A Surrogate for Elusive Diarylboryl Anion Nucleophiles. *Angew. Chem. - Int. Ed.* **2020**, *59* (14), 5621–5625. https://doi.org/10.1002/anie.201914219; (f) Budy, H.; Kaese, T.; Bolte, M.; Lerner, H.; Wagner, M. A Chemiluminescent Tetraaryl Diborane(4) Tetraanion. *Angew. Chem. Int. Ed.* **2021**, *60* (35), 19397–19405. https://doi.org/10.1002/anie.202106980; (g) Metzler, M.; Virovets, A.; Lerner, H.-W.; Wagner, M. B2,N4-Doped Heptacenes: Ambipolar Charge-Transfer Compounds with Deep LUMO Levels. *J. Am. Chem. Soc.* **2023**, *145* (43), 23824–23831. https://doi.org/10.1021/jacs.3c09029.

(S5) Prey, S. E.; Herok, C.; Fantuzzi, F.; Bolte, M.; Lerner, H. W.; Engels, B.; Wagner, M. Multifaceted Behavior of a Doubly Reduced Arylborane in B-H-Bond Activation and Hydroboration Catalysis. *Chem. Sci.* **2023**, *14* (4), 849–860. https://doi.org/10.1039/d2sc05518j.

(S6) Kai, Y.; Oku, S.; Tani, T.; Sakurai, K.; Tsuchimoto, T. A Drastic Effect of TEMPO in Zinc-Catalyzed Stannylation of Terminal Alkynes with Hydrostannanes via Dehydrogenation and Oxidative Dehydrogenation. *Adv. Synth. Catal.* **2019**, *361* (18), 4314–4323. https://doi.org/10.1002/adsc.201900540.

(S7) Rosenau, C. P.; Jelier, B. J.; Gossert, A. D.; Togni, A. Exposing the Origins of Irreproducibility in Fluorine NMR Spectroscopy. *Angew. Chemie - Int. Ed.* **2018**, *57* (30), 9528–9533. https://doi.org/10.1002/anie.201802620.

(S8) Orbach, M.; Choudhury, J.; Lahav, M.; Zenkina, O. V.; Diskin-Posner, Y.; Leitus, G.; Iron, M. A.; van der Boom, M. E. Palladium-Catalyzed Cross-Coupling Reactions with Fluorinated Substrates: Mechanistic Insights into the Undesired Hydrodehalogenation of Aryl Halides. *Organometallics* **2012**, *31* (4), 1271–1274. https://doi.org/10.1021/om200898t.

(S9) Berger, S.; Etten, R. L.; Risley, J. M.; Sergeyev, N. M. *Isotope Effects in NMR Spectroscopy*; 1990.

(S10) Lu, J.; Khetrapal, N. S.; Johnson, J. A.; Zeng, X. C.; Zhang, J. “π-Hole−π” Interaction Promoted Photocatalytic Hydrodefluorination via Inner-Sphere Electron Transfer. *J. Am. Chem. Soc.* **2016**, *138* (49), 15805–15808. https://doi.org/10.1021/jacs.6b08620.

(S11) Doster, M. E.; Johnson, S. A. Selective C-F Bond Activation of Tetrafluorobenzenes by Nickel(0) with a Nitrogen Donor Analogous to N-Heterocyclic Carbenes. *Angew. Chemie Int. Ed.* **2009**, *48* (12), 2185–2187. https://doi.org/10.1002/anie.200806048.

(S12) Budy, H.; Prey, S. E.; Buch, C. D.; Bolte, M.; Lerner, H.-W.; Wagner, M. Nucleophilic Borylation of Fluorobenzenes with Reduced Arylboranes. *Chem. Commun.* **2022**, *58* (2), 254–257. https://doi.org/10.1039/d1cc06225e.

(S13) Sheldrick, G. M. SHELXT – Integrated Space-Group and Crystal-Structure Determination. *Acta Crystallogr. Sect. A Found. Adv.* **2015**, *71* (1), 3–8. https://doi.org/10.1107/S2053273314026370.

(S14) Sheldrick, G. M. A Short History of SHELX. *Acta Crystallogr. Sect. A Found. Crystallogr.* **2008**, *64* (1), 112–122. https://doi.org/10.1107/S0108767307043930.

(S15) Sheldrick, G. M. Crystal Structure Refinement with SHELXL. *Acta Crystallogr. Sect. C Struct. Chem.* **2015**, *71* (1), 3–8. https://doi.org/10.1107/S2053229614024218.

(S16) M. J. Frisch, G. W. Trucks, H. B. Schlegel, G. E. Scuseria, M. A. Robb, J. R. Cheeseman, G. Scalmani, V. Barone, G. A. Petersson, H. Nakatsuji, X. Li, M. Caricato, A. V. Marenich, J. Bloino, B. G. Janesko, R. Gomperts, B. Mennucci, H. P. Hratchian, J. V. Gaussian 16, Revision B.01. **2016**.

(S17) Legault, C. Y. CYLview, 1.0b. *CYLview, 1.0b* **2009**.

(S18) Roy Dennington, Todd Keith, and John Millam, Semichem Inc., S. M. GaussView, Version 6.1.1. **2019**.

(S19) Marenich, A. V.; Cramer, C. J.; Truhlar, D. G. Universal Solvation Model Based on Solute Electron Density and on a Continuum Model of the Solvent Defined by the Bulk Dielectric Constant and Atomic Surface Tensions. *J. Phys. Chem. B* **2009**, *113* (18), 6378–6396. https://doi.org/10.1021/jp810292n.

(S20) Grimme, S.; Antony, J.; Ehrlich, S.; Krieg, H. A Consistent and Accurate Ab Initio Parametrization of Density Functional Dispersion Correction (DFT-D) for the 94 Elements H-Pu. *J. Chem. Phys.* **2010**, *132* (15), 154104. https://doi.org/10.1063/1.3382344.

(S21) Grimme, S.; Ehrlich, S.; Goerigk, L. Effect of the Damping Function in Dispersion Corrected Density Functional Theory. *J. Comput. Chem.* **2011**, *32* (7), 1456–1465. https://doi.org/10.1002/jcc.21759.

(S22) Perdew, J. P. Density-Functional Approximation for the Correlation Energy of the Inhomogeneous Electron Gas. *Phys. Rev. B* **1986**, *33* (12), 8822–8824. https://doi.org/10.1103/PhysRevB.33.8822.

(S23) Becke, A. D. Density-Functional Exchange-Energy Approximation with Correct

Asymptotic Behavior. *Phys. Rev. A* **1988**, *38* (6), 3098–3100. https://doi.org/10.1103/PhysRevA.38.3098.

(S24) Zhao, Y.; Truhlar, D. G. The M06 Suite of Density Functionals for Main Group Thermochemistry, Thermochemical Kinetics, Noncovalent Interactions, Excited States, and Transition Elements: Two New Functionals and Systematic Testing of Four M06-Class Functionals and 12 Other Function. *Theor. Chem. Acc.* **2008**, *120* (1–3), 215–241. https://doi.org/10.1007/s00214-007-0310-x.

(S25) Ernzerhof, M.; Scuseria, G. E. Assessment of the Perdew–Burke–Ernzerhof Exchange-Correlation Functional. *J. Chem. Phys.* **1999**, *110* (11), 5029–5036. https://doi.org/10.1063/1.478401.

(S26) Adamo, C.; Barone, V. Toward Reliable Density Functional Methods without Adjustable Parameters: The PBE0 Model. *J. Chem. Phys.* **1999**, *110* (13), 6158–6170. https://doi.org/10.1063/1.478522.

(S27) Chai, J.-D.; Head-Gordon, M. Long-Range Corrected Hybrid Density Functionals with Damped Atom–Atom Dispersion Corrections. *Phys. Chem. Chem. Phys.* **2008**, *10* (44), 6615–6620. https://doi.org/10.1039/b810189b.

(S28) Weigend, F.; Ahlrichs, R. Balanced Basis Sets of Split Valence, Triple Zeta Valence and Quadruple Zeta Valence Quality for H to Rn: Design and Assessment of Accuracy. *Phys. Chem. Chem. Phys.* **2005**, *7* (18), 3297–3305. https://doi.org/10.1039/b508541a.

(S29) Gilmer, J.; Trageser, T.; Čaić, L.; Virovets, A.; Bolte, M.; Lerner, H. W.; Fantuzzi, F.; Wagner, M. Catalyst-Free Diboration and Silaboration of Alkenes and Alkynes Using Bis(9-Heterofluorenyl)S. *Chem. Sci.* **2023**, *14* (17), 4589–4596. https://doi.org/10.1039/d3sc01395b.

(S30) Prey, S. E.; Gilmer, J.; Teichmann, S. V.; Čaić, L.; Wenisch, M.; Bolte, M.; Virovets, A.; Lerner, H. W.; Fantuzzi, F.; Wagner, M. Synthesis, Bridgehead Functionalization, and Photoisomerization of 9,10-Diboratatriptycene Dianions. *Chem. Sci.* **2023**, *14* (20), 5316–5322. https://doi.org/10.1039/d3sc00555k.

(S31) Kelly, C. P.; Cramer, C. J.; Truhlar, D. G. SM6: A Density Functional Theory Continuum Solvation Model for Calculating Aqueous Solvation Free Energies of Neutrals, Ions, and Solute−Water Clusters. *J. Chem. Theory Comput.* **2005**, *1* (6), 1133–1152. https://doi.org/10.1021/ct050164b.

(S32) Sparta, M.; Riplinger, C.; Neese, F. Mechanism of Olefin Asymmetric Hydrogenation Catalyzed by Iridium Phosphino-Oxazoline: A Pair Natural Orbital Coupled Cluster Study. *J. Chem. Theory Comput.* **2014**, *10* (3), 1099–1108. https://doi.org/10.1021/ct400917j.

(S33) Hou, X. J.; Huang, M. B. Structure of the Hexafluorobenzene Anion. *J. Mol. Struct. THEOCHEM* **2003**, *638* (1–3), 209–214. https://doi.org/10.1016/S0166-1280(03)00583-9.

(S34) Voora, V. K.; Jordan, K. D. Nonvalence Correlation-Bound Anion State of C_6F_6: Doorway to Low-Energy Electron Capture. *J. Phys. Chem. A* **2014**, *118* (35), 7201–7205. https://doi.org/10.1021/jp408386f.

(S35) Shchegoleva, L. N.; Beregovaya, I. V.; Schastnev, P. V. Potential Energy Surface of $C_6F_6^-$ Radical Anion. *Chem. Phys. Lett.* **1999**, *312* (2–4), 325–332. https://doi.org/10.1016/S0009-2614(99)00944-6.

(S36) Carpenter, J. E.; Weinhold, F. Analysis of the Geometry of the Hydroxymethyl Radical

by the “Different Hybrids for Different Spins” Natural Bond Orbital Procedure. *J. Mol. Struct. THEOCHEM* **1988**, *169*, 41–62. https://doi.org/10.1016/0166-1280(88)80248-3.

(S37) Foster, J. P.; Weinhold, F. Natural Hybrid Orbitals. *J. Am. Chem. Soc.* **1980**, *102* (24), 7211–7218. https://doi.org/10.1021/ja00544a007.

(S38) *The Structure of Small Molecules and Ions*; Naaman, R., Vager, Z., Eds.; Springer US: Boston, MA, 1988. https://doi.org/10.1007/978-1-4684-7424-4.

(S39) McGee, C. J.; McGinnis, K. R.; Jarrold, C. C. Anion Photoelectron Imaging Spectroscopy of $C_6HF_5^-$, $C_6F_6^-$, and the Absence of $C_6H_2F_4^-$. *J. Phys. Chem. A* **2023**, *127* (41), 8556–8565. https://doi.org/10.1021/acs.jpca.3c04016.

(S40) Song, H.; Guo, H. Theoretical Insights into the Dynamics of Gas-Phase Bimolecular Reactions with Submerged Barriers. *ACS Phys. Chem. Au* **2023**, *3* (5), 406–418. https://doi.org/10.1021/acsphyschemau.3c00009.

(S41) Shiels, O. J.; Marlton, S. J. P.; Poad, B. L. J.; Blanksby, S. J.; da Silva, G.; Trevitt, A. J. Gas-Phase Phenyl Radical + O_2 Reacts via a Submerged Transition State. *J. Phys. Chem. A* **2024**, *128* (2), 413–419. https://doi.org/10.1021/acs.jpca.3c06878.

(S42) Maeda, S.; Harabuchi, Y.; Ono, Y.; Taketsugu, T.; Morokuma, K. Intrinsic Reaction Coordinate: Calculation, Bifurcation, and Automated Search. *Int. J. Quantum Chem.* **2015**, *115* (5), 258–269. https://doi.org/10.1002/qua.24757.

(S43) Würmel, J.; Simmie, J. M. H-Atom Abstraction Reactions by Ground-State Ozone from Saturated Oxygenates. *J. Phys. Chem. A* **2017**, *121* (42), 8053–8060. https://doi.org/10.1021/acs.jpca.7b07760.

(S44) El-Sheshtawy, H. S.; Pischel, U.; Nau, W. M. Solvent Polarity Affects H Atom Abstractions from C–H Donors. *Org. Lett.* **2011**, *13* (10), 2694–2697. https://doi.org/10.1021/ol2007956.
